# EOMES and IL-10 regulate antitumor activity of T regulatory type 1 CD4^+^ T cells in chronic lymphocytic leukemia

**DOI:** 10.1038/s41375-021-01136-1

**Published:** 2021-02-01

**Authors:** Philipp M. Roessner, Laura Llaó Cid, Ekaterina Lupar, Tobias Roider, Marie Bordas, Christoph Schifflers, Lavinia Arseni, Ann-Christin Gaupel, Fabian Kilpert, Marit Krötschel, Sebastian J. Arnold, Leopold Sellner, Dolors Colomer, Stephan Stilgenbauer, Sascha Dietrich, Peter Lichter, Ana Izcue, Martina Seiffert

**Affiliations:** 1 Division of Molecular Genetics, German Cancer Research Center (DKFZ), Heidelberg, Germany; 2 Faculty of Biosciences, University of Heidelberg, Heidelberg, Germany; 3 Max-Planck-Institute of Immunobiology and Epigenetics, Freiburg, Germany; 4 Department of Medicine V, Hematology, Oncology and Rheumatology, University of Heidelberg, Heidelberg, Germany; 5 Institute of Experimental and Clinical Pharmacology and Toxicology, Faculty of Medicine, University of Freiburg, Freiburg, Germany; 6 Signalling Research Centres BIOSS and CIBSS, University of Freiburg, Freiburg, Germany; 7 Institut d’Investigacions Biomèdiques August Pi i Sunyer (IDIBAPS), Hematopathology Unit, Hospital Clinic, CIBERONC, Barcelona, Spain; 8 Department of Internal Medicine III, University of Ulm, Ulm, Germany; 9 Center for Chronic Immunodeficiency, University Medical Center Freiburg and University of Freiburg, Freiburg, Germany; 10 Institute of Molecular Medicine, University Hospital RWTH Aachen, Aachen, Germany; 11 Present Address: Cellzome, Heidelberg, Germany; 12 Present Address: Cell Biology Research Unit (URBC)—Namur Research Institute of Life Science (Narilis), University of Namur, Namur, Belgium; 13 Present Address: Immunotherapy and Immunoprevention, German Cancer Research Center (DKFZ), Heidelberg, Germany; 14 Present Address: Essen University Hospital, Institute of Human Genetics, Genome Informatics, Essen, Germany; 15 Present Address: BioMed X Institute, Heidelberg, Germany

**Keywords:** Cancer microenvironment, CD4-positive T cells, Immunosurveillance, Chronic lymphocytic leukaemia, Non-hodgkin lymphoma

## Abstract

The transcription factor eomesodermin (EOMES) promotes interleukin (IL)-10 expression in CD4^+^ T cells, which has been linked to immunosuppressive and cytotoxic activities. We detected cytotoxic, programmed cell death protein-1 (PD-1) and EOMES co-expressing CD4^+^ T cells in lymph nodes (LNs) of patients with chronic lymphocytic leukemia (CLL) or diffuse large B-cell lymphoma. Transcriptome and flow cytometry analyses revealed that EOMES does not only drive IL-10 expression, but rather controls a unique transcriptional signature in CD4^+^ T cells, that is enriched in genes typical for T regulatory type 1 (T_R_1) cells. The T_R_1 cell identity of these CD4^+^ T cells was supported by their expression of interferon gamma and IL-10, as well as inhibitory receptors including PD-1. T_R_1 cells with cytotoxic capacity accumulate also in Eµ-TCL1 mice that develop CLL-like disease. Whereas wild-type CD4^+^ T cells control TCL1 leukemia development after adoptive transfer in leukopenic *Rag2*^*−/*^^−^ mice, EOMES-deficient CD4^+^ T cells failed to do so. We further show that T_R_1 cell-mediated control of TCL1 leukemia requires IL-10 receptor (IL-10R) signaling, as *Il10rb*-deficient CD4^+^ T cells showed impaired antileukemia activity. Altogether, our data demonstrate that EOMES is indispensable for the development of IL-10-expressing, cytotoxic T_R_1 cells, which accumulate in LNs of CLL patients and control TCL1 leukemia in mice in an IL-10R-dependent manner.

## Introduction

Despite abundant phenotypical data characterizing CD4^+^ T cells in chronic lymphocytic leukemia (CLL), their role in disease development and progression is controversial and poorly understood [1]. Besides the well-known T helper (Th) cell subsets [1], interleukin (IL-)10-producing, FOXP3^−^ CD4^+^ T cells, named T regulatory type 1 (T_R_1) cells, are lately gaining attention in chronic inflammatory diseases and cancer [2–5]. T_R_1 cells are described as IL-10-induced cells that produce IL-10 and harbor cytotoxic activity, but also express several co-inhibitory receptors such as programmed cell death protein-1 (PD-1) [5].

Increased expression of PD-1 in blood-derived CD4^+^ T cells has been reported for CLL [1, 6–8] and other B-cell non-Hodgkin lymphomas (B-NHL), including diffuse large B-cell lymphoma (DLBCL) [9, 10]. Yet, the role of these PD-1^+^ CD4^+^ T cells in CLL has not been studied. Blockade of PD-1 or its ligand PD-L1, with the aim to enhance CD8^+^ T cell-mediated antitumoral immunity, resulted in good response rates in the Eµ-TCL1 mouse model of CLL [11, 12]. However, clinical trials using immune checkpoint inhibitors targeting the PD-1/PD-L1 axis lead to disappointing results. None of the included CLL patients achieved remission in response to therapy and only a subgroup of patients, harboring a more aggressive Richter’s transformation, benefitted from this treatment (NCT02332980) [13]. It was hypothesized that the lack of clinical success of PD-1 blockade in CLL was due to the fact that tumor-infiltrating T cells in CLL have a lower expression of PD-1 in comparison to other B-NHL entities, including DLBCL [10]. In DLBCL, PD-1 expression was shown to correlate with better survival [14, 15]. However, immune checkpoint blockade resulted in an overall response rate of only about 10% in DLBCL patients (NCT02038933) [16].

Recently, the transcription factor eomesodermin (EOMES) has been shown to promote IL-10 production of T_R_1 cells together with other factors, like PR domain zinc finger protein 1 (BLIMP1), both in mice and humans [2–4]. EOMES belongs to the T-box transcription factor family, which is expressed in many organs including the immune system [17]. Redundantly with its paralogue T-BET, EOMES has been shown to promote interferon gamma (IFNγ) production and cytotoxicity in natural killer (NK) cells [18–20], CD8^+^ [19–21], and CD4^+^ T cells [2, 3, 19, 22, 23]. EOMES has also a nonredundant role in promoting maturation of classical NK cells [24], and in the accumulation of central memory [20, 25] and exhausted, PD-1-expressing CD8^+^ T cells [26]. Co-expression of PD-1 and EOMES in CD8^+^ T cells was recently shown by us in the Eµ-TCL1 adoptive transfer (TCL1 AT) mouse model of CLL [27].

In contrast to NK cells and CD8^+^ T lymphocytes, very few CD4^+^ T cells express EOMES without immunological challenge. However, we and others have shown that EOMES can be upregulated in CD4^+^ T cells upon their activation, which affects their differentiation into helper cell lineages [28]. Moreover, EOMES promotes IFNγ expression by Th1 T cells [29–31] and inhibits differentiation of Th17 [3, 31, 32] and FOXP3^+^ regulatory T cells (Treg) [28].

Here, we observed the accumulation of an EOMES-expressing PD-1^+^ CD4^+^ T-cell subset in CLL patients and mouse models, which we identify as T_R_1 cells. We further show that these cells harbor cytotoxic function and are able to control leukemia development in mice in an EOMES- and IL-10 receptor (IL-10R)-dependent way.

## Methods

### Patient samples

Patient samples were obtained after approval of study protocols by local ethics committees from the Department of Internal Medicine III of the University Clinic Ulm, the Department of Medicine V of the University Clinic Heidelberg, and the Hospital Clinic of Barcelona according to the declaration of Helsinki, and after obtaining informed consent of patients. Patients met standard diagnosis criteria for CLL or DLBCL, respectively. Patient characteristics such as age, mutational state, and Binet stage are provided in Supplementary Tables 1–3. Healthy, age-matched controls were obtained from Biomex GmbH (Heidelberg, Germany) after informed consent.

### Tumor models and adoptive CD4^+^ T-cell transfer

Adoptive transfer of mouse leukemic cells was performed by i.p. or i.v. transplantation of 1–2 × 10^7^ Eμ-TCL1 splenocytes into C57BL/6N or J wild-type (WT) female animals of 6–8 weeks of age. For CD4^+^ co-transfer experiments, female *Rag2*^*−/−*^ mice of 6–8 weeks of age were i.v. transplanted with 2 × 10^5^ CD4^+^ T cells or PBS as control. The following day, 1 × 10^6^ purified TCL1 leukemic cells were transferred i.v. into recipients.

Adoptive transfer of naive CD4^+^ T cells was performed as previously described [28]. In brief, *Rag2*^*−/−*^ recipient mice received 4 × 10^5^ FACS-sorted CD4^+^CD45RB^high^ T cells by i.p. injection. Three weeks post transfer, mice were sacrificed, and spleens were analyzed. A list of antibodies used in this study is available in Supplementary Table 4.

All animal experiments were carried out according to institutional and governmental guidelines approved by the local authorities (Regierungspräsidium Karlsruhe, permit numbers: G36/14, G98/16, G123/14, and Regierungspräsidium Freiburg, permit number: 35-9185.81/G-13/73).

### Statistical analysis

Sample size was determined based on expected variance of readout. No samples or animals were excluded from the analyses. No randomization or blinding was used in animal studies. The statistical test used for each data set is indicated in the figure legends. Samples of different groups were compared using nonparametric Mann–Whitney test. Comparison of matched samples was performed using Wilcoxon matched-pairs signed rank test. Values of *p* < 0.05 were considered statistically significant. All graphs show means ± standard error of the mean (SEM), unless otherwise indicated.

## Results

### Cytotoxic EOMES^+^ PD-1^+^ T_R_1-like CD4^+^ T cells accumulate in lymph nodes (LNs) of CLL patients

According to published data [1, 6, 8–10, 33–35], we observed an accumulation of PD-1-expressing CD4^+^ T cells in blood samples of patients with CLL or DLBCL in comparison to healthy controls (HC) (Supplementary Fig. 1A–E), and subsequently aimed to characterize these cells. Since PD-1 has been shown to be expressed by T_R_1 cells [5], we investigated the frequency of EOMES and PD-1 co-expressing T_R_1-like cells and observed no difference in the frequency of these cells in the blood of CLL patients and controls (Supplementary Fig. 1F). In line, no substantial changes in the frequency of EOMES^+^ PD-1^+^ T_R_1-like cells were observed in sequential blood samples of CLL patients over 5 years (Supplementary Fig. 1G). Since T_R_1 cells were shown to exert cytotoxic functions [5], we analyzed the cytotoxic molecule granzyme B (GzmB), the degranulation marker CD107a, and IFNγ in PD-1^+^ versus PD-1^−^ CD4^+^ T cells. This clearly showed that PD-1^+^ cells have a higher cytotoxic capacity compared to PD-1^−^ cells in both CLL patients and controls (Supplementary Fig. 2A–F) confirming that PD-1-expressing CD4^+^ T cells have cytotoxic capacity, also in CLL.

**Fig. 1:**
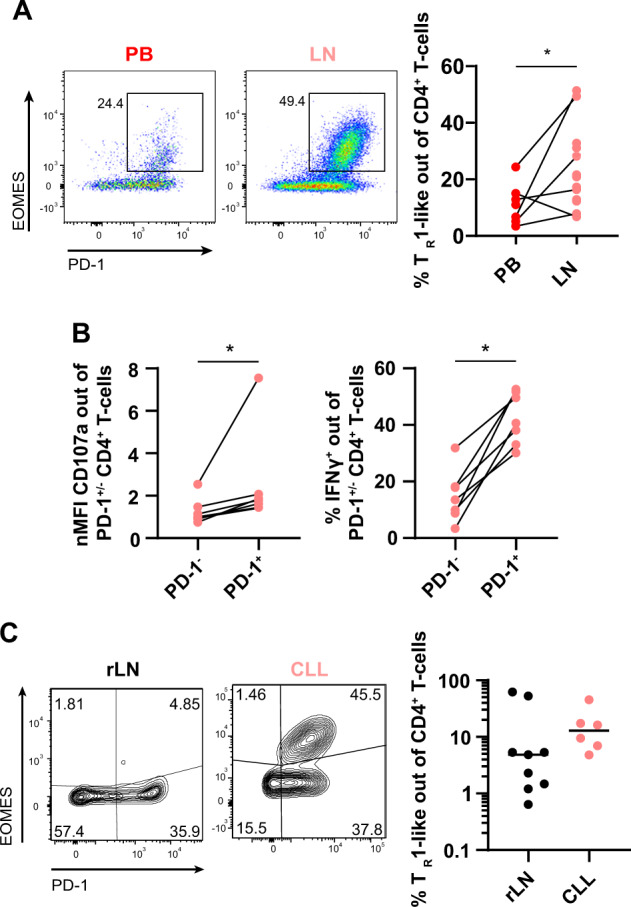
EOMES^+^ PD-1^+^ T_R_1-like cells with cytotoxic function accumulate in CLL lymph node samples. **A** Paired PB and lymph node (LN) samples of patients with CLL were analyzed by flow cytometry. Representative dot plots as well as frequency of Eomes^+^ PD-1^+^ T_R_1-like cells. **B** LN samples of CLL patients were stimulated with PMA/ionomycin ex vivo. nMFI of CD107a, and frequency of IFNγ^+^ cells of PD-1^+^ or PD-1^−^ CD4^+^ T cells. **C** Representative contour plots and frequency of EOMES^+^ PD-1^+^ T_R_1-like cells out of CD4^+^ T cells in reactive, nonpathogenic lymph nodes (rLN) and CLL patient LN samples. Each dot represents data of an individual patient. Data derived from paired samples are connected by a line. In (**C**), median of data is depicted. Statistical analysis was performed using Mann–Whitney test. Comparison of matched samples was performed using Wilcoxon matched-pairs signed rank test *p < 0.05. nMFI = normalized median fluorescence intensity.

As we and others have recently highlighted clear differences in the phenotype of T cells in peripheral blood compared to LNs in CLL, with a phenotype of T-cell activation and exhaustion only in the tissue but not in blood [34, 36], we investigated the abundance and frequency of EOMES^+^ PD-1^+^ T_R_1-like cells in secondary lymphoid organs (SLO) of patients. Intriguingly, we detected a higher number of CD4^+^ T cells per CLL cell in LNs compared to paired blood samples (Supplementary Fig. 2G), and a significantly higher frequency of T_R_1-like cells in LNs (Fig. 1A), which moreover revealed a higher activation state based on CD69 and HLA-DR expression (Supplementary Fig. 2H). These results suggest that T_R_1-like cells are of pathological relevance in SLO of patients with CLL.

We further analyzed the transcription factors interferon regulatory factor 4 (IRF4) and basic leucine zipper ATF-like transcription factor (BATF), which were shown to be involved in the development of T_R_1 cells [5], in paired blood and LN samples of CLL patients. We observed a higher expression of IRF4 and BATF in PD-1^+^ CD4^+^ T cells in LN compared to blood (Supplementary Fig. 2I, J), supporting the results of an increased frequency of T_R_1-like cells in SLO of CLL patients. By analyzing the cytotoxic proficiency of T cells, we observed a higher CD107a and IFNγ expression in PD-1^+^ compared to PD-1^−^ cells in the LN (Fig. 1B), which is in line with our observation in blood and our hypothesis of a pathological relevance of T_R_1-like cells.

Ultimately, we aimed to compare the frequency of T_R_1-like cells in reactive, nonmalignant LNs (rLNs) versus CLL and DLBCL LNs. We detected a substantial proportion of T_R_1-like cells in all investigated CLL (median: 12.90%) and DLBCL (median: 14.75%) LN samples. In contrast, the majority of rLNs had a lower frequency (median: 4.85%) of this cell type (Fig. 1C and Supplementary Fig. 2K).

Hence, T_R_1-like CD4^+^ T cells show cytotoxic abilities and accumulate in LNs of CLL and DLBCL patients, making their potential pathological involvement in these malignancies very likely.

### EOMES controls a T_R_1 cell-specific gene signature including inhibitory receptors in CD4^+^ T cells

**Fig. 2:**
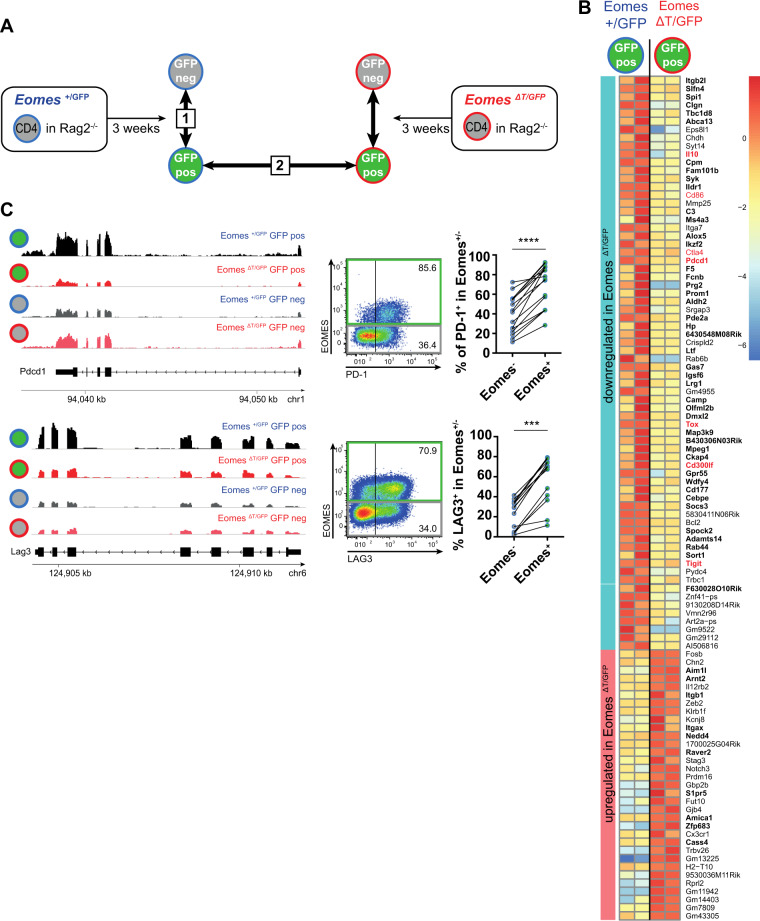
EOMES controls a T_R_1 cell-specific gene signature including inhibitory receptors in CD4^+^ T cells. Naive CD25^−^ CD45RB^high^ CD4^+^ T cells of *Eomes-GFP* reporter mice (*Eomes*^*+/GFP*^), and of mice with a T cell-specific deletion of *Eomes* in combination with a GFP reporter (*Eomes*^*ΔT/GFP*^) were transferred into *Rag2*^*−/−*^ mice. Three weeks after adoptive transfer, RNA sequencing of sorted splenic GFP^+^ and GFP^−^ CD4^+^ T-cell subsets was performed. **A** Analysis strategy: comparison 1 reveals transcriptional differences between GFP^+^ T_R_1 cells and GFP^−^ CD4^+^ T cells from *Eomes*^*+/GFP*^ donor mice; comparison 2 shows differential gene expression between EOMES-proficient (*Eomes*^*+/GFP*^) and EOMES-deficient (*Eomes*^*ΔT/GFP*^) GFP^+^ CD4^+^ T cells. **B** Heatmap of log2 normalized expression of EOMES-dependent genes (comparison 2). T-cell receptor transcripts were excluded from the heatmap. Bold font shows genes which are also differentially expressed in comparison 1. Red highlights genes of interest. **C** Representative RNA-seq tracks and flow cytometry plots of PD-1 and LAG3 expression in *Eomes-*GFP^+^ and *Eomes-*GFP^−^ CD4^+^ T cells, and quantification of percentage PD-1- or LAG3-expressing cells out of GFP^+^ or GFP^−^ cells. Each dot represents data of an individual mouse. Lines link data of EOMES^−^ and EOMES^+^ cells from the same animal. Statistical analysis of matched samples in (**C**) was performed using Wilcoxon matched-pairs signed rank test. ***p < 0.001; ****p < 0.0001.

In order to understand the role of EOMES in T_R_1 cells, we turned to animal models and took advantage of *Eomes-*GFP reporter mice (*Eomes*^*+/GFP*^) in which *Eomes*-expressing CD4^+^ T cells can be identified as GFP-positive cells [37]. As we have previously demonstrated that adoptive transfer of naive CD4^+^ T cells into lymphopenic *Rag2*^−/−^ hosts leads to an accumulation of EOMES^+^ PD-1^+^ cells with T_R_1 phenotype [28], we transferred CD4^+^ T cells of *Eomes-*GFP reporter mice into *Rag2*^−/−^ mice, and 3 weeks later, sorted GFP-positive and GFP-negative CD4^+^ T cells of these mice for RNA sequencing. This allowed us to identify a transcriptional signature of 1,048 genes, with 568 upregulated and 480 downregulated genes, in *Eomes-*GFP-positive versus *Eomes-*GFP-negative CD4^+^ T cells (Fig. 2A, comparison 1, and Supplementary Tables 5 and 6). As expected, one of the top upregulated genes was *Eomes* itself, validating our experimental approach. Among the top upregulated genes in GFP^+^ cells, we identified several molecules that were associated with a T_R_1 phenotype in mice and men, such as *Il10*, *Il10ra*, *Pdcd1* (encoding PD-1), *Lag3*, *Tigit*, and *Cd27* (Supplementary Table 6) [5]. This supports the validity of EOMES as a marker for T_R_1 cells. Among the top downregulated genes in GFP^+^ cells, we detected *Foxp3* (Supplementary Table 6), which is in line with our previous findings of an antagonistic relationship between *Eomes* and *Foxp3* [28].

To analyze the relevance of EOMES in regulating T_R_1 cell identity, we included a *Eomes* knock-out mouse model *(Eomes*^*ΔT*/GFP^) in which one allele of the *Eomes* gene is disrupted by a GFP knock-in, while the second allele lacks exons 2–5, which encode the DNA-binding domain of EOMES. The truncated mRNA and protein of this second *Eomes* allele are expressed and detectable in these mice (Supplementary Fig. 3A), but EOMES is dysfunctional and cannot induce target genes [38]. Hence, GFP expression in these mice identifies cells with active *Eomes* transcription, but lack of EOMES activity. We used the same adoptive transfer approach of CD4^+^ T cells in *Rag2*^−/−^ hosts as described above, and performed RNA sequencing of sorted GFP^+^ and GFP^−^ CD4^+^ T cells 3 weeks after the transfer. This allowed us to compare gene expression profiles of EOMES-proficient and EOMES-deficient CD4^+^ T cells upon activation of *Eomes* transcription, and thereby to determine which genes are transcriptionally regulated by EOMES (Fig. 2A, comparison 2). The results of these comparative analyses showed that 109 genes were dependent on transcriptional activity of EOMES, with 71 genes showing a lower expression in EOMES-deficient cells, which are therefore most likely direct transcriptional targets of EOMES, while 38 genes showed a higher expression in EOMES-deficient cells (Fig. 2B and Supplementary Table 6). Despite a low number of differentially expressed genes, EOMES-deficient GFP^+^ cells from two biological replicates clustered separately from EOMES-proficient GFP^+^ samples on a multidimensional scaling plot (Supplementary Fig. 3B). This suggests that EOMES transcriptional proficiency is indeed required to express a specific transcriptional program. As expected, the GFP-negative samples of both genotypes did not separate well.

Out of these 109 genes, 61 genes were also differentially expressed in comparison 1 representing the transcriptional signature of T_R_1 cells (Supplementary Fig. 3C; genes highlighted in bold in Fig. 2B), indicating that the T_R_1 phenotype is driven partly by EOMES.

EOMES has been previously associated with T-cell exhaustion, and abundant expression of inhibitory receptors is a hallmark of T_R_1 cells [5]. In line with this, we observed that expression of several inhibitory receptor genes, such as *Ctla4*, *Pdcd1*, *Cd300lf*, and *Tigit*, depends on EOMES, and so does the expression of the exhaustion-associated transcription factor *Tox* [39, 40] (genes highlighted in red in Fig. 2B; read alignment for *Pdcd1* and *Lag3* in Fig. 2C). We further validated that EOMES^+^ cells show a higher protein expression of PD-1 and LAG3 in comparison to EOMES^−^ cells (Fig. 2C). Using Ingenuity Pathway Analysis of the differentially expressed genes in comparisons 1 and 2, we identified “neuroinflammation signaling” and “T-cell exhaustion signaling” as the top deregulated pathways in GFP^+^ cells that are controlled by EOMES (Supplementary Fig. 3D). Altogether, our results suggest that EOMES, via its relevance in T_R_1 cells, contributes to pathogenesis of inflammatory diseases and potentially also of cancer, including CLL.

To address the question whether EOMES only promotes transcription of a few specific genes (e.g., inhibitory receptors) or acts as a driver of T_R_1 cells, we developed a marker combination for flow cytometry, which allowed us to enrich for EOMES^+^ CD4^+^ T cells in the *Rag2*^−/−^ adoptive transfer model. We observed that EOMES^+^ cells contain the majority of PD-1^+^ CD44^lo^ cells in these mice (Supplementary Fig. 3E). Interestingly, adoptive transfer of *Eomes*-deficient naive CD4^+^ T cells yielded a significantly lower number of PD-1^+^ CD44^lo^ CD4^+^ T cells compared to transfer of *Eomes*-proficient cells, an effect that was preserved when both *Eomes*-proficient and -deficient T cells were co-transferred into the same *Rag2*^−/−^ recipient (Supplementary Fig. 3F). These results suggest that EOMES does not only activate the expression of several target genes but is rather controlling the accumulation of this specific CD4^+^ T-cell subset. Whereas the marker combination PD-1^+^ CD44^lo^ was suitable to identify EOMES^+^ CD4^+^ T cells in T-cell transfer experiments in *Rag2*^*−/−*^ mice, a respective cell population was not detectable in aged mice (Supplementary Fig. 3E), which argues against the general suitability of this marker combination for identifying T_R_1 cells.

In summary, our transcriptome analyses identify a T_R_1 cell-specific gene signature that includes several inhibitory receptors and is enriched for key T_R_1-associated pathways and markers. We further show that a part of this signature is driven by EOMES.

### EOMES drives IL-10 expression and genes enriched in the human T_R_1 signature

**Fig. 3:**
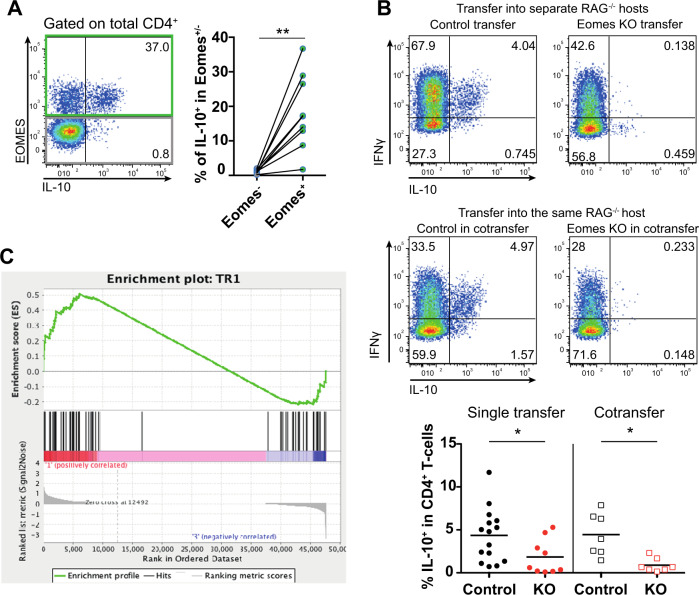
EOMES-expressing CD4^+^ T cells express IL-10 and map with T_R_1 cells. Cells for analyses were prepared as described in Fig. 2. **A** Representative dot plot and quantification of IL-10 production by splenic EOMES^+^ and EOMES^−^ CD4^+^ T cells 3 weeks post transfer into *Rag2*^−/−^ hosts as analyzed by intracellular flow cytometry after stimulation with PMA/ionomycin ex vivo. **B** Congenically labeled EOMES-proficient (Control) or EOMES-deficient (KO) CD4^+^ T cells were transferred into separate, or the same *Rag2*^*−/−*^ hosts, and 3 weeks later, splenocytes were analyzed by flow cytometry. Dot plots show representative expression of IL-10 and IFNγ. Quantification of IL-10-producing CD4^+^ T cells in single transfer and co-transfer experiments is depicted at the bottom. **C** Gene set enrichment analysis plot showing that EOMES-dependent T_R_1 genes (comparison 2 in Fig. 2A) are enriched in human T_R_1 signature genes [3]. Each dot represents data of an individual mouse. Lines in (**A**) link data of EOMES^−^ and EOMES^+^ cells from the same mouse; lines in (**B**) show median. Statistical analysis was performed using Mann–Whitney test. Comparison of matched samples in co-transfer setting was performed using Wilcoxon matched-pairs signed rank test. *p < 0.05, **p < 0.01.

To further investigate pathways controlled by EOMES in T_R_1 cells, we performed KEGG pathway analysis. The top identified pathway map was “cytokine–cytokine receptor interaction,” followed by several pathways in which cytokines are of major importance (Supplementary Fig. 4A). This suggests that EOMES regulates cytokines and cytokine receptors in T_R_1 cells. Along this line, we identified among the upregulated genes in GFP^+^ cells that depend on the presence of EOMES, the T_R_1 signature cytokine *Il10* (Fig. 2B and Supplementary Fig. 4B), which is in agreement with previous reports [2–4]. Transplantation of T cells into *Rag2*^−^^*/−*^ mice confirmed that the majority of IL-10-producing CD4^+^ T cells co-express EOMES (Fig. 3A). Importantly, most IL-10-producing cells also co-expressed IFNγ (Fig. 3B), concordant with previously published T_R_1 descriptions [3, 41]. IL-10/IFNγ co-expression was dependent on EOMES and not on non-cell autonomous factors within the microenvironment, as *Eomes*-deficient CD4^+^ T cells did not produce IL-10, regardless whether they were transferred alone or together with *Eomes*-proficient, CD4^+^ T cells (Fig. 3B).

Finally, we performed gene set enrichment analysis, which showed that the identified EOMES-dependent transcripts in GFP^+^ cells (comparison 2) were enriched in a published human T_R_1 gene signature (Fig. 3C). Beyond underlining the similarity between human and mouse T_R_1 cells, these results confirm that a small subset of EOMES-dependent genes is crucial for T_R_1 cell identity.

### Leukemia development in the Eµ-TCL1 mouse model is associated with an accumulation of cytotoxic T_R_1 cells

**Fig. 4:**
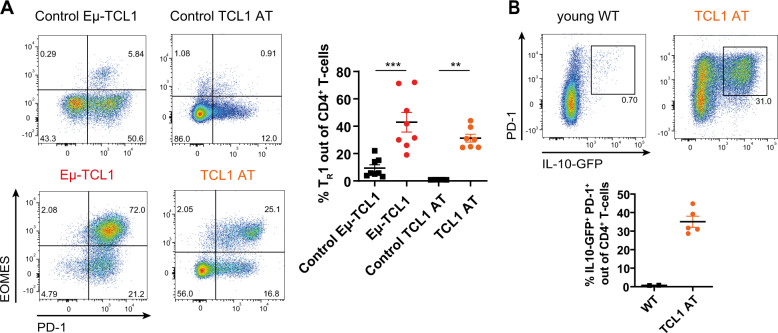
PD-1^+^ CD4^+^ T cells that co-express EOMES are enriched in the Eµ-TCL1 mouse model of CLL. **A** Spleens of hemizygous Eµ-TCL1 leukemic mice and control littermates at a median age of 65 weeks, or animals after transplantation of Eµ-TCL1 leukemic cells into syngeneic wild type (WT) mice (TCL1 AT) were analyzed by flow cytometry. Representative dot plots and frequency of T_R_1 cells (EOMES^+^ PD-1^+^) out of CD4^+^ T cells in Eµ-TCL1 as well as TCL1 AT mice and their respective controls. **B** *FIR* × *tiger* mice were transplanted with Eµ-TCL1 leukemic splenocytes and expression of IL-10-GFP in splenic PD-1^+^ CD4^+^ T cells was analyzed by flow cytometry. Representative dot plots as well as percentage of IL-10-expressing cells out of CD4^+^ T cells in WT versus TCL1 AT *FIR* × *tiger* mice. All graphs show mean ± SEM. Each dot represents data of an individual mouse. Statistical analysis was performed using Mann–Whitney test. **p < 0.01; ***p < 0.001.

To explore the role of T_R_1 cells (defined as EOMES^+^ PD-1^+^) in CLL, we first analyzed their presence in the Eµ-TCL1 (TCL1) mouse model of CLL. Comparing age- and sex-matched leukemic TCL1 mice and WT littermates revealed a higher abundance of T_R_1 cells in the spleen of TCL1 mice (Fig. 4A, gating strategy in Supplementary Fig. 5A). The majority of these EOMES^+^ PD-1^+^ cells also expressed LAG3 (Supplementary Fig. 5B), which we previously showed to be dependent on EOMES. To overcome long latency of CLL development in this mouse model, leukemic splenocytes of TCL1 mice were adoptively transferred into syngeneic WT mice (TCL1 AT), as previously described [34, 42, 43]. Upon leukemia development in the TCL1 AT model, we observed an accumulation of antigen-experienced CD4^+^ T cells (Supplementary Fig. 5C) that show signs of activation as measured by CD69 expression (Supplementary Fig. 5D). Moreover, a higher frequency of T_R_1 CD4^+^ T cells was detected (Fig. 4A), which showed a high co-expression rate of LAG3 (Supplementary Fig. 5B). Interestingly, the frequency of T_R_1 cells was higher in aging Eµ-TCL1 mice compared to the younger TCL1 AT mice (Fig. 4A), which is in line with our previous observation that EOMES^+^ CD4^+^ T cells accumulate with age [28].

Functional investigation of T_R_1 cells in TCL1 AT mice confirmed the phenotype observed in CLL patients, with an induced IL-10 expression that increased over time (Fig. 4B and Supplementary Fig. 5E), and higher levels of IFNγ, CD107a, and GzmB (Supplementary Fig. 5F, G), as markers of cytotoxicity, in these cells. These data show that T_R_1 cells, which produce IL-10 and harbor cytotoxic potential, accumulate in mouse models of CLL.

To investigate the impact of this cell type on CLL progression, we transferred CD4^+^ T cells into *Rag2*^−/−^ mice, which lack mature B and T cells [44] to exclude the contribution of CD8^+^ T cells to leukemia control. Next, the mice were transplanted with TCL1 leukemia cells, as previously described [34, 42, 43]. As reported before in other mouse models, we detected an expansion of CD4^+^ T cells in *Rag2*^*−/*^^−^ mice with and without transfer of leukemic cells (Supplementary Fig. 6A). Interestingly, CD4^+^ T cells controlled leukemia progression, as indicated by lower spleen weight and leukemia cell content per spleen compared to mice without T-cell transfer (Supplementary Fig. 6B, C). Of interest, CD4^+^ T cells from spleens of leukemia-bearing *Rag2*^*−/*^^−^ mice showed a higher frequency of T_R_1 cells (Supplementary Fig. 6D) in comparison to non-leukemic control mice, accompanied by a high co-expression of LAG3 on these cells (Supplementary Fig. 6E). The leukemic challenge of animals induced a higher proliferation rate of T_R_1 cells in comparison to non-diseased mice (Supplementary Fig. 6F). In contrast, the cytotoxic ability of T_R_1 cells was not changed upon development of TCL1 leukemia (Supplementary Fig. 6G, H).

Next, we clustered FOXP3^−^ CD4^+^ T cells derived of mice with and without TCL1 AT based on their expression of EOMES, PD-1, LAG3, and KI-67 measured by flow cytometry using T-distributed Stochastic Neighbor Embedding (t-SNE) algorithm to infer if CLL development is changing the phenotype of this cell type. As expected based on prior analyses, T_R_1 cells clustered similar, regardless if mice were challenged with leukemic cells or not (Supplementary Fig. 6I), suggesting that TCL1 leukemia does not alter the phenotype of this cell type but rather induces its expansion.

In sum, the accumulation of cytotoxic T_R_1 cells advises to investigate their role in controlling CLL progression.

### EOMES is indispensable for T_R_1 cell-mediated control of leukemia development in TCL1 AT mice

**Fig. 5:**
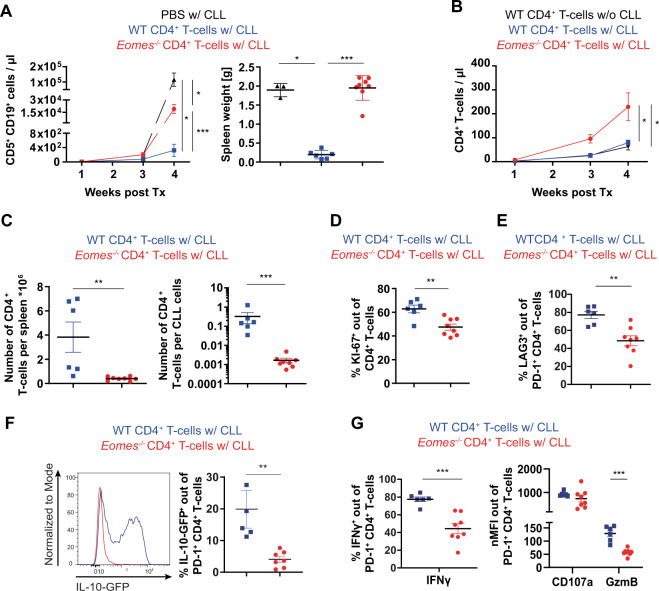
EOMES is crucial for T_R_1 cell-mediated CLL control. *Rag2*^*−/−*^ mice were transplanted i.v. with PBS or CD4^+^ T cells of wild type (WT) or *Eomes*^−^^*/*^^−^ origin on day -1 and the following day with leukemic splenocytes of Eµ-TCL1 mice. Blood and spleen samples were analyzed at indicated time points by flow cytometry. **A** Absolute numbers of CD5^+^ CD19^+^ CLL cells in peripheral blood are shown over time and spleen weights at endpoint, 4 weeks after transfer of leukemic cells. **B** Absolute numbers of CD4^+^ T cells in peripheral blood are depicted over time. **C** Numbers of CD4^+^ T cells per spleen, and numbers of CD4^+^ T cells per CD5^+^ CD19^+^ CLL cell are shown. **D** Percentages of KI-67^+^ cells out of splenic CD4^+^ T cells. **E** Percentages of LAG3^+^ cells out of splenic PD-1^+^ CD4^+^ T cells. **F** Expression of IL-10-GFP out of splenic PD-1^+^ CD4^+^ T cells shown as representative histogram and quantification of data. **G** Splenocytes were stimulated ex vivo with PMA/ionomycin and cytokine expression was analyzed by intracellular flow cytometry. Frequencies of IFNγ^+^ cells out of PD-1^+^ CD4^+^ T cells, and nMFI of CD107a and GzmB of PD-1^+^ CD4^+^ T cells. All graphs show mean ± SEM. Each dot represents data of an individual mouse. Statistical analysis was performed using Mann–Whitney test. *p < 0.05; **p < 0.01; ***p < 0.001. Tx = transplantation, nMFI =  normalized median fluorescence intensity.

To analyze the role of T_R_1 cells in controlling CLL progression, we utilized conditional *Eomes* knock-out (*Eomes*^−/−^) mice, as we have shown that EOMES is essential for the generation of functional T_R_1 cells, in concordance with previous reports [2]. WT or *Eomes*^−/−^ CD4^+^ T cells were transplanted into *Rag2*^−/−^ mice followed by adoptive transfer of TCL1 leukemia cells. Analysis of CLL progression in these mice showed that *Eomes*^−/−^ CD4^+^ T cells failed to control CLL development as evidenced by higher numbers of CD5^+^ CD19^+^ CLL cells in blood and higher spleen weights (Fig. 5A). We further monitored CD4^+^ T-cell expansion in these mice over time and observed higher T-cell numbers in the blood of recipient mice of *Eomes*^−/−^ CD4^+^ T cells compared to WT T cells (Fig. 5B). However, in the spleen of these animals, a lower absolute number of CD4^+^ T cells per spleen and per CLL cell was noted in the *Eomes*^−/−^ in comparison to the WT group (Fig. 5C). In line with this, *Eomes*^−/−^ T cells in the spleen showed a lower proliferation rate based on KI-67 staining compared to WT T cells (Fig. 5D), suggesting that EOMES is important for the expansion of T_R_1 cells. As our data showed an enrichment and higher activation state of T_R_1 cells in lymphoid organs of CLL patients, we subsequently analyzed splenic T cells of these mice for T_R_1-related molecules. In line with the RNA-sequencing results, EOMES-deficiency resulted in a reduced expression of LAG3 in comparison to WT T cells (Fig. 5E). Moreover, EOMES was indispensable for IL-10 production (Fig. 5F) and cytotoxic function, as we observed a lower expression of IFNγ, a slight reduction in CD107a, and a significantly lower production of GzmB in EOMES-deficient T cells (Fig. 5G).

In summary, EOMES is important for the control of CLL progression in mice as it mediates expansion and cytotoxicity of CD4^+^ T cells.

### IL-10R signaling maintains cytotoxic T_R_1 cells and allows them to control CLL

**Fig. 6:**
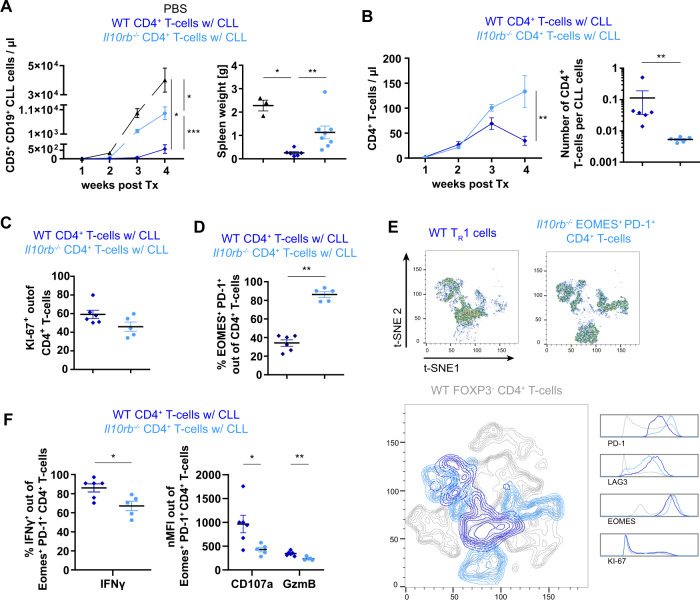
IL-10R signaling controls CLL development by maintaining functional T_R_1 cells. *Rag2*^*−/−*^ mice were transplanted i.v. with PBS or CD4^+^ T cells of wild type (WT) or *Il10rb*^*−/−*^ origin on day -1 and the following day with leukemic splenocytes of Eµ-TCL1 mice. Blood and spleen samples were analyzed by flow cytometry. **A** Absolute numbers of CD5^+^ CD19^+^ CLL cells in peripheral blood are shown over time and spleen weights at endpoint, 4 weeks after transfer of leukemic cells. **B** Absolute numbers of CD4^+^ T cells in peripheral blood are depicted over time, and numbers of CD4^+^ T cells per CD5^+^ CD19^+^ CLL cell are shown. **C** Percentage of KI-67^+^ cells out of splenic CD4^+^ T cells. **D** Frequency of EOMES^+^ PD-1^+^ cells out of splenic CD4^+^ T cells. **E** t-SNE plots of concatenated, splenic WT T_R_1 cells and *Il10rb*^−/−^ EOMES^+^ PD-1^+^ CD4^+^ T cells based on flow cytometry data for PD-1, EOMES, LAG3, and KI-67 expression (upper plots). Overlay of both cell types in dark and light blue with WT FOXP3^−^ CD4^+^ T cells in gray (lower plot). **F** Splenocytes were stimulated ex vivo with PMA/ionomycin and cytokine expression was analyzed by intracellular flow cytometry. Frequencies of IFNγ^+^ cells out of PD-1^+^ CD4^+^ T cells, and nMFI of CD107a and GzmB of PD-1^+^ CD4^+^ T cells. All graphs show mean ± SEM. Each dot represents data of an individual mouse. Statistical analysis was performed using Mann–Whitney. *p < 0.05; **p < 0.01; ***p < 0.001. nMFI = normalized median fluorescence intensity.

Ingenuity pathway analysis of *Eomes*-dependent genes indicated *Il10* and *Stat3* as the top potential upstream regulators (Supplementary Table 7). In line, IL-10R signaling has been shown to be essential for T_R_1 function [45]. Therefore, we decided to investigate the role of IL-10R-mediated signaling in T_R_1 cells and its impact on control of TCL1 leukemia. Either *Il10rb*^*+/+*^ (WT) or *Il10rb*^*−/−*^ CD4^+^ T cells were injected into *Rag2*^−/−^ mice followed by transplantation of TCL1 leukemia. Of interest, *Il10rb*-deficient CD4^+^ T cells showed a reduced control of CLL as measured by higher CD5^+^ CD19^+^ CLL counts in blood over time and by higher spleen weights 4 weeks after TCL1 AT in comparison to control mice receiving WT T cells (Fig. 6A). To evaluate whether a reduced expansion of *Il10rb*^*−/−*^ CD4^+^ T cells contributes to the diminished CLL control, T-cell counts were monitored in blood over time. Three and 4 weeks after transfer of leukemic cells, a higher number of *Il10Rb*^*−/−*^ versus WT CD4^+^ T cells was seen in blood (Fig. 6B). CD4^+^ T-cell counts in spleen showed a trend toward a higher number of *Il10rb*^*−/−*^ CD4^+^ T cells compared to WT T cells per spleen (Supplementary Fig. 7A), which is likely a reflection of the bigger spleen sizes in the *Il10rb*^*−/*^^−^ group, as the number of CD4^+^ T cells per CLL cell was reduced in these mice in comparison to the WT group (Fig. 6B). Nevertheless, proliferation of CD4^+^ T cells, as measured by KI-67, did not differ significantly between the two groups (Fig. 6C). Hence, IL-10R-mediated signaling in CD4^+^ T cells is required for their efficient control of CLL development which is not primarily due to an impact on T-cell expansion.

IL-10Rα signaling was shown to be dispensable for the differentiation of T_R_1 cells, but not for the function of this cell type [45]. Similarly, we investigated the relevance of *Il10Rb* for T_R_1 cells. As *Il10rb*^*−/−*^ CD4^+^ T cells showed a higher frequency of T_R_1 cells than WT T cells (Fig. 6D), we clustered FOXP3^−^ CD4^+^ T cells of WT and *Il10Rb*^*−/−*^ origin based on their expression of EOMES, PD-1, LAG3, and KI-67 measured by flow cytometry using t-SNE algorithm for a comparative analysis. Intriguingly, EOMES^+^ PD-1^+^ CD4^+^ T_R_1-like cells of *Il10Rb*^*−/−*^ origin clustered distinct of WT T_R_1 cells (Fig. 6E and Supplementary Fig. 7B), likely driven by a higher expression of PD-1. This suggests that IL-10R signaling is crucial for maintaining the T_R_1 cell phenotype. This finding was further supported by a reduced cytotoxic function of *Il10rb*^*−/−*^ EOMES^+^ PD-1^+^ CD4^+^ T cells in comparison to WT T_R_1 cells (Fig. 6F), arguing for a different phenotype of these cells arising upon IL-10R deficiency.

Taken together, our data suggests that EOMES is indispensable for the accumulation of cytotoxic T_R_1 cells, a cell type whose function depends on IL-10R signaling and that limits CLL progression.

## Discussion

An altered frequency of CD4^+^ T-cell subsets in B-NHL patients is widely described [9, 35, 42, 46], but their function and pathological relevance are not well understood [1]. Therefore, we aimed to elucidate the role of PD-1-expressing CD4^+^ T cells in these malignancies. By analyzing blood samples of CLL and DLBCL patients, we observed a higher proportion of PD-1-expressing CD4^+^ T cells in both patient groups compared to HC thus confirming published data [6–10]. Moreover, we noted that a subset of PD-1^+^ cells co-expressed EOMES, which has been observed for T_R_1 cells. Similar to PD-1-expressing CD8^+^ T cells [34, 36], T_R_1-like T cells were enriched in LN samples of CLL patients, the site of tight interactions between malignant B and T cells and of their proliferation [47, 48], and showed a higher activity than respective cells in paired blood samples. We further observed that these cells harbor cytotoxic capacities, which is in line with published data shown for CD4^+^ T cells in the blood of B-NHL patients [49–51]. Interestingly, these CD4^+^ T cells have been shown to kill autologous B cells, regardless if they were derived from CLL patients or HC [49].

T_R_1 cells do not constitutively express FOXP3, produce the immunosuppressive cytokine IL-10, express co-inhibitory receptors such as PD-1 or LAG3, and produce cytotoxic molecules [5]. EOMES has recently been shown to be of importance for the generation of T_R_1 cells [2, 3] and to regulate the expression of IL-10 in these cells [2, 3, 52]. Furthermore, the importance of EOMES for the production of cytotoxic molecules of CD4^+^ T cells is well described [22, 23, 53]. Using RNA-sequencing and phenotypic analyses of T cells from mouse models, we confirmed an overlap of EOMES^+^ PD-1^+^ CD4^+^ T cells analyzed in our study with human T_R_1 cells, and proved that EOMES is involved in their generation and indispensable for their function. Of importance, EOMES only controlled a small gene set, which was enriched in factors that were previously described for T_R_1 cells, such as IL-10, PD-1, and LAG3. Among those genes were also several integrins which might be involved in migration and homing of T cells, and therefore responsible for the higher number of EOMES-deficient compared to -proficient CD4^+^ T cells remaining in the blood after their transfer in *Rag2*^−/−^ mice with CLL.

Intriguingly, using mouse models of CLL, we showed that EOMES is essential for the CD4^+^ T cell-mediated control of CLL in *Rag2*^−/−^ mice. In these mice, CD4^+^ T cells expanded and acquired cytotoxic activity, which is in line with similar results in mouse models of melanoma [54].

Since IL-10-driven signaling via p38 MAPK was shown to be important to maintain IL-10 production in T_R_1 cells [45], we investigated the role of IL-10R signaling in T_R_1 cells and the control of leukemia progression. Interestingly, *Il10rb*^*−/−*^ CD4^+^ T cells showed a reduced CLL control alongside with a high expression of PD-1 and EOMES, being distinct from that of WT T_R_1 cells. This increase in PD-1 and EOMES expression was accompanied by a reduction in IFNγ production, suggesting that IL-10R signaling is important to maintain a functional cytotoxic T_R_1 subset. This is in line with published results for CD8^+^ T cells, showing that overexpression of EOMES resulted in an increased expression of exhaustion molecules such as *CD244*, *Havcr2*, and *Il10ra*, implicating a role for IL-10-mediated signaling in regulating T-cell exhaustion [55]. Studies in infection models and from murine and human cancer showed that the expression level of PD-1 in CD8^+^ T cells determines their state of exhaustion and potential for reinvigoration by PD-1 blockade [56]. Along this line, we observed that IL-10-mediated signaling maintains a subset of CD8^+^ T cells with intermediate PD-1 expression and high potential to control CLL in mice [57]. Together, this suggests that IL-10-mediated signals are important to maintain effector function not only in CD8^+^ T cells but also in T_R_1 cells.

Genome-wide association studies showed that single nucleotide polymorphisms (SNPs) in proximity of the *EOMES* gene are associated with a higher risk of CLL (rs9880772) [58, 59], DLBCL (rs6773363) [60], and Hodgkin’s Lymphoma (rs3806624) [61]. The higher likelihood of B-NHL in individuals carrying these SNPs was thought to be caused by a deregulated immune function, which could at least partially be explained by the reduced control of CLL development, as seen in our study. Alongside, preclinical data in mouse models [62], and data of a phase 1 basket trial (NCT02009449) using pegylated IL-10 for treatment of solid cancer, demonstrated that IL-10 helps to maintain CD8^+^ T cell-mediated tumor control and improves patients’ responses to PD-1 blockade [63].

In summary, this report highlights the presence of cytotoxic T_R_1 cells in LNs of CLL and DLBCL patients and in the TCL1 mouse model of CLL. Our data in this animal model clearly show that EOMES-expressing CD4^+^ T cells are crucial for disease control. We further demonstrate the importance IL-10-mediated signaling in maintaining T_R_1 cells, which mediate effector activity and thus control of leukemia.

## Supplementary information

Suppl. MM and Suppl. Tables and Figure legends

Supplementary Figure 1

Supplementary Figure 2

Supplementary Figure 3

Supplementary Figure 4

Supplementary Figure 5

Supplementary Figure 6

Supplementary Figure 7

Suppl. Table 6_sheet 1_comparison 1

Suppl. Table 6_sheet 2_comparison 2

Suppl. Table 6_sheet 3_intersection

Suppl. Table 7
