# Supplementary material for: EOMES and IL-10 regulate antitumor activity of T regulatory type 1 CD4+ T cells in chronic lymphocytic leukemia
Source: Leukemia. 2021 Feb 1;35(8):2311–24. doi: 10.1038/s41375-021-01136-1 (PMC8324479; doi:10.1038/s41375-021-01136-1)
Supplement: Supplementary file 1 — Suppl. MM and Suppl. Tables and Figure legends [file 41375_2021_1136_MOESM1_ESM.pdf]

Supplementary Material and Methods

Processing of patient samples

Peripheral blood (PB) was drawn using ethylene diamine tetraacetic acid (EDTA)-coated tubes (Sarstedt, Nümbrecht, Germany). PB mononuclear cells (PBMCs) were isolated by Ficoll (Biochrom, Berlin, Germany) density gradient centrifugation. If necessary, PBMCs were viably frozen and when needed, frozen PBMCs were thawed and rested for three hours until further processing. Lymph node (LN) samples were processed as previously described (1).

Collection of murine tissue samples and preparation of cell suspensions

Mice were euthanized by increasing concentrations of carbon dioxide (CO_2). PB was drawn from the submandibular vein or via cardiac puncture and collected in EDTA-coated tubes (Sarstedt). Single-cell suspensions from spleens were prepared as previously described (2, 3). Splenocytes were enriched in $CD19^+$ cells using EasySep™ Mouse Pan-B Cell Isolation Kit (Stemcell Technologies, Vancouver, Canada) yielding a purity above 95% of $CD5^+CD19^+$ cells. $CD4^+$ T-cells were isolated from splenocytes using EasySep™ Mouse $CD4^+$ T Cell Isolation Kit resulting in a purity of about 95% $CD4^+$ T-cells.

Mice

Eomesodermin (*Eomes*) conditional allele mice that harbour loxP sites flanking the exons 2-5 encoding the T-box DNA-binding domain, were previously described (4). In order to achieve a T-cell-specific deletion of *Eomes*, these mice were crossed to a transgenic *Cre* line in which the expression of *Cre* recombinase is regulated by the mouse proximal *Lck* promoter (5). Mice carrying a *GFP* reporter allele of *Eomes* were previously described (6). This mouse line was crossed with the *Eomes*$^{fl/fl}$ *x Lck-cre* strain to generate *Eomes*$^{\Delta T/GFP}$ knock-out mice. Moreover, *Lck-cre x Eomes*$^{fl/fl}$ *x Foxp3-IRES-mRFP (FIR)* (7) x *Il10-GFP (tiger)* (8) (*Eomes*$^{-/-}$ *knock-out* mice) or *Eomes*$^{fl/fl}$ *x FIR x tige*r (WT) mice were used for TCL1 leukemia transfer. All mouse strains described above were maintained under conventional or specific pathogen-free conditions at Max-Planck Institute of Immunobiology and Epigenetics (Freiburg, Germany). Eμ-TCL1 (C. Croce, OH, USA) mice were held at specific

pathogen-free conditions on a pure C57BL/6N or J background at the central animal facility of the German Cancer Research Center (DKFZ). *Rag2*$^{-/-}$ mice were held at specific pathogen-free conditions at the central animal facility of DKFZ.

Flow cytometric analysis and cell sorting

Whole blood stainings were performed by addition of the surface antibody cocktail (list of antibodies supplied in Supplementary Table 5) to a defined volume of blood. Red blood cell lysis was performed by addition of 1x Red blood cell lysis buffer (BD Bioscience, Heidelberg, Germany or ThermoFisher Scientific, Dreieich, Germany) and subsequent incubation at room temperature. After centrifugation, cell suspension was resuspended in 1x Red blood cell lysis buffer and 123count eBeads™ Counting Beads (ThermoFisher Scientific) were added to determine cell concentrations by the formula: Absolute Count (cells/µl) = (Cell count x eBead™ Volume) / (eBead™ Count x Cell Volume) x (eBead™ concentration).

Single cell suspensions were stained in phosphate-buffered saline (PBS) with addition Fixable Viability Dye eFluor® 506 (ThermoFisher Scientific) at a concentration of 1:1000 for 30 min at 4 °C. Cells were fixed using eBioscience™ Foxp3 / Transcription Factor Staining Buffer Set (ThermoFisher Scientific) for 30 min at room temperature. Subsequently, cells were permeabilized with eBioscience™ Permeabilization Buffer (ThermoFisher Scientific) and stained intracellularly for 30 min at room temperature. Samples were stored at 4 °C in the dark until acquisition.

Cytokine stainings were performed as previously described (3, 9). Briefly, cells were stimulated using either 1x PMA/ionomycin-based eBioscience™ Cell Stimulation and Inhibitor Cocktail (ThermoFisher Scientific) or a stimulation cocktail containing 0.1 µg/ml of PMA (Enzo Life Sciences, Lausen, Switzerland), 1 µg/ml of ionomycin (SERVA Electrophoresis, Heidelberg, Germany) and 1x protein transport inhibitor Monensin (BioLegend, London, United Kingdom) for 4-6 hours at 37 °C and 5 % CO_2. Subsequently, cells were washed, surface stained, fixed and permeabilized as detailed above.

Flow cytometry data was acquired using a BD FACS Canto II, BD LSR II or BD LSR Fortessa (BD Biosciences) FACS analyzer and analyzed using FlowJo X 10.0.7 software (FlowJo, Ashland, OR, USA). $CD4^+$ T-cells were defined as $CD3^+$ $CD4^+$ or $CD3^+$ $CD8^-$.

Fluorescence-activated cell sorting of naive $CD25^-$ $CD45RB^{hi}$ $CD4^+$ T-cells or of activated GFP^+ and GFP^- $CD4^+$ splenic T-cells (purity typically >98 %) after 3 weeks of adoptive transfer was performed using BD FACSAria™ III or BD FACSAria™ Fusion (BD Biosciences) instruments as previously described (3).

Data analysis and graphical display were performed using Prism 7 GraphPad software (GraphPad Software, La Jolla, USA).

RNA sequencing and analysis

For RNA sequencing, total RNA was extracted with TRI reagent (Sigma-Aldrich, Munich, Germany) according to the manufacturer's instructions. Depletion of ribosomal RNA and library preparation was performed with TruSeq® Stranded Total RNA Gold kit (former name TruSeq® Stranded Total RNA LT - (with Ribo-Zero TM GOLD), Illumina, San Diego, USA). The obtained RNA was quality-controlled in a Fragment Analyzer (Agilent Technologies, Santa Clara, USA) and sequenced with an Illumina HiSeq2500 in a 2 x 75 bp paired end, multiplexing run, aiming for ~25 million reads per sample. Raw data from the Illumina HiSeq 2500 sequencing machine was demultiplexed and converted into FASTQ files using Illumina bcl2fastq2 (version 1.8.4, http://support.illumina.com/downloads/bcl2fastq_conversion_software_184.html). Data was screened for contamination with fastq_screen (version 0.5.1, (http://www.bioinformatics.babraham.ac.uk/projects/fastq_screen/). Subsequent analysis steps including quality control, detection and trimming of adaptors, alignment of the trimmed reads to mouse genome (build mm10) and counting of aligned reads were performed with mRNA-seq workflow from snakePipes (version 2.1.1) (10). Versions of all the tools utilized by the snakePipes workflow are listed in Supplementary Table 4. Samples with low number of uniquely mapping reads and high percent of duplicate reads (> 30 %) were not considered for differential gene expression

analysis or any of the visualizations. Differential gene expression analysis was performed using the DESeq2 (11). Comparison 2 was performed with the standard settings of DESeq2, while for comparison 1 we performed pairwise comparisons, since GFP^+ and GFP^- subsets came from the same recipient mouse. We considered genes with adjusted p-values less than 0.05 as significantly differentially expressed. For both comparisons shrunk log2 fold changes are presented in Supplementary Table 5. DESeq2 normalized counts were used to build a heatmap in Figure 2B; heatmap was created with R package pheatmap (version 1.0.12) (https://CRAN.R-project.org/package=pheatmap). Multidimensional scaling plot was created with plotMDS function from edgeR Bioconductor package (12). DESeq2-generated log2 fold change data and p values were used for canonical pathway analysis, performed with Ingenuity Pathway Analysis (IPA) software (version 52912811, Qiagen) (13). Canonical pathways analysis identified the pathways from the Ingenuity Pathway Analysis library that were most significant for the comparison 1 or comparison 2 datasets. Further, IPA comparison analysis was performed to identify canonical pathways, differentially regulated in both datasets. Absolute value of z-scores from Pathway Activity Analysis was used to identify top common canonical pathways, which have been plotted in a heatmap in Figure 2E. Venn diagram in Figure 2C was created from DESeq2 differential expression data using python library matplotlib-venn (https://pypi.python.org/pypi/matplotlib-venn). RNA-seq track visualizations were exported from Integrative Genomics Viewer (IGV, version 2.4.17) (14). Comparison of RNA-Seq data with the published human T_R1 signature (15) was performed with gene set enrichment analysis (GSEA) as described (16, 17). Our data are available on Gene Expression Omnibus (GEO) under the accession numbers GSE145145.

References

1. Roider T, Seufert J, Uvarovskii A, Frauhammer F, Bordas M, Abedpour N, et al. Dissecting intratumour heterogeneity of nodal B-cell lymphomas at the transcriptional, genetic and drug-response levels. Nat Cell Biol. 2020.
2. Hanna BS, McClanahan F, Yazdanparast H, Zaborsky N, Kalter V, Rossner PM, et al. Depletion of CLL-associated patrolling monocytes and macrophages controls disease development and repairs immune dysfunction in vivo. Leukemia. 2016;30(3):570-9.
3. Lupar E, Brack M, Garnier L, Laffont S, Rauch KS, Schachtrup K, et al. Eomesodermin Expression in CD4+ T Cells Restricts Peripheral Foxp3 Induction. Journal of immunology (Baltimore, Md : 1950). 2015;195(10):4742-52.
4. Arnold SJ, Hofmann UK, Bikoff EK, Robertson EJ. Pivotal roles for eomesodermin during axis formation, epithelium-to-mesenchyme transition and endoderm specification in the mouse. Development. 2008;135(3):501-11.
5. Orban PC, Chui D, Marth JD. Tissue- and site-specific DNA recombination in transgenic mice. Proceedings of the National Academy of Sciences of the United States of America. 1992;89(15):6861-5.
6. Arnold SJ, Sugnaseelan J, Groszer M, Srinivas S, Robertson EJ. Generation and analysis of a mouse line harboring GFP in the Eomes/Tbr2 locus. Genesis (New York, NY : 2000). 2009;47(11):775-81.
7. Wan YY, Flavell RA. Identifying Foxp3-expressing suppressor T cells with a bicistronic reporter. Proceedings of the National Academy of Sciences of the United States of America. 2005;102(14):5126-31.
8. Kamanaka M, Kim ST, Wan YY, Sutterwala FS, Lara-Tejero M, Galan JE, et al. Expression of interleukin-10 in intestinal lymphocytes detected by an interleukin-10 reporter knockin tiger mouse. Immunity. 2006;25(6):941-52.
9. Hanna BS, Roessner PM, Yazdanparast H, Colomer D, Campo E, Kugler S, et al. Control of chronic lymphocytic leukemia development by clonally-expanded CD8(+) T-cells that undergo functional exhaustion in secondary lymphoid tissues. Leukemia. 2019;33(3):625-37.
10. Bhardwaj V, Heyne S, Sikora K, Rabbani L, Rauer M, Kilpert F, et al. snakePipes: facilitating flexible, scalable and integrative epigenomic analysis. Bioinformatics (Oxford, England). 2019;35(22):4757-9.
11. Anders S, Pyl PT, Huber W. HTSeq--a Python framework to work with high-throughput sequencing data. Bioinformatics (Oxford, England). 2015;31(2):166-9.
12. Robinson MD, Oshlack A. A scaling normalization method for differential expression analysis of RNA-seq data. Genome Biol. 2010;11(3):R25.
13. Krämer A, Green J, Pollard J, Jr., Tugendreich S. Causal analysis approaches in Ingenuity Pathway Analysis. Bioinformatics (Oxford, England). 2014;30(4):523-30.
14. Robinson JT, Thorvaldsdóttir H, Winckler W, Guttman M, Lander ES, Getz G, et al. Integrative genomics viewer. Nature biotechnology. 2011;29(1):24-6.
15. Gruarin P, Maglie S, De Simone M, Haringer B, Vasco C, Ranzani V, et al. Eomesodermin controls a unique differentiation program in human IL-10 and IFN-gamma coproducing regulatory T cells. European journal of immunology. 2019;49(1):96-111.
16. Subramanian A, Tamayo P, Mootha VK, Mukherjee S, Ebert BL, Gillette MA, et al. Gene set enrichment analysis: a knowledge-based approach for interpreting genome-wide expression profiles. Proceedings of the National Academy of Sciences of the United States of America. 2005;102(43):15545-50.
17. Mootha VK, Lindgren CM, Eriksson KF, Subramanian A, Sihag S, Lehar J, et al. PGC-1alpha-responsive genes involved in oxidative phosphorylation are coordinately downregulated in human diabetes. Nature genetics. 2003;34(3):267-73.

Supplementary Table 1: **Clinical information for whole blood samples of CLL patients and healthy controls**

	HC	CLL
**Number of samples**	19	17
**Sex**	26.3 % female (5/19)	41.2 % female (7/17)
**Age (years)**	mean: 61.2<br>median: 61	mean: 60.4<br>median: 60
**CMV IgG positivity**	55.6 % CMV IgG^+ (10/18)	40.0 % CMV IgG^+ (6/15)
**Binet stage**		70.6 % A (12/17)<br>17.6 % B (3/17)<br>11.8 % C (2/17)
**Mutational state of *IGHV***		58.8 % mutated (10/17)
**Chromosomal aberration**		64.7 % del13q14.3 (11/17)<br>23.5 % normal karyotype (4/17)<br>5.9 % trisomy 12 (1/17)<br>5.9 % IGH translocation
**TP53 mutation**		20.0 % (2/10)
**Prior treatment**		0.0 % (0/17)

Supplementary Table 2: **Clinical information for whole blood samples of DLBCL patients and healthy controls**

	HC	DLBCL
**Number of samples**	19	18
**Age (years)**	mean: 61.2<br>median: 61	mean: 66.29 (17/18)<br>median: 65 (17/18)
**Relapsed**		5.6 % (1/18)

Supplementary Table 3: Clinical information for comparison of lymph node samples of CLL and DLBCL patients

	CLL	DLBCL	rLN
**Number of samples**	7	7	9
**Sub-classification**	Not applicable	Germinal center B-cell: 12.5 % (1/7)<br>Non-germinal center B-cell: 85.7 % (6/7)	Not applicable
**Clinical Situation**	Initial diagnosis: 100 % (7/7)	Initial diagnosis: 28.6 % (2/7)<br>Relapse: 71.4 % (5/7)	Not applicable
**Age**	Average: 72 years	Average: 67	Average: 39
**Gender**	Male (7/7)	Male (2/7)<br>Female (5/7)	Male (7/9)<br>Female (2/9)

Supplementary Table 4: List of flow cytometry antibodies

Live/dead dye:	Supplier
BD Horizon™ Fixable Viability Stain 780	BD Biosciences, Heidelberg, Germany
eBioscience™ Fixable Viability Dye eFluor® 450	ThermoFisher Scientific, Dreieich, Germany
eBioscience™ Fixable Viability Dye eFluor® 506	ThermoFisher Scientific, Dreieich, Germany
eBioscience™ Fixable Viability Dye eFluor® 780	ThermoFisher Scientific, Dreieich, Germany

antibody	Clone	Supplier
anti-human BATF PE	S39-1060	BD Biosciences, Heidelberg, Germany
anti-human CD107a (LAMP-1) APC	H4A3	BioLegend, London, United Kingdom
anti-human CD279 (PD-1) APC	EH12.2H7	BioLegend, London, United Kingdom
anti-human CD279 (PD-1) PE	EH12.2H7	BioLegend, London, United Kingdom
anti-human CD279 (PD-1) PE/Cy7	EH12.2H7	BioLegend, London, United Kingdom
anti-human CD279 (PD-1) PE/Dazzle™ 594	EH12.2H7	BioLegend, London, United Kingdom
anti-human CD3 Brilliant Violet 510™	OKT3	BioLegend, London, United Kingdom
anti-human CD3 PerCP/Cy5.5	OKT3	BioLegend, London, United Kingdom
anti-human CD4 APC/Cy7	OKT4	BioLegend, London, United Kingdom
anti-human CD4 BD Horizon™ BUV395	SK3	BD Biosciences, Heidelberg, Germany
anti-human CD4 Brilliant Violet 510™	OKT3	BioLegend, London, United Kingdom
anti-human CD4 Brilliant Violet 650™	OKT4	BioLegend, London, United Kingdom
anti-human CD69 PE/Dazzle™ 594	FN50	BioLegend, London, United Kingdom
anti-human CD8a Brilliant Violet 605™	RPA-T8	BioLegend, London, United Kingdom
anti-human CD8a FITC	RPA-T8	BioLegend, London, United Kingdom
anti-human EOMES eFluor® 660 eBioscience™	WD1928	ThermoFisher Scientific, Dreieich, Germany
anti-human HLA-DR Brilliant Violet 711™	L243	BioLegend, London, United Kingdom
anti-human IFN-γ PE/Cy7	4S.B3	BioLegend, London, United Kingdom
anti-human IRF4 PerCP-eFluor® 710 eBioscience™	3E4	ThermoFisher Scientific, Dreieich, Germany
anti-human/mouse Granzyme B FITC	GB11	BioLegend, London, United Kingdom
anti-mouse CD107a (LAMP-1) PE eBioscience™	eBio1D4B	ThermoFisher Scientific, Dreieich, Germany
anti-mouse CD127 APC	A7R 34	BioLegend, London, United Kingdom
anti-mouse CD127 BV605	A7R 35	BioLegend, London, United Kingdom
anti-mouse CD127 PE	A7R 34	BioLegend, London, United Kingdom
anti-mouse CD127 PE/Cy7	A7R 34	BioLegend, London, United Kingdom
anti-mouse CD127 PE/Dazzle™ 594	A7R 34	BioLegend, London, United Kingdom
anti-mouse CD19 FITC eBioscience™	eBio1D3	ThermoFisher Scientific, Dreieich, Germany
anti-mouse CD19 PE eBioscience™	eBio1D3	ThermoFisher Scientific, Dreieich, Germany
anti-mouse CD19 PE/Dazzle™ 594	6D5	BioLegend, London, United Kingdom
anti-mouse CD223 (LAG-3) Biotin	C9B7W	BioLegend, London, United Kingdom
anti-mouse CD223 (LAG-3) PE	C9B7W	BioLegend, London, United Kingdom
anti-mouse CD3e V450	500A2	BD Biosciences, Heidelberg, Germany
anti-mouse CD4 APC	RM4-4	BioLegend, London, United Kingdom
anti-mouse CD4 APC	RM4-5	ThermoFisher Scientific, Dreieich, Germany
anti-mouse CD4 APC/Cy7	RM4-5	BioLegend, London, United Kingdom
anti-mouse CD4 APC/Cy7	GK1.5	BioLegend, London, United Kingdom or

		ThermoFisher Scientific, Dreieich, Germany
anti-mouse CD4 Biotin	GK1.5	BioLegend, London, United Kingdom or ThermoFisher Scientific, Dreieich, Germany
anti-mouse CD4 Brilliant Violet 421™	GK1.5	BioLegend, London, United Kingdom or ThermoFisher Scientific, Dreieich, Germany
anti-mouse CD4 BV421	RM4-5	ThermoFisher Scientific, Dreieich, Germany
anti-mouse CD4 FITC	GK1.5	BioLegend, London, United Kingdom or ThermoFisher Scientific, Dreieich, Germany
anti-mouse CD4 PE	GK1.5	BioLegend, London, United Kingdom or ThermoFisher Scientific, Dreieich, Germany
anti-mouse CD4 PE/Cy7	GK1.5	BioLegend, London, United Kingdom or ThermoFisher Scientific, Dreieich, Germany
anti-mouse CD4 PerCP/Cy5.5	RM4-4	BioLegend, London, United Kingdom
anti-mouse CD44 Alexa Fluor® 700 eBioscience™	IM7	ThermoFisher Scientific, Dreieich, Germany
anti-mouse CD44 APC	IM7	ThermoFisher Scientific, Dreieich, Germany or BioLegend, London, United Kingdom
anti-mouse CD44 APC/Cy7	IM7	ThermoFisher Scientific, Dreieich, Germany or BioLegend, London, United Kingdom
anti-mouse CD44 FITC eBioscience™	IM7	ThermoFisher Scientific, Dreieich, Germany
anti-mouse CD44 PE/Cy5	IM7	ThermoFisher Scientific, Dreieich, Germany or BioLegend, London, United Kingdom
anti-mouse CD44 PerCP/Cy5.5 eBioscience™	IM7	ThermoFisher Scientific, Dreieich, Germany
anti-mouse CD45 Alexa Fluor® 700	30F-11	BioLegend, London, United Kingdom
anti-mouse CD45 Brilliant Violet 711™	30F-11	BioLegend, London, United Kingdom
anti-mouse CD45 PerCP/Cy5.5	30F-11	BioLegend, London, United Kingdom
anti-mouse CD45.1 Biotin	A20	BioLegend, London, United Kingdom or ThermoFisher Scientific, Dreieich, Germany
anti-mouse CD45.1 FITC	A20	BioLegend, London, United Kingdom or ThermoFisher Scientific, Dreieich, Germany
anti-mouse CD45.1 PE	A20	BioLegend, London, United Kingdom or ThermoFisher Scientific, Dreieich, Germany
anti-mouse CD45.2 Biotin	104	BioLegend, London, United Kingdom
anti-mouse CD45.2 FITC	104	BioLegend, London, United Kingdom
anti-mouse CD45.2 Pacific Blue™	104	BioLegend, London, United Kingdom
anti-mouse CD45.2 PE	104	BioLegend, London, United Kingdom
anti-mouse CD45RB Biotin	C363-16A	BioLegend, London, United Kingdom or ThermoFisher Scientific, Dreieich, Germany
anti-mouse CD45RB PerCP/Cy5.5	C363-16A	BioLegend, London, United Kingdom or ThermoFisher Scientific, Dreieich, Germany
anti-mouse CD5 APC eBioscience™	53-7.3	ThermoFisher Scientific, Dreieich, Germany
anti-mouse CD5 BD Horizon™ BV605	53-7.3	BD Biosciences, Heidelberg, Germany
anti-mouse CD5 FITC eBioscience™	53-7.3	ThermoFisher Scientific, Dreieich, Germany
anti-mouse CD69 Pe-Dazzle	H1.2F3	BioLegend, London, United Kingdom
anti-mouse CD8a APC/Cy7	53-6.7	BioLegend, London, United Kingdom
anti-mouse CD8a Brilliant Violet 605™	53-6.7	BioLegend, London, United Kingdom
anti-mouse Eomes eFluor®450	Dan11mag	ThermoFisher Scientific, Dreieich, Germany
anti-mouse Eomes eFluor®660	WD1928	ThermoFisher Scientific, Dreieich, Germany
anti-mouse Eomes eFluor®660	Dan11mag	ThermoFisher Scientific, Dreieich, Germany

anti-mouse Eomes PE	Dan11mag	ThermoFisher Scientific, Dreieich, Germany
anti-mouse Eomes PerCP-eFluor® 710 eBioscience™	Dan11mag	ThermoFisher Scientific, Dreieich, Germany
anti-mouse Foxp3 Alexa Fluor® 700	FJK-16s	ThermoFisher Scientific, Dreieich, Germany
anti-mouse Granzyme B FITC eBioscience™	NGZB	ThermoFisher Scientific, Dreieich, Germany
anti-mouse IFN-g Brilliant Violet 421™	XMG1.2	BioLegend, London, United Kingdom
anti-mouse IFN-g FITC	XMG1.2	BioLegend, London, United Kingdom
anti-mouse IFN-g PE	XMG1.2	BioLegend, London, United Kingdom
anti-mouse IFN-g PerCP/Cy5.5 eBioscience™	XMG1.2	ThermoFisher Scientific, Dreieich, Germany
anti-mouse IL-10 PE eBioscience™	JES5-16E3	ThermoFisher Scientific, Dreieich, Germany
Anti-mouse IL-10 PE-Cy7	JES5-16E3	BioLegend, London, United Kingdom
anti-mouse KI-67 FITC	SolA15	ThermoFisher Scientific, Dreieich, Germany
anti-mouse PD-1 APC	RMP1-30	BioLegend, London, United Kingdom
anti-mouse PD-1 PE	29F.1A12	BioLegend, London, United Kingdom
anti-mouse PD-1 PE/Cy7	RPM1-30	BioLegend, London, United Kingdom
anti-mouse PD-1 PE/Dazzle™ 594	RMP1-30	BioLegend, London, United Kingdom
Streptavidin Alexa Fluor® 488		BioLegend, London, United Kingdom or ThermoFisher Scientific, Dreieich, Germany
Streptavidin APC-Alexa Fluor™ 780		BioLegend, London, United Kingdom or ThermoFisher Scientific, Dreieich, Germany
Streptavidin eFluor™ 450		BioLegend, London, United Kingdom or ThermoFisher Scientific, Dreieich, Germany
Streptavidin PE/Cy7		BioLegend, London, United Kingdom or ThermoFisher Scientific, Dreieich, Germany
Streptavidin PerCP/Cy5.5		BioLegend, London, United Kingdom or ThermoFisher Scientific, Dreieich, Germany

Supplementary Table 5: mRNA-seq tools

tool	version	tool	version
deeptools	3.3.2	salmon	1.1.0
seqtk	1.3	r-base	3.6.2
pigz	2.3.4	r-wasabi	1.0.1
snpsplit	0.3.4	r-sleuth	0.30.0
samtools	1.10	r-dplyr	0.8.4
fastqc	0.11.9	r-ggplot2	3.2.1
cutadapt	2.8	r-pheatmap	
trim-galore	0.6.5	r-rmarkdown	
multiqc	1.8	r-knitr	>= 1.28
fastp	0.20.0	pandoc	
umi_tools	1.0.1	r-dt	
bedtools	2.29.2	r-rcolorbrewer	
samtools	1.10	bioconductor-deseq2	1.26.0
subread	2.0.0	bioconductor-tximport	1.14.0
hisat2	2.1.0	r-knitcitations	
star	2.7.3a	bioconductor-apeglm	

Supplementary Figures

Suppl. Figure 1: Quantification of T_R1-like cells in CLL and DLBCL blood samples

A) Representative gating strategy for flow cytometry analysis to define viable, single CD4$^+$ T-cells. **B)-E)** Peripheral blood samples of age-matched healthy controls (HC), or patients with CLL or DLBCL were stained for flow cytometry. **B)** Representative contour plots and frequency of PD-1$^+$ cells out of CD4$^+$ T-cells in HC and CLL patients. **C)** Absolute numbers of PD-1$^+$ CD4$^+$ T-cells in HC and CLL patients. **D)** Representative contour plots and frequency of PD-1$^+$ cells out of CD4$^+$ T-cells in HC and DLBCL patients. **E)** Absolute numbers of PD-1$^+$ CD4$^+$ T-cells in HC and DLBCL patients. **F)** Frequency of EOMES$^+$ PD-1$^+$ T_R1-like cells out of CD4$^+$ T-cells in HC and CLL patients. **G)** PBMC of treatment naïve CLL patients (n = 3) were collected over approximately five years and viably frozen. For analysis, sequential samples of one patient were thawed and analyzed the same day. Leukocyte counts in blood of CLL patients and frequency of T_R1-like EOMES$^+$ PD-1$^+$ cells out of CD4$^+$ T-cells are shown.

Graphs show mean ± SEM. Each dot represents data of an individual patient. Statistical analysis was performed using Mann-Whitney test. $^{**}p<0.01$, $^{***}p<0.001$.

Suppl. Figure 2: EOMES$^+$ PD-1$^+$ T_R1-like cells harbor cytotoxic functions

A)-C) PBMCs of CLL patients were stimulated with PMA/ionomycin *ex vivo*. **A)** GzmB expression, **B)** nMFI of CD107a, and **C)** IFNγ-secretion of PD-1$^+$ or PD-1$^-$ CD4$^+$ T-cells are shown as representative histograms and quantification of data. **D)-F)** PBMC of healthy controls (HC) or CLL patients were stimulated with PMA/ionomycin *ex vivo*. **D)** Frequency of GzmB$^+$ cells, **E)** nMFI of CD107a, and **F)** frequency of IFNγ$^+$ cells out of PD-1$^+$ CD4$^+$ T-cells are depicted. **G)-K)** Samples obtained from PB and lymph nodes (LN) of CLL patients were analyzed by flow cytometry. Data derived from paired samples is connected by a line. **G)** Number of CD4$^+$ T-cells per CLL cell in PB and paired LN samples of CLL patients. **H)** Representative histograms and frequency of CD69- and HLA-DR-positive T_R1-like cells. **I)** Representative histograms and nMFI of IRF4, and **J)** BATF of PD-1$^+$ CD4$^+$ T-cells from PB or LN

specimen. **K)** Frequency of EOMES$^+$ PD-1$^+$ T_R1-like cells out of CD4$^+$ T-cells in reactive, non-pathogenic lymph nodes (rLN) and in LNs of patients with CLL or DLBCL.

Graphs show mean ± SEM. In K) median is depicted. Each dot represents data of an individual patient. Statistical analysis was performed using Mann-Whitney test. Comparison of matched samples was performed using Wilcoxon matched-pairs signed rank test. *p<0.05, ***p<0.001. nMFI = normalized median fluorescence intensity.

Suppl. Figure 3: RNA-sequencing analysis of Eomes-deficient CD4$^+$ T-cells

Naïve CD25$^-$ CD45RB^{high} CD4$^+$ T-cells of *Eomes-GFP* reporter mice (*Eomes*$^{+/GFP}$), as well as of mice with a T-cell-specific deletion of *Eomes* in combination with a GFP reporter (*Eomes*$^{\Delta T/GFP}$) were transferred into *Rag2*$^{-/-}$ mice. Three weeks after adoptive transfer, RNA sequencing of sorted splenic GFP$^+$ and GFP$^-$ CD4$^+$ T-cell subsets was performed. **A**) Representative RNA-seq tracks of *Eomes* of all groups. **B)** Principle component analysis of the log2 fold changes of the different groups. **C)** Venn diagram showing overlap of comparison 1 (transcriptional differences between GFP$^+$ T_R1 cells and GFP$^-$ CD4$^+$ T-cells from *Eomes*$^{+/GFP}$ donor mice) and comparison 2 (differential gene expression between EOMES-proficient *Eomes*$^{+/GFP}$ and EOMES-deficient *Eomes*$^{\Delta T/GFP}$ GFP$^+$ CD4$^+$ T-cells) including numbers of genes in each subset. **D)** Top pathways identified as differentially expressed in both comparison 1 (left) and 2 (right) by Ingenuity Pathway Analysis software. Numbers in the heatmap are the absolute values of z-score for each of the pathways in a respective comparison. **E)** Representative dot plots of flow cytometric analysis of PD-1 and CD44 of CD4$^+$ T-cells after adoptive T-cell transfer in *Rag2*$^{-/-}$ mice (left) or in aged wildtype mice (right) gated either on total CD4$^+$ T-cells, EOMES$^-$ or EOMES$^+$ CD4$^+$ T-cells. **F)** Flow cytometric analysis showing representative dot plots and quantification of the frequency of the splenic CD44^{lo} PD-1$^+$ T-cell population after transfer of EOMES-proficient or -deficient CD4$^+$ T-cells into different or the same *Rag2*$^{-/-}$ hosts.

Graphs show mean ± SEM. Each dot represents data of an individual mouse. Statistical analysis was performed using Mann-Whitney test. Comparison of matched samples was performed using Wilcoxon matched-pairs signed rank test *p<0.05.

Suppl. Figure 4: Cytokine-related pathways are regulated by EOMES in $CD4^+$ T-cells

Cells for analyses were prepared as described in Figure 2. **A)** Gene set enrichment analysis of EOMES-dependent genes (comparison 2 in Figure 2A) against KEGG_Mouse_2019 database was performed, and the top 7 categories are depicted. Length of a bar indicates -log10 of adjusted p-value for gene set enrichment. **B)** Representative RNA-seq tracks demonstrating *Il10* gene expression in GFP^+ and GFP^- subsets of EOMES-proficient and -deficient $CD4^+$ T-cells.

Suppl. Figure 5: PD-1^+ $CD4^+$ T-cells are enriched in the Eμ-TCL1 mouse model of CLL and harbor cytotoxic function

Spleens of hemizygous Eμ-TCL1 leukemic mice and control littermates at a median age of 65 weeks, or animals after transplantation of Eμ-TCL1 leukemic cells into syngeneic wildtype (WT) mice (TCL1 AT) were analyzed by flow cytometry. **A)** Representative gating strategy for flow cytometry analysis to define viable, single $CD4^+$ T-cells in murine splenocytes. **B)** Representative histogram and frequency of LAG3 expression by T_R1 cells in CLL mouse models and their controls. **C)** Representative contour plots and frequency of $CD62L^{hi}$ $CD44^{low}$ naïve and $CD62L^{low}$ $CD44^{hi}$ antigen-experienced memory/effector (M/E) cells out of $CD4^+$ T-cells in WT mice or 4 weeks after TCL1 AT. **D)** Representative histogram and percentage of $CD69^+$ cells out of $CD4^+$ T-cells in WT mice and after TCL1 AT. **E)** Splenocytes of untransplanted mice (week 0) or leukemic TCL1 AT mice at indicated time points were stimulated *ex vivo* with PMA/ionomycin and expression of IL-10 was analyzed by intracellular flow cytometry. Representative histogram and longitudinal analysis of frequency of IL-10-expressing cells out of $CD4^+$ T-cells. **F)-G)** Splenocytes of leukemic TCL1 AT mice were stimulated with PMA/ionomycin *ex vivo*. Representative histogram and frequency of **F)** $IFN\gamma^+$ cells, and **G)** nMFI of CD107a and GzmB of PD-1^+ or PD-1^- $CD4^+$ T-cells.

All graphs show mean ± SEM. Each dot represents data of an individual mouse. Statistical analysis was performed using Mann-Whitney test. Comparison of matched samples was performed using Wilcoxon matched-pairs signed rank test. *p<0.05, **p<0.01. nMFI = normalized median fluorescence intensity; Tx = transplantation.

Suppl. Figure 6: Accumulation of cytotoxic T_R1 cells in the TCL1 AT mouse model of CLL

Rag2$^{-/-}$ mice were transplanted i.v. with CD4$^+$ T-cells or PBS on day -1, and the following day with (w/) or without (w/o) leukemic splenocytes of Eµ-TCL1 mice (CLL). **A)** Number of CD4$^+$ T-cells per µl blood over time. **B)** Spleen weight at endpoint, 4 weeks after transfer of leukemic cells. **C)** Number of CD5$^+$ CD19$^+$ CLL cells per spleen. **D)** Representative dot plots and frequency of T_R1 cells (EOMES$^+$ PD-1$^+$) in the spleen of mice w/ or w/o CLL transfer. **E)** Frequency of LAG3$^+$ cells out of splenic T_R1 cells. **F)** Frequency of KI-67$^+$ cells out of T_R1 cells in spleen. **G)-H)** Splenocytes were stimulated *ex vivo* with PMA/ionomycin and cytokine expression was analyzed by intracellular flow cytometry. **G)** Frequency of IFNγ$^+$ cells, as well as **H)** nMFI of CD107a and GzmB of T_R1 cells. **I)** t-SNE plot of concatenated, splenic FOXP3$^-$ CD4$^+$ T-cells of mice w/o and w/ TCL1 AT (CLL) based on flow cytometry data of EOMES, PD-1, LAG3 and KI-67 expression (top). Cells from samples w/o and w/ CLL transfer are depicted separately (middle left) and overlaid (middle right). Bottom part shows the clustering of T_R1 cells w/o and w/ CLL (left) and the respective position in the t-SNE plot, separately and overlaid (right).

All graphs show mean ± SEM. In B)-H), each dot represents data of an individual mouse. Statistical analysis was performed using Mann-Whitney test. *p<0.05, **p<0.01. Tx = transplantation. nMFI = normalized median fluorescence intensity.

Suppl. Figure 7: EOMES$^+$ PD-1$^+$ CD4$^+$ T-cells of *Il10Rb*$^{-/-}$ origin cluster distinct of WT T_R1 cells in the TCL1 AT mouse model of CLL

Rag2$^{-/-}$ mice were transplanted i.v. with PBS or CD4$^+$ T-cells of WT or *Il10rb*$^{-/-}$ origin on day -1, and the following day and the following day with leukemic splenocytes of Eµ-TCL1 mice. Spleen samples were analyzed by flow cytometry. **A)** Numbers of CD4$^+$ T-cells per spleen are shown. **B)** t-SNE plot of concatenated, splenic WT FOXP3$^-$ CD4$^+$ T-cells cells based on the flow cytometry data of EOMES, PD-1, LAG3 and KI-67 expression (upper row). Distribution of WT and *Il10rb*$^{-/-}$ CD4$^+$ T-cells in the t-SNE

plot separately and overlaid (middle row). Gating of T_R1 cells in concatenated samples is depicted in the bottom row.
