# Supplementary figures and images for: EOMES and IL-10 regulate antitumor activity of T regulatory type 1 CD4+ T cells in chronic lymphocytic leukemia
Source: Leukemia. 2021 Feb 1;35(8):2311–24. doi: 10.1038/s41375-021-01136-1 (PMC8324479; doi:10.1038/s41375-021-01136-1)

Suppl. Figure 1

A

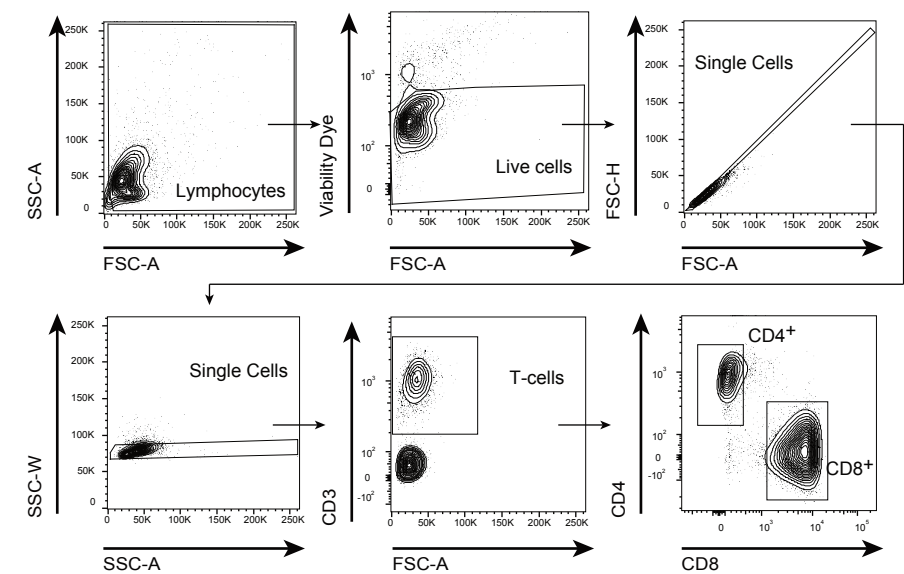

B

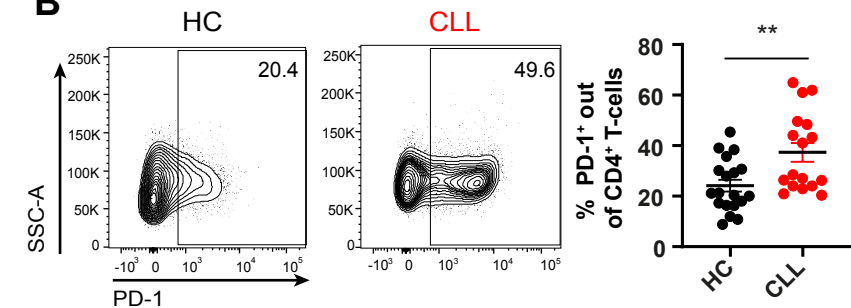

C

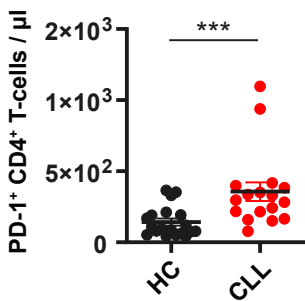

D

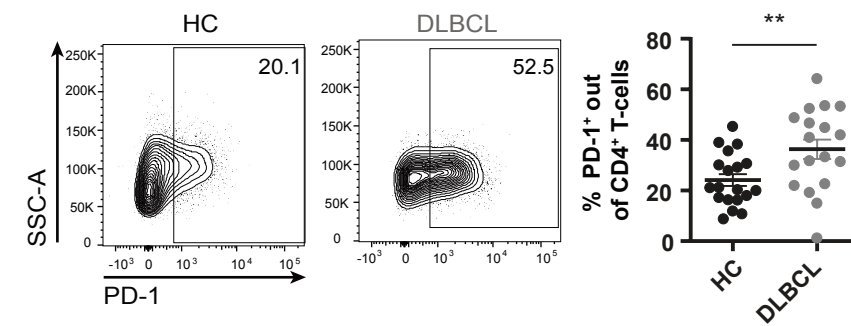

E

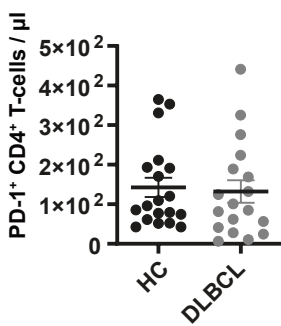

F

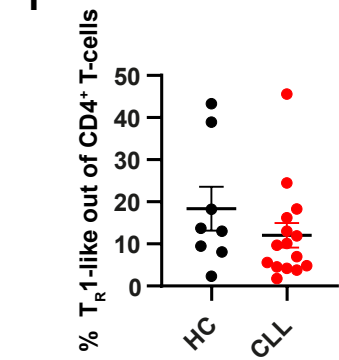

G

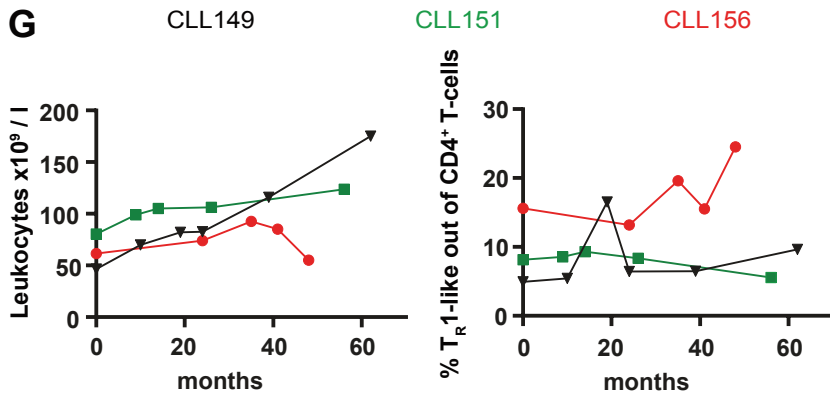

Supplement: Supplementary file 2 — Supplementary Figure 1 [file 41375_2021_1136_MOESM2_ESM.pdf]

Suppl. Figure 2

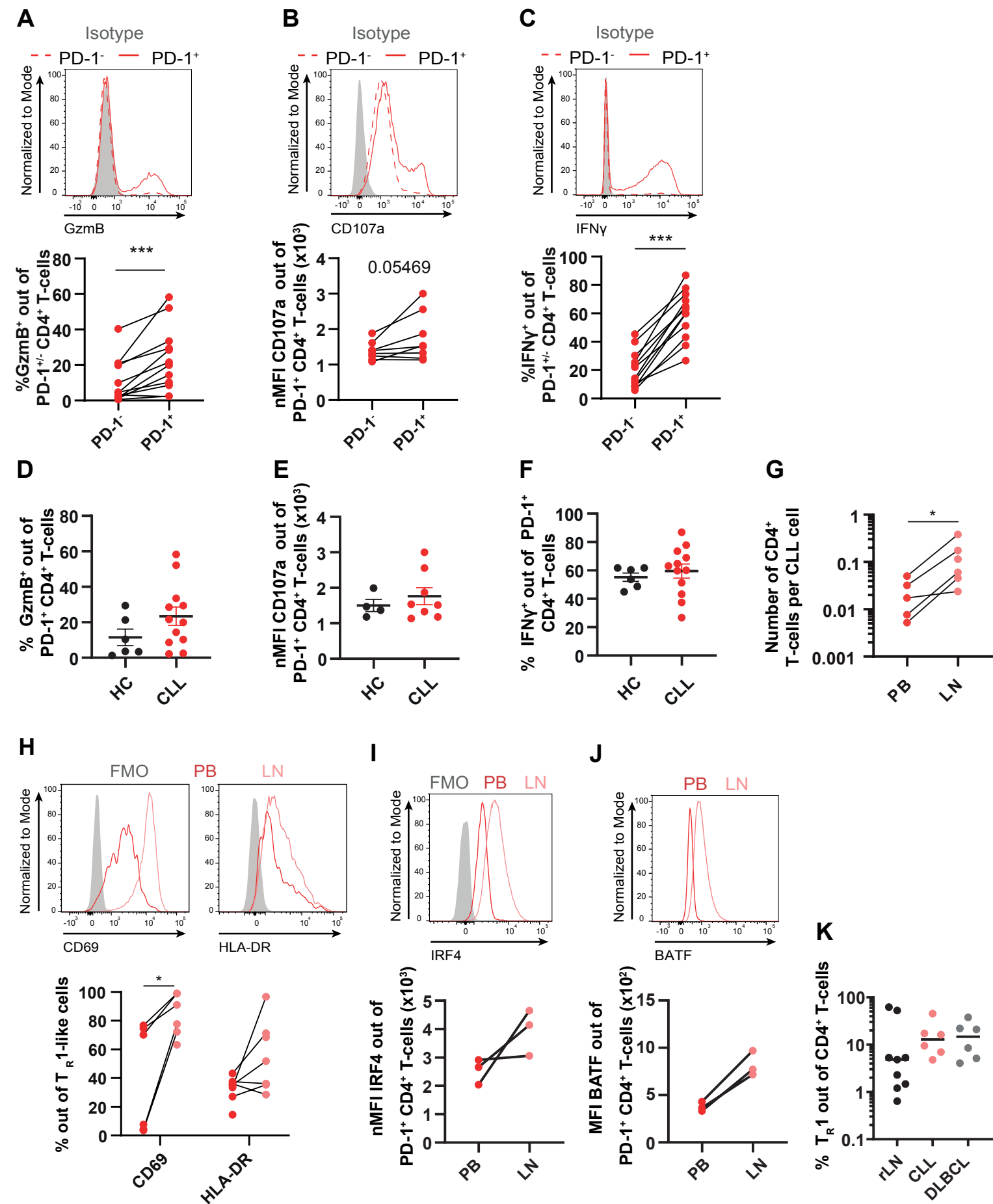

Supplement: Supplementary file 3 — Supplementary Figure 2 [file 41375_2021_1136_MOESM3_ESM.pdf]

Suppl. Figure 3

A

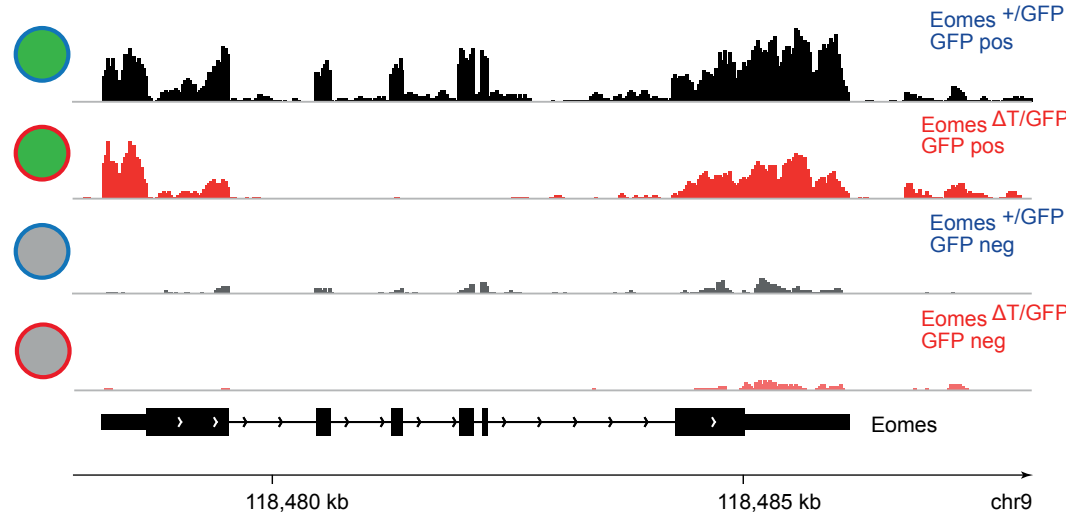

B

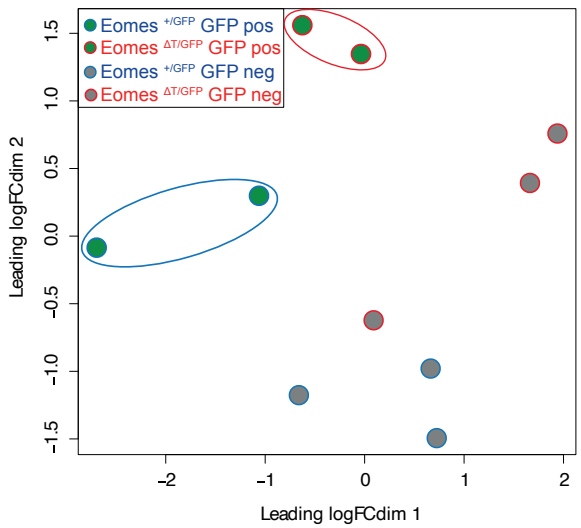

C

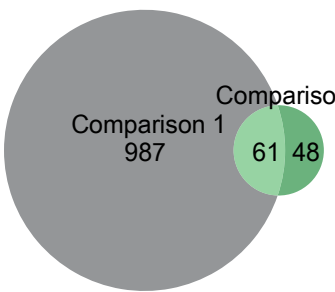

D

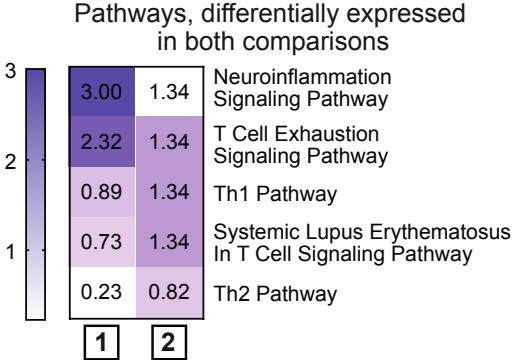

E

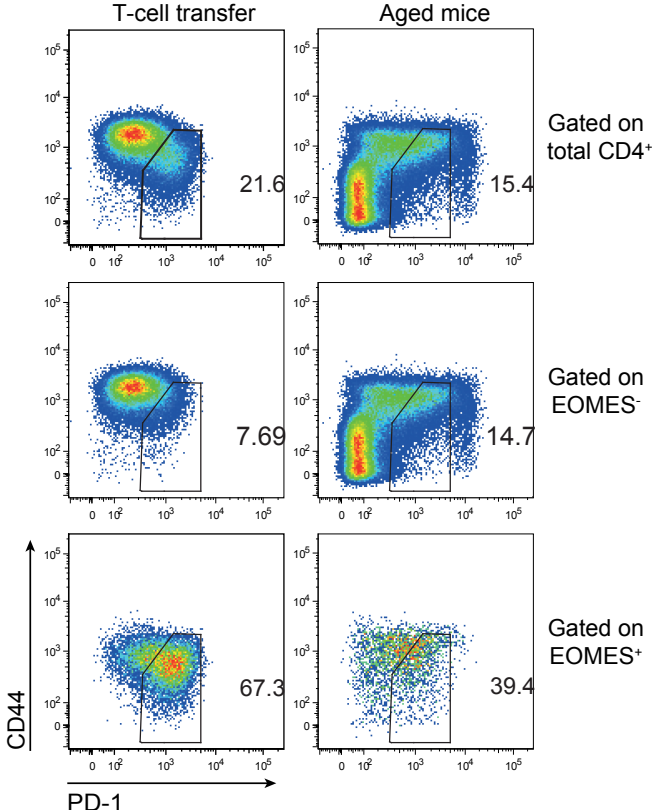

F

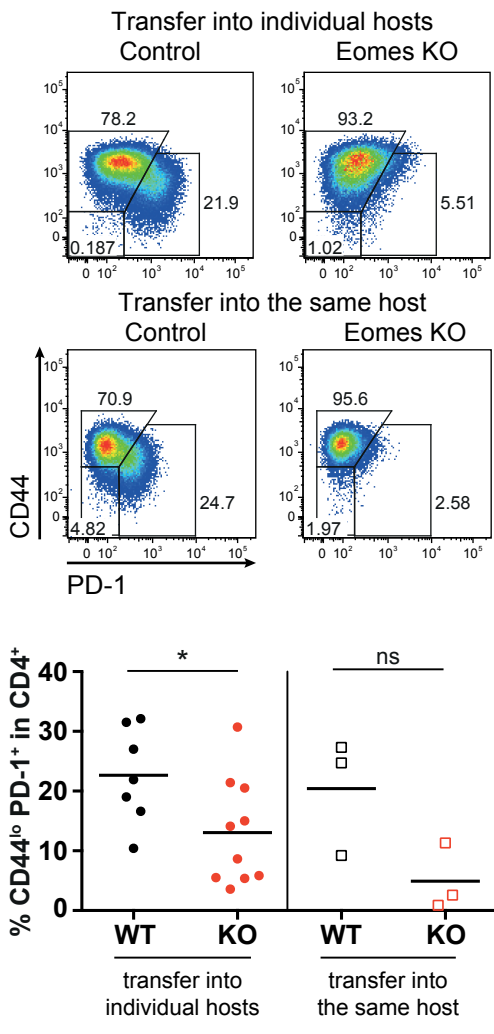

Supplement: Supplementary file 4 — Supplementary Figure 3 [file 41375_2021_1136_MOESM4_ESM.pdf]

Suppl. Figure 4

A

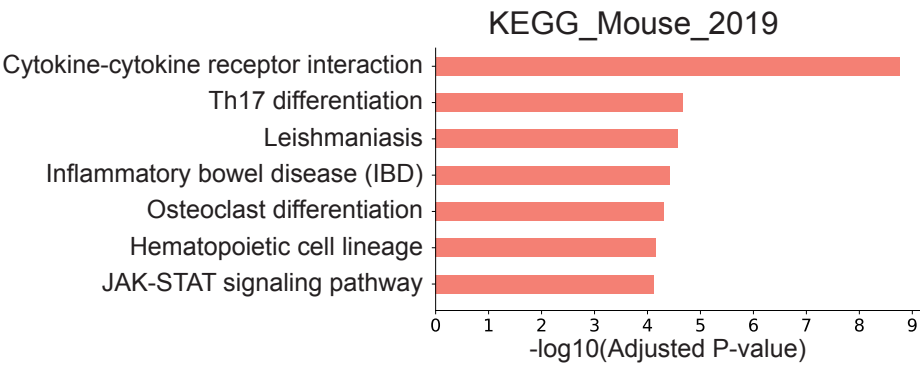

B

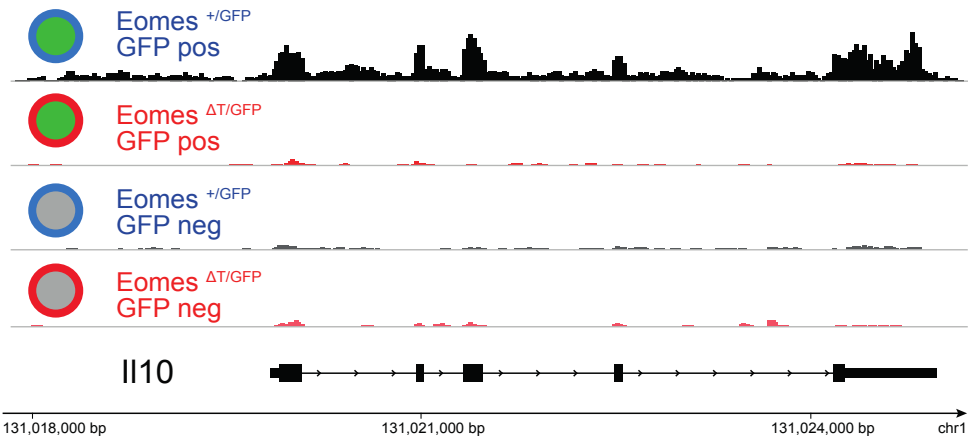

Supplement: Supplementary file 5 — Supplementary Figure 4 [file 41375_2021_1136_MOESM5_ESM.pdf]

Suppl. Figure 5

**A**

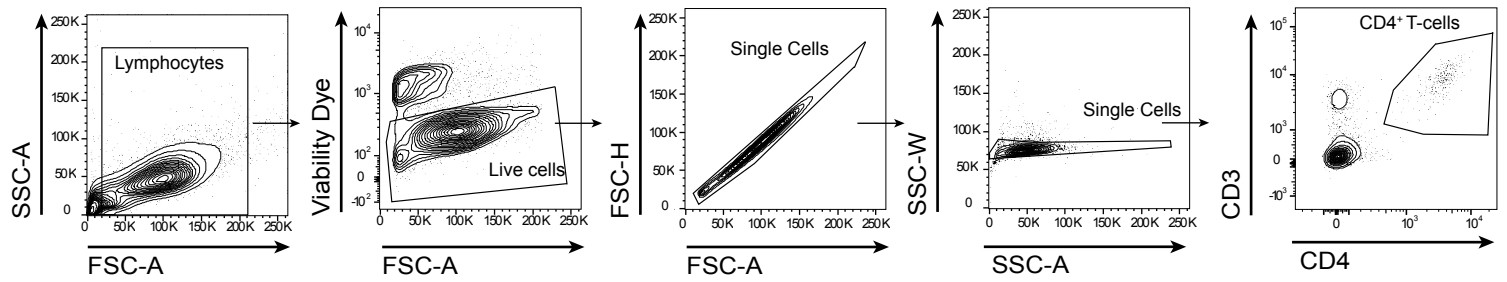

**B**

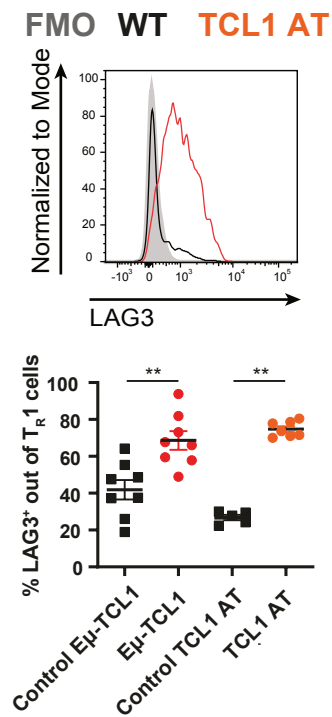

**C**

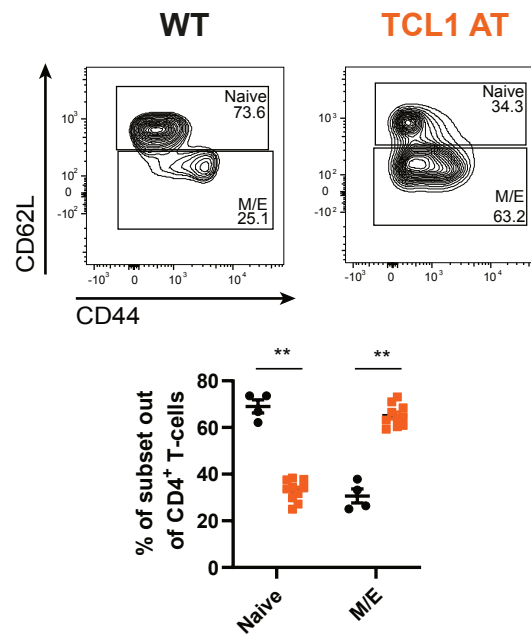

**D**

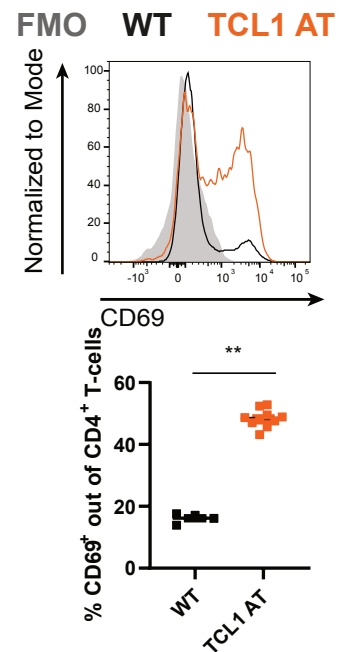

**E**

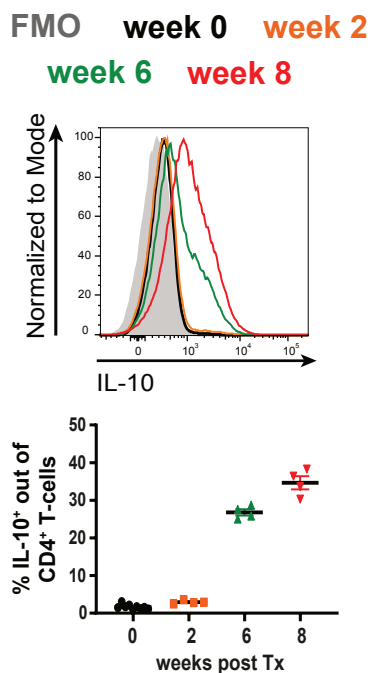

**F**

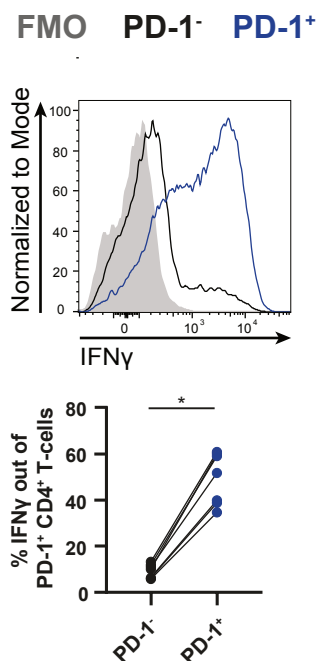

**G**

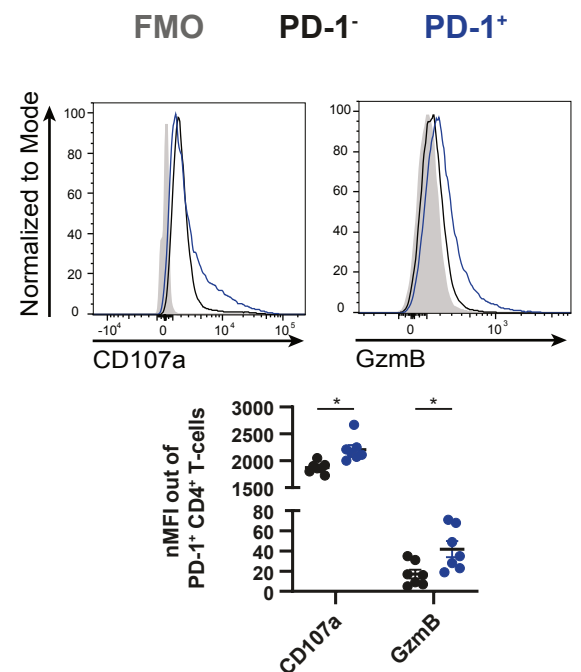

Supplement: Supplementary file 6 — Supplementary Figure 5 [file 41375_2021_1136_MOESM6_ESM.pdf]

Suppl. Figure 6

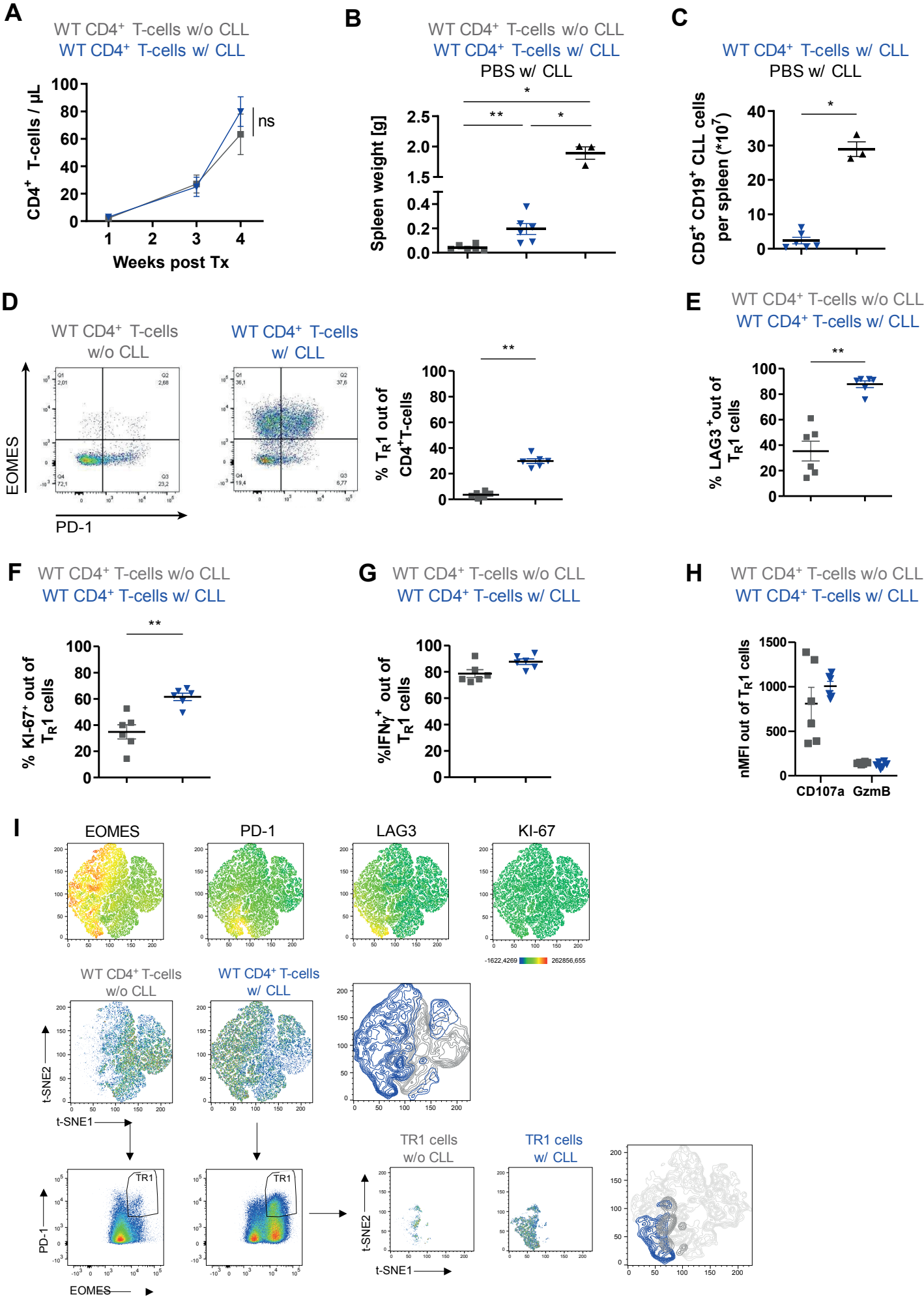

Supplement: Supplementary file 7 — Supplementary Figure 6 [file 41375_2021_1136_MOESM7_ESM.pdf]

Suppl. Figure 7

A

WT CD4<sup>+</sup> T-cells w/ CLL  
*Il10rb*<sup>-/-</sup> CD4<sup>+</sup> T-cells w/ CLL

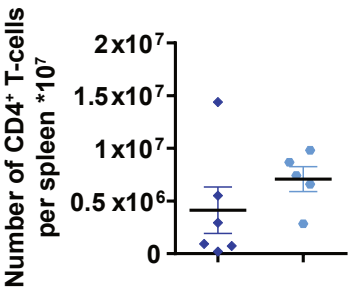

B

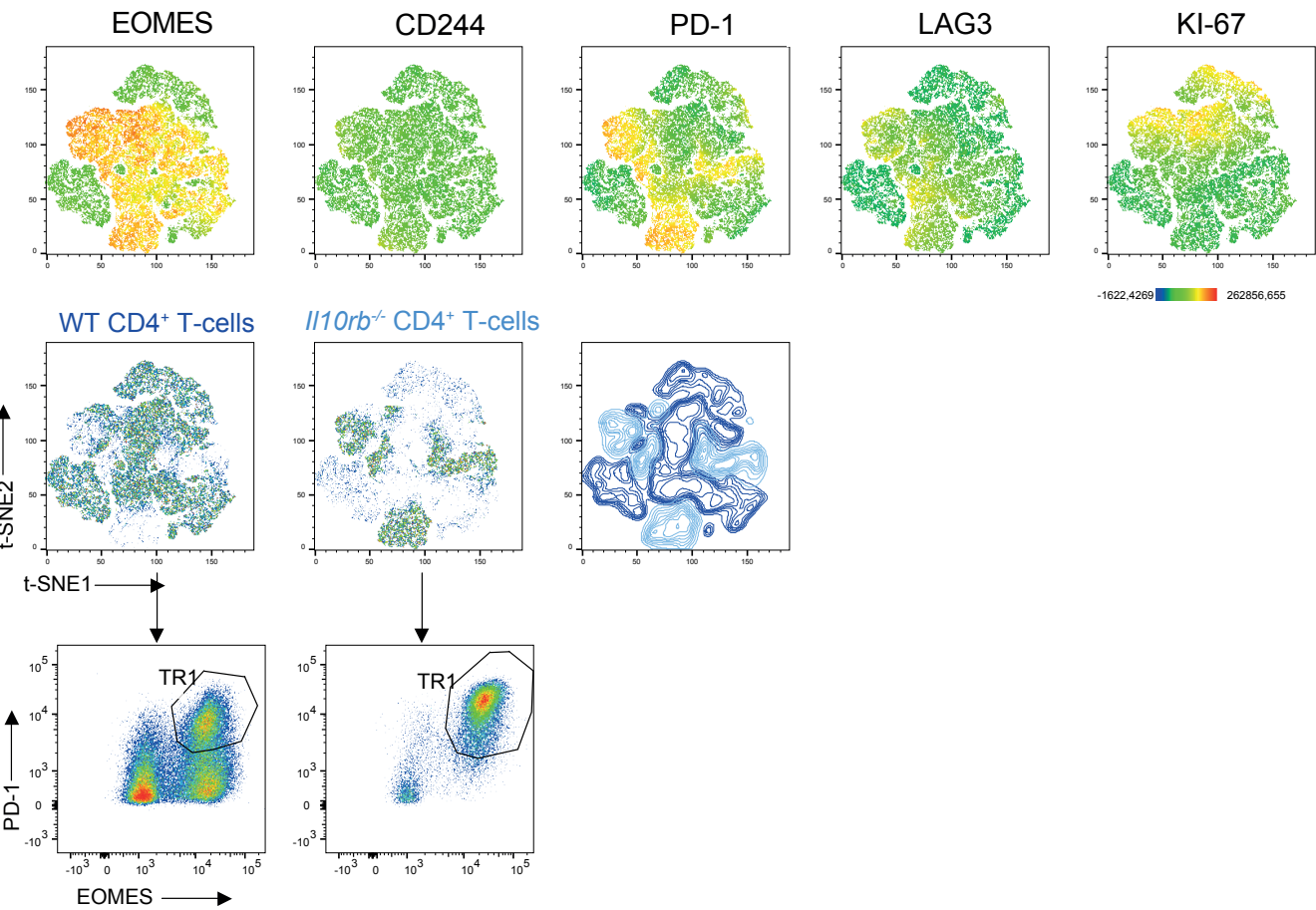

Supplement: Supplementary file 8 — Supplementary Figure 7 [file 41375_2021_1136_MOESM8_ESM.pdf]
