# Supplementary material for: EOMES and IL-10 regulate antitumor activity of T regulatory type 1 CD4+ T cells in chronic lymphocytic leukemia
Source: Leukemia. 2021 Feb 1;35(8):2311–24. doi: 10.1038/s41375-021-01136-1 (PMC8324479; doi:10.1038/s41375-021-01136-1)
Supplement: Supplementary file 9 — Suppl. Table 6_sheet 1_comparison 1 [file 41375_2021_1136_MOESM9_ESM.pdf]

Comparison 1

ENSID	baseMean	log2FoldChar	lfcSE	pvalue	padj	Status	external_gen	gene_coordinates	gene_type	strand
ENSMUSG00000041272.11	942,978254	4,59254476	0,32061953	7,67E-50	1,02E-45	UP	Tox	4:6686353-6991557	protein_coding	-
ENSMUSG00000032446.14	537,345345	4,42200701	0,37363371	1,01E-35	1,68E-32	UP	Eomes	9:118478212-118486132	protein_coding	+
ENSMUSG00000025997.13	882,374523	2,64197277	0,25074758	2,59E-27	3,12E-24	UP	Ikzf2	1:69531214-69687245	protein_coding	-
ENSMUSG00000076258.1	3118,55568	2,22525873	0,23093949	2,03E-23	1,68E-20	UP	Gm23935	16:11144125-11144181	miRNA	+
ENSMUSG00000030336.14	378,673159	2,84835862	0,30217192	1,70E-22	1,19E-19	UP	Cd27	6:125232622-125237010	protein_coding	-
ENSMUSG00000042385.14	553,832815	2,70217457	0,29631992	1,91E-21	1,18E-18	UP	Gzmk	13:113171608-113225908	protein_coding	-
ENSMUSG00000032496.7	4129,86	1,94836238	0,21366661	4,20E-21	2,32E-18	UP	Ltf	9:111019271-111042767	protein_coding	+
ENSMUSG00000026285.7	686,480485	2,25222917	0,24930741	9,88E-21	5,23E-18	UP	Pdcd1	1:94038305-94052553	protein_coding	-
ENSMUSG00000016529.5	122,852133	4,05825893	0,45986192	6,66E-20	3,39E-17	UP	Il10	1:131019845-131024974	protein_coding	+
ENSMUSG00000032089.16	1458,48194	2,1329648	0,24460453	1,08E-19	5,30E-17	UP	Il10ra	9:45253837-45269149	protein_coding	-
ENSMUSG00000026193.15	505,419478	2,56229061	0,29730449	2,27E-19	1,04E-16	UP	Fn1	1:71585520-71653200	protein_coding	-
ENSMUSG00000004668.14	1015,94497	2,05553575	0,23649105	2,20E-19	1,04E-16	UP	Abca13	11:9191942-9684259	protein_coding	+
ENSMUSG00000024164.15	831,939819	2,18170945	0,25182302	2,36E-19	1,04E-16	UP	C3	17:57203970-57228136	protein_coding	-
ENSMUSG00000040249.15	359,173496	2,42365831	0,29684065	1,81E-17	7,05E-15	UP	Lrp1	10:127538161-127621148	protein_coding	-
ENSMUSG00000069516.7	2040,29817	1,77748023	0,21996392	4,86E-17	1,84E-14	UP	Lyz2	10:117277334-117282274	protein_coding	-
ENSMUSG00000000204.15	694,578652	2,27356226	0,2914449	1,77E-16	6,51E-14	UP	Slfn4	11:83175186-83190221	protein_coding	+
ENSMUSG00000035042.2	2828,1942	1,76539156	0,22451588	1,85E-16	6,61E-14	UP	Ccl5	11:83525778-83530518	protein_coding	-
ENSMUSG00000026822.14	1881,7352	1,72583836	0,22276188	5,96E-16	2,02E-13	UP	Lcn2	2:32384633-32388252	protein_coding	-
ENSMUSG00000052212.6	971,542062	1,92852029	0,25310559	1,29E-15	4,18E-13	UP	Cd177	7:24743983-24760311	protein_coding	-
ENSMUSG00000038679.16	882,043287	1,80156452	0,23851751	2,46E-15	7,41E-13	UP	Trps1	15:50654752-50890463	protein_coding	-
ENSMUSG00000015950.13	799,697642	1,8875624	0,25279161	4,16E-15	1,17E-12	UP	Ncf1	5:134220053-134229625	protein_coding	-
ENSMUSG00000032484.8	5209,88267	1,54295769	0,20622156	4,95E-15	1,36E-12	UP	Ngp	9:110419747-110423012	protein_coding	+
ENSMUSG00000032020.15	561,1846	1,92350731	0,25992682	7,79E-15	2,02E-12	UP	Ubash3b	9:41011098-41161697	protein_coding	-
ENSMUSG00000047507.12	2859,41326	1,80983819	0,24672039	8,53E-15	2,17E-12	UP	Baiap3	17:25242659-25256364	protein_coding	-
ENSMUSG00000026950.16	720,461278	1,9285095	0,26506717	1,37E-14	3,43E-12	UP	Neb	2:52136647-52338798	protein_coding	-
ENSMUSG00000018925.3	342,826262	2,14903234	0,30135279	5,44E-14	1,31E-11	UP	Heatr9	11:83511736-83522175	protein_coding	-
ENSMUSG00000099954.1	118,170896	3,3087155	0,46380946	5,55E-14	1,31E-11	UP	Gm28112	1:69660187-69662061	sense_intronic	-
ENSMUSG00000038357.10	1442,35067	1,66430984	0,23553616	9,42E-14	2,12E-11	UP	Camp	9:109847379-109849617	protein_coding	-
ENSMUSG00000009350.13	739,436644	1,89813683	0,26990133	1,16E-13	2,56E-11	UP	Mpo	11:87793581-87804413	protein_coding	+
ENSMUSG00000015133.17	669,399405	2,07862097	0,30233746	1,83E-13	3,97E-11	UP	Lrrk1	7:66226912-66388350	protein_coding	-
ENSMUSG00000031722.9	612,431732	1,84347502	0,26533226	2,30E-13	4,91E-11	UP	Hp	8:109575130-109579172	protein_coding	-
ENSMUSG00000046841.4	292,01399	2,40306076	0,3533976	3,43E-13	7,11E-11	UP	Ckap4	10:84526305-84534036	protein_coding	-
ENSMUSG00000060012.7	1414,30336	1,5396837	0,22380441	4,14E-13	8,44E-11	UP	Kif13b	14:64652531-64806296	protein_coding	+
ENSMUSG00000035493.9	394,925579	1,98062759	0,29594814	1,27E-12	2,44E-10	UP	Tgfbi	13:56609603-56639339	protein_coding	+
ENSMUSG00000026826.13	318,937703	2,46073795	0,38028039	1,72E-12	3,25E-10	UP	Nr4a2	2:57106830-57124003	protein_coding	-
ENSMUSG00000028859.14	246,238123	2,32573837	0,35476524	2,04E-12	3,80E-10	UP	Csf3r	4:126024550-126044440	protein_coding	+
ENSMUSG00000015340.10	1378,81057	1,51834389	0,23033139	2,95E-12	5,36E-10	UP	Cybb	X:9435252-9487771	protein_coding	-
ENSMUSG00000021614.16	248,102837	2,17007287	0,33342507	4,31E-12	7,56E-10	UP	Vcan	13:89655312-89742509	protein_coding	-
ENSMUSG00000021457.14	316,248498	1,99069025	0,30602355	4,75E-12	8,17E-10	UP	Syk	13:52583173-52648792	protein_coding	+
ENSMUSG00000074607.11	175,03575	2,55473785	0,40139641	6,49E-12	1,06E-09	UP	Tox2	2:163203125-163324170	protein_coding	+
ENSMUSG00000098178.1	36750,451	1,66739298	0,26251902	6,43E-12	1,06E-09	UP	Gm42418	17:39846958-39848788	lincRNA	+
ENSMUSG00000024548.11	434,477008	2,09458261	0,33737799	1,42E-11	2,27E-09	UP	Setbp1	18:78750380-79109391	protein_coding	-
ENSMUSG00000097971.3	350221,196	1,26950386	0,19916881	1,45E-11	2,29E-09	UP	Gm26917	17:39843013-39846341	lincRNA	+
ENSMUSG00000033066.15	614,819392	1,62359836	0,25751499	1,83E-11	2,81E-09	UP	Gas7	11:67455437-67688990	protein_coding	+
ENSMUSG00000000157.15	287,667607	2,02561374	0,32607515	2,74E-11	4,13E-09	UP	Itgb2l	16:96422288-96443619	protein_coding	-
ENSMUSG00000030124.2	426,937463	2,06680515	0,33997482	2,89E-11	4,31E-09	UP	Lag3	6:124904361-124911705	protein_coding	-
ENSMUSG00000041268.16	175,158932	2,36064218	0,38117656	3,05E-11	4,45E-09	UP	Dmxl2	9:54365158-54501760	protein_coding	-
ENSMUSG00000071713.4	360,436707	1,93753036	0,31307841	3,03E-11	4,45E-09	UP	Csf2rb	15:78325990-78351000	protein_coding	+
ENSMUSG00000024053.10	168,991241	2,41958395	0,39037418	3,16E-11	4,54E-09	UP	Emilin2	17:71252176-71310965	protein_coding	-
ENSMUSG00000037095.7	210,95977	2,246238	0,36391547	3,60E-11	5,08E-09	UP	Lrg1	17:56119680-56121946	protein_coding	-
ENSMUSG00000029408.13	258,070503	2,0463834	0,33875849	6,94E-11	9,19E-09	UP	Abcb9	5:124061530-124095798	protein_coding	-
ENSMUSG00000006574.15	187,814372	2,37596096	0,39794413	8,84E-11	1,15E-08	UP	Slc4a1	11:102348824-102366203	protein_coding	-
ENSMUSG00000034573.14	1094,87435	1,4280296	0,23533311	8,88E-11	1,15E-08	UP	Ptpn13	5:103425192-103598303	protein_coding	+
ENSMUSG00000100815.1	75,9541852	3,25972783	0,54250256	9,77E-11	1,26E-08	UP	Gm29112	1:69595036-69598522	sense_intronic	-
ENSMUSG00000031824.14	224,680709	2,08012369	0,34636509	1,08E-10	1,36E-08	UP	6430548M08	8:120114152-120165306	protein_coding	+
ENSMUSG00000039109.15	260,761463	2,01707308	0,33629731	1,12E-10	1,39E-08	UP	F13a1	13:36867178-37050244	protein_coding	-
ENSMUSG00000017737.2	364,731004	1,80214172	0,30059981	1,18E-10	1,44E-08	UP	Mmp9	2:164940780-164955850	protein_coding	+
ENSMUSG00000025877.14	268,747135	1,94056131	0,32756244	1,78E-10	2,15E-08	UP	Hk3	13:55005985-55021385	protein_coding	-
ENSMUSG00000021281.15	250,802929	1,93435352	0,3296377	2,47E-10	2,89E-08	UP	Tnfaip2	12:111442469-111455018	protein_coding	+
ENSMUSG00000059326.6	184,193458	2,18211756	0,37482765	2,87E-10	3,34E-08	UP	Csf2ra	19:61224402-61228418	protein_coding	-
ENSMUSG00000026475.7	199,377663	2,42196847	0,42989913	3,29E-10	3,75E-08	UP	Rgs16	1:153740349-153745468	protein_coding	+
ENSMUSG00000048120.16	703,195265	1,50197625	0,25731223	3,26E-10	3,75E-08	UP	Entpd1	19:40612366-40741602	protein_coding	+
ENSMUSG00000058624.12	468,382486	1,65394396	0,283254	3,47E-10	3,92E-08	UP	Gda	19:21391307-21473445	protein_coding	-
ENSMUSG00000042724.7	152,282147	2,34081429	0,4075803	4,43E-10	4,93E-08	UP	Map3k9	12:81714950-81781170	protein_coding	-
ENSMUSG00000026480.12	307,226607	1,78859358	0,31155331	5,56E-10	6,08E-08	UP	Ncf2	1:152800194-152836991	protein_coding	+
ENSMUSG00000040809.10	3864,53938	1,31973295	0,22995846	6,30E-10	6,84E-08	UP	Chil3	3:106147554-106167564	protein_coding	-
ENSMUSG00000021069.16	407,119036	1,63949725	0,29362472	1,45E-09	1,51E-07	UP	Pygl	12:70190811-70231488	protein_coding	-
ENSMUSG00000037202.5	270,33615	1,89691699	0,34410159	1,53E-09	1,58E-07	UP	Prf1	10:61297836-61304263	protein_coding	+
ENSMUSG00000024621.15	278,471458	1,7751754	0,32434019	2,45E-09	2,44E-07	UP	Csf1r	18:61105572-61132149	protein_coding	+
ENSMUSG00000078942.10	156,697925	2,07204731	0,38520131	3,98E-09	3,90E-07	UP	Naip6	13:100281121-100317674	protein_coding	-
ENSMUSG00000028246.13	70,818685	2,94561882	0,55166167	4,25E-09	4,14E-07	UP	Faxc	4:21931329-21996839	protein_coding	+
ENSMUSG00000057729.12	247,632424	1,94282381	0,37104996	8,46E-09	7,78E-07	UP	Prtn3	10:79874476-79883174	protein_coding	+
ENSMUSG00000031530.6	249,992738	1,73564643	0,33079898	9,07E-09	8,28E-07	UP	Dusp4	8:34807297-34819894	protein_coding	+
ENSMUSG00000044340.7	231,19356	1,75996611	0,33719212	1,02E-08	9,18E-07	UP	Phlpp1	1:106171752-106394250	protein_coding	+
ENSMUSG00000043940.14	147,310432	2,07531201	0,40062998	1,20E-08	1,07E-06	UP	Wdfy3	5:101832956-102069921	protein_coding	-
ENSMUSG00000050600.5	936,320467	1,25598771	0,23975923	1,21E-08	1,07E-06	UP	Zfp831	2:174643534-174710832	protein_coding	+
ENSMUSG00000032548.14	67,7987134	3,07355492	0,60132911	1,22E-08	1,07E-06	UP	Slco2a1	9:102988712-103096002	protein_coding	+
ENSMUSG00000015968.16	46,7628638	4,15321163	0,8032863	1,41E-08	1,23E-06	UP	Cacna1d	14:30039949-30491156	protein_coding	-
ENSMUSG00000064147.6	113,073106	2,47697586	0,49264895	1,45E-08	1,25E-06	UP	Rab44	17:29135056-29148980	protein_coding	+
ENSMUSG00000047798.15	191,058269	1,88315368	0,36723704	1,60E-08	1,36E-06	UP	Cd300lf	11:115116214-115133992	protein_coding	-
ENSMUSG00000031543.18	110,899882	2,38590971	0,47258274	1,72E-08	1,46E-06	UP	Ank1	8:22974844-23150497	protein_coding	+
ENSMUSG00000051747.14	7690,71217	1,37171634	0,27172569	1,84E-08	1,53E-06	UP	Ttn	2:76703980-76982547	protein_coding	-

ENSMUSG00000036273.15	202,492725	1,81510099	0,3557274	1,87E-08	1,54E-06	UP	Lrrk2	15:91673175-91816120	protein_coding	+
ENSMUSG00000034930.15	94,0028468	2,43513306	0,47964268	1,95E-08	1,60E-06	UP	Rtkn	6:83135463-83152579	protein_coding	+
ENSMUSG00000056071.12	6849,27987	1,06632545	0,20591932	2,02E-08	1,64E-06	UP	S100a9	3:90692632-90695721	protein_coding	-
ENSMUSG00000066363.12	1072,16297	1,23744869	0,24209627	2,14E-08	1,73E-06	UP	Serpina3f	12:104214544-104221129	protein_coding	+
ENSMUSG00000058099.15	207,209627	1,80163873	0,35456765	2,18E-08	1,75E-06	UP	Nfam1	15:82997721-83033306	protein_coding	-
ENSMUSG00000058818.13	257,639015	1,77598091	0,35263029	2,36E-08	1,87E-06	UP	Pirb	7:3711409-3720391	protein_coding	-
ENSMUSG00000078247.3	100,714255	2,53255132	0,51400319	2,37E-08	1,87E-06	UP	Airn	17:12741311-12860122	antisense	+
ENSMUSG00000031785.15	61,4047763	2,90122331	0,58617016	3,76E-08	2,90E-06	UP	Adgrg1	8:94977109-95014208	protein_coding	+
ENSMUSG00000021061.15	104,749338	2,37658109	0,48577588	3,84E-08	2,94E-06	UP	Sptb	12:76580488-76710547	protein_coding	-
ENSMUSG00000004609.11	157,174449	2,00308713	0,40434974	3,90E-08	2,97E-06	UP	Cd33	7:43524216-43544428	protein_coding	-
ENSMUSG00000024451.8	82,0069274	2,58516465	0,52875945	4,30E-08	3,26E-06	UP	Arap3	18:37972624-37997574	protein_coding	-
ENSMUSG00000031933.17	93,4024228	2,34590653	0,47780294	4,48E-08	3,37E-06	UP	Izumo1r	9:14885814-14903949	protein_coding	-
ENSMUSG00000051339.8	102,092405	2,70127211	0,58689064	4,51E-08	3,38E-06	UP	2900026A02I	5:113086323-113163351	protein_coding	-
ENSMUSG00000037902.18	351,602641	1,50493117	0,30523758	5,04E-08	3,75E-06	UP	Sirpa	2:129592835-129632228	protein_coding	+
ENSMUSG00000038213.7	604,542667	1,28536013	0,25969701	5,38E-08	3,98E-06	UP	Tapbpl	6:125223933-125231860	protein_coding	-
ENSMUSG00000019982.14	341,334905	1,47671593	0,30071737	5,66E-08	4,17E-06	UP	Myb	10:21124936-21160905	protein_coding	-
ENSMUSG00000018168.8	3910,27596	1,04533713	0,2095092	5,92E-08	4,33E-06	UP	Ikzf3	11:98464902-98546031	protein_coding	-
ENSMUSG00000029455.14	249,88739	1,67078607	0,34343314	6,30E-08	4,58E-06	UP	Aldh2	5:121566027-121593824	protein_coding	-
ENSMUSG00000024074.7	237,558036	2,02700676	0,4367661	6,74E-08	4,88E-06	UP	Crim1	17:78200248-78376592	protein_coding	+
ENSMUSG00000045636.16	137,387787	2,10835897	0,43730875	6,97E-08	5,02E-06	UP	Mtus1	8:40990914-41133726	protein_coding	-
ENSMUSG00000045071.13	218,257014	1,70533875	0,3556904	8,26E-08	5,88E-06	UP	E130308A19I	4:59626211-59761439	protein_coding	+
ENSMUSG00000058297.16	163,025207	1,92030019	0,40584991	9,40E-08	6,65E-06	UP	Spock2	10:60106219-60135198	protein_coding	+
ENSMUSG00000003283.13	219,254481	1,72006949	0,36088603	9,77E-08	6,84E-06	UP	Hck	2:153108468-153151441	protein_coding	+
ENSMUSG00000002190.13	93,3730916	2,40406413	0,51951771	1,22E-07	8,37E-06	UP	Clgn	8:83389867-83428552	protein_coding	+
ENSMUSG00000020593.14	1009,22287	1,23812191	0,26114142	1,33E-07	9,06E-06	UP	Lpin1	12:16535669-16589770	protein_coding	-
ENSMUSG00000027073.5	32,1825617	5,03194263	1,05048011	1,42E-07	9,62E-06	UP	Prg2	2:84980461-84983632	protein_coding	+
ENSMUSG00000023830.13	2610,58746	1,02169754	0,21325166	1,51E-07	1,02E-05	UP	Igf2r	17:12682406-12769664	protein_coding	-
ENSMUSG00000002111.8	170,207214	1,79932656	0,38553304	1,66E-07	1,11E-05	UP	Spi1	2:91082390-91115756	protein_coding	+
ENSMUSG00000066278.6	482,974498	1,2710526	0,27168374	2,07E-07	1,36E-05	UP	Vps37b	5:124004641-124032270	protein_coding	-
ENSMUSG00000039852.16	548,484701	1,22979897	0,26345454	2,19E-07	1,43E-05	UP	Rere	4:150281646-150621966	protein_coding	+
ENSMUSG00000022892.10	308,376631	1,50270937	0,32618966	2,31E-07	1,50E-05	UP	App	16:84954440-85173707	protein_coding	-
ENSMUSG00000020125.6	202,858641	1,80924924	0,3952035	2,69E-07	1,71E-05	UP	Elane	10:79886312-79888216	protein_coding	+
ENSMUSG00000028977.16	94,3260372	2,14216395	0,4739407	3,11E-07	1,94E-05	UP	Casz1	4:148804429-148954889	protein_coding	+
ENSMUSG00000068747.14	212,371515	1,64541297	0,36191327	3,20E-07	1,98E-05	UP	Sort1	3:108284082-108361511	protein_coding	+
ENSMUSG00000026131.17	392,945599	1,35413421	0,29933641	3,75E-07	2,31E-05	UP	Dst	1:33908225-34308661	protein_coding	+
ENSMUSG00000030589.15	145,861197	1,80587738	0,40185936	3,82E-07	2,34E-05	UP	Rasgrp4	7:29134851-29153961	protein_coding	+
ENSMUSG00000029869.7	192,231995	1,78712828	0,40609645	3,94E-07	2,39E-05	UP	Ephb6	6:41605482-41620509	protein_coding	+
ENSMUSG00000029406.15	693,615681	1,15983746	0,25542388	4,16E-07	2,50E-05	UP	Pitpnm2	5:124118690-124249760	protein_coding	-
ENSMUSG00000028874.14	211,000607	1,56875402	0,34949918	4,34E-07	2,60E-05	UP	Fgr	4:132974095-133001910	protein_coding	+
ENSMUSG00000051354.13	46,5671657	2,98154095	0,67259487	4,43E-07	2,64E-05	UP	Samd3	10:26229707-26272172	protein_coding	+
ENSMUSG00000060063.9	266,023408	1,55240778	0,34757709	4,57E-07	2,71E-05	UP	Alox5ap	5:149264767-149288153	protein_coding	+
ENSMUSG00000038623.9	310,798727	1,3859007	0,30873461	4,68E-07	2,77E-05	UP	Tm6sf1	7:81859001-81884434	protein_coding	+
ENSMUSG00000042082.6	1780,34446	1,01047507	0,22263967	5,00E-07	2,94E-05	UP	Arsb	13:93771679-93943016	protein_coding	+
ENSMUSG00000002028.12	6379,62772	0,94355791	0,20527606	5,39E-07	3,13E-05	UP	Kmt2a	9:44803355-44881296	protein_coding	-
ENSMUSG00000046805.9	767,124883	1,1382867	0,25321887	5,63E-07	3,26E-05	UP	Mpeg1	19:12460779-12465284	protein_coding	+
ENSMUSG00000071552.4	733,075441	1,32991869	0,30622603	6,80E-07	3,90E-05	UP	Tigit	16:43648861-43664184	protein_coding	-
ENSMUSG00000029516.19	1170,14642	1,03377206	0,23084085	6,87E-07	3,92E-05	UP	Cit	5:115845278-116008947	protein_coding	+
ENSMUSG00000027035.10	593,989076	1,26320485	0,28902809	7,39E-07	4,20E-05	UP	Cers6	2:68861441-69114282	protein_coding	+
ENSMUSG00000035004.3	180,893177	1,72447191	0,39947753	7,55E-07	4,27E-05	UP	Igsf6	7:121064067-121074572	protein_coding	-
ENSMUSG00000048612.15	121,773564	1,9204076	0,44369594	7,69E-07	4,34E-05	UP	Myof	19:37899036-38043577	protein_coding	-
ENSMUSG00000028078.14	272,246345	1,38954586	0,31713361	7,74E-07	4,34E-05	UP	Dclk2	3:86786151-86920852	protein_coding	-
ENSMUSG00000045573.9	67,4462328	2,66686186	0,65311786	8,96E-07	5,01E-05	UP	Penk	4:4133531-4138819	protein_coding	-
ENSMUSG00000005640.11	60,8302352	2,69149746	0,64935068	9,62E-07	5,31E-05	UP	Insrr	3:87796951-87816101	protein_coding	+
ENSMUSG00000015222.17	44,9674462	3,06225202	0,74330805	1,04E-06	5,70E-05	UP	Map2	1:66175273-66442583	protein_coding	+
ENSMUSG00000009292.17	135,156573	1,86308472	0,44340478	1,06E-06	5,76E-05	UP	Trpm2	10:77907722-77970563	protein_coding	-
ENSMUSG00000097796.1	75,3864838	2,24335452	0,53101017	1,10E-06	5,93E-05	UP	Gm16702	17:8379045-8389468	lincRNA	-
ENSMUSG00000031012.17	75,2136549	2,26316063	0,53590073	1,11E-06	5,95E-05	UP	Cask	X:13517080-13851367	protein_coding	-
ENSMUSG00000046223.10	114,418144	2,02908154	0,4831837	1,12E-06	5,98E-05	UP	Plaur	7:24462484-24475968	protein_coding	+
ENSMUSG00000029026.16	96,4531943	2,0026195	0,47261021	1,12E-06	5,98E-05	UP	Trp73	4:154056253-154140208	protein_coding	-
ENSMUSG00000022377.16	1324,82622	1,15758367	0,27159615	1,21E-06	6,39E-05	UP	Asap1	15:64086857-64382919	protein_coding	-
ENSMUSG00000062995.12	104,087398	1,91934459	0,45635437	1,33E-06	6,97E-05	UP	Ica1	6:8630527-8778488	protein_coding	-
ENSMUSG00000059901.12	45,1491974	2,88365195	0,69326629	1,47E-06	7,65E-05	UP	Adamts14	10:61197112-61273438	protein_coding	-
ENSMUSG00000003134.10	203,070914	1,56870884	0,3748921	1,50E-06	7,74E-05	UP	Tbc1d8	1:39371492-39478755	protein_coding	-
ENSMUSG00000046207.14	74,9067605	2,24819216	0,54084136	1,51E-06	7,77E-05	UP	Pik3r6	11:68503019-68552698	protein_coding	+
ENSMUSG00000074151.12	9144,67472	0,95772885	0,22170186	1,52E-06	7,79E-05	UP	Nlrc5	8:94472763-94527272	protein_coding	+
ENSMUSG00000022900.14	234,058738	1,50835057	0,36217458	1,56E-06	7,95E-05	UP	Ildr1	16:36693978-36726804	protein_coding	+
ENSMUSG00000032724.5	927,632071	1,03834593	0,24217492	1,61E-06	8,15E-05	UP	Abtb2	2:103566310-103718423	protein_coding	+
ENSMUSG00000027398.13	108,128805	2,0439279	0,50153126	1,66E-06	8,39E-05	UP	Il1b	2:129364570-129371139	protein_coding	-
ENSMUSG00000025701.12	115,507841	2,00014393	0,49024609	1,77E-06	8,82E-05	UP	Alox5	6:116410077-116461178	protein_coding	-
ENSMUSG00000050931.7	95,6049424	2,28044513	0,56795392	1,97E-06	9,79E-05	UP	Sgms2	3:131318985-131491411	protein_coding	-
ENSMUSG00000059316.2	544,892242	1,16269629	0,27725067	1,97E-06	9,79E-05	UP	Slc27a4	2:29802634-29817522	protein_coding	+
ENSMUSG00000035711.4	81,7172571	2,11864582	0,5161157	2,00E-06	9,88E-05	UP	Dok3	13:55523235-55528538	protein_coding	-
ENSMUSG00000079056.12	78,5107284	2,13146803	0,52224766	2,10E-06	0,00010308	UP	Kcnip3	2:127456498-127522094	protein_coding	-
ENSMUSG00000034271.15	131,861411	1,71717724	0,42101869	2,35E-06	0,00011336	UP	Jdp2	12:85599105-85639878	protein_coding	+
ENSMUSG00000048154.16	7712,85922	0,86652651	0,20398098	2,50E-06	0,00011818	UP	Kmt2d	15:98831669-98871204	protein_coding	-
ENSMUSG00000096054.2	1137,14946	1,00480177	0,24103846	2,56E-06	0,00012051	UP	Syne1	10:5020203-5550692	protein_coding	-
ENSMUSG00000071203.6	87,8748011	1,9867051	0,49147756	2,64E-06	0,00012411	UP	Naip5	13:100211739-100246323	protein_coding	-
ENSMUSG00000024011.16	63,7602119	2,48075505	0,63535393	2,89E-06	0,00013421	UP	Pi16	17:29318877-29329413	protein_coding	+
ENSMUSG00000032815.15	473,272142	1,13275494	0,27492095	2,91E-06	0,00013459	UP	Fanca	8:123268300-123318576	protein_coding	-
ENSMUSG00000019302.16	73,5604021	2,18568497	0,54705613	3,03E-06	0,00013933	UP	Atp6v0a1	11:101009452-101063719	protein_coding	+
ENSMUSG00000030852.16	108,211648	1,83954548	0,4607241	3,09E-06	0,00014168	UP	Tacc2	7:130577438-130764785	protein_coding	+
ENSMUSG00000035900.18	976,027603	1,00232336	0,24367219	3,26E-06	0,00014827	UP	Gramd4	15:86057695-86137634	protein_coding	+
ENSMUSG00000083950.1	29,5386812	3,67120761	0,92127139	3,53E-06	0,0001595	UP	Gm14466	2:79022821-79023271	processed_pseudogene	-
ENSMUSG00000056399.1	80,9376689	2,29588531	0,59136578	3,69E-06	0,0001664	UP	Prss34	17:25298394-25300161	protein_coding	+

ENSMUSG00000030263.13	1289,81216	0,94087031	0,22897436	3,74E-06	0,00016781	UP	Lrmp	6:145115653-145174934	protein_coding	+
ENSMUSG00000025558.15	644,70082	1,06005945	0,26026157	3,78E-06	0,00016925	UP	Dock9	14:121542039-121797734	protein_coding	-
ENSMUSG00000032690.16	126,471878	1,67966992	0,42253887	3,85E-06	0,0001716	UP	Oas2	5:120730333-120749853	protein_coding	-
ENSMUSG00000028456.18	35,3808753	3,14668852	0,81002785	3,86E-06	0,00017172	UP	Unc13b	4:43058953-43264871	protein_coding	+
ENSMUSG00000032021.13	99,481917	2,17162489	0,59116748	4,04E-06	0,00017875	UP	Crtam	9:40972753-41004628	protein_coding	-
ENSMUSG00000070291.4	41,040435	2,81682199	0,72225905	4,21E-06	0,00018564	UP	Ddx43	9:78395777-78423587	protein_coding	+
ENSMUSG00000052632.15	157,134378	1,56702511	0,3975118	4,35E-06	0,00019083	UP	Asap2	12:21111748-21270171	protein_coding	+
ENSMUSG00000038384.16	1009,67489	0,96672334	0,23720066	4,40E-06	0,00019211	UP	Setd1b	5:123142193-123168629	protein_coding	+
ENSMUSG00000001156.9	1354,50761	0,92912356	0,22800583	4,72E-06	0,00020501	UP	Mxd1	6:86647042-86669161	protein_coding	-
ENSMUSG00000005800.2	220,720233	1,40949226	0,35643991	4,77E-06	0,00020659	UP	Mmp8	9:7558429-7568486	protein_coding	+
ENSMUSG00000105504.4	613,795928	1,07738188	0,27241752	6,01E-06	0,00025682	UP	Gbp5	3:142493978-142522344	protein_coding	+
ENSMUSG00000025330.6	86,2842249	2,04093061	0,54461429	6,36E-06	0,00027012	UP	Padi4	4:140745865-140774236	protein_coding	-
ENSMUSG00000034312.13	808,154464	1,00494613	0,2535642	6,44E-06	0,0002727	UP	Iqsec1	6:90656088-90810123	protein_coding	-
ENSMUSG00000029561.17	185,932019	1,42461843	0,36846824	6,63E-06	0,00027948	UP	Oasl2	5:114896936-114912234	protein_coding	+
ENSMUSG00000023915.4	66,6622672	2,26858991	0,60401338	7,07E-06	0,00029639	UP	Tnfrsf21	17:43016555-43089188	protein_coding	+
ENSMUSG00000040613.14	202,013905	1,39325262	0,36358974	7,47E-06	0,00031022	UP	Apobec1	6:122577792-122602444	protein_coding	-
ENSMUSG00000052534.15	93,4922292	1,88825698	0,50001028	8,06E-06	0,00033255	UP	Pbx1	1:168119364-168432270	protein_coding	-
ENSMUSG00000061175.11	111,608577	1,69337539	0,44964474	8,48E-06	0,00034782	UP	Fnip2	3:79455974-79567796	protein_coding	-
ENSMUSG00000009739.16	366,063136	1,24693481	0,33042905	8,72E-06	0,0003563	UP	Pou6f1	15:100575318-100599984	protein_coding	-
ENSMUSG00000053101.2	163,638733	1,533414	0,4058313	9,09E-06	0,00037044	UP	Gpr141	13:19749682-19824257	protein_coding	-
ENSMUSG00000058881.12	98,5230861	1,84609203	0,49724619	9,35E-06	0,00038001	UP	Zfp516	18:82910879-83005314	protein_coding	+
ENSMUSG00000049577.14	844,799657	0,95612328	0,24485099	9,44E-06	0,00038221	UP	Zfpm1	8:122282141-122337251	protein_coding	+
ENSMUSG00000024247.14	63,5722251	2,17545711	0,59628395	9,90E-06	0,00039871	UP	Pkdcc	17:83215292-83225070	protein_coding	+
ENSMUSG00000022637.10	1993,58203	0,98458208	0,25866303	1,01E-05	0,00040627	UP	Cblb	16:52031549-52208047	protein_coding	+
ENSMUSG00000022788.16	100,951343	1,76239058	0,47449692	1,05E-05	0,0004216	UP	Fgd4	16:16416917-16600549	protein_coding	-
ENSMUSG00000030214.6	183,94583	1,42817383	0,38185336	1,11E-05	0,00044157	UP	Plbd1	6:136612070-136661928	protein_coding	-
ENSMUSG00000074272.10	120,937152	1,64358217	0,44471925	1,17E-05	0,00046379	UP	Ceacam1	7:25461707-25477603	protein_coding	-
ENSMUSG00000028644.16	95,9268759	1,97706356	0,56022327	1,23E-05	0,00048108	UP	Ermap	4:119175457-119190011	protein_coding	-
ENSMUSG00000025375.15	41,038717	2,73264024	0,75463669	1,24E-05	0,00048547	UP	Aatk	11:120007313-120047167	protein_coding	-
ENSMUSG00000018899.16	4599,77021	0,81047729	0,20946033	1,26E-05	0,00049006	UP	Irf1	11:53770014-53778374	protein_coding	+
ENSMUSG00000031822.18	982,240241	0,91595983	0,23874504	1,27E-05	0,00049067	UP	Gse1	8:120230536-120581390	protein_coding	+
ENSMUSG00000027360.5	152,373204	1,48340184	0,40235347	1,31E-05	0,00050601	UP	Hdc	2:126593667-126619299	protein_coding	-
ENSMUSG00000025555.14	649,004668	1,0289771	0,27358893	1,31E-05	0,00050601	UP	Farp1	14:121035200-121283744	protein_coding	+
ENSMUSG00000029478.16	1161,56179	0,9259351	0,24516547	1,43E-05	0,000546	UP	Ncor2	5:125017153-125179219	protein_coding	-
ENSMUSG00000036499.8	1013,81725	1,10943989	0,30557289	1,51E-05	0,00057572	UP	Eea1	10:95940663-96045518	protein_coding	+
ENSMUSG00000026180.8	108,171462	1,74189305	0,48613794	1,60E-05	0,00060276	UP	Cxcr2	1:74153989-74161246	protein_coding	+
ENSMUSG00000068587.9	92,4284009	1,82152288	0,50876175	1,70E-05	0,00063168	UP	Mgam	6:40628831-40769123	protein_coding	+
ENSMUSG00000040314.1	118,343741	1,80803734	0,50748619	1,77E-05	0,00065321	UP	Ctsg	14:56099881-56102574	protein_coding	-
ENSMUSG00000063450.14	926,685422	0,90390328	0,24018394	1,77E-05	0,00065425	UP	Syne2	12:75818134-76110926	protein_coding	+
ENSMUSG00000026532.7	69,3836471	2,05424503	0,58693937	1,87E-05	0,00068417	UP	Spta1	1:174172776-174248450	protein_coding	+
ENSMUSG00000056413.16	807,051288	0,93009902	0,24964604	1,88E-05	0,00068576	UP	Adap1	5:139271876-139325622	protein_coding	-
ENSMUSG00000042228.14	258,271745	1,24051485	0,34375857	1,99E-05	0,00071891	UP	Lyn	4:3678115-3813122	protein_coding	+
ENSMUSG00000038884.14	188,785789	1,30997924	0,36364314	2,03E-05	0,00072984	UP	A230050P20I	9:20868642-20874307	protein_coding	+
ENSMUSG00000089809.8	26,90517	3,68109987	1,10913545	2,08E-05	0,00074513	UP	A930011G23	5:99297244-99729065	protein_coding	-
ENSMUSG00000023913.17	149,115696	1,50074495	0,42315894	2,12E-05	0,00076035	UP	Pla2g7	17:43568098-43612201	protein_coding	+
ENSMUSG00000041515.9	617,544401	1,04654588	0,28972179	2,18E-05	0,00077653	UP	Irf8	8:120736358-120756694	protein_coding	+
ENSMUSG00000047139.8	325,193994	1,14664952	0,31892285	2,36E-05	0,0008329	UP	Cd24a	10:43579169-43584262	protein_coding	+
ENSMUSG00000070327.14	5211,08654	0,78404648	0,20959941	2,36E-05	0,0008329	UP	Rnf213	11:119393100-119487418	protein_coding	+
ENSMUSG00000064246.10	120,018344	1,63928543	0,46822066	2,44E-05	0,00085835	UP	Chil1	1:134182176-134190181	protein_coding	+
ENSMUSG00000038058.14	1197,50561	0,86837994	0,23275105	2,46E-05	0,00086104	UP	Nod1	6:54923949-54972612	protein_coding	-
ENSMUSG00000089525.1	28,7179499	3,02020171	0,88789918	2,80E-05	0,00097262	UP	Gm23833	17:12770631-12770882	misc_RNA	+
ENSMUSG00000033350.7	584,039806	0,96082924	0,26606488	2,83E-05	0,0009795	UP	Chst2	9:95399292-95406722	protein_coding	-
ENSMUSG00000024812.9	334,485428	1,13218589	0,32104417	2,85E-05	0,00098281	UP	Tjp2	19:24094523-24225026	protein_coding	-
ENSMUSG00000028245.15	3811,96799	0,78888422	0,21443082	2,89E-05	0,00099027	UP	Nsmaf	4:6396207-6454271	protein_coding	-
ENSMUSG00000056501.3	211,72787	1,23842094	0,35336172	3,00E-05	0,00102746	UP	Cebpb	2:167688915-167690418	protein_coding	+
ENSMUSG00000045094.7	33,6303883	2,85724094	0,8743268	3,07E-05	0,00104623	UP	Arhgef37	18:61493794-61536536	protein_coding	-
ENSMUSG00000062044.14	41,3826386	2,43858003	0,73159446	3,13E-05	0,00106648	UP	Lmtk3	7:45783947-45804140	protein_coding	+
ENSMUSG00000038463.8	97,6453836	1,77976346	0,52260235	3,29E-05	0,00110906	UP	Olfml2b	1:170644532-170682789	protein_coding	+
ENSMUSG00000032198.8	66,1665777	1,92408366	0,56833633	3,34E-05	0,00111908	UP	Dock6	9:21800184-21852635	protein_coding	-
ENSMUSG00000102151.1	319,275585	1,15631745	0,33368526	3,40E-05	0,00113241	UP	Gm37472	14:61677443-61681494	TEC	-
ENSMUSG00000032841.15	88,7960298	1,68768212	0,49726626	3,44E-05	0,00113831	UP	Prr5l	2:101714285-101883027	protein_coding	-
ENSMUSG00000020422.13	121,936522	1,48524046	0,43661717	3,70E-05	0,00121752	UP	Tns3	11:8431652-8664535	protein_coding	-
ENSMUSG00000035283.4	24,3795176	3,25889847	0,97865698	3,79E-05	0,00123861	UP	Adrb1	19:56722372-56724862	protein_coding	+
ENSMUSG00000030761.15	72,0057207	1,82912039	0,54608277	3,78E-05	0,00123861	UP	Myo7a	7:98051060-98119524	protein_coding	-
ENSMUSG00000044320.14	36,6496363	2,57627981	0,77574083	3,84E-05	0,00125154	UP	1700001O22I	2:30794769-30803661	protein_coding	-
ENSMUSG00000041481.16	2972,72995	0,85714628	0,24178608	3,88E-05	0,00125986	UP	Serpina3g	12:104236245-104241939	protein_coding	+
ENSMUSG00000035735.10	20,8992917	3,70635408	1,13694861	3,93E-05	0,00126406	UP	Dagla	19:10245265-10304877	protein_coding	-
ENSMUSG00000004864.12	63,4633974	2,11192321	0,64305197	3,91E-05	0,00126406	UP	Mapk13	17:28769307-28778698	protein_coding	+
ENSMUSG00000046245.13	81,8841118	1,78441799	0,53348011	3,94E-05	0,00126406	UP	Pilra	5:137821952-137836281	protein_coding	-
ENSMUSG00000103869.1	221,528922	1,25125031	0,36977393	3,97E-05	0,00126883	UP	Gm37420	14:61667220-61670131	TEC	-
ENSMUSG00000025409.14	299,341871	1,09301096	0,31514762	3,99E-05	0,00127402	UP	Mbd6	10:127281956-127289018	protein_coding	-
ENSMUSG00000030653.16	562,088479	0,99895805	0,28902827	4,03E-05	0,0012841	UP	Pde2a	7:101421691-101512829	protein_coding	+
ENSMUSG00000025427.14	21,9304185	3,82620756	1,3067771	4,23E-05	0,00134007	UP	Rnf165	18:77456110-77565147	protein_coding	-
ENSMUSG00000034438.16	996,952714	0,8630577	0,24452015	4,41E-05	0,00138274	UP	Gbp8	5:105014150-105139540	protein_coding	-
ENSMUSG00000015981.12	538,511837	0,95773633	0,27615984	4,63E-05	0,00144742	UP	Stk32c	7:139103638-139213307	protein_coding	-
ENSMUSG00000030134.11	76,024705	1,8237997	0,56227046	4,90E-05	0,0015164	UP	Rasgef1a	6:118011438-118091546	protein_coding	+
ENSMUSG00000021322.7	68,9839521	1,89475329	0,58058908	4,98E-05	0,00153476	UP	Aoah	13:20794119-21024252	protein_coding	+
ENSMUSG00000040276.14	130,629703	1,44313502	0,4385795	5,04E-05	0,00154627	UP	Pacsin1	17:27655509-27711106	protein_coding	+
ENSMUSG00000023473.11	37,7058677	2,59383199	0,80901038	5,30E-05	0,00161408	UP	Celsr3	9:108826320-108852969	protein_coding	+
ENSMUSG00000020846.6	271,732562	1,13500745	0,33619502	5,42E-05	0,00164132	UP	Fam101b	11:76019194-76027782	protein_coding	-
ENSMUSG00000030339.7	56,1287228	2,14172711	0,67449814	5,58E-05	0,00168001	UP	Ltbr	6:125306571-125313885	protein_coding	-
ENSMUSG00000056054.9	4949,13729	0,74185335	0,21010881	5,71E-05	0,00171056	UP	S100a8	3:90668978-90670035	protein_coding	+
ENSMUSG00000022651.5	156,979633	1,38885346	0,42162441	5,76E-05	0,00171876	UP	Retnlg	16:48872608-48874498	protein_coding	+
ENSMUSG00000029484.12	89,0417797	1,73640031	0,53836036	6,07E-05	0,00179317	UP	Anxa3	5:96793339-96845966	protein_coding	+

ENSMUSG00000026579.8	105,366469	1,55749123	0,48304708	6,42E-05	0,00188108	UP	F5	1:164151838-164220277	protein_coding	+
ENSMUSG00000062866.15	453,564257	1,09223155	0,33506482	6,41E-05	0,00188108	UP	Phactr2	10:13207717-13474396	protein_coding	-
ENSMUSG00000018930.3	188,112865	1,55379372	0,53721773	6,82E-05	0,00196425	UP	Ccl4	11:83662584-83664683	protein_coding	+
ENSMUSG00000022636.13	356,205687	1,0691002	0,32725331	7,33E-05	0,00210598	UP	Alcam	16:52248996-52454074	protein_coding	-
ENSMUSG00000059108.3	413,267954	1,02516706	0,30987718	7,58E-05	0,00216959	UP	Ifitm6	7:141015812-141016892	protein_coding	-
ENSMUSG00000061533.15	450,806093	0,93274378	0,27860619	7,59E-05	0,00216959	UP	Cep128	12:90998492-91384409	protein_coding	-
ENSMUSG00000026928.14	84,7576932	1,62851459	0,5149301	7,78E-05	0,00221156	UP	Card9	2:26352176-26360918	protein_coding	-
ENSMUSG00000027669.14	197,659579	1,21617442	0,38031974	8,02E-05	0,00226538	UP	Gnb4	3:32580332-32616585	protein_coding	-
ENSMUSG00000033777.3	92,7967953	1,63168752	0,51786898	8,12E-05	0,0022846	UP	Tlr13	X:106143204-106160493	protein_coding	+
ENSMUSG00000045078.12	551,645035	0,89192444	0,26678659	8,28E-05	0,00232414	UP	Rnf216	5:142990893-143112994	protein_coding	-
ENSMUSG00000027506.15	117,607917	1,40305568	0,44579131	9,16E-05	0,00256537	UP	Tpd52	3:8925593-9004723	protein_coding	-
ENSMUSG00000102051.1	164,033991	1,3214124	0,41825069	9,24E-05	0,00258082	UP	I830127L07R	15:75131377-75135128	transcribed_unprocesse	-
ENSMUSG00000053846.4	111,126295	1,5101722	0,48167752	9,65E-05	0,00266691	UP	Lipg	18:74939322-74961263	protein_coding	-
ENSMUSG00000042129.8	143,42255	1,30684246	0,41750483	9,79E-05	0,00270073	UP	Rassf4	6:116633008-116673952	protein_coding	-
ENSMUSG00000025429.8	38,5831095	2,31831261	0,76787966	0,00010015	0,00275664	UP	Pstpip2	18:77794545-77882007	protein_coding	+
ENSMUSG00000030786.18	117,135505	1,45529339	0,47661397	0,00011031	0,00301069	UP	Itgam	7:128062640-128118491	protein_coding	+
ENSMUSG00000047250.13	79,5706492	1,66138171	0,54701278	0,00011422	0,00308727	UP	Ptgs1	2:36230426-36252272	protein_coding	+
ENSMUSG00000026880.11	159,263242	1,26538138	0,40715615	0,00011508	0,00309916	UP	Stom	2:35313986-35336976	protein_coding	-
ENSMUSG00000020642.12	163,184417	1,2305509	0,39532176	0,00011513	0,00309916	UP	Rnf144a	12:26306797-26415256	protein_coding	-
ENSMUSG00000058794.12	97,185562	1,53940673	0,504352	0,00011984	0,00321946	UP	Nfe2	15:103248212-103258403	protein_coding	-
ENSMUSG00000004655.5	85,334562	1,62301129	0,5411553	0,00012054	0,00322524	UP	Aqp1	6:55336432-55348555	protein_coding	+
ENSMUSG00000019843.14	4416,09093	0,69524259	0,20735768	0,00012032	0,00322524	UP	Fyn	10:39368855-39565381	protein_coding	+
ENSMUSG00000033147.16	527,181029	0,92488143	0,29018644	0,00012289	0,00327907	UP	Slc22a15	3:101855776-101924453	protein_coding	-
ENSMUSG00000081769.8	301,757818	1,0226589	0,32415626	0,0001239	0,00329517	UP	Gm12216	11:53783418-53859256	protein_coding	-
ENSMUSG00000055013.14	78,588558	1,6080591	0,53453905	0,00012769	0,00336201	UP	Agap1	1:89454806-89897617	protein_coding	+
ENSMUSG00000074604.9	126,607276	1,33926832	0,44181791	0,00012874	0,00338294	UP	Mgst2	3:51660360-51682677	protein_coding	+
ENSMUSG00000033705.16	88,7133967	1,51873633	0,50456783	0,00013276	0,00347753	UP	Stard9	2:120629126-120731895	protein_coding	+
ENSMUSG00000038872.9	51,9136295	1,91816048	0,65102413	0,00013423	0,00350627	UP	Zfhx3	8:108703100-108961630	protein_coding	+
ENSMUSG00000042333.14	448,147993	0,90976465	0,28637772	0,00013666	0,00356291	UP	Tnfrsf14	4:154922210-154928563	protein_coding	-
ENSMUSG00000073418.4	89,0807844	1,57265299	0,52704826	0,00013881	0,00360568	UP	C4b	17:34728380-34743882	protein_coding	-
ENSMUSG00000004446.12	176,266746	1,17708286	0,3844236	0,00013885	0,00360568	UP	Bid	6:120891930-120916853	protein_coding	-
ENSMUSG00000049804.9	38,3041609	2,19481842	0,75331044	0,00014104	0,00365556	UP	Armcx4	X:134686519-134696757	protein_coding	+
ENSMUSG00000097636.7	448,363627	0,93153602	0,29777593	0,00014816	0,00381768	UP	Mirt1	19:53441212-53464796	lincRNA	-
ENSMUSG00000005686.16	91,1193363	1,4986388	0,50386686	0,00014883	0,00382747	UP	Ampd3	7:110768206-110812405	protein_coding	+
ENSMUSG00000031444.16	63,2654128	1,83036192	0,62370285	0,00014991	0,00384774	UP	F10	8:13037308-13056676	protein_coding	+
ENSMUSG00000030748.9	768,337196	0,80660217	0,25030501	0,00015135	0,00387703	UP	Il4ra	7:125552120-125579474	protein_coding	+
ENSMUSG00000020486.18	23,1315827	2,84091008	0,99139003	0,00015506	0,0039568	UP	Sep 04	11:87568903-87590539	protein_coding	+
ENSMUSG00000026835.15	93,7129293	1,54350762	0,52348155	0,00015923	0,00404767	UP	Fcnb	2:28076378-28084885	protein_coding	-
ENSMUSG00000041143.16	631,052442	0,82378949	0,25851198	0,00015971	0,00405215	UP	Tmco4	4:138972888-139059171	protein_coding	+
ENSMUSG00000026893.4	71,7524964	1,71148838	0,60284475	0,00016359	0,00414257	UP	Gca	2:62664285-62694109	protein_coding	+
ENSMUSG00000022500.14	427,102513	0,93134761	0,30285716	0,00017296	0,00436309	UP	Litaf	16:10959275-11066157	protein_coding	-
ENSMUSG00000029915.14	90,2681727	1,57537423	0,54209777	0,00017371	0,0043656	UP	Clec5a	6:40574894-40585821	protein_coding	-
ENSMUSG00000034275.17	133,945064	1,26910415	0,42673676	0,00017447	0,00437641	UP	Igsf9b	9:27299228-27334763	protein_coding	+
ENSMUSG00000033400.14	115,412426	1,37141795	0,47327822	0,00018249	0,00456874	UP	Agl	3:116739999-116808166	protein_coding	-
ENSMUSG00000080984.1	15,8900005	4,05774331	1,43068441	0,0001832	0,0045779	UP	Gm14469	2:79038533-79038734	processed_pseudogene	-
ENSMUSG00000020042.15	417,078098	0,91017519	0,29550494	0,00018725	0,00466156	UP	Btbd11	10:85386814-85660292	protein_coding	+
ENSMUSG00000021108.17	2602,64424	0,69854927	0,21803749	0,00019106	0,00473865	UP	Prkch	12:73584797-73778185	protein_coding	+
ENSMUSG00000069873.4	68,8812123	1,78140565	0,62347787	0,00019272	0,00477084	UP	4930438A08I	11:58274799-58294289	protein_coding	+
ENSMUSG00000056394.17	837,962925	0,7715462	0,24419214	0,00019614	0,00481934	UP	Lig1	7:13277283-13311433	protein_coding	+
ENSMUSG00000031441.15	1005,92298	0,82024425	0,26195935	0,00019655	0,00482062	UP	Atp11a	8:12757014-12868728	protein_coding	+
ENSMUSG00000075122.4	201,567695	1,23862633	0,44073093	0,0002053	0,00501668	UP	Cd80	16:38458933-38486933	protein_coding	+
ENSMUSG00000025017.9	326,764937	0,97407416	0,32405694	0,00020674	0,00503329	UP	Pik3ap1	19:41274218-41385070	protein_coding	-
ENSMUSG00000025648.17	91,8432913	1,44301436	0,50644907	0,0002242	0,00539876	UP	Pfkfb4	9:108991778-109032228	protein_coding	+
ENSMUSG00000096751.3	403,503923	1,01359789	0,3481985	0,00022583	0,00539938	UP	Gm28373	12:102673017-102727333	processed_transcript	-
ENSMUSG00000108129.2	37,5467651	2,23206559	0,89518219	0,00022926	0,00547095	UP	4930417O13I	6:125265476-125287286	processed_transcript	+
ENSMUSG00000026923.15	4376,62872	0,65964512	0,20773741	0,00023641	0,00563123	UP	Notch1	2:26457903-26516663	protein_coding	-
ENSMUSG00000055782.8	86,3928019	1,5680361	0,57294063	0,0002698	0,00632431	UP	Abcd2	15:91145884-91191807	protein_coding	-
ENSMUSG00000045817.8	2651,14155	0,74679379	0,24530407	0,00027731	0,0064661	UP	Zfp36l2	17:84183931-84187947	protein_coding	-
ENSMUSG00000034177.15	567,135084	0,80880684	0,26869412	0,00028059	0,00653091	UP	Rnf43	11:87662722-87735539	protein_coding	+
ENSMUSG00000059498.13	137,72512	1,21331346	0,43062362	0,00028769	0,00667288	UP	Fcgr3	1:171051174-171064935	protein_coding	-
ENSMUSG00000040761.16	1570,81622	0,69610383	0,22389476	0,00028769	0,00667288	UP	Spen	4:141467890-141538597	protein_coding	-
ENSMUSG00000078122.4	194,607838	1,14595228	0,40987722	0,00029508	0,00678478	UP	F630028O10I	X:96239926-96243636	antisense	+
ENSMUSG00000056091.12	53,8317006	1,73026065	0,64113449	0,00029748	0,00681636	UP	St3gal5	6:72097592-72154571	protein_coding	+
ENSMUSG00000027797.15	38,2910092	2,48681214	1,00186672	0,00030065	0,00687708	UP	Dclk1	3:55242364-55539068	protein_coding	+
ENSMUSG00000041831.15	2141,57253	0,67044831	0,21679826	0,0003026	0,00690982	UP	Sytl3	17:6673458-6738042	protein_coding	+
ENSMUSG00000060568.14	31,2400615	2,67316581	1,12513631	0,00030541	0,00696178	UP	Fam78b	1:167001417-167091302	protein_coding	+
ENSMUSG00000107476.2	45,690148	1,83777197	0,69040481	0,00031998	0,00724414	UP	Zfp862-ps	6:48504337-48534832	transcribed_unitary_pse	+
ENSMUSG00000030148.15	73,4709138	1,64309237	0,6225127	0,00032304	0,00730086	UP	Clec4a2	6:123106428-123143999	protein_coding	+
ENSMUSG00000051748.2	158,91996	1,23396198	0,44586917	0,00033266	0,00746745	UP	Wfdc21	11:83746940-83752642	protein_coding	+
ENSMUSG00000000489.6	221,941838	0,99565101	0,34862277	0,00033648	0,00752761	UP	Pdgfb	15:79995900-80014808	protein_coding	-
ENSMUSG00000049807.16	62,4293765	1,59946907	0,59911551	0,00033764	0,00754074	UP	Arhgap23	11:97415533-97502402	protein_coding	+
ENSMUSG00000029086.15	66,2295545	1,66297306	0,62355713	0,0003409	0,00760088	UP	Prom1	5:43993620-44102032	protein_coding	-
ENSMUSG00000031264.13	46,3901933	1,91619581	0,72911761	0,00034841	0,00773037	UP	Btk	X:134542336-134583570	protein_coding	-
ENSMUSG00000064043.13	2627,01283	0,66474844	0,21785579	0,00035181	0,00779157	UP	Trerf1	17:47140875-47361958	processed_transcript	+
ENSMUSG00000021756.12	421,874922	0,86494379	0,29934899	0,00036094	0,00796722	UP	Il6st	13:112464070-112510086	protein_coding	+
ENSMUSG00000052234.2	11,3469337	4,9509728	2,04289917	0,00036216	0,00798082	UP	Epx	11:87864000-87875536	protein_coding	-
ENSMUSG00000040274.11	4455,60977	0,64139061	0,20994175	0,00036456	0,00802026	UP	Cdk6	5:3341485-3531008	protein_coding	+
ENSMUSG00000026177.11	49,4266526	1,80647764	0,69533375	0,00036829	0,00808894	UP	Slc11a1	1:74375195-74386062	protein_coding	+
ENSMUSG00000036882.7	137,9707	1,14257725	0,41565036	0,00038208	0,00836136	UP	Arhgap33	7:30522226-30535060	protein_coding	-
ENSMUSG00000024187.14	225,84735	0,97139136	0,34424126	0,00038293	0,00836136	UP	Fam234a	17:26211822-26244223	protein_coding	-
ENSMUSG00000039477.16	1394,15571	0,70046854	0,23340486	0,00038628	0,00838669	UP	Tnrc18	5:142724661-142817662	protein_coding	-
ENSMUSG00000021375.9	51,6735408	1,74445544	0,67343917	0,00039447	0,00853659	UP	Kif13a	13:46749087-46929718	protein_coding	-
ENSMUSG00000027864.9	95,3639865	1,36729796	0,52339444	0,00039398	0,00853659	UP	Ptgfrn	3:101040232-101110278	protein_coding	-
ENSMUSG00000031428.11	78,3503487	1,42263724	0,53705778	0,00039711	0,00857971	UP	Zcchc18	X:136993155-136998472	protein_coding	+

ENSMUSG00000003484.4	35,8659961	2,1039017	0,82243416	0,00039838	0,00859306	UP	Cyp4f18	8:71988482-72009626	protein_coding	-
ENSMUSG00000063810.6	484,040922	0,80292628	0,27685446	0,00041036	0,00883711	UP	Alms1	6:85587531-85702753	protein_coding	+
ENSMUSG00000042476.12	48,6464419	1,9946771	0,88554445	0,00042962	0,00919211	UP	Abcb4	5:8893717-8959231	protein_coding	+
ENSMUSG00000034730.16	16,1613023	3,15683089	1,29314021	0,00043575	0,00930827	UP	Adgrb1	15:74516195-74589465	protein_coding	+
ENSMUSG00000018334.17	1488,37493	0,66699398	0,2248837	0,00044135	0,00938239	UP	Ksr1	11:79013440-79146407	protein_coding	-
ENSMUSG00000016028.9	2233,69914	0,67051103	0,2229429	0,00044134	0,00938239	UP	Celsr1	15:85898758-86033777	protein_coding	-
ENSMUSG00000024769.7	137,364324	1,14523212	0,42629111	0,00044309	0,00940429	UP	Cdc42bpg	19:6306456-6325652	protein_coding	+
ENSMUSG00000024393.14	3998,94255	0,6290783	0,20787496	0,00044661	0,00946393	UP	Prrc2a	17:35149076-35164897	protein_coding	-
ENSMUSG00000017861.11	325,79063	0,89255016	0,32020635	0,000462	0,00974317	UP	Mybl2	2:163054687-163084688	protein_coding	+
ENSMUSG00000030579.10	192,967029	1,05521736	0,39003189	0,00046352	0,00975972	UP	Tyrobp	7:30413760-30417585	protein_coding	+
ENSMUSG00000030747.5	43,6574404	1,93376207	0,76560051	0,00046808	0,00982445	UP	Dgat2	7:99153658-99182719	protein_coding	-
ENSMUSG00000104475.1	40,4054731	1,83955572	0,73953387	0,00049635	0,01025523	UP	D630036G22	1:34236596-34238820	TEC	+
ENSMUSG00000037706.17	138,142103	1,27631414	0,52363279	0,00049591	0,01025523	UP	Cd81	7:143052739-143067934	protein_coding	+
ENSMUSG00000034522.9	69,382407	1,45039875	0,57005958	0,00051046	0,0104653	UP	Zfp395	14:65358676-65398930	protein_coding	+
ENSMUSG00000026581.14	450,931493	0,84746686	0,30582441	0,00051597	0,01056173	UP	Sell	1:164061982-164084181	protein_coding	+
ENSMUSG00000040479.11	4491,76995	0,65595859	0,22382544	0,00052259	0,01068088	UP	Dgkz	2:91932824-91975864	protein_coding	-
ENSMUSG00000103233.1	64,8293042	1,53666433	0,63100696	0,00052639	0,01074201	UP	Gm37159	2:68891366-68894970	TEC	+
ENSMUSG00000002204.9	202,993193	1,01196736	0,37983961	0,00053286	0,01082385	UP	Napsa	7:44572380-44586862	protein_coding	+
ENSMUSG00000033253.18	1674,05776	0,66370475	0,2271042	0,00053722	0,01088047	UP	Szt2	4:118362743-118409273	protein_coding	-
ENSMUSG00000011256.16	5032,97827	0,61316589	0,20662058	0,00053729	0,01088047	UP	Adam19	11:46055992-46147343	protein_coding	+
ENSMUSG00000004508.6	93,574878	1,32815375	0,52075682	0,00055499	0,01117075	UP	Gab2	7:97081586-97308946	protein_coding	+
ENSMUSG00000055639.16	42,144739	1,88345145	0,77075151	0,00055848	0,01119336	UP	Dach1	14:97786853-98169765	protein_coding	-
ENSMUSG00000046591.10	536,346453	0,76100165	0,26919529	0,00055865	0,01119336	UP	Ticrr	7:79660196-79698148	protein_coding	+
ENSMUSG00000042046.15	124,424459	1,14507547	0,43822554	0,00056205	0,01124443	UP	Dstyk	1:132417555-132466958	protein_coding	+
ENSMUSG00000037148.7	503,371328	0,77299635	0,27477134	0,00056352	0,01125671	UP	Arhgap10	8:77250366-77517907	protein_coding	-
ENSMUSG00000026158.11	125,625941	1,15897983	0,44841694	0,00056685	0,01130627	UP	Ogfrl1	1:23366424-23397772	protein_coding	-
ENSMUSG00000030474.9	52,066554	1,65027804	0,67323203	0,00058692	0,011654	UP	Siglece	7:43651070-43660161	protein_coding	-
ENSMUSG00000000555.6	44,4681975	1,79642013	0,74834295	0,0005941	0,01177883	UP	Itga5	15:103344289-103366763	protein_coding	-
ENSMUSG00000075028.11	154,800632	1,11230117	0,43718026	0,00060391	0,01193761	UP	Prdm11	2:92965151-93046167	protein_coding	-
ENSMUSG00000058290.3	665,403079	0,75400327	0,26931768	0,00060533	0,0119479	UP	Espl1	15:102296293-102324356	protein_coding	+
ENSMUSG00000028961.15	691,706463	0,79699326	0,29109943	0,0006171	0,01214387	UP	Pgd	4:149149991-149166771	protein_coding	-
ENSMUSG00000040957.14	33,2736726	1,9647297	0,8277679	0,00063358	0,0124091	UP	Cables1	18:11839220-11945627	protein_coding	+
ENSMUSG00000046574.7	686,112046	0,71717888	0,25481339	0,00064367	0,01257336	UP	Prr12	7:45027707-45052881	protein_coding	-
ENSMUSG00000052776.10	103,189612	1,23125063	0,49435111	0,00064939	0,0126479	UP	Oas1a	5:120896256-120907521	protein_coding	-
ENSMUSG00000042744.16	2513,72209	0,62234252	0,21367902	0,0006513	0,01265868	UP	Gm15800	5:121220219-121368577	protein_coding	+
ENSMUSG00000037410.13	1825,41581	0,63537358	0,21967432	0,00065713	0,01274234	UP	Tbc1d2b	9:90202027-90270804	protein_coding	-
ENSMUSG00000039021.15	56,5661552	1,53879381	0,64077741	0,00066503	0,01285788	UP	Ttc16	2:32757026-32775633	protein_coding	-
ENSMUSG00000026980.15	1191,32974	0,69729402	0,24901776	0,00068374	0,01318117	UP	Ly75	2:60292103-60383303	protein_coding	-
ENSMUSG00000008540.11	104,385959	1,28260586	0,52128853	0,00069118	0,01328593	UP	Mgst1	6:138140316-138156755	protein_coding	+
ENSMUSG00000047181.12	34,1148595	2,07338947	0,98880124	0,00069637	0,01336631	UP	Samd14	11:95009879-95026087	protein_coding	+
ENSMUSG00000039953.13	324,957705	0,84536034	0,31618763	0,00071398	0,0136646	UP	Clstn1	4:149586468-149648899	protein_coding	+
ENSMUSG00000029528.17	1253,91971	0,652074	0,22943757	0,000719	0,01374082	UP	Pxn	5:115506676-115555987	protein_coding	+
ENSMUSG00000039218.16	12338,8326	0,57973216	0,20135851	0,00072178	0,01377423	UP	Srrm2	17:23790662-23824741	protein_coding	+
ENSMUSG00000033767.14	1200,47339	0,65541544	0,23093776	0,00072688	0,01385157	UP	D930015E06I	3:83897655-84040175	protein_coding	-
ENSMUSG00000001763.14	42,7570345	1,87385228	0,85798732	0,00073344	0,01395646	UP	Tspan33	6:29694222-29718559	protein_coding	+
ENSMUSG00000075591.2	34,7941388	1,95881343	0,86677859	0,00074854	0,01420292	UP	Gm10874	5:138363719-138388287	lincRNA	+
ENSMUSG00000020432.12	82,7961102	1,32861333	0,55594164	0,00077673	0,01463306	UP	Tcn2	11:3917192-3932159	protein_coding	-
ENSMUSG00000013974.3	113,099121	1,19241598	0,49040468	0,00079104	0,01488149	UP	Mcemp1	8:3665754-3669259	protein_coding	+
ENSMUSG00000007080.14	997,380854	0,66340618	0,23723984	0,00079631	0,01495934	UP	Pole	5:110286306-110337474	protein_coding	+
ENSMUSG00000024885.8	62,0727317	1,48847014	0,62946016	0,00080619	0,01510214	UP	Aldh3b1	19:3913493-3929761	protein_coding	-
ENSMUSG00000045671.17	204,62563	0,94421768	0,37020932	0,0008195	0,01532972	UP	Spred2	11:19924375-20024026	protein_coding	+
ENSMUSG00000022126.6	41,0975622	1,75262019	0,76393043	0,00082211	0,0153353	UP	Irg1	14:103046977-103056573	protein_coding	+
ENSMUSG00000038252.13	2270,76546	0,62347183	0,21989244	0,00082539	0,01537477	UP	Ncapd2	6:125168007-125191701	protein_coding	-
ENSMUSG00000025702.15	49,6327257	1,68358788	0,76008378	0,00082722	0,01538532	UP	Mrz 08	6:116338024-116409540	protein_coding	+
ENSMUSG00000061414.7	363,167994	0,88439294	0,34760868	0,00082828	0,01538532	UP	Cracr2a	6:127561338-127630033	protein_coding	+
ENSMUSG00000042265.13	65,546138	1,47362784	0,62972431	0,00084799	0,01572636	UP	Trem1	17:48232768-48246924	protein_coding	+
ENSMUSG00000043740.14	131,670205	1,1293071	0,46942288	0,00084901	0,01572636	UP	B430306N03	17:48316141-48327024	protein_coding	+
ENSMUSG00000049985.14	15,4800272	2,9288793	1,37837518	0,00085979	0,01585946	UP	Ankrd55	13:112288451-112384002	protein_coding	+
ENSMUSG00000062991.7	15,7427476	3,15562796	1,45574656	0,00089407	0,01637764	UP	Nrg1	8:31814551-32884029	protein_coding	-
ENSMUSG00000097006.7	50,0965827	1,55042597	0,67808431	0,000897	0,01640865	UP	9530082P21F	17:23743234-23754065	lincRNA	+
ENSMUSG00000021596.16	65,8671218	1,4418655	0,62915693	0,00090536	0,01653874	UP	Mctp1	13:76384535-77031810	protein_coding	+
ENSMUSG00000022946.8	1199,47609	0,64285903	0,2319426	0,00090958	0,01659297	UP	Dopey2	16:93711907-93810585	protein_coding	+
ENSMUSG00000038644.14	705,102864	0,70006575	0,25878438	0,00091483	0,0166658	UP	Pold1	7:44532746-44548849	protein_coding	-
ENSMUSG00000020387.15	993,296698	0,65143038	0,23698491	0,00093007	0,01692008	UP	Jade2	11:51813455-51857653	protein_coding	-
ENSMUSG00000035835.14	21,0301276	2,65748136	1,29445312	0,00094156	0,01705878	UP	Plppr3	10:79860475-79874634	protein_coding	-
ENSMUSG00000027514.14	903,274326	0,6652924	0,24464112	0,00094862	0,01711656	UP	Zbp1	2:173206612-173218923	protein_coding	-
ENSMUSG00000025507.13	226,092497	0,88240864	0,34667247	0,00095804	0,01726053	UP	Pidd1	7:141438113-141444025	protein_coding	-
ENSMUSG00000023067.13	244,172873	0,90726232	0,36372982	0,00096465	0,01733491	UP	Cdkn1a	17:29090979-29100722	protein_coding	+
ENSMUSG00000030187.15	68,4871772	1,37716581	0,61031395	0,00101291	0,01813776	UP	Klra2	6:131219223-131247362	protein_coding	-
ENSMUSG00000038594.8	218,843978	0,88147025	0,34986842	0,00102912	0,0183688	UP	Cep85l	10:53278081-53379851	protein_coding	-
ENSMUSG00000040693.7	60,099754	1,48536853	0,66297561	0,00103743	0,01848289	UP	Slco4c1	1:96816270-96872171	protein_coding	-
ENSMUSG00000024681.11	94,3781931	1,31903905	0,57313454	0,0010383	0,01848289	UP	Ms4a3	19:11629496-11640851	protein_coding	-
ENSMUSG00000102662.1	68,9548529	1,43551291	0,7214112	0,00108214	0,01914327	UP	Gm38377	2:69018096-69021867	TEC	+
ENSMUSG00000031642.8	146,476006	0,99755224	0,41316901	0,00109833	0,01931771	UP	Sh3rf1	8:61224171-61396072	protein_coding	+
ENSMUSG00000034957.10	46,6138994	1,58430604	0,72540069	0,00110472	0,01940434	UP	Cebpa	7:35119293-35121928	protein_coding	+
ENSMUSG00000037129.7	129,143941	1,05679236	0,44780885	0,00111487	0,01955677	UP	Tmprss13	9:45319100-45347581	protein_coding	+
ENSMUSG00000050989.9	104,20706	1,11660432	0,47710855	0,00112331	0,01967877	UP	Sepn1	4:134537892-134552166	protein_coding	-
ENSMUSG00000043629.12	20,3447448	2,43019358	1,30362605	0,00113024	0,01977392	UP	1700019D03I	1:52922324-53020179	protein_coding	-
ENSMUSG00000019312.10	67,0109818	1,36936292	0,63379694	0,00113252	0,01978772	UP	Grb7	11:98446394-98455373	protein_coding	+
ENSMUSG00000022157.7	43,7270604	1,72496786	0,80713333	0,00115106	0,02003238	UP	Mcpt8	14:56082166-56085216	protein_coding	-
ENSMUSG00000040907.15	82,5476523	1,21781828	0,53455117	0,001168	0,02030052	UP	Atp1a3	7:24978167-25005958	protein_coding	-
ENSMUSG00000066952.11	40,2622056	1,65444154	0,77800903	0,00117225	0,020321	UP	Myo1h	5:114289166-114365357	protein_coding	+
ENSMUSG00000038578.15	59,5288263	1,43901205	0,66087902	0,0012074	0,02076729	UP	Susd1	4:59314683-59438633	protein_coding	-
ENSMUSG00000031805.17	2896,64185	0,5958215	0,22082912	0,00120737	0,02076729	UP	Jak3	8:71676296-71690575	protein_coding	+

ENSMUSG00000052435.6	65,215219	1,43189904	0,6606725	0,00121706	0,02085489	UP	Cebpe	14:54710363-54712174	protein_coding	-
ENSMUSG00000042064.13	142,004178	1,00098248	0,42397102	0,00121722	0,02085489	UP	Myo3b	2:70039126-70429198	protein_coding	+
ENSMUSG00000037020.16	227,927725	0,85278057	0,34532344	0,00123839	0,02110843	UP	Wdr62	7:30240138-30280419	protein_coding	-
ENSMUSG00000006307.16	2010,79677	0,60245353	0,22286548	0,00126519	0,0215099	UP	Kmt2b	7:30568858-30588726	protein_coding	-
ENSMUSG00000036469.16	97,9997084	1,19759693	0,5349112	0,00127179	0,02159432	UP	Mrz 01	8:65617900-66471637	protein_coding	+
ENSMUSG00000020647.9	762,253019	0,67176749	0,25857578	0,00127686	0,02165269	UP	Ncoa1	12:4247363-4477182	protein_coding	-
ENSMUSG00000030522.14	224,370737	0,86672546	0,35712739	0,00129044	0,02179922	UP	Mtmr10	7:64287653-64340806	protein_coding	+
ENSMUSG00000018476.7	676,967101	0,68510875	0,2648843	0,0012931	0,02181624	UP	Kdm6b	11:69398508-69413675	protein_coding	-
ENSMUSG00000028717.12	33,048388	1,83440381	0,91183065	0,00129647	0,02184542	UP	Tal1	4:115056426-115071755	protein_coding	+
ENSMUSG00000037552.17	1057,0984	0,63804829	0,24137997	0,00130337	0,02190584	UP	Plekhg2	7:28359604-28372599	protein_coding	-
ENSMUSG00000030000.10	38,4419118	1,76770108	0,9677713	0,00132	0,02215728	UP	Add2	6:86028681-86124409	protein_coding	+
ENSMUSG00000001995.8	92,7881989	1,13532775	0,50241061	0,00133079	0,0222819	UP	Sipa1l2	8:125418063-125492710	protein_coding	-
ENSMUSG00000069601.13	27,8052968	1,97818611	1,15591905	0,00133959	0,0223727	UP	Ank3	10:69533772-70027438	protein_coding	+
ENSMUSG00000025321.14	30,1374002	1,8512108	0,95328637	0,00133858	0,0223727	UP	Itgb8	12:119158022-119238802	protein_coding	-
ENSMUSG00000040964.16	33,571288	1,79460129	0,90468513	0,00134479	0,0223769	UP	Arhgef10l	4:140514485-140666012	protein_coding	-
ENSMUSG00000031877.8	42,4237047	1,70024924	0,84169502	0,00134816	0,0223769	UP	Ces2g	8:104961718-104969537	protein_coding	+
ENSMUSG00000028927.6	612,749357	0,77181967	0,31572521	0,00134713	0,0223769	UP	Padi2	4:140906344-140952586	protein_coding	+
ENSMUSG00000035311.16	1475,5767	0,59990481	0,22486021	0,00139804	0,02305813	UP	Gnptab	10:88379132-88447329	protein_coding	+
ENSMUSG00000096900.3	29,6275089	1,89005	1,06341415	0,00141294	0,02324598	UP	Trav9-1	14:53488045-53488567	TR_V_gene	+
ENSMUSG00000004085.14	54,3777018	1,4340986	0,68068967	0,00143434	0,0235688	UP	Zak	2:72285637-72442610	protein_coding	+
ENSMUSG00000038725.11	22,0825574	2,13462784	1,12046613	0,00144097	0,02364838	UP	Pkhd1l1	15:44457553-44597143	protein_coding	+
ENSMUSG00000022026.6	56,1231707	1,50991974	0,72267881	0,00145616	0,02386812	UP	Olfm4	14:80000302-80021930	protein_coding	+
ENSMUSG00000100354.1	16,2020288	2,49522266	1,31134213	0,00146227	0,02392109	UP	Gm29113	1:69490357-69493788	lincRNA	-
ENSMUSG00000025993.10	106,900388	1,08205769	0,48208	0,00146629	0,02392109	UP	Slc40a1	1:45908068-45926523	protein_coding	-
ENSMUSG00000063146.11	140,106059	0,9808646	0,42723469	0,00146748	0,02392109	UP	Clip2	5:134489383-134552434	protein_coding	-
ENSMUSG00000053113.3	793,658676	0,64539431	0,24854145	0,00147023	0,02392109	UP	Socs3	11:117966079-117970047	protein_coding	-
ENSMUSG00000043733.14	1726,73655	0,59439136	0,22360842	0,00146413	0,02392109	UP	Ptpn11	5:121130533-121191397	protein_coding	-
ENSMUSG00000042671.12	23,0255204	2,15976159	1,60494254	0,00148145	0,02407403	UP	Rgs8	1:153653025-153700323	protein_coding	+
ENSMUSG00000040524.9	242,569783	0,82712969	0,34469494	0,00149892	0,02432807	UP	Zfp609	9:65692391-65827564	protein_coding	-
ENSMUSG00000022861.16	29,2910955	1,89343307	0,9671548	0,00150631	0,02439065	UP	Dgkg	16:22468461-22657221	protein_coding	-
ENSMUSG00000039158.11	10244,2586	0,57893881	0,21823723	0,00150828	0,02439065	UP	Akna	4:63367125-63403354	protein_coding	-
ENSMUSG00000005534.10	244,378837	0,8321882	0,34808948	0,00153432	0,02469088	UP	Insr	8:3122061-3279617	protein_coding	-
ENSMUSG00000029674.13	586,990415	0,67108021	0,26315513	0,00153934	0,02471796	UP	Limk1	5:134656039-134688598	protein_coding	-
ENSMUSG00000073434.11	379,637986	0,72790475	0,29227848	0,00155402	0,0247969	UP	Wdr90	17:25844771-25861501	protein_coding	-
ENSMUSG00000027030.15	998,602782	0,63389759	0,24573985	0,00157591	0,02506079	UP	Stk39	2:68210445-68472268	protein_coding	-
ENSMUSG00000030302.16	17,7078418	2,70035015	1,39357828	0,00157979	0,02507121	UP	Atp2b2	6:113743831-114042613	protein_coding	-
ENSMUSG00000017057.9	47,4150995	1,54743244	0,76852278	0,00158238	0,02507121	UP	Il13ra1	X:36112110-36171259	protein_coding	+
ENSMUSG00000040624.17	46,0248075	1,5505141	0,76332685	0,00158601	0,02509579	UP	Plekhg1	10:3740364-3967303	protein_coding	+
ENSMUSG00000039262.16	8350,2401	0,55625668	0,20859784	0,00160049	0,02529459	UP	Prrc2b	2:32151082-32234537	protein_coding	+
ENSMUSG00000035640.18	53,3831426	1,38258077	0,67573181	0,00160623	0,02535503	UP	Cbarp	10:80130451-80140835	protein_coding	-
ENSMUSG00000030142.10	56,6724708	1,4129848	0,69485254	0,00164508	0,02581455	UP	Clec4e	6:123281789-123289870	protein_coding	-
ENSMUSG00000005696.11	533,64876	0,67289862	0,26719946	0,00164808	0,025831	UP	Sh2d1a	X:42502453-42529330	protein_coding	+
ENSMUSG00000019726.10	3692,26408	0,55448592	0,20854023	0,00165486	0,02590657	UP	Lyst	13:13590409-13777440	protein_coding	+
ENSMUSG00000104262.1	54,2106818	1,37864461	0,69431474	0,00166973	0,02610853	UP	Gm37747	2:68989381-68993383	TEC	+
ENSMUSG00000079293.11	65,4794905	1,29670431	0,62609717	0,00167327	0,02613306	UP	Clec7a	6:129461591-129472779	polymorphic_pseudogen	-
ENSMUSG00000055413.12	882,111141	0,65940002	0,26255101	0,00169649	0,02640227	UP	H2-Q5	17:35394126-35397800	polymorphic_pseudogen	+
ENSMUSG00000040026.7	77,0505699	1,24286908	0,59219123	0,0017286	0,0268075	UP	Saa3	7:46711998-46715676	protein_coding	-
ENSMUSG00000026335.16	52,0553378	1,41379341	0,71883996	0,00173364	0,02684168	UP	Pam	1:97795114-98095646	protein_coding	-
ENSMUSG00000044033.16	27,945883	1,79856373	0,94556643	0,00175704	0,02711401	UP	Ccdc141	2:77009902-77170636	protein_coding	-
ENSMUSG00000026879.14	269,526162	0,78854408	0,33159215	0,00177005	0,02725346	UP	Gsn	2:35256380-35307892	protein_coding	+
ENSMUSG00000038173.14	21,5657549	2,04837452	1,17648545	0,00178099	0,02736367	UP	Enpp6	8:46986925-47094895	protein_coding	+
ENSMUSG00000029490.3	17,2391324	2,43972832	1,36629905	0,00179547	0,02752223	UP	Mfsd7a	5:108441054-108448891	protein_coding	-
ENSMUSG00000037321.17	5342,809	0,55826349	0,21310274	0,00180021	0,02755755	UP	Tap1	17:34187553-34197225	protein_coding	+
ENSMUSG00000040584.8	425,974555	0,72344376	0,29915468	0,00181054	0,02765722	UP	Abcb1a	5:8660077-8748575	protein_coding	+
ENSMUSG00000029101.14	180,668344	0,86614263	0,37671162	0,00182095	0,02768852	UP	Rgs12	5:34949445-35039644	protein_coding	+
ENSMUSG00000027009.18	16577,656	0,56920413	0,2191186	0,00181951	0,02768852	UP	Itga4	2:79255426-79333123	protein_coding	+
ENSMUSG00000003032.8	62,6079271	1,29617057	0,63796625	0,00183792	0,02788454	UP	Klf4	4:55527143-55532466	protein_coding	-
ENSMUSG00000103546.1	21,0207593	2,04600792	1,16272579	0,00185467	0,02810446	UP	Gm37666	2:69013612-69014477	TEC	+
ENSMUSG00000058325.5	37,4155722	1,65116487	0,85912333	0,00186302	0,02816641	UP	Dock1	7:134670687-135173639	protein_coding	+
ENSMUSG00000049625.5	49,6688411	1,40038884	0,71534925	0,00186817	0,02818007	UP	Tifab	13:56173703-56178885	protein_coding	-
ENSMUSG00000042745.9	22,146544	2,217128	1,21142174	0,00188959	0,02840611	UP	Id1	2:152736251-152737410	protein_coding	+
ENSMUSG00000020057.2	62,0351415	1,27609886	0,6349765	0,00188669	0,02840611	UP	Dram1	10:88322804-88379080	protein_coding	-
ENSMUSG00000102189.1	62,3257291	1,39211137	0,912618	0,00189613	0,02847206	UP	Gm37194	14:50061360-50064390	TEC	+
ENSMUSG00000024222.16	2095,58927	0,56873261	0,22001502	0,00192358	0,02881885	UP	Fkbp5	17:28399095-28517524	protein_coding	-
ENSMUSG00000029554.15	450,451887	0,69613095	0,28557046	0,00192639	0,02882835	UP	Mad1l1	5:140008689-140321552	protein_coding	-
ENSMUSG00000038894.7	121,64852	1,00104396	0,46379595	0,00197029	0,02938566	UP	Irs2	8:10984681-11008458	protein_coding	-
ENSMUSG00000079020.9	310,61587	0,73962542	0,31024554	0,00196975	0,02938566	UP	Slc45a4	15:73577424-73645762	protein_coding	-
ENSMUSG00000028760.16	2177,17261	0,55719894	0,21496986	0,00198459	0,02953254	UP	Eif4g3	4:137993022-138208508	protein_coding	+
ENSMUSG00000018927.3	57,1332008	1,34388886	0,68439513	0,00199569	0,02961571	UP	Ccl6	11:83587882-83593087	protein_coding	-
ENSMUSG00000032812.16	1103,29553	0,61633827	0,24597296	0,00199689	0,02961571	UP	Arap1	7:101348067-101412586	protein_coding	+
ENSMUSG00000022965.7	58,5183042	1,30448394	0,65660863	0,00200607	0,0296853	UP	Ifngr2	16:91547072-91565169	protein_coding	+
ENSMUSG00000001517.14	720,003289	0,64427396	0,2571038	0,00201564	0,02979373	UP	Foxm1	6:128362967-128376146	protein_coding	+
ENSMUSG00000034023.16	330,315767	0,7261181	0,30641021	0,00202553	0,0299	UP	Fancd2	6:113531682-113597017	protein_coding	+
ENSMUSG00000037685.15	734,145585	0,64311589	0,26077921	0,00203641	0,03000026	UP	Atp8a1	5:67618140-67847434	protein_coding	-
ENSMUSG00000002835.8	470,780325	0,66999892	0,2752199	0,00208016	0,03061076	UP	Chaf1a	17:56040416-56068026	protein_coding	+
ENSMUSG00000063430.9	202,230122	0,8319552	0,36915019	0,00212209	0,0311585	UP	Wscd2	5:113490333-113589725	protein_coding	+
ENSMUSG00000041801.5	51,5285411	1,3379444	0,70555576	0,00215518	0,03157438	UP	Phlda3	1:135766119-135769136	protein_coding	+
ENSMUSG00000025270.13	35,3429477	1,67479485	0,98961399	0,00219169	0,0320737	UP	Alas2	X:150547375-150570638	protein_coding	+
ENSMUSG00000031497.9	64,7263946	1,20931968	0,61653411	0,0022014	0,03214488	UP	Tnfsf13b	8:10006467-10039072	protein_coding	+
ENSMUSG00000028496.17	1333,52844	0,69890689	0,30505222	0,00221511	0,03229922	UP	Mllt3	4:87769925-88033364	protein_coding	-
ENSMUSG00000038775.14	34,6872701	1,76663763	1,08696254	0,00222564	0,03235601	UP	Vill	9:119052778-119071525	protein_coding	+
ENSMUSG00000028602.12	37,8001519	1,51644276	0,88898752	0,00224784	0,03264301	UP	Tnfrsf8	4:145267137-145315164	protein_coding	-
ENSMUSG00000038151.12	822,304054	0,60473864	0,24386245	0,00225076	0,03264958	UP	Prdm1	10:44437177-44528501	protein_coding	-
ENSMUSG00000038473.14	23,254711	1,88575043	1,11746919	0,00226383	0,03276733	UP	Nos1ap	1:170302668-170589861	protein_coding	-

ENSMUSG00000025236.10	507,092718	0,66838036	0,27732985	0,00226713	0,03277933	UP	Adpgk	9:59291572-59316199	protein_coding	+
ENSMUSG00000029163.9	68,5529369	1,18089198	0,60146339	0,0022854	0,03293563	UP	Emilin1	5:30913402-30921277	protein_coding	+
ENSMUSG00000056515.8	110,02609	1,00422417	0,48518175	0,00229027	0,03296996	UP	Rab31	17:65651726-65772752	protein_coding	-
ENSMUSG00000056529.7	60,6494238	1,2499899	0,65032119	0,00231337	0,03313538	UP	Ptafr	4:132564067-132582683	protein_coding	+
ENSMUSG00000038264.7	271,463641	0,8019504	0,36205027	0,00231001	0,03313538	UP	Sema7a	9:57940113-57962864	protein_coding	+
ENSMUSG00000035929.11	4894,86468	0,62374395	0,25915628	0,00231928	0,03313538	UP	H2-Q4	17:35379617-35385290	protein_coding	+
ENSMUSG00000023827.8	247,077839	0,79160228	0,35267202	0,00232666	0,03320505	UP	Agpat4	17:12118704-12219645	protein_coding	+
ENSMUSG00000049999.4	28,4896958	1,82145853	1,03953959	0,00233225	0,03324897	UP	Ppp1r3d	2:178411206-178414472	protein_coding	-
ENSMUSG00000032691.14	48,4343275	1,37414319	0,74617858	0,00237183	0,03366825	UP	Nlrp3	11:59541568-59566956	protein_coding	+
ENSMUSG00000020474.11	250,601199	0,76652325	0,33636998	0,00237009	0,03366825	UP	Polm	11:5827860-5838016	protein_coding	-
ENSMUSG00000037855.15	33,9958783	1,5753361	0,90397604	0,00240558	0,03407439	UP	Zfp365	10:67886103-67912662	protein_coding	-
ENSMUSG00000061589.14	955,201035	0,58772959	0,23745389	0,00242145	0,03426244	UP	Dot1l	10:80755206-80795461	protein_coding	+
ENSMUSG00000028268.14	922,902621	0,58759312	0,23994452	0,00250268	0,03527213	UP	Gbp3	3:142560026-142573209	protein_coding	+
ENSMUSG00000033826.9	1382,96808	0,59934884	0,24743668	0,00251123	0,0353011	UP	Dnah8	17:30624354-30875264	protein_coding	+
ENSMUSG00000072214.6	80,208316	1,10488067	0,5628813	0,00253431	0,03555549	UP	Sep 05	16:18621811-18629938	protein_coding	-
ENSMUSG00000034707.6	300,942277	0,72751965	0,31908812	0,00257658	0,03592021	UP	Gns	10:121365090-121397245	protein_coding	+
ENSMUSG00000029925.13	32,8264509	1,56415964	0,9137054	0,00264938	0,03681886	UP	Tbxas1	6:38875404-39084585	protein_coding	+
ENSMUSG00000029716.13	19,5307432	1,90578397	2,10404891	0,00268412	0,03718461	UP	Tfr2	5:137569840-137587481	protein_coding	+
ENSMUSG00000035873.7	31,192129	1,59995805	0,94481714	0,00270478	0,03743165	UP	Pawr	10:108332189-108414391	protein_coding	+
ENSMUSG00000046169.9	111,564771	0,97038243	0,48645167	0,00275919	0,03814484	UP	Adamts6	13:104287873-104494763	protein_coding	+
ENSMUSG00000002365.9	118,383326	0,93080639	0,45600488	0,00278084	0,03840404	UP	Snx9	17:5841328-5931033	protein_coding	+
ENSMUSG00000092251.3	20,3153679	0,27833529	0,51644185	0,00279198	0,03842967	UP	Trav9n-1	14:53101801-53102265	TR_V_pseudogene	+
ENSMUSG00000029298.15	2161,72652	0,59001882	0,24717062	0,0027869	0,03842967	UP	Gbp9	5:105077630-105139539	protein_coding	-
ENSMUSG00000022568.16	768,448568	0,60442931	0,25251564	0,00281628	0,03865159	UP	Scrib	15:76047158-76069784	protein_coding	-
ENSMUSG00000037936.15	133,922845	0,9006343	0,44097012	0,00289499	0,03948633	UP	Scarb1	5:125277087-125341094	protein_coding	-
ENSMUSG00000027562.12	137,472636	0,92489949	0,45973523	0,00290826	0,03962658	UP	Car2	3:14886273-14900770	protein_coding	+
ENSMUSG00000028965.13	234,193562	0,85784361	0,4360849	0,00292607	0,03982828	UP	Tnfrsf9	4:150914562-150946102	protein_coding	+
ENSMUSG00000086425.7	34,1039477	1,49742374	0,90382353	0,00293917	0,03996549	UP	F730016J06R	2:28095477-28127731	lincRNA	+
ENSMUSG00000055725.11	61,6758691	1,16131678	0,63054514	0,00297328	0,04005018	UP	Paqr3	5:97082329-97111596	protein_coding	-
ENSMUSG00000090942.1	76,8491204	1,07388858	0,5696201	0,00296551	0,04005018	UP	F830016B08R	18:60293380-60303016	protein_coding	+
ENSMUSG00000039512.11	137,81564	0,87977292	0,4282059	0,00297564	0,04005018	UP	Uhrf1bp1	17:27856490-27900040	protein_coding	+
ENSMUSG00000053063.11	180,669827	0,84170189	0,40214581	0,00295714	0,04005018	UP	Clec12a	6:129342691-129365303	protein_coding	+
ENSMUSG00000073902.5	3288,99326	0,53012287	0,21387099	0,0029593	0,04005018	UP	Gm1966	7:106596743-106604035	unprocessed_pseudogen	-
ENSMUSG00000031834.15	86,3024565	1,02578343	0,53356411	0,0029999	0,04033577	UP	Pik3r2	8:70768176-70776713	protein_coding	-
ENSMUSG00000020120.15	2027,09014	0,53294368	0,21566778	0,00300379	0,04034711	UP	Plek	11:16971206-17052381	protein_coding	-
ENSMUSG00000056917.12	2897,1137	0,57583554	0,24144881	0,00302139	0,04054239	UP	Sipa1	19:5651185-5663707	protein_coding	-
ENSMUSG00000039046.15	283,717908	0,71150429	0,31902442	0,00304695	0,04084398	UP	Usp6nl	2:6322667-6446390	protein_coding	+
ENSMUSG00000107971.1	42,9867308	1,32675364	0,7674987	0,00305406	0,04089791	UP	Gm44260	6:120912900-120914993	TEC	-
ENSMUSG00000038708.9	1880,54843	0,55480913	0,22933603	0,00306592	0,04101523	UP	Golga4	9:118506318-118582522	protein_coding	+
ENSMUSG00000036718.17	17,8334359	2,19851312	1,63225771	0,00307392	0,04106932	UP	Micall2	5:139706696-139736336	protein_coding	-
ENSMUSG00000033720.12	39,6709721	1,369727	0,80836529	0,00307617	0,04106932	UP	Sfxn5	6:85213049-85333422	protein_coding	-
ENSMUSG00000052270.7	153,905765	0,91046215	0,46153063	0,00317081	0,04203623	UP	Fpr2	17:17887824-17893952	protein_coding	+
ENSMUSG00000025743.14	91,0411023	0,99666947	0,52159215	0,00325969	0,04304226	UP	Sdc3	4:130792537-130826319	protein_coding	+
ENSMUSG00000040152.8	45,2564851	1,36418594	0,82560561	0,00329448	0,04337187	UP	Thbs1	2:118111876-118127133	protein_coding	+
ENSMUSG00000038126.17	682,159173	0,59052593	0,25173004	0,00332561	0,04369485	UP	Mphosph9	5:124250959-124327972	protein_coding	-
ENSMUSG00000041598.7	50,6986043	1,23021457	0,72195902	0,00335612	0,04400839	UP	Cdc42ep4	11:113726850-113751881	protein_coding	-
ENSMUSG00000041936.18	116,405064	0,93056782	0,48429946	0,00339384	0,04437117	UP	Agrn	4:156165290-156197488	protein_coding	-
ENSMUSG00000029192.17	879,991153	0,62519966	0,2772254	0,00342557	0,04469781	UP	Tbc1d14	5:36490604-36593276	protein_coding	-
ENSMUSG00000037336.14	65,0857517	1,10627352	0,62184147	0,00345655	0,04503611	UP	Mfsd2b	12:4862440-4874359	protein_coding	-
ENSMUSG00000028494.12	179,133822	0,7989749	0,38655639	0,0034583	0,04503611	UP	Plin2	4:86648386-86670060	protein_coding	-
ENSMUSG00000062157.6	26,4644237	1,62805285	1,122271	0,00348576	0,04530465	UP	Ifnlr1	4:135686287-135708181	protein_coding	+
ENSMUSG00000041528.15	1172,16462	0,55035354	0,22925487	0,00350945	0,04543416	UP	Rnf123	9:108051534-108083346	protein_coding	-
ENSMUSG00000032216.14	231,903614	0,75833778	0,36294696	0,00351823	0,04550334	UP	Nedd4	9:72662346-72749852	protein_coding	+
ENSMUSG00000069919.7	36,385648	1,42832111	0,89802113	0,00356081	0,0459196	UP	Hba-a1	11:32283511-32284465	protein_coding	+
ENSMUSG00000040785.17	658,311661	0,58636155	0,25329756	0,00360279	0,04637054	UP	Ttc3	16:94370618-94469222	protein_coding	+
ENSMUSG00000008999.7	24,7661536	1,66915697	1,20834279	0,00362081	0,04651216	UP	Bmp7	2:172868012-172940321	protein_coding	-
ENSMUSG00000022558.15	646,041404	0,59055778	0,25603535	0,00364795	0,04677002	UP	Mroh1	15:76380261-76453038	protein_coding	+
ENSMUSG00000063952.15	507,918542	0,62725747	0,2787917	0,00368111	0,04714959	UP	Brpf3	17:28801090-28838546	protein_coding	+
ENSMUSG00000102594.1	17,2025376	1,59694288	3,05698727	0,0037271	0,04760044	UP	Gm38381	1:168348534-168350808	TEC	-
ENSMUSG00000050965.14	723,47223	0,58760416	0,25589065	0,00375119	0,04786195	UP	Prkca	11:107933387-108343928	protein_coding	-
ENSMUSG00000041594.17	122,975502	0,90247109	0,48404916	0,00384292	0,04889116	UP	Tmtc4	14:122918971-122984035	protein_coding	-
ENSMUSG00000051506.16	356,683512	0,65764231	0,30035699	0,0038929	0,049432	UP	Wdfy4	14:32959547-33185508	protein_coding	-
ENSMUSG00000016024.9	75,3872377	1,09510196	0,65940941	0,0039101	0,0495554	UP	Lbp	2:158306493-158332852	protein_coding	+
ENSMUSG00000033249.10	43,355112	1,25960967	0,78893276	0,00391861	0,04958255	UP	Hsf4	8:105269801-105275845	protein_coding	+
ENSMUSG00000027293.13	357,573167	0,65396684	0,29872062	0,00391973	0,04958255	UP	Ehd4	2:120089175-120154606	protein_coding	-
ENSMUSG00000051343.11	28,8922647	1,51490863	1,06173136	0,00394207	0,04981748	UP	Rab11fip5	6:85334962-85374634	protein_coding	-
ENSMUSG00000053965.10	565,629933	-4,8285688	0,33182271	4,56E-49	3,02E-45	DOWN	Pde5a	3:122728947-122859374	protein_coding	+
ENSMUSG00000025491.14	1150,98424	-3,8922414	0,26857482	7,29E-49	3,22E-45	DOWN	Ifitm1	7:140967221-140969825	protein_coding	+
ENSMUSG00000023367.14	589,685097	-5,1639627	0,36066743	1,15E-47	3,82E-44	DOWN	Tmem176a	6:48840919-48847071	protein_coding	+
ENSMUSG00000029810.15	516,414763	-4,7598929	0,35098631	4,67E-43	1,24E-39	DOWN	Tmem176b	6:48833818-48841496	protein_coding	-
ENSMUSG00000053702.16	1029,74563	-3,2681399	0,26384266	8,85E-37	1,95E-33	DOWN	Nebl	2:17343909-17731464	protein_coding	-
ENSMUSG00000049103.13	5875,92977	-2,5483714	0,20544114	1,42E-36	2,68E-33	DOWN	Ccr2	9:124101950-124113557	protein_coding	+
ENSMUSG00000043088.16	414,156111	-5,4786807	0,46897373	9,34E-33	1,37E-29	DOWN	Il17re	6:113458484-113470758	protein_coding	+
ENSMUSG00000026072.12	517,798636	-3,6944273	0,33317466	2,31E-30	3,06E-27	DOWN	Il1r1	1:40225080-40317257	protein_coding	+
ENSMUSG00000028150.14	352,850163	-4,4566829	0,43285334	1,67E-26	1,84E-23	DOWN	Rorc	3:94372794-94398276	protein_coding	+
ENSMUSG00000030283.7	398,470727	-3,5489818	0,3514649	1,48E-25	1,51E-22	DOWN	St8sia1	6:142821545-142964452	protein_coding	-
ENSMUSG00000048521.7	4267,2053	-2,4330134	0,25278355	1,23E-23	1,16E-20	DOWN	Cxcr6	9:123806477-123811754	protein_coding	+
ENSMUSG00000030167.15	537,547806	-2,8416254	0,2931163	1,38E-23	1,22E-20	DOWN	Klrc1	6:129666015-129678973	protein_coding	-
ENSMUSG00000023927.15	4718,25527	-2,0533254	0,21347333	3,14E-23	2,45E-20	DOWN	Satb1	17:51736187-51833290	protein_coding	-
ENSMUSG00000070407.5	614,982253	-2,6783853	0,28298444	1,34E-22	9,89E-20	DOWN	Hs3st3b1	11:63885792-63922290	protein_coding	-
ENSMUSG00000020183.11	1339,89806	-2,2646077	0,2410845	2,81E-22	1,86E-19	DOWN	Cpm	10:117629500-117687352	protein_coding	+
ENSMUSG00000005802.12	430,554163	-3,1722834	0,35018073	1,97E-21	1,18E-18	DOWN	Slc30a4	2:122681233-122702663	protein_coding	-
ENSMUSG00000033849.3	213,669606	-6,3826883	0,67921876	2,78E-21	1,60E-18	DOWN	B3galt2	1:143640664-143654614	protein_coding	+
ENSMUSG00000033213.16	345,484319	-3,3721853	0,40726428	1,37E-18	5,85E-16	DOWN	AA467197	2:122636986-122641191	protein_coding	+

ENSMUSG00000042284.9	313,830039	-3,3989499	0,41949066	5,35E-18	2,22E-15	DOWN	Itga1	13:114958079-115101964	protein_coding	-
ENSMUSG00000001270.8	304,478727	-2,7198609	0,33246757	1,43E-17	5,73E-15	DOWN	Ckb	12:111669355-111672338	protein_coding	-
ENSMUSG00000009588.9	778,918255	-1,9731282	0,25258333	3,34E-16	1,17E-13	DOWN	St6galnac1	11:116765025-116775507	protein_coding	-
ENSMUSG00000020644.8	3513,19976	-1,6221335	0,21179087	1,16E-15	3,85E-13	DOWN	Id2	12:25093801-25096092	protein_coding	-
ENSMUSG00000030208.15	1106,43573	-1,8040128	0,2372126	1,70E-15	5,35E-13	DOWN	Emp1	6:135362545-135383173	protein_coding	+
ENSMUSG00000043008.8	1806,07136	-1,7386035	0,22938039	2,03E-15	6,26E-13	DOWN	Klhl6	16:19946499-19983037	protein_coding	-
ENSMUSG00000039943.16	328,509374	-2,4062237	0,3204521	3,40E-15	1,00E-12	DOWN	Plcb4	2:135659011-136014593	protein_coding	+
ENSMUSG00000025809.15	5062,39346	-1,629254	0,21703842	3,62E-15	1,04E-12	DOWN	Itgb1	8:128685654-128733200	protein_coding	+
ENSMUSG00000001020.8	1811,74145	-1,6812064	0,2263177	6,69E-15	1,81E-12	DOWN	S100a4	3:90603771-90606045	protein_coding	+
ENSMUSG00000022013.3	622,601228	-1,9610189	0,26473136	7,72E-15	2,02E-12	DOWN	Dnajc15	14:77826217-77874917	protein_coding	-
ENSMUSG00000003882.4	4408,01531	-1,7894628	0,2503042	3,21E-14	7,87E-12	DOWN	Il7r	15:9506161-9529876	protein_coding	-
ENSMUSG00000076472.2	255,86605	-3,1511859	0,45563679	5,96E-14	1,38E-11	DOWN	Trbv15	6:41141188-41141658	TR_V_gene	+
ENSMUSG00000103779.1	619,892997	-2,1004176	0,29882611	6,50E-14	1,48E-11	DOWN	Gm36931	17:51766643-51771443	TEC	-
ENSMUSG00000032420.7	292,75389	-2,2452458	0,32596536	3,09E-13	6,50E-11	DOWN	Nt5e	9:88327197-88372092	protein_coding	+
ENSMUSG00000020617.13	95,2226528	-4,652692	0,67503401	7,66E-13	1,54E-10	DOWN	1700012B07F	11:109787651-109828046	protein_coding	-
ENSMUSG00000030281.16	98,424532	-5,0903058	0,7409338	1,01E-12	2,00E-10	DOWN	Il17rc	6:113471427-113483140	protein_coding	+
ENSMUSG00000061577.11	344,744329	-2,3487582	0,35607033	1,18E-12	2,31E-10	DOWN	Adgrg5	8:94923694-94943290	protein_coding	+
ENSMUSG00000028525.16	1560,9847	-1,515747	0,229551	2,62E-12	4,82E-10	DOWN	Pde4b	4:102087543-102607259	protein_coding	+
ENSMUSG00000102212.1	551,571223	-2,020391	0,31254111	3,20E-12	5,73E-10	DOWN	C230085N15I	17:51757295-51761621	TEC	-
ENSMUSG00000095574.2	130,944322	-3,0384734	0,46789557	4,34E-12	7,56E-10	DOWN	Trbv12-1	6:41113550-41114067	TR_V_gene	+
ENSMUSG00000102973.1	318,540504	-2,1669512	0,33773548	5,29E-12	8,98E-10	DOWN	E430014B02F	17:51745529-51748684	TEC	-
ENSMUSG00000021360.15	459,712075	-2,3533556	0,37704647	5,98E-12	1,00E-09	DOWN	Gcnt2	13:40859768-40960891	protein_coding	+
ENSMUSG00000043807.6	131,382212	-3,0905477	0,48524552	1,09E-11	1,77E-09	DOWN	Ly6g5b	17:35113948-35115428	protein_coding	-
ENSMUSG00000022657.9	1605,99362	-1,4158946	0,22397947	1,78E-11	2,77E-09	DOWN	Cd96	16:46035657-46120251	protein_coding	-
ENSMUSG00000024646.13	666,436426	-1,6024984	0,25676471	2,63E-11	4,01E-09	DOWN	Cyb5a	18:84851338-84880401	protein_coding	+
ENSMUSG00000034220.7	983,964622	-1,4889563	0,23949956	3,29E-11	4,69E-09	DOWN	Gpc1	1:92831645-92860779	protein_coding	+
ENSMUSG00000052821.3	84,808418	-4,7752329	0,76110562	3,67E-11	5,11E-09	DOWN	Cysltr1	X:106574346-106603679	protein_coding	-
ENSMUSG00000021728.7	3262,70129	-1,3092242	0,21069744	3,84E-11	5,30E-09	DOWN	Emb	13:117220625-117274098	protein_coding	+
ENSMUSG00000025461.10	207,710851	-2,2451575	0,36532263	4,25E-11	5,80E-09	DOWN	Cd163l1	7:140218267-140231145	protein_coding	+
ENSMUSG00000026770.5	663,739918	-1,6155665	0,26431065	5,62E-11	7,60E-09	DOWN	Il2ra	2:11642807-11693193	protein_coding	+
ENSMUSG00000052736.15	201,596249	-2,3474248	0,38538476	5,91E-11	7,91E-09	DOWN	Klrc2	6:129647496-129660689	protein_coding	-
ENSMUSG00000019256.17	328,803607	-2,1122275	0,35606166	1,01E-10	1,29E-08	DOWN	Ahr	12:35497974-35535038	protein_coding	-
ENSMUSG00000000682.7	4674,45777	-1,2677821	0,20959119	1,09E-10	1,36E-08	DOWN	Cd52	4:134082448-134095082	protein_coding	-
ENSMUSG00000026070.15	7730,84209	-1,2406799	0,20560311	1,18E-10	1,44E-08	DOWN	Il18r1	1:40465552-40500854	protein_coding	+
ENSMUSG00000033174.17	151,890503	-2,5365548	0,43346715	2,20E-10	2,63E-08	DOWN	Mgll	6:88724412-88828360	protein_coding	+
ENSMUSG00000006342.14	566,399718	-1,5945353	0,27028355	2,32E-10	2,74E-08	DOWN	Susd2	10:75636619-75644008	protein_coding	-
ENSMUSG00000006345.10	650,98532	-1,5361798	0,26396251	3,78E-10	4,24E-08	DOWN	Ggt1	10:75561604-75586200	protein_coding	+
ENSMUSG00000015355.13	2902,01058	-1,2471812	0,21490561	4,90E-10	5,41E-08	DOWN	Cd48	1:171682009-171705258	protein_coding	+
ENSMUSG00000103560.1	74,052399	-4,2864396	0,75565129	7,63E-10	8,21E-08	DOWN	Gm38070	1:40238020-40244499	TEC	+
ENSMUSG00000046807.9	232,650897	-2,1400533	0,37977844	7,85E-10	8,39E-08	DOWN	Lrrc75b	10:75550125-75560330	protein_coding	-
ENSMUSG00000051212.7	711,636025	-1,5526912	0,27574008	9,31E-10	9,86E-08	DOWN	Gpr183	14:121952331-121965193	protein_coding	-
ENSMUSG00000030156.5	1948,31702	-1,2452888	0,21926103	1,02E-09	1,07E-07	DOWN	Cd69	6:129267325-129275436	protein_coding	-
ENSMUSG00000086968.8	84,2486082	-3,3929151	0,60621567	1,55E-09	1,59E-07	DOWN	4933431E20F	3:107888850-107896213	antisense	-
ENSMUSG00000032336.17	2069,37336	-1,2178752	0,21938105	2,16E-09	2,20E-07	DOWN	Nptn	9:58582240-58657955	protein_coding	+
ENSMUSG00000040229.11	75,3518112	-3,8803195	0,69894494	2,18E-09	2,20E-07	DOWN	Gpr34	X:13632089-13640858	protein_coding	+
ENSMUSG00000103216.1	447,543365	-1,8248004	0,3379466	2,41E-09	2,42E-07	DOWN	Gm37248	17:51796017-51799190	TEC	-
ENSMUSG00000043252.8	376,19895	-1,6880448	0,31058098	3,00E-09	2,97E-07	DOWN	Tmem64	4:15265831-15286753	protein_coding	+
ENSMUSG00000074570.13	225,223795	-1,8639494	0,34902842	5,18E-09	5,01E-07	DOWN	Cass4	2:172393794-172433757	protein_coding	+
ENSMUSG00000087497.7	250,622063	-1,8295136	0,34338497	5,82E-09	5,59E-07	DOWN	2810001G20F	11:64079484-64083259	antisense	+
ENSMUSG00000102744.1	204,569943	-2,1440085	0,41030002	6,04E-09	5,76E-07	DOWN	5830444F18F	17:51791470-51793461	TEC	-
ENSMUSG00000025790.14	1034,44122	-1,3058135	0,24579788	7,24E-09	6,85E-07	DOWN	Slco3a1	7:74275419-74554780	protein_coding	-
ENSMUSG00000030844.11	235,454504	-1,8666606	0,3556697	7,46E-09	6,95E-07	DOWN	Rgs10	7:128373621-128418758	protein_coding	-
ENSMUSG00000034586.14	580,82541	-1,5237974	0,28884756	7,44E-09	6,95E-07	DOWN	Hid1	11:115347707-115367756	protein_coding	-
ENSMUSG00000030789.9	295,28011	-1,9277294	0,37250097	8,24E-09	7,63E-07	DOWN	Itgax	7:128129547-128150657	protein_coding	+
ENSMUSG00000065176.1	2623,63263	-1,3223583	0,25408956	1,02E-08	9,18E-07	DOWN	Rnu12	15:83149644-83149794	snRNA	+
ENSMUSG00000049410.8	95,2781028	-2,7003886	0,5212021	1,21E-08	1,07E-06	DOWN	Zfp683	4:134053838-134058996	protein_coding	+
ENSMUSG00000055629.4	522,360195	-1,7398596	0,34677633	1,43E-08	1,23E-06	DOWN	B4galnt4	7:141061274-141072119	protein_coding	+
ENSMUSG00000049866.12	3056,6531	-1,2420786	0,24238054	1,75E-08	1,48E-06	DOWN	Arl4c	1:88673125-88702221	protein_coding	-
ENSMUSG00000026830.9	372,757752	-1,8109467	0,36307305	1,82E-08	1,53E-06	DOWN	Ermn	2:58045113-58052864	protein_coding	-
ENSMUSG00000047898.6	164,178737	-2,1111543	0,41532328	1,93E-08	1,59E-06	DOWN	Ccr4	9:114490316-114496544	protein_coding	-
ENSMUSG00000035725.13	1179,04995	-1,1956274	0,23300857	2,26E-08	1,80E-06	DOWN	Prkx	X:77761411-77796278	protein_coding	-
ENSMUSG00000020009.12	6623,39191	-1,1340272	0,22503233	3,04E-08	2,38E-06	DOWN	Ifngr1	10:19591949-19610229	protein_coding	+
ENSMUSG00000028435.8	109,831447	-2,9127599	0,61347572	3,16E-08	2,46E-06	DOWN	Aqp3	4:41092722-41098183	protein_coding	-
ENSMUSG00000079641.3	1082,0115	-1,2976184	0,25980992	3,46E-08	2,68E-06	DOWN	Rpl39	X:37082520-37085402	protein_coding	-
ENSMUSG00000060112.1	225,351557	-1,7264767	0,35930698	8,04E-08	5,76E-06	DOWN	Olfr60	7:140345052-140345987	protein_coding	-
ENSMUSG00000049608.8	232,803895	-1,6939135	0,3532662	9,55E-08	6,73E-06	DOWN	Gpr55	1:85938318-85961007	protein_coding	-
ENSMUSG00000020520.14	632,853898	-1,5507354	0,33141035	9,82E-08	6,84E-06	DOWN	Galnt10	11:57645442-57787514	protein_coding	+
ENSMUSG00000041912.12	75,3754321	-3,5297281	0,7304124	1,04E-07	7,24E-06	DOWN	Tdrkh	3:94413273-94434668	protein_coding	+
ENSMUSG00000076470.1	69,6817399	-3,3985726	0,72727945	1,12E-07	7,72E-06	DOWN	Trbv13-3	6:41130147-41130585	TR_V_gene	+
ENSMUSG00000021831.8	980,784673	-1,3432802	0,28789841	1,59E-07	1,07E-05	DOWN	Ero1l	14:45283092-45318572	protein_coding	-
ENSMUSG00000018398.18	311,878934	-1,5478437	0,33307051	1,74E-07	1,16E-05	DOWN	Sep 08	11:53519257-53549565	protein_coding	+
ENSMUSG00000015533.8	485,761679	-1,5090156	0,32760461	1,87E-07	1,24E-05	DOWN	Itga2	13:114835916-114932041	protein_coding	-
ENSMUSG00000020027.18	283,768071	-1,7659271	0,39060561	2,03E-07	1,34E-05	DOWN	Socs2	10:95385362-95417180	protein_coding	-
ENSMUSG00000034591.5	103,497916	-2,4130269	0,53575957	2,34E-07	1,51E-05	DOWN	Slc41a2	10:83230848-83337882	protein_coding	-
ENSMUSG00000031132.1	457,0797	-1,4787999	0,32475772	2,42E-07	1,55E-05	DOWN	Cd40lg	X:57212143-57224042	protein_coding	+
ENSMUSG00000045362.8	354,449231	-1,3990781	0,30368192	2,66E-07	1,70E-05	DOWN	Tnfrsf26	7:143607659-143628722	protein_coding	-
ENSMUSG00000040747.9	4681,07167	-1,1741015	0,25647863	2,89E-07	1,83E-05	DOWN	Cd53	3:106759921-106790149	protein_coding	-
ENSMUSG00000015709.9	206,99561	-1,6463567	0,36109365	2,92E-07	1,84E-05	DOWN	Arnt2	7:84246278-84410176	protein_coding	-
ENSMUSG00000027605.18	248,318957	-1,5763905	0,34370141	2,93E-07	1,84E-05	DOWN	Acss2	2:155517948-155585724	protein_coding	+
ENSMUSG00000047821.16	370,767674	-1,4702287	0,3236301	3,03E-07	1,90E-05	DOWN	Trim16	11:62820231-62850808	protein_coding	+
ENSMUSG00000049775.16	27934,2472	-0,9266735	0,20040346	3,88E-07	2,37E-05	DOWN	Tmsb4x	X:167207093-167209315	protein_coding	-
ENSMUSG00000026009.14	1881,60121	-1,1861585	0,26383474	3,97E-07	2,40E-05	DOWN	Icos	1:60977927-61000320	protein_coding	+
ENSMUSG00000029840.8	2598,38994	-1,0491226	0,23287827	5,06E-07	2,96E-05	DOWN	Mtpn	6:35508906-35539888	protein_coding	-
ENSMUSG00000019876.15	65,3478754	-2,9061629	0,66468012	5,23E-07	3,05E-05	DOWN	Pkib	10:57631981-57741112	protein_coding	+

ENSMUSG00000022724.15	1005,38562	-1,1033739	0,24706676	6,35E-07	3,65E-05	DOWN	Mina	16:59471775-59492461	protein_coding	+
ENSMUSG00000046949.15	448,113836	-1,265284	0,28977791	9,23E-07	5,14E-05	DOWN	Nqo2	13:33964659-33988465	protein_coding	+
ENSMUSG00000019951.9	1908,51211	-0,9815217	0,22269185	9,62E-07	5,31E-05	DOWN	Uhrf1bp1l	10:89744991-89819869	protein_coding	+
ENSMUSG00000045991.18	48,0270294	-5,5040238	1,16971329	1,01E-06	5,54E-05	DOWN	Onecut2	18:64340364-64398488	protein_coding	+
ENSMUSG00000040183.13	124,717101	-1,957271	0,46187294	1,09E-06	5,91E-05	DOWN	Ankrd6	4:32804035-32950841	protein_coding	-
ENSMUSG00000045092.8	2966,50045	-0,9454653	0,21533577	1,10E-06	5,93E-05	DOWN	S1pr1	3:115710433-115715072	protein_coding	-
ENSMUSG00000042272.17	290,874883	-1,5385326	0,36995621	1,27E-06	6,71E-05	DOWN	Sestd1	2:77180340-77280592	protein_coding	-
ENSMUSG00000024317.14	808,916392	-1,0774377	0,24933611	1,28E-06	6,71E-05	DOWN	Rnf138	18:21001341-21028223	protein_coding	+
ENSMUSG00000020865.16	66,7658293	-2,5999209	0,62614256	1,46E-06	7,60E-05	DOWN	Abcc3	11:94343295-94392997	protein_coding	-
ENSMUSG00000020614.13	108,103854	-2,0494565	0,49593505	1,61E-06	8,15E-05	DOWN	Fam20a	11:109669749-109722279	protein_coding	-
ENSMUSG00000041959.14	4179,30735	-0,9395202	0,21912614	1,71E-06	8,57E-05	DOWN	S100a10	3:93555080-93564643	protein_coding	+
ENSMUSG00000030530.15	7844,26931	-0,9218708	0,21429088	1,71E-06	8,57E-05	DOWN	Furin	7:80388585-80405436	protein_coding	-
ENSMUSG00000005947.11	70,8449847	-2,4198254	0,59052061	2,07E-06	0,00010192	DOWN	Itgae	11:73090583-73147446	protein_coding	+
ENSMUSG00000020601.7	1200,49744	-0,9896904	0,23402963	2,13E-06	0,00010413	DOWN	Trib2	12:15791727-15816877	protein_coding	-
ENSMUSG00000034751.15	371,22732	-1,2450073	0,29898531	2,15E-06	0,00010449	DOWN	Mast4	13:102732486-103334497	protein_coding	-
ENSMUSG00000019943.9	3458,17716	-0,9381934	0,22169473	2,15E-06	0,00010449	DOWN	Atp2b1	10:98915152-99026143	protein_coding	+
ENSMUSG00000023132.7	737,496837	-1,1911517	0,28969975	2,33E-06	0,00011281	DOWN	Gzma	13:113093825-113100979	protein_coding	-
ENSMUSG00000022270.15	694,263056	-1,0795946	0,25708077	2,37E-06	0,00011383	DOWN	Fam134b	15:25843264-25973687	protein_coding	+
ENSMUSG00000017897.18	81,7428046	-2,5011983	0,63682238	2,40E-06	0,00011476	DOWN	Eya2	2:165595032-165771727	protein_coding	+
ENSMUSG00000032184.4	168,849417	-1,6433176	0,40366589	2,43E-06	0,00011572	DOWN	Lysmd2	9:75625732-75637773	protein_coding	+
ENSMUSG00000070056.5	631,398732	-1,08577	0,26033465	2,48E-06	0,00011781	DOWN	Mfhas1	8:35587798-35679449	protein_coding	+
ENSMUSG00000068220.5	7420,04616	-0,9083287	0,21644183	2,65E-06	0,00012422	DOWN	Lgals1	15:78926725-78930465	protein_coding	+
ENSMUSG00000026019.15	517,797621	-1,2726811	0,31533063	2,85E-06	0,00013313	DOWN	Wdr12	1:60069785-60098645	protein_coding	-
ENSMUSG00000054555.11	80,2278545	-2,4906131	0,63507904	2,92E-06	0,00013459	DOWN	Adam12	7:133883199-134232146	protein_coding	-
ENSMUSG00000005087.17	3829,7238	-0,9565401	0,23015076	3,22E-06	0,00014693	DOWN	Cd44	2:102811141-102901665	protein_coding	-
ENSMUSG00000034117.3	50,8473004	-4,2829985	1,00462181	3,52E-06	0,0001595	DOWN	Ptgdr2	19:10937160-10942511	protein_coding	+
ENSMUSG00000035891.16	291,544101	-1,2796778	0,32029081	4,35E-06	0,00019083	DOWN	Cerk	15:86139128-86186141	protein_coding	-
ENSMUSG00000049093.9	96,3614259	-2,0935085	0,54791495	4,59E-06	0,00019984	DOWN	Il23r	6:67422932-67491855	protein_coding	-
ENSMUSG00000032656.14	184,617257	-1,4916024	0,38197336	5,34E-06	0,00023027	DOWN	Mrz 03	18:56761716-56925548	protein_coding	-
ENSMUSG00000040451.17	1500,46789	-0,9145881	0,22630837	5,36E-06	0,00023059	DOWN	Sgms1	19:32122727-32389714	protein_coding	-
ENSMUSG00000063410.7	3740,53397	-0,8688556	0,21554528	5,88E-06	0,00025217	DOWN	Stk24	14:121286343-121379334	protein_coding	-
ENSMUSG00000044345.9	392,419891	-1,24683	0,32203906	6,29E-06	0,00026798	DOWN	Marveld1	19:42147400-42151703	protein_coding	+
ENSMUSG00000027374.12	371,723282	-1,1754018	0,30229036	6,95E-06	0,00029241	DOWN	Mrps5	2:127587222-127606829	protein_coding	+
ENSMUSG00000027375.14	46,62502	-6,7314409	2,62381659	7,33E-06	0,00030618	DOWN	Mal	2:127633226-127656695	protein_coding	-
ENSMUSG00000001663.10	99,3233502	-1,9514218	0,51901093	7,40E-06	0,00030833	DOWN	Gstt1	10:75783813-75798584	protein_coding	-
ENSMUSG00000065232.1	2386,56894	-1,0785691	0,28225643	7,52E-06	0,00031119	DOWN	Gm22973	12:59040335-59040525	snRNA	+
ENSMUSG00000028460.6	535,630496	-1,0628029	0,27328729	8,23E-06	0,00033842	DOWN	Sit1	4:43482081-43483734	protein_coding	-
ENSMUSG00000009687.14	3271,70888	-0,8415843	0,21404681	9,52E-06	0,00038437	DOWN	Fxyd5	7:31032722-31042481	protein_coding	-
ENSMUSG00000017754.13	166,699283	-1,4702385	0,39418996	1,09E-05	0,00043378	DOWN	Pltp	2:164839518-164857711	protein_coding	-
ENSMUSG00000097415.2	3633,74904	-0,8364867	0,21429544	1,15E-05	0,00045466	DOWN	AU020206	7:75769038-75782099	lincRNA	-
ENSMUSG00000028843.8	2499,10784	-0,843666	0,21782618	1,19E-05	0,00046876	DOWN	Sh3bgrl3	4:134127406-134128789	protein_coding	-
ENSMUSG00000093843.1	6139,7157	-0,8613254	0,22346534	1,23E-05	0,00048108	DOWN	Gm25939	1:72255008-72255198	snRNA	+
ENSMUSG00000031304.18	1862,39432	-0,8550576	0,22178473	1,26E-05	0,00049006	DOWN	Il2rg	X:101264378-101268255	protein_coding	-
ENSMUSG00000076476.1	54,0252028	-3,1228779	0,86936365	1,40E-05	0,0005358	DOWN	Trbv20	6:41188273-41188977	TR_V_gene	+
ENSMUSG00000001025.8	2314,94382	-0,8549208	0,22313994	1,41E-05	0,00054069	DOWN	S100a6	3:90612882-90624181	protein_coding	+
ENSMUSG00000028480.14	3370,55353	-0,8084415	0,21159543	1,53E-05	0,00058156	DOWN	Glipr2	4:43957401-43979118	protein_coding	+
ENSMUSG00000000594.7	1547,46557	-0,9520876	0,25503547	1,57E-05	0,00059528	DOWN	Gm2a	11:55098115-55113029	protein_coding	+
ENSMUSG00000020889.11	38,9966559	-3,0997466	0,85466901	1,60E-05	0,00060276	DOWN	Nr1d1	11:98767932-98775333	protein_coding	-
ENSMUSG00000076467.3	98,3252653	-1,7863961	0,49781395	1,64E-05	0,0006159	DOWN	Trbv13-1	6:41116007-41116468	TR_V_gene	+
ENSMUSG00000029641.8	80,3990709	-2,147133	0,60095341	1,66E-05	0,00061994	DOWN	Rasl11a	5:146845071-146847726	protein_coding	+
ENSMUSG00000053716.9	406,490279	-1,0849117	0,29333783	1,66E-05	0,00061994	DOWN	Dusp7	9:106368632-106375724	protein_coding	+
ENSMUSG00000030165.16	228,151756	-1,2950954	0,35449699	1,68E-05	0,00062378	DOWN	Klrd1	6:129591782-129598775	protein_coding	+
ENSMUSG00000044465.18	793,226529	-0,9923691	0,26836914	1,78E-05	0,00065633	DOWN	Fam160a2	7:105371211-105400054	protein_coding	-
ENSMUSG00000031278.12	1283,45548	-0,9314403	0,2506897	1,81E-05	0,00066287	DOWN	Acsl4	X:142317993-142390535	protein_coding	-
ENSMUSG00000104620.1	39,0768152	-2,9156099	0,81966385	1,88E-05	0,00068455	DOWN	Trav7-1	14:52654817-52655328	TR_V_gene	+
ENSMUSG00000012123.15	119,446549	-1,6174282	0,45544568	1,98E-05	0,00071533	DOWN	Aim1l	4:134065912-134092504	protein_coding	+
ENSMUSG00000031391.18	1282,2277	-0,8789188	0,23461643	1,98E-05	0,00071533	DOWN	L1cam	X:73853778-73896105	protein_coding	-
ENSMUSG00000026919.1	75,5454262	-2,0284111	0,57886335	2,25E-05	0,00080074	DOWN	Lcn4	2:26667674-26671282	protein_coding	-
ENSMUSG00000089672.4	2758,28165	-0,8927413	0,24409292	2,26E-05	0,00080074	DOWN	Lilr4b	10:51480632-51486316	protein_coding	+
ENSMUSG00000000782.15	1453,01218	-0,9433169	0,25987664	2,31E-05	0,00081635	DOWN	Tcf7	11:52252371-52283014	protein_coding	-
ENSMUSG00000044505.5	37,5711757	-3,2553042	0,92012759	2,63E-05	0,00091905	DOWN	Lingo4	3:94398517-94404501	protein_coding	+
ENSMUSG00000080538.1	222,867573	-1,3622336	0,38912354	2,65E-05	0,00092461	DOWN	Gm25541	2:27539805-27539930	snRNA	-
ENSMUSG00000043004.13	1917,78799	-0,8293689	0,22652903	2,82E-05	0,00097792	DOWN	Gng2	14:19872559-19977627	protein_coding	-
ENSMUSG00000025473.16	2193,06289	-0,8645138	0,23862048	2,87E-05	0,00098593	DOWN	Adam8	7:139978932-139992562	protein_coding	-
ENSMUSG00000009927.8	2480,98762	-0,7886869	0,21605864	3,14E-05	0,00106648	DOWN	Rps25	9:44407714-44410405	protein_coding	+
ENSMUSG00000050592.8	1644,26694	-0,8262264	0,22767714	3,21E-05	0,0010881	DOWN	Fam78a	2:32050055-32084857	protein_coding	-
ENSMUSG00000020423.6	2430,63374	-0,8015908	0,22282995	3,23E-05	0,00109267	DOWN	Btg2	1:134075170-134079120	protein_coding	-
ENSMUSG00000079138.3	102,197074	-1,7366554	0,51762854	3,32E-05	0,00111451	DOWN	Gm8818	1:153305432-153306478	processed_pseudogene	+
ENSMUSG00000036503.13	596,946627	-0,9976432	0,28184148	3,37E-05	0,00112837	DOWN	Rnf13	3:57736062-57835233	protein_coding	+
ENSMUSG00000066026.14	227,504835	-1,2185284	0,3506506	3,40E-05	0,00113241	DOWN	Dhrs3	4:144892827-144928209	protein_coding	+
ENSMUSG00000032750.13	284,752764	-1,1391906	0,32634962	3,44E-05	0,00113831	DOWN	Gab3	X:74966843-75085458	protein_coding	-
ENSMUSG00000028618.11	774,109033	-0,911727	0,25677392	3,59E-05	0,0011858	DOWN	Tmem59	4:107178399-107200996	protein_coding	+
ENSMUSG00000020715.9	1362,92457	-0,8441017	0,23621771	3,77E-05	0,00123861	DOWN	Ern1	11:106394650-106487852	protein_coding	-
ENSMUSG00000051504.18	59,2251959	-2,8968877	1,01901091	3,87E-05	0,0012587	DOWN	Siglech	7:55768178-55778925	protein_coding	+
ENSMUSG00000031015.7	464,082965	-1,084316	0,31639169	3,94E-05	0,00126406	DOWN	Swap70	7:110221703-110283503	protein_coding	+
ENSMUSG00000032412.8	3435,02078	-0,8311822	0,23332323	3,92E-05	0,00126406	DOWN	Atp1b3	9:96332655-96364442	protein_coding	-
ENSMUSG00000055170.3	1619,10735	-0,8654793	0,24577764	4,21E-05	0,00133582	DOWN	Ifng	10:118441047-118445892	protein_coding	+
ENSMUSG00000071180.4	618,32575	-0,9444148	0,27011709	4,28E-05	0,00135334	DOWN	Smim15	13:108046424-108049146	protein_coding	+
ENSMUSG00000027854.12	1442,81834	-0,8183194	0,23035765	4,35E-05	0,00137311	DOWN	Sike1	3:102995708-103008459	protein_coding	+
ENSMUSG00000026478.14	1693,65625	-0,8471542	0,24021874	4,37E-05	0,00137618	DOWN	Lamc1	1:153218922-153332786	protein_coding	-
ENSMUSG00000024235.10	768,098421	-0,8856099	0,25289163	4,59E-05	0,00143704	DOWN	Map3k8	18:4331327-4353015	protein_coding	-
ENSMUSG00000030775.9	649,227341	-0,9732207	0,28354596	4,81E-05	0,0014996	DOWN	Trat1	16:48734690-48771956	protein_coding	-
ENSMUSG00000037003.15	188,030004	-1,3354677	0,40151949	4,84E-05	0,00150577	DOWN	Tns2	15:102102988-102116401	protein_coding	+
ENSMUSG00000006360.11	2505,25334	-0,8370131	0,23815267	4,89E-05	0,0015164	DOWN	Crip1	12:113146316-113153879	protein_coding	+

ENSMUSG00000036944.5	1361,04778	-0,8106685	0,22998028	4,92E-05	0,00151861	DOWN	Tmem71	15:66526212-66561046	protein_coding	-
ENSMUSG00000054843.8	812,058036	-0,8883767	0,25572447	5,04E-05	0,00154627	DOWN	Atrnl1	19:57611034-58133338	protein_coding	+
ENSMUSG00000024910.4	1756,71476	-0,9548898	0,28306033	5,08E-05	0,001554	DOWN	Ctsw	19:5465240-5468498	protein_coding	-
ENSMUSG00000042747.12	433,302546	-0,9862601	0,29002794	5,20E-05	0,00158665	DOWN	Krtcap2	3:89245966-89249906	protein_coding	+
ENSMUSG00000072235.5	657,529287	-0,9067535	0,26206024	5,34E-05	0,00162272	DOWN	Tuba1a	15:98949841-98953551	protein_coding	-
ENSMUSG00000025058.4	178,657597	-1,3895487	0,42711393	5,43E-05	0,00164132	DOWN	5430427O19I	X:85870354-85891499	protein_coding	+
ENSMUSG00000040703.11	83,0481464	-1,788426	0,55446831	5,49E-05	0,00165715	DOWN	Cyp2s1	7:25802475-25816913	protein_coding	-
ENSMUSG00000032249.14	1067,80061	-0,8417818	0,24288677	5,66E-05	0,00169834	DOWN	Anp32a	9:62341293-62378812	protein_coding	+
ENSMUSG00000035199.6	1674,01884	-0,785045	0,22440966	5,76E-05	0,00171876	DOWN	Arl6ip5	6:97210689-97233315	protein_coding	+
ENSMUSG00000076463.2	153,476298	-1,6463108	0,52632663	5,79E-05	0,00172418	DOWN	Trbv3	6:41048290-41048828	TR_V_gene	+
ENSMUSG00000060802.8	14842,6951	-0,7616666	0,21807233	5,93E-05	0,00176147	DOWN	B2m	2:122147686-122153083	protein_coding	+
ENSMUSG00000039153.16	2169,17181	-0,7632748	0,21771179	6,03E-05	0,00178737	DOWN	Runx2	17:44495987-44814797	protein_coding	-
ENSMUSG00000003153.10	1665,84018	-0,8549568	0,24955883	6,15E-05	0,00181545	DOWN	Slc2a3	6:122727809-122801640	protein_coding	-
ENSMUSG00000016087.13	1855,42738	-0,775109	0,22253473	6,21E-05	0,00182638	DOWN	Fli1	9:32422204-32542861	protein_coding	-
ENSMUSG00000097365.7	67,820348	-1,9749999	0,6187665	6,54E-05	0,00191182	DOWN	C030034L19F	3:9403064-9437233	lincRNA	+
ENSMUSG00000026384.13	1614,16544	-0,7793482	0,22446012	6,59E-05	0,00192143	DOWN	Ptpn4	1:119652467-119837613	protein_coding	-
ENSMUSG00000024124.9	40,2557696	-2,6178731	0,82109665	6,60E-05	0,0019222	DOWN	Prss30	17:23972126-23975230	protein_coding	-
ENSMUSG00000014932.15	75,6571423	-1,8996508	0,60447933	6,66E-05	0,00193312	DOWN	Yes1	5:32611171-32687057	protein_coding	+
ENSMUSG00000000686.11	141,849958	-1,3641718	0,42050584	6,70E-05	0,00193878	DOWN	Abhd15	11:77515121-77538607	protein_coding	+
ENSMUSG00000008683.16	3732,58011	-0,7562613	0,21781547	6,70E-05	0,00193878	DOWN	Rps15a	7:118104372-118116188	protein_coding	-
ENSMUSG00000021733.9	1908,06294	-0,7710517	0,22281958	6,74E-05	0,00194524	DOWN	Slc4a7	14:14703025-14799943	protein_coding	+
ENSMUSG00000093661.1	462,455203	-0,9425998	0,28196998	7,60E-05	0,00216959	DOWN	Eif4e3	6:99625135-99666771	protein_coding	-
ENSMUSG00000038393.14	4965,63663	-0,749708	0,21984707	7,75E-05	0,00220744	DOWN	Txnip	3:96557957-96561883	protein_coding	+
ENSMUSG00000044408.6	465,844789	-1,0110877	0,30861139	7,81E-05	0,00221443	DOWN	Sptssa	12:54645374-54656572	protein_coding	-
ENSMUSG00000017485.10	3734,28458	-0,7299464	0,21193507	7,86E-05	0,00222294	DOWN	Top2b	14:16365179-16430787	protein_coding	+
ENSMUSG00000025531.14	704,517039	-0,8609187	0,25702878	8,05E-05	0,00226978	DOWN	Chm	X:113040593-113185517	protein_coding	-
ENSMUSG00000037815.6	655,083292	-0,9221669	0,28102159	9,28E-05	0,00258747	DOWN	Ctnna1	18:35118888-35254773	protein_coding	+
ENSMUSG00000074695.3	15,7881381	-6,3134172	2,81021302	9,32E-05	0,00259281	DOWN	Il22	10:118204942-118210047	protein_coding	+
ENSMUSG00000029213.11	738,565466	-0,8330549	0,25401255	9,49E-05	0,00263413	DOWN	Commd8	5:72156575-72168189	protein_coding	-
ENSMUSG00000064797.1	196,111453	-1,2605311	0,4031305	9,55E-05	0,00264613	DOWN	Gm24357	9:15315522-15315595	snoRNA	+
ENSMUSG00000075602.10	3287,35864	-0,7589988	0,22671521	0,00010032	0,00275664	DOWN	Ly6a	15:74994877-74998031	protein_coding	-
ENSMUSG00000020949.8	410,160443	-0,9507636	0,29548488	0,00010909	0,00299135	DOWN	Fkbp3	12:65062436-65073944	protein_coding	-
ENSMUSG00000031231.4	595,518399	-0,8772844	0,26763768	0,00010965	0,00300039	DOWN	Cox7b	X:106015700-106022450	protein_coding	+
ENSMUSG00000015671.10	1078,88847	-0,8322554	0,25435017	0,00011048	0,00301069	DOWN	Psma2	13:14613242-14625671	protein_coding	+
ENSMUSG00000025647.16	6818,47543	-0,6935182	0,20545563	0,00011223	0,00305221	DOWN	Shisa5	9:109038565-109057777	protein_coding	+
ENSMUSG00000060591.8	819,095856	-2,4412658	0,89467087	0,00011321	0,00307178	DOWN	Ifitm2	7:140954839-140955961	protein_coding	-
ENSMUSG00000108436.1	55,1469127	-2,0799415	0,70080523	0,00011342	0,00307178	DOWN	RP23-137A24	7:80404269-80406577	antisense	+
ENSMUSG00000064373.11	376,205184	-1,0965491	0,35561607	0,00012305	0,00327907	DOWN	Sepp1	15:3268547-3280508	protein_coding	+
ENSMUSG00000033446.7	424,527444	-0,9909696	0,31457357	0,00012449	0,00330422	DOWN	Lpar6	14:73237895-73243294	protein_coding	+
ENSMUSG00000003623.4	1612,23637	-0,7775732	0,23790577	0,00012637	0,00334723	DOWN	Crot	5:8966033-8997324	protein_coding	-
ENSMUSG00000034833.9	444,896753	-0,9188658	0,28745924	0,00012696	0,00334963	DOWN	Tespa1	10:130322852-130362642	protein_coding	+
ENSMUSG00000022106.14	1015,31109	-0,7819508	0,23891443	0,00012689	0,00334963	DOWN	Rcbtb2	14:73123037-73207843	protein_coding	+
ENSMUSG00000022014.14	2032,59951	-0,7690307	0,2362652	0,00013286	0,00347753	DOWN	Epsti1	14:77904239-78002656	protein_coding	+
ENSMUSG00000050335.16	2353,87808	-0,713105	0,21760164	0,00014168	0,00366478	DOWN	Lgals3	14:47367751-47386160	protein_coding	+
ENSMUSG00000038764.14	483,462524	-0,8904555	0,28068319	0,00014536	0,00375264	DOWN	Ptpn3	4:57190841-57301837	protein_coding	-
ENSMUSG00000026594.14	492,024596	-0,8760865	0,27751053	0,00015229	0,00389359	DOWN	Ralgps2	1:156804166-156939626	protein_coding	-
ENSMUSG00000026068.11	8775,73983	-0,6664229	0,20289053	0,00015738	0,00400836	DOWN	Il18rap	1:40515362-40551705	protein_coding	+
ENSMUSG00000005824.6	315,069537	-0,9813812	0,31844267	0,00016912	0,00427439	DOWN	Tnfsf14	17:57189492-57194189	protein_coding	-
ENSMUSG00000058587.7	1806,57304	-0,7272866	0,2257805	0,00017344	0,0043656	DOWN	Tmod3	9:75497796-75559657	protein_coding	-
ENSMUSG00000062014.12	1626,76513	-0,7232492	0,22561576	0,0001837	0,0045818	DOWN	Gmfb	14:46808149-46822242	protein_coding	-
ENSMUSG00000000088.6	716,785744	-0,8213073	0,26294107	0,00018817	0,00467556	DOWN	Cox5a	9:57521232-57532424	protein_coding	+
ENSMUSG00000023074.11	165,022959	-1,1940206	0,40409021	0,00019381	0,00478879	DOWN	Mospd1	X:53344598-53370502	protein_coding	-
ENSMUSG00000009418.15	88,323018	-1,5462303	0,53561077	0,000195	0,00480041	DOWN	Nav1	1:135434580-135607295	protein_coding	-
ENSMUSG00000029254.16	692,51207	-0,8112725	0,25935002	0,00019465	0,00480041	DOWN	Stap1	5:86071746-86106125	protein_coding	+
ENSMUSG00000032238.17	5661,41066	-0,6748067	0,21019394	0,0002018	0,00494026	DOWN	Rora	9:68653786-69388246	protein_coding	+
ENSMUSG00000087651.2	199,688665	-1,1587188	0,39728667	0,00020592	0,00502256	DOWN	1500009L16F	10:83722865-83762761	protein_coding	+
ENSMUSG00000022587.14	7365,0613	-0,6671371	0,20835909	0,00021309	0,00517817	DOWN	Ly6e	15:74955051-74959905	protein_coding	+
ENSMUSG00000016758.3	105,242201	-1,6266124	0,6311414	0,00021623	0,00524496	DOWN	Bik	15:83526862-83544635	protein_coding	+
ENSMUSG00000026012.2	3750,28458	-0,7650561	0,2498256	0,00021964	0,005318	DOWN	Cd28	1:60716800-60773359	protein_coding	+
ENSMUSG00000023075.9	791,431972	-0,7821308	0,25176994	0,00022179	0,00535052	DOWN	Akirin1	4:123734559-123750345	protein_coding	-
ENSMUSG00000064341.1	9748,17553	-0,6844106	0,21593936	0,0002217	0,00535052	DOWN	mt-Nd1	MT:2751-3707	protein_coding	+
ENSMUSG00000043230.2	467,182842	-0,9663476	0,32872546	0,00022551	0,00539938	DOWN	Fam124b	1:80198706-80218473	protein_coding	-
ENSMUSG00000031309.15	1523,70879	-0,8560142	0,28290628	0,00022586	0,00539938	DOWN	Rps6ka3	X:159210307-159368244	protein_coding	+
ENSMUSG00000028211.11	2965,32814	-0,6778548	0,21333066	0,00022519	0,00539938	DOWN	Trp53inp1	4:11156431-11174379	protein_coding	+
ENSMUSG00000090841.1	1984,35757	-0,6976989	0,22194883	0,000239	0,00568281	DOWN	Myl6	10:128490860-128493875	protein_coding	-
ENSMUSG00000034401.16	297,708289	-0,9580973	0,32216103	0,0002461	0,00584112	DOWN	Spata6	4:111719984-111829184	protein_coding	+
ENSMUSG00000013707.3	574,858116	-0,8315029	0,27486255	0,00025537	0,00605037	DOWN	Tnfaip8l2	3:95139521-95142360	protein_coding	-
ENSMUSG00000079186.2	32,6735784	-2,5726706	0,9372087	0,00025828	0,00609818	DOWN	Gzmc	14:56231401-56234656	protein_coding	-
ENSMUSG00000017466.9	302,986978	-0,9477097	0,31989912	0,00025831	0,00609818	DOWN	Timp2	11:118301069-118355740	protein_coding	-
ENSMUSG00000004383.17	66,739365	-1,6373132	0,59509007	0,00026103	0,00615134	DOWN	Large	8:72814599-73353540	protein_coding	-
ENSMUSG00000084786.9	511,186071	-0,8364866	0,27711398	0,00026662	0,00627207	DOWN	Ubl5	9:20642878-20647140	protein_coding	+
ENSMUSG00000026547.15	2784,81919	-0,6823991	0,21967008	0,00026718	0,00627393	DOWN	Tagln2	1:172500047-172507380	protein_coding	+
ENSMUSG00000048534.6	235,314357	-1,0242572	0,35295651	0,00027196	0,00636378	DOWN	Amica1	9:45079183-45108530	protein_coding	+
ENSMUSG00000026728.9	16901,747	-0,6413789	0,20379574	0,00027608	0,00644878	DOWN	Vim	2:13573927-13582826	protein_coding	+
ENSMUSG00000024387.13	904,278301	-0,7447848	0,24436692	0,00028927	0,00669765	DOWN	Csnk2b	17:35116196-35122053	protein_coding	-
ENSMUSG00000050912.15	1565,832	-0,7567126	0,25074647	0,00029224	0,00675474	DOWN	Tmem123	9:7764041-7794333	protein_coding	+
ENSMUSG00000043102.2	107,237388	-1,3548385	0,48930033	0,00029393	0,00677008	DOWN	Qrfp	2:31806166-31810580	protein_coding	-
ENSMUSG00000025362.5	1777,28009	-0,7660337	0,25459945	0,0002937	0,00677008	DOWN	Rps26	10:128624530-128626506	protein_coding	-
ENSMUSG00000030729.17	627,326875	-0,8723924	0,29930017	0,00029737	0,00681636	DOWN	Pgm2l1	7:100227394-100278868	protein_coding	+
ENSMUSG00000079523.8	4961,19243	-0,7096995	0,2341515	0,00030828	0,00701524	DOWN	Tmsb10	6:72957347-72958748	protein_coding	-
ENSMUSG00000060636.13	1352,56225	-0,7016269	0,22987062	0,00031159	0,00707846	DOWN	Rpl35a	16:33056453-33060189	protein_coding	+
ENSMUSG00000042043.5	303,087981	-0,946394	0,32722477	0,00031508	0,00714549	DOWN	Tbca	13:94788943-94842899	protein_coding	+
ENSMUSG00000059920.9	828,3859	-0,7522361	0,24969574	0,00032417	0,00731395	DOWN	4930453N24I	16:64751751-64771161	protein_coding	-
ENSMUSG00000025935.10	3158,72432	-0,6816745	0,22311106	0,00032502	0,00732066	DOWN	Tram1	1:13564698-13589910	protein_coding	-

ENSMUSG00000021253.6	85,8366683	-1,4836993	0,54715526	0,00033122	0,0074476	DOWN	Tgfb3	12:86056744-86079041	protein_coding	-
ENSMUSG00000058600.13	1341,18718	-0,715533	0,23685299	0,00033544	0,00751695	DOWN	Rpl30	15:34440505-34443640	protein_coding	-
ENSMUSG00000053477.16	144,209505	-1,2102168	0,44452575	0,00034723	0,00772893	DOWN	Tcf4	18:69343356-69689079	protein_coding	+
ENSMUSG00000019866.13	3180,36132	-0,6531257	0,21405874	0,00034846	0,00773037	DOWN	Aim1	10:43950307-44148853	protein_coding	-
ENSMUSG00000022604.18	1126,98222	-0,7339846	0,24619145	0,00035782	0,00791137	DOWN	Cep97	16:55899888-55934855	protein_coding	-
ENSMUSG00000059708.12	117,467262	-1,2614088	0,46381956	0,00037124	0,00814019	DOWN	Akap17b	X:36608314-36645395	protein_coding	-
ENSMUSG00000026535.9	24,2216425	-3,0695324	1,14963341	0,00038322	0,00836136	DOWN	Ifi202b	1:173962568-173982744	protein_coding	-
ENSMUSG00000075010.4	3244,90309	-0,6930939	0,23267465	0,00038439	0,00837308	DOWN	AW112010	19:11047617-11050566	lincRNA	-
ENSMUSG00000026832.12	6261,57423	-0,6261984	0,20463212	0,00038532	0,00837962	DOWN	Cytip	2:58129137-58195532	protein_coding	-
ENSMUSG00000037826.5	525,576175	-0,7930947	0,27318463	0,00041265	0,00887201	DOWN	Ppm1k	6:57506502-57535468	protein_coding	-
ENSMUSG00000064370.1	8468,68604	-0,6551803	0,21838166	0,00041348	0,00887544	DOWN	mt-Cytb	MT:14145-15288	protein_coding	+
ENSMUSG00000029658.17	89,3596667	-1,6747466	0,76273223	0,00042305	0,00906611	DOWN	Wdr95	5:149528679-149611894	protein_coding	+
ENSMUSG00000047415.11	1509,32369	-0,6826917	0,23014205	0,00043704	0,00932081	DOWN	Gpr68	12:100876682-100908198	protein_coding	-
ENSMUSG00000053897.15	94,71974	-1,4808326	0,59649051	0,00044867	0,00949228	DOWN	Slc39a8	3:135825279-135888572	protein_coding	+
ENSMUSG00000076462.2	145,422518	-1,2140406	0,45410309	0,00046194	0,00974317	DOWN	Trbv2	6:41047340-41047995	TR_V_gene	+
ENSMUSG00000035227.7	919,520091	-0,7863728	0,2785741	0,0004668	0,00981326	DOWN	Spcs2	7:99837569-99863462	protein_coding	-
ENSMUSG00000020263.14	560,804813	-0,7876314	0,27596867	0,00047136	0,00987759	DOWN	Appl2	10:83600033-83648738	protein_coding	-
ENSMUSG00000037346.4	34,7872375	-3,2693131	1,23324979	0,00047375	0,00991211	DOWN	Hrh4	18:13006990-13022882	protein_coding	+
ENSMUSG00000033306.14	1068,62924	-0,7004905	0,23953127	0,00047553	0,00993368	DOWN	Lpp	16:24393350-24992576	protein_coding	+
ENSMUSG00000001627.11	1199,64965	-0,6955595	0,2367772	0,00047765	0,00996223	DOWN	Ifrd1	12:40201567-40248504	protein_coding	-
ENSMUSG00000021520.4	250,070091	-0,9727778	0,3564144	0,00048011	0,00999775	DOWN	Uqcrb	13:66900617-66905378	protein_coding	-
ENSMUSG00000006699.17	4692,49948	-0,6635237	0,22321092	0,00048448	0,01007298	DOWN	Cdc42	4:137319696-137357720	protein_coding	-
ENSMUSG00000039431.16	70,9890234	-1,613104	0,65058847	0,00048586	0,01008582	DOWN	Mtmr7	8:40551095-40634797	protein_coding	-
ENSMUSG00000087265.1	23,0526347	-3,8799694	1,37829253	0,00048829	0,0101204	DOWN	Gm12349	11:100291671-100293711	lincRNA	+
ENSMUSG00000041180.13	144,862822	-1,1769567	0,45166766	0,00049946	0,01028403	DOWN	Hectd2	19:36554639-36621135	protein_coding	+
ENSMUSG00000055044.12	198,840602	-1,0137701	0,37472899	0,00050058	0,01028403	DOWN	Pdlim1	19:40221173-40271842	protein_coding	-
ENSMUSG00000037341.13	215,8777	-0,998071	0,36816713	0,00049883	0,01028403	DOWN	Slc9a7	X:20105755-20291807	protein_coding	-
ENSMUSG00000022336.2	1714,67316	-0,670335	0,22787794	0,00050085	0,01028403	DOWN	Eif3e	15:43250040-43282736	protein_coding	-
ENSMUSG00000027858.13	204,387165	-1,1545864	0,45369073	0,00052747	0,01074737	DOWN	Tspan2	3:102734529-102801513	protein_coding	+
ENSMUSG00000032281.11	1673,29162	-0,8121422	0,29955155	0,0005308	0,01079855	DOWN	Acsbg1	9:54604877-54661870	protein_coding	-
ENSMUSG00000000142.15	880,607437	-0,823033	0,3008547	0,00054519	0,01102373	DOWN	Axin2	11:108920349-108950783	protein_coding	+
ENSMUSG00000026701.15	915,042956	-0,740787	0,26136333	0,0005491	0,01108585	DOWN	Prdx6	1:161240112-161251219	protein_coding	-
ENSMUSG00000044177.4	82,5839556	-1,686743	0,84696171	0,00055077	0,01110249	DOWN	Wfikkn2	11:94235956-94246005	protein_coding	-
ENSMUSG00000103358.1	69,5868689	-1,5308798	0,61097623	0,0005576	0,01119336	DOWN	Gm37593	17:51842861-51846114	TEC	+
ENSMUSG00000028668.5	2028,59209	-0,6705264	0,23046	0,00058044	0,01155999	DOWN	Tceb3	4:136003368-136021763	protein_coding	-
ENSMUSG00000015619.10	794,235501	-0,7138831	0,25028139	0,00058404	0,01161423	DOWN	Gata3	2:9857078-9890034	protein_coding	-
ENSMUSG00000076745.4	20,16913	-4,5526265	1,61331567	0,00059583	0,01179545	DOWN	Tcrg-V4	13:19184974-19185506	TR_V_gene	+
ENSMUSG00000030792.7	114,378202	-1,3079308	0,53664556	0,00060638	0,01195073	DOWN	Dkkl1	7:45207525-45211883	protein_coding	-
ENSMUSG00000050029.7	782,175443	-0,7413636	0,26598904	0,00061949	0,01217296	DOWN	Rap2c	X:51003912-51018018	protein_coding	-
ENSMUSG00000028937.14	677,329485	-0,7336958	0,2602501	0,00062675	0,01229726	DOWN	Acot7	4:152178134-152271855	protein_coding	+
ENSMUSG00000030220.13	6436,14062	-0,6046279	0,20704296	0,00063432	0,0124091	DOWN	Arhgdib	6:136923655-136941899	protein_coding	-
ENSMUSG00000034868.8	2253,42595	-0,6279621	0,21709054	0,00064748	0,01262915	DOWN	Myl12b	17:70973920-70990787	protein_coding	-
ENSMUSG00000027712.13	710,113722	-0,723623	0,25839395	0,00065186	0,01265868	DOWN	Anxa5	3:36448923-36475894	protein_coding	-
ENSMUSG00000076469.3	178,679117	-1,0588068	0,41167196	0,00066206	0,01281926	DOWN	Trbv13-2	6:41121396-41121832	TR_V_gene	+
ENSMUSG00000106620.1	13,3168824	-0,267727	0,51486564	0,00067154	0,01296477	DOWN	Trav7-5	14:53530786-53531313	TR_V_gene	+
ENSMUSG00000040760.10	1333,93943	-0,6882484	0,24693684	0,00068613	0,01320794	DOWN	Appl1	14:26918988-26971232	protein_coding	-
ENSMUSG00000059325.14	842,355162	-0,6988096	0,24994468	0,00070303	0,01347448	DOWN	Hopx	5:77086988-77115121	protein_coding	-
ENSMUSG00000040322.10	1504,66974	-0,6443023	0,22756826	0,00074392	0,01413557	DOWN	Slc25a24	3:109123149-109168457	protein_coding	+
ENSMUSG00000026655.14	5946,40614	-0,5900841	0,2050281	0,00075143	0,0142373	DOWN	Fam107b	2:3613758-3782142	protein_coding	+
ENSMUSG00000018846.8	1002,66045	-0,7108347	0,258323	0,00076373	0,01444976	DOWN	Pank3	11:35769484-35791285	protein_coding	+
ENSMUSG00000032301.13	791,733298	-0,6925834	0,25033585	0,00077125	0,01457121	DOWN	Psma4	9:54950790-54958030	protein_coding	+
ENSMUSG00000045973.18	1098,5012	-0,6639938	0,23742315	0,00077362	0,01459525	DOWN	Slc25a51	4:45395923-45408766	protein_coding	-
ENSMUSG00000021716.14	239,013303	-0,9076846	0,34980803	0,0008037	0,01507676	DOWN	Srek1ip1	13:104792484-104839274	protein_coding	+
ENSMUSG00000032018.13	445,85103	-0,7647761	0,28459458	0,00082159	0,0153353	DOWN	Sc5d	9:42254177-42264300	protein_coding	-
ENSMUSG00000041849.7	360,92276	-0,841485	0,32479124	0,00085533	0,0158083	DOWN	Card6	15:5095981-5108539	protein_coding	-
ENSMUSG00000021676.9	3984,68315	-0,6098219	0,21636729	0,00085583	0,0158083	DOWN	Iqgap2	13:95627177-95891922	protein_coding	-
ENSMUSG00000071528.3	237,332006	-0,917444	0,36264485	0,00087416	0,01610209	DOWN	Usmg5	19:47083471-47090625	protein_coding	-
ENSMUSG00000036309.14	656,831735	-0,7313458	0,27274021	0,00087662	0,01612492	DOWN	Skp1a	11:52231995-52246858	protein_coding	+
ENSMUSG00000042063.11	592,665837	-0,7249851	0,26905646	0,00088158	0,01619368	DOWN	Zfp386	12:116047724-116063360	protein_coding	+
ENSMUSG00000042312.9	597,047144	-0,7636057	0,28937518	0,00088936	0,01631399	DOWN	S100a13	3:90514435-90524581	protein_coding	+
ENSMUSG00000056737.14	894,043191	-0,7170048	0,26734598	0,00093211	0,016934	DOWN	Capg	6:72544391-72562983	protein_coding	+
ENSMUSG00000028221.3	187,906071	-0,9633288	0,38652363	0,00093736	0,01700601	DOWN	Tmem55a	4:14864076-14915176	protein_coding	+
ENSMUSG00000048264.15	94,5804632	-1,2219858	0,51949683	0,00094766	0,01711656	DOWN	Dip2c	13:9276528-9668928	protein_coding	+
ENSMUSG00000031129.9	865,952571	-0,6695834	0,24554167	0,0009477	0,01711656	DOWN	Slc9a9	9:94669909-95230445	protein_coding	+
ENSMUSG00000075595.9	1638,73838	-0,6578847	0,24199676	0,00095921	0,01726053	DOWN	Zfp652	11:95712673-95835115	protein_coding	+
ENSMUSG00000008682.13	3358,56019	-0,5887344	0,21103778	0,00100111	0,01796571	DOWN	Rpl10	X:74270812-74273135	protein_coding	+
ENSMUSG00000050075.8	716,891702	-0,7061843	0,26512481	0,00101344	0,01813776	DOWN	Gpr171	3:59096448-59101821	protein_coding	-
ENSMUSG00000036438.12	1042,12238	-0,6718045	0,24992001	0,00102085	0,01824576	DOWN	Calm2	17:87433412-87446935	protein_coding	-
ENSMUSG00000020393.16	409,129831	-0,7659348	0,29530605	0,00104507	0,01857846	DOWN	Kremen1	11:5191552-5261558	protein_coding	-
ENSMUSG00000090733.6	2518,84398	-0,6136519	0,2293992	0,00105497	0,01872931	DOWN	Rps27	3:90212522-90213651	protein_coding	-
ENSMUSG00000052374.14	223,550769	-0,4096609	0,95202739	0,00108291	0,01914327	DOWN	Actn2	13:12269426-12340760	protein_coding	-
ENSMUSG00000105708.1	12,6476804	-0,2349146	0,46140646	0,00108552	0,01914327	DOWN	Gm36070	3:94356507-94360842	processed_transcript	-
ENSMUSG00000022416.15	195,84287	-0,9412356	0,38368569	0,0010841	0,01914327	DOWN	Cacna1i	15:80287238-80398279	protein_coding	+
ENSMUSG00000021262.14	599,533316	-0,7117872	0,2713758	0,00108336	0,01914327	DOWN	Evl	12:108554720-108688513	protein_coding	+
ENSMUSG00000087066.1	28,4561982	-2,3741682	1,12833043	0,00109339	0,01925643	DOWN	Gm15518	16:49758576-49798870	antisense	-
ENSMUSG00000002996.17	662,868527	-0,6848797	0,25839508	0,00113973	0,01988746	DOWN	Hbp1	12:31926254-31950535	protein_coding	-
ENSMUSG00000031397.11	27,1372172	-2,972502	1,28102474	0,00115095	0,02003238	DOWN	Tktl1	X:74177259-74208500	protein_coding	+
ENSMUSG00000030103.11	7557,11614	-0,5604672	0,20248765	0,00117199	0,020321	DOWN	Bhlhe40	6:108660629-108666925	protein_coding	+
ENSMUSG00000027508.15	2073,02838	-0,6227013	0,23247617	0,00117535	0,02034243	DOWN	Pag1	3:9687479-9833679	protein_coding	-
ENSMUSG00000034252.14	2013,19261	-0,5979338	0,21872967	0,00117656	0,02034243	DOWN	Senp6	9:80066903-80144953	protein_coding	+
ENSMUSG00000047996.16	112,769928	-1,139978	0,49547007	0,00118905	0,02053159	DOWN	Prrg1	X:78449613-78583896	protein_coding	-
ENSMUSG00000025781.14	1390,97376	-0,6191861	0,23022167	0,00120318	0,02074866	DOWN	Atp5c1	2:10056016-10080510	protein_coding	-
ENSMUSG00000045087.8	77,0444707	-1,3096959	0,59488489	0,00121186	0,02081688	DOWN	S1pr5	9:21242912-21248443	protein_coding	-
ENSMUSG00000035578.15	38,2792959	-1,8837069	0,87861547	0,00121939	0,02086504	DOWN	Iqcg	16:33015385-33056218	protein_coding	-

ENSMUSG00000051359.14	601,867142	-0,7177095	0,27925576	0,00122465	0,02092803	DOWN	Ncald	15:37366175-37792570	protein_coding	-
ENSMUSG00000061411.12	58,584125	-1,5093195	0,69846476	0,00122962	0,02098601	DOWN	Nol4l	2:153407462-153529971	protein_coding	-
ENSMUSG00000030149.15	245,943738	-0,9735255	0,42846802	0,00124313	0,02116191	DOWN	Klrk1	6:129610323-129623864	protein_coding	-
ENSMUSG00000044026.2	457,624405	-0,7241081	0,28300223	0,00128345	0,02173664	DOWN	Slc35g1	19:38395980-38405607	protein_coding	+
ENSMUSG00000094655.1	1325,0995	-0,6294154	0,23768203	0,00128929	0,02179922	DOWN	Gm25360	1:72226240-72226430	snRNA	+
ENSMUSG00000047187.9	814,424464	-0,6538122	0,24918131	0,00130129	0,02189867	DOWN	Rab2a	4:8535644-8607778	protein_coding	+
ENSMUSG00000019907.9	4447,01539	-0,5673224	0,20862071	0,00132686	0,02224416	DOWN	Ppp1r12a	10:108162400-108277575	protein_coding	+
ENSMUSG00000073616.10	264,683454	-0,8331604	0,34048365	0,00134313	0,0223769	DOWN	Myeov2	1:92637145-92641985	protein_coding	-
ENSMUSG00000025314.16	836,332778	-0,6565545	0,2536698	0,00134998	0,0223769	DOWN	Ptprj	2:90429754-90580647	protein_coding	-
ENSMUSG00000021556.11	1733,80153	-0,6176712	0,23404686	0,00134975	0,0223769	DOWN	Golm1	13:59634996-59675784	protein_coding	-
ENSMUSG00000066621.12	3964,22432	-0,6292482	0,24113838	0,00138011	0,02284778	DOWN	Tecpr1	5:144194442-144223615	protein_coding	-
ENSMUSG00000025287.15	579,825858	-0,6897034	0,26887042	0,00138186	0,02284815	DOWN	Acot9	X:155262443-155297654	protein_coding	+
ENSMUSG00000087141.1	565,02244	-0,6868626	0,26796299	0,00139682	0,02305813	DOWN	Plcxd2	16:45959263-46010218	protein_coding	-
ENSMUSG00000040653.5	9,94684132	-0,1778891	0,39671273	0,00141022	0,02322999	DOWN	Ppp1r14c	10:3366150-3464975	protein_coding	+
ENSMUSG00000025903.14	668,988905	-0,6679345	0,26048236	0,00146928	0,02392109	DOWN	Lypla1	1:4807788-4848410	protein_coding	+
ENSMUSG00000021917.14	404,96877	-0,7671289	0,31418717	0,0015083	0,02439065	DOWN	Spcs1	14:30999826-31001672	protein_coding	-
ENSMUSG00000025464.14	80,448844	-1,2304386	0,57188425	0,00152288	0,0245719	DOWN	Paox	7:140125657-140134334	protein_coding	+
ENSMUSG00000087260.5	282,296439	-0,8376068	0,35455959	0,00152322	0,0245719	DOWN	Lamtor5	3:107278858-107284082	protein_coding	+
ENSMUSG00000042655.4	21,9043011	-3,366788	1,45762452	0,00153241	0,02469002	DOWN	Fam159b	13:104845283-104863893	protein_coding	-
ENSMUSG00000038028.9	281,111852	-0,7951751	0,32794475	0,00153974	0,02471796	DOWN	Tigar	6:127085116-127109557	protein_coding	-
ENSMUSG00000032561.14	474,917106	-0,718235	0,28818774	0,00155156	0,0247969	DOWN	Acpp	9:104288316-104337728	protein_coding	-
ENSMUSG00000073643.11	618,915531	-0,6646382	0,26063844	0,0015497	0,0247969	DOWN	Wdfy1	1:79702262-79776143	protein_coding	-
ENSMUSG00000096210.1	839,702216	-0,6620444	0,26058343	0,00155338	0,0247969	DOWN	H1f0	15:79028212-79030498	protein_coding	+
ENSMUSG00000024670.16	4046,74753	-0,5549097	0,20765566	0,00155166	0,0247969	DOWN	Cd6	19:10789341-10830058	protein_coding	-
ENSMUSG00000038128.6	2546,22395	-0,5651484	0,21319838	0,00157221	0,02505694	DOWN	Camk4	18:32939041-33195767	protein_coding	+
ENSMUSG00000041841.7	1380,28736	-0,6067415	0,23518426	0,00157623	0,02506079	DOWN	Rpl37	15:5116613-5119140	protein_coding	+
ENSMUSG00000024816.12	1995,15453	-0,6176942	0,24001665	0,00158257	0,02507121	DOWN	Frmd8	19:5849702-5875274	protein_coding	-
ENSMUSG00000028277.13	1288,87095	-0,6232676	0,24159995	0,00161335	0,02543721	DOWN	Ube2j1	4:33031416-33052363	protein_coding	+
ENSMUSG00000064023.4	240,718115	-0,9271477	0,42061422	0,00161569	0,02544372	DOWN	Klk8	7:43797577-43803826	protein_coding	+
ENSMUSG00000032228.16	1487,23743	-0,5895	0,22614534	0,00162778	0,02560377	DOWN	Tcf12	9:71842688-72111871	protein_coding	-
ENSMUSG00000028803.18	2361,59168	-0,6359986	0,2511275	0,00163187	0,02563761	DOWN	Nipal3	4:135445420-135495038	protein_coding	-
ENSMUSG00000094766.3	22,1995128	-2,2887234	1,18543592	0,00168031	0,0262121	DOWN	Trav7-4	14:53461099-53461738	TR_V_gene	+
ENSMUSG00000038633.5	940,560095	-0,6766445	0,27260832	0,00168306	0,02622402	DOWN	Degs1	1:182275772-182282804	protein_coding	-
ENSMUSG00000018819.10	9687,40599	-0,6123087	0,24153847	0,00170565	0,02651365	DOWN	Lsp1	7:142460809-142494867	protein_coding	+
ENSMUSG00000102561.1	199,254913	-0,8730658	0,37885621	0,00172181	0,02673348	DOWN	Gm37039	1:60989479-60991963	TEC	+
ENSMUSG00000040929.16	323,448266	-0,7768945	0,32604727	0,00173486	0,02684168	DOWN	Rfx3	19:27761721-28011166	protein_coding	-
ENSMUSG00000001281.9	9422,08858	-0,586032	0,22803773	0,00175144	0,02706652	DOWN	Itgb7	15:102215995-102231935	protein_coding	-
ENSMUSG00000090100.7	289,148305	-0,7779483	0,32619428	0,0017586	0,02711401	DOWN	Ttbk2	2:120732816-120850604	protein_coding	-
ENSMUSG00000050229.3	1166,81493	-0,597121	0,23207157	0,00177176	0,02725346	DOWN	Pigm	1:172376546-172384099	protein_coding	+
ENSMUSG00000054404.12	367,790396	-0,8205946	0,36008285	0,00179367	0,02752223	DOWN	Slfn5	11:82951349-82962941	protein_coding	+
ENSMUSG00000045414.7	330,926756	-0,7592408	0,31753986	0,00180194	0,02755755	DOWN	1190002N15	9:94517864-94538081	protein_coding	-
ENSMUSG00000049044.16	86,6578308	-1,2997868	0,70489201	0,00181543	0,02768852	DOWN	Rapgef4	2:71981240-72257474	protein_coding	+
ENSMUSG00000064345.1	2919,78735	-0,5784721	0,22407552	0,00182083	0,02768852	DOWN	mt-Nd2	MT:3914-4951	protein_coding	+
ENSMUSG00000006169.19	2733,52818	-0,5598002	0,21237012	0,00183806	0,02788454	DOWN	Clint1	11:45852051-45910625	protein_coding	+
ENSMUSG00000046311.13	1037,54972	-0,6173537	0,24427895	0,00185745	0,02811438	DOWN	Zfp62	11:49203292-49218816	protein_coding	+
ENSMUSG00000014313.14	728,365286	-0,6387369	0,25436143	0,00186741	0,02818007	DOWN	Cox6c	15:35925886-35938246	protein_coding	-
ENSMUSG00000024480.7	374,956	-0,7197596	0,29793754	0,00188825	0,02840611	DOWN	Ap3s1	18:46741917-46790826	protein_coding	+
ENSMUSG00000023048.13	1713,47531	-0,5817609	0,22623573	0,00190517	0,02857544	DOWN	Prr13	15:102459028-102462806	protein_coding	+
ENSMUSG00000015575.14	465,025765	-0,6931901	0,28632861	0,00196137	0,0293187	DOWN	Atp6v0e	17:26676396-26699644	protein_coding	+
ENSMUSG00000032011.4	5570,22609	-0,5334118	0,20676603	0,00197362	0,02940223	DOWN	Thy1	9:44043384-44048579	protein_coding	+
ENSMUSG00000096908.3	22,0142062	-0,3080825	0,57882909	0,00199278	0,02961571	DOWN	Trav7-3	14:53443249-53443839	TR_V_gene	+
ENSMUSG00000026069.15	578,445047	-0,7774683	0,34622024	0,00200368	0,02968319	DOWN	Il1rl1	1:40429570-40465415	protein_coding	+
ENSMUSG00000030110.13	62,2159116	-1,345131	0,69704101	0,00202735	0,0299	DOWN	Ret	6:118151745-118197718	protein_coding	-
ENSMUSG00000031902.9	4845,5366	-0,5341232	0,20550683	0,00208575	0,0306589	DOWN	Nfatc3	8:106059603-106130537	protein_coding	+
ENSMUSG00000084803.8	56,5332047	-1,3784219	0,71412071	0,00214349	0,03143792	DOWN	5830444B04	4:155398763-155421704	processed_transcript	-
ENSMUSG00000037463.14	81,3364098	-1,2263243	0,65066937	0,00219465	0,03208164	DOWN	Fbxo27	7:28692849-28699338	protein_coding	+
ENSMUSG00000040212.11	1133,56613	-0,5881306	0,23510992	0,00221879	0,03229922	DOWN	Emp3	7:45918023-45921426	protein_coding	-
ENSMUSG00000038024.17	1798,32845	-0,5579649	0,21944372	0,00221929	0,03229922	DOWN	Dennd4c	4:86748555-86850603	protein_coding	+
ENSMUSG00000024725.12	1999,21627	-0,5520818	0,21724639	0,00225882	0,03273066	DOWN	Ostf1	19:18579328-18631789	protein_coding	-
ENSMUSG00000050147.8	200,977493	-0,8307643	0,37290294	0,0022743	0,03284715	DOWN	F2rl3	8:72761880-72763874	protein_coding	+
ENSMUSG00000021236.16	933,357089	-0,6005548	0,24222484	0,00227917	0,03288164	DOWN	Entpd5	12:84373857-84409029	protein_coding	-
ENSMUSG00000028943.18	91,5840247	-1,1718214	0,6337149	0,00229362	0,03298233	DOWN	Espn	4:152120331-152152371	protein_coding	-
ENSMUSG00000079298.9	26,4681595	-1,9428965	1,12595922	0,00231927	0,03313538	DOWN	Klrb1b	6:128813706-128826331	protein_coding	-
ENSMUSG00000035258.14	16,6166489	-0,4113713	1,00188406	0,00231019	0,03313538	DOWN	Abi3bp	16:56477846-56690128	protein_coding	+
ENSMUSG00000000732.8	367,750192	-0,7245114	0,31292873	0,00231609	0,03313538	DOWN	Icosl	10:78069360-78079525	protein_coding	+
ENSMUSG00000064351.1	36773,9485	-0,5399525	0,20864409	0,00236196	0,03363634	DOWN	mt-Co1	MT:5328-6872	protein_coding	+
ENSMUSG00000025289.15	194,964284	-0,8560821	0,39310254	0,00236464	0,03363834	DOWN	Prdx4	X:155323918-155340754	protein_coding	-
ENSMUSG00000032330.6	491,138713	-0,6663292	0,27891564	0,00238749	0,03385434	DOWN	Cox7a2	9:79755241-79759853	protein_coding	-
ENSMUSG00000021879.12	20,8023644	-2,2557612	1,33753843	0,00245285	0,03466973	DOWN	Dnah12	14:26693274-26891703	protein_coding	+
ENSMUSG00000027500.10	18,9356085	-0,3569585	0,71588429	0,00247326	0,03492096	DOWN	Stmn2	3:8509360-8561606	protein_coding	+
ENSMUSG00000025868.6	218,689442	-0,8235852	0,37838247	0,00250346	0,03527213	DOWN	Higd2a	13:54590207-54591158	protein_coding	+
ENSMUSG00000024513.16	2129,75117	-0,5577648	0,22401238	0,00251351	0,0353011	DOWN	Mbd2	18:70568189-70626131	protein_coding	+
ENSMUSG00000023274.14	7925,86023	-0,5142352	0,20113567	0,0025101	0,0353011	DOWN	Cd4	6:124864692-124888221	protein_coding	-
ENSMUSG00000054493.2	14,0102558	-0,3188632	0,62504216	0,00253747	0,03556219	DOWN	Gm9947	1:14752937-14776931	antisense	+
ENSMUSG00000032376.12	1330,83208	-0,5610382	0,2267349	0,00254497	0,03562962	DOWN	Usp3	9:66514637-66593142	protein_coding	-
ENSMUSG00000029530.15	109,380448	-1,1124494	0,84190679	0,00254833	0,03563896	DOWN	Ccr9	9:123678439-123783457	protein_coding	+
ENSMUSG00000033186.8	509,936718	-0,651386	0,27513132	0,00257034	0,0359089	DOWN	Mzt1	14:99034544-99046136	protein_coding	-
ENSMUSG00000031950.7	386,519406	-0,689231	0,29708276	0,00257573	0,03592021	DOWN	Gabarapl2	8:111940703-111953612	protein_coding	+
ENSMUSG00000021076.5	535,344813	-0,6688205	0,2877055	0,00261163	0,03637058	DOWN	Actr10	12:70937857-70964718	protein_coding	+
ENSMUSG00000000581.8	338,236038	-0,7110896	0,31102564	0,00263938	0,03671839	DOWN	C1d	11:17257579-17269176	protein_coding	+
ENSMUSG00000020038.9	781,217378	-0,6232888	0,26255719	0,00265605	0,03687286	DOWN	Cry1	10:85131702-85185054	protein_coding	-
ENSMUSG00000021302.9	371,683379	-0,688963	0,29964622	0,00268009	0,03716767	DOWN	Ggps1	13:14052449-14063401	protein_coding	-
ENSMUSG00000071253.8	261,152629	-0,7694063	0,3522598	0,00279721	0,03842967	DOWN	Slc25a16	10:62920633-62946498	protein_coding	+
ENSMUSG00000042348.9	448,703992	-0,6571165	0,2823463	0,0027902	0,03842967	DOWN	Arl15	13:113794505-114157461	protein_coding	+

ENSMUSG00000031197.11	534,464972	-0,6509116	0,28061866	0,00279476	0,03842967	DOWN	Vbp1	X:75514299-75534942	protein_coding	+
ENSMUSG00000064363.1	1924,09148	-0,5944819	0,25066067	0,00282852	0,03877945	DOWN	mt-Nd4	MT:10167-11544	protein_coding	+
ENSMUSG00000008333.12	480,908073	-0,6497925	0,2792475	0,0028321	0,03878834	DOWN	Snrpb2	2:143063039-143072853	protein_coding	+
ENSMUSG00000044734.15	614,648899	-0,7801538	0,38534675	0,0028442	0,03891389	DOWN	Serpinb1a	13:32842092-32851185	protein_coding	-
ENSMUSG00000003429.10	4529,6076	-0,5496623	0,22470714	0,00285287	0,03899211	DOWN	Rps11	7:45122388-45124389	protein_coding	-
ENSMUSG00000036751.7	748,29576	-0,6088851	0,25585896	0,00286543	0,03912343	DOWN	Cox6b1	7:30616861-30626151	protein_coding	-
ENSMUSG00000024539.17	679,533448	-0,6064879	0,25577391	0,0029449	0,04000234	DOWN	Ptpn2	18:67665511-67724595	protein_coding	-
ENSMUSG00000035275.14	109,126382	-0,9631012	0,48562603	0,00297248	0,04005018	DOWN	Raver2	4:101068983-101152370	protein_coding	+
ENSMUSG00000039168.15	804,873828	-0,5952505	0,2506295	0,00295489	0,04005018	DOWN	Dap	15:31224314-31274341	protein_coding	+
ENSMUSG00000016319.3	2015,14457	-0,5367811	0,21829104	0,00297274	0,04005018	DOWN	Slc25a5	X:36795651-36798807	protein_coding	+
ENSMUSG00000026107.11	3733,15999	-0,527265	0,21240049	0,00297167	0,04005018	DOWN	Nabp1	1:51465862-51478425	protein_coding	-
ENSMUSG00000029672.16	759,022895	-0,6173868	0,26626838	0,00308269	0,0411149	DOWN	Fam3c	6:22306520-22356243	protein_coding	-
ENSMUSG00000037071.2	16,3747351	-0,340866	0,66869791	0,00309074	0,04118091	DOWN	Scd1	19:44394451-44407709	protein_coding	-
ENSMUSG00000028362.2	307,539041	-0,7812119	0,37977275	0,00310341	0,04130805	DOWN	Tnfsf8	4:63831308-63861347	protein_coding	-
ENSMUSG00000020250.9	1903,21372	-0,5347648	0,21885403	0,00311184	0,04137878	DOWN	Txnrd1	10:82859206-82897724	protein_coding	+
ENSMUSG00000000346.8	2203,76633	-0,549203	0,22661137	0,00313582	0,04165577	DOWN	Dazap2	15:100615628-100620731	protein_coding	+
ENSMUSG00000014867.9	2302,91025	-0,5252645	0,21383155	0,00315151	0,0418222	DOWN	Surf4	2:26920040-26933928	protein_coding	-
ENSMUSG00000079845.8	219,997398	-0,8307417	0,41660257	0,00319822	0,04231496	DOWN	Xlr4a	X:73074345-73082478	protein_coding	-
ENSMUSG00000066798.3	340,374535	-0,6953937	0,31329228	0,00319649	0,04231496	DOWN	Zbtb6	2:37425500-37443171	protein_coding	-
ENSMUSG00000102600.1	44,1480901	-1,3916306	0,89969034	0,00325371	0,04300614	DOWN	Gm37266	17:51850239-51852525	TEC	+
ENSMUSG00000001248.14	3582,34218	-0,6282588	0,28439426	0,00328814	0,04335908	DOWN	Gramd1a	7:31130127-31155896	protein_coding	-
ENSMUSG00000020732.13	560,489744	-0,6263215	0,27556766	0,00329024	0,04335908	DOWN	Rab37	11:115091431-115162236	protein_coding	+
ENSMUSG00000076498.2	3117,39641	-0,5292596	0,21874015	0,00332128	0,04368132	DOWN	Trbc2	6:41546730-41548352	TR_C_gene	+
ENSMUSG00000055447.18	4190,7585	-0,5531892	0,22947228	0,00334601	0,04391932	DOWN	Cd47	16:49855618-49915010	protein_coding	+
ENSMUSG00000022015.8	84,2675182	-1,0774274	0,61149913	0,00336468	0,044077	DOWN	Tnfsf11	14:78277445-78308043	protein_coding	-
ENSMUSG00000062006.12	2091,55816	-0,5259144	0,21651837	0,00338234	0,04426448	DOWN	Rpl34	3:130726831-130730398	protein_coding	-
ENSMUSG00000040537.17	55,7331259	-1,2428607	0,72454344	0,00341945	0,0446619	DOWN	Adam22	5:8072352-8368160	protein_coding	-
ENSMUSG00000023921.8	194,200486	-0,8011372	0,39163629	0,00348287	0,04530465	DOWN	Mut	17:40934685-40961989	protein_coding	+
ENSMUSG00000036299.4	287,210416	-0,7072894	0,32600142	0,0034934	0,04531928	DOWN	BC031181	18:75005900-75009933	protein_coding	+
ENSMUSG00000016756.16	1186,66917	-0,5575709	0,23556467	0,00349373	0,04531928	DOWN	Cmah	13:24327420-24477285	protein_coding	+
ENSMUSG00000027835.11	522,128986	-0,6280779	0,27616276	0,00350388	0,04540647	DOWN	Pdcd10	3:75516490-75556856	protein_coding	-
ENSMUSG00000003031.14	1466,5628	-0,5399697	0,22792605	0,0035287	0,04559424	DOWN	Cdkn1b	6:134920401-134925513	protein_coding	+
ENSMUSG00000024677.13	5710,55033	-0,5010037	0,20548343	0,00355605	0,04590289	DOWN	Ms4a6b	19:11516512-11531256	protein_coding	+
ENSMUSG00000039521.12	16,4838144	-0,3394246	0,66059717	0,00359829	0,04635773	DOWN	Foxp3	X:7579676-7595243	protein_coding	+
ENSMUSG00000039001.12	1998,09493	-0,5213108	0,21637317	0,00361487	0,04648095	DOWN	Rps21	2:180257377-180258445	protein_coding	+
ENSMUSG00000040044.11	872,532374	-0,573711	0,24541108	0,00362678	0,04654364	DOWN	Orc3	4:34570796-34614944	protein_coding	-
ENSMUSG00000045165.6	1265,94537	-0,5453422	0,23089642	0,00369839	0,04732505	DOWN	AI467606	7:127091359-127093986	protein_coding	+
ENSMUSG00000073155.12	331,965549	-0,6786249	0,31201842	0,00372647	0,04760044	DOWN	1810058I24R	6:35252654-35263496	lincRNA	+
ENSMUSG00000019877.10	1088,58352	-0,5823067	0,25693305	0,00377905	0,04817101	DOWN	Serinc1	10:57515774-57532530	protein_coding	-
ENSMUSG00000032589.14	326,227141	-0,7636098	0,39013859	0,00378908	0,04825251	DOWN	Bsn	9:108096022-108190384	protein_coding	-
ENSMUSG00000055865.8	116,790522	-0,9059576	0,47832454	0,00387955	0,04930975	DOWN	Fam19a3	3:104767406-104781840	protein_coding	-
ENSMUSG00000032735.14	23,5175533	-1,7547655	1,23072916	0,00390359	0,0495203	DOWN	Ablim3	18:61799395-61911852	protein_coding	-
