# Supplementary material for: EOMES and IL-10 regulate antitumor activity of T regulatory type 1 CD4+ T cells in chronic lymphocytic leukemia
Source: Leukemia. 2021 Feb 1;35(8):2311–24. doi: 10.1038/s41375-021-01136-1 (PMC8324479; doi:10.1038/s41375-021-01136-1)
Supplement: Supplementary file 10 — Suppl. Table 6_sheet 2_comparison 2 [file 41375_2021_1136_MOESM10_ESM.pdf]

Comparison 2

ENSID	baseMean	log2FoldChange	lfcSE	pvalue	padj	Status	external_gene_name	gene_coordinates	gene_type	strand
ENSMUSG00000049410.8	294,4673543	5,185598355	0,516952299	4,22E-25	5,85E-21	UP	Zfp683	4:134053838-134058996	protein_coding	+
ENSMUSG00000040264.10	154,1895617	4,614349318	0,549309526	3,31E-18	2,29E-14	UP	Gbp2b	3:142594847-142619179	protein_coding	+
ENSMUSG00000045087.8	359,2312831	4,6628732	0,592241471	1,47E-16	6,79E-13	UP	S1pr5	9:21242912-21248443	protein_coding	-
ENSMUSG00000012123.15	239,5721177	3,497746725	0,451715021	4,39E-16	1,52E-12	UP	Aim1l	4:134065912-134092504	protein_coding	+
ENSMUSG00000078503.9	85,72228211	7,939975946	1,382947659	1,16E-08	1,46E-05	UP	Gm13225	4:145510759-145539188	protein_coding	+
ENSMUSG00000076479.3	47,00717193	5,189699961	0,972032493	1,12E-08	1,46E-05	UP	Trbv26	6:41227505-41227993	TR_V_gene	+
ENSMUSG00000025809.15	4196,177758	2,457510614	0,50194604	4,70E-08	4,65E-05	UP	Itgb1	8:128685654-128733200	protein_coding	+
ENSMUSG00000094344.1	24,43970945	7,900749157	2,770981369	1,01E-06	0,000609866	UP	Gm11942	4:32964888-32965232	processed_pseudogen	+
ENSMUSG00000026872.17	1373,724123	1,573655486	0,381947892	1,46E-06	0,000843423	UP	Zeb2	2:44983632-45117395	protein_coding	-
ENSMUSG00000030247.9	66,78265576	3,348067505	0,824481699	2,05E-06	0,001050771	UP	Kcnj8	6:142564837-142571614	protein_coding	-
ENSMUSG00000038146.7	85,93139859	2,724130715	0,687139854	2,46E-06	0,001218338	UP	Notch3	17:32120820-32166880	protein_coding	-
ENSMUSG00000074570.13	168,05276	1,888360759	0,496041537	5,08E-06	0,002271299	UP	Cass4	2:172393794-172433757	protein_coding	+
ENSMUSG00000030154.10	364,1985144	1,675827281	0,44399571	5,83E-06	0,002308077	UP	Klrb1f	6:129045901-129057464	protein_coding	+
ENSMUSG00000018341.12	866,7191376	1,531128847	0,403430399	5,49E-06	0,002308077	UP	Il12rb2	6:67291318-67376188	protein_coding	-
ENSMUSG00000030789.9	232,6714994	2,238960887	0,62940151	1,31E-05	0,004193041	UP	Itgax	7:128129547-128150657	protein_coding	+
ENSMUSG00000046623.8	15,9505479	7,008303664	2,776877301	1,81E-05	0,00557116	UP	Gjb4	4:127351086-127354081	protein_coding	-
ENSMUSG00000106620.1	16,03907843	7,012822117	2,785042329	1,91E-05	0,005735571	UP	Trav7-5	14:53530786-53531313	TR_V_gene	+
ENSMUSG00000046152.16	30,25281676	3,466851435	0,973028516	1,95E-05	0,005735571	UP	Fut10	8:31187331-31261738	protein_coding	+
ENSMUSG00000052336.6	919,8638222	1,885602523	0,549581392	2,07E-05	0,005986227	UP	Cx3cr1	9:119901616-120068283	protein_coding	-
ENSMUSG00000035275.14	126,3092858	1,894658338	0,555821148	2,19E-05	0,006204626	UP	Raver2	4:101068983-101152370	protein_coding	+
ENSMUSG00000087670.1	21,29263307	4,128900925	1,211322677	4,36E-05	0,011044499	UP	9530036M11Rik	6:54146887-54150937	antisense	-
ENSMUSG00000099397.1	45,22980639	2,35567818	0,740892559	5,15E-05	0,01208903	UP	Gm7809	17:21967501-21968242	processed_pseudogen	-
ENSMUSG00000094786.1	25,12101296	3,552958359	1,098405771	6,66E-05	0,014413415	UP	Gm14403	2:177498215-177509763	protein_coding	+
ENSMUSG00000015709.9	142,7494731	1,615598498	0,523599036	6,56E-05	0,014413415	UP	Arnt2	7:84246278-84410176	protein_coding	-
ENSMUSG00000048534.6	214,0377191	1,461327067	0,473083448	6,54E-05	0,014413415	UP	Amica1	9:45079183-45108530	protein_coding	+
ENSMUSG00000003545.3	1161,829572	1,252860571	0,417189566	8,68E-05	0,017949502	UP	Fosb	7:19302696-19310051	protein_coding	-
ENSMUSG00000105703.1	287,5788478	1,565392303	0,541790344	0,000116222	0,022686154	UP	Gm43305	14:54292443-54297343	TEC	-
ENSMUSG00000087775.1	19,5202338	5,431475726	1,580366625	0,00011889	0,022884765	UP	Rprl2	3:22251374-22251663	ribozyme	+
ENSMUSG00000004633.17	306,3088267	1,361712672	0,476718828	0,000131568	0,024978175	UP	Chn2	6:54039554-54301810	protein_coding	+
ENSMUSG00000104876.1	25,4797631	5,70427491	1,780936358	0,00016496	0,029193156	UP	Trdc	14:54142851-54148868	TR_C_gene	+
ENSMUSG00000039521.12	25,828265	4,192510025	1,464524708	0,000171139	0,029647652	UP	Foxp3	X:7579676-7595243	protein_coding	+
ENSMUSG00000032666.16	183,4535432	1,427850921	0,517380263	0,000174394	0,029652956	UP	1700025G04Rik	1:151852403-152090125	protein_coding	-
ENSMUSG00000039410.16	56,31347904	1,861562369	0,697791432	0,000229895	0,035799013	UP	Prdm16	4:154316125-154636873	protein_coding	-
ENSMUSG00000032216.14	387,4336361	1,170092773	0,448911845	0,000268779	0,039210638	UP	Nedd4	9:72662346-72749852	protein_coding	+
ENSMUSG00000036928.14	49,17417761	2,120850837	0,817894255	0,000280154	0,040444365	UP	Stag3	5:138280240-138312393	protein_coding	+
ENSMUSG00000079173.11	20,16190008	4,484247769	1,566253121	0,000314546	0,042968217	UP	Zan	5:137378637-137477064	protein_coding	-
ENSMUSG00000079491.9	826,2888445	1,094049062	0,433166526	0,000329379	0,043892891	UP	H2-T10	17:36115876-36121465	polymorphic_pseudoge	-
ENSMUSG00000076826.5	12,00003853	6,024229781	3,065153822	0,000384173	0,048846423	UP	Trav4-2	14:53418349-53418873	TR_V_gene	+
ENSMUSG00000073491.10	117,8109913	-3,723398473	0,613300249	5,66E-11	1,57E-07	DOWN	Pydc4	1:173566284-173599274	protein_coding	-
ENSMUSG00000002190.13	71,04649061	-4,045605357	0,671344999	9,64E-11	2,23E-07	DOWN	Clgn	8:83389867-83428552	protein_coding	+
ENSMUSG00000041272.11	985,4995857	-2,161906549	0,375994125	3,70E-10	7,32E-07	DOWN	Tox	4:6686353-6991557	protein_coding	-
ENSMUSG00000016529.5	112,5260836	-7,57E-06	0,001442704	5,40E-10	9,35E-07	DOWN	Il10	1:131019845-131024974	protein_coding	+
ENSMUSG00000006154.13	50,46114745	-5,655837284	1,033333706	8,18E-09	1,26E-05	DOWN	Eps8l1	7:4460674-4480487	protein_coding	+
ENSMUSG00000037849.7	130,7869907	-2,53766986	0,514471866	3,25E-08	3,75E-05	DOWN	Gm4955	1:173468509-173491041	protein_coding	-
ENSMUSG00000020183.11	147,1030981	-2,394373225	0,489571356	4,02E-08	4,28E-05	DOWN	Cpm	10:117629500-117687352	protein_coding	+
ENSMUSG00000004668.14	812,0341261	-2,682561598	0,55967123	6,22E-08	5,75E-05	DOWN	Abca13	11:9191942-9684259	protein_coding	+
ENSMUSG00000022900.14	163,0022328	-2,388891636	0,500042652	7,14E-08	6,19E-05	DOWN	Ildr1	16:36693978-36726804	protein_coding	+
ENSMUSG00000105987.4	456,6061197	-1,11E-05	0,001442711	1,86E-07	0,00015141	DOWN	AI506816	5:23698296-23712667	processed_transcript	-
ENSMUSG00000020846.6	181,9316059	-2,728704487	0,600353166	2,11E-07	0,000162693	DOWN	Fam101b	11:76019194-76027782	protein_coding	-
ENSMUSG00000053113.3	495,0868685	-1,973971809	0,437640045	2,45E-07	0,000178801	DOWN	Socs3	11:117966079-117970047	protein_coding	-
ENSMUSG00000026285.7	605,4210095	-1,734382018	0,405447181	7,07E-07	0,00048982	DOWN	Pdcd1	1:94038305-94052553	protein_coding	-
ENSMUSG00000027073.5	27,88307271	-8,115096591	2,866249024	8,41E-07	0,0005549	DOWN	Prg2	2:84980461-84983632	protein_coding	+
ENSMUSG00000030257.16	79,95891303	-2,999392567	0,711975192	9,55E-07	0,000601591	DOWN	Srgap3	6:112717971-112947266	protein_coding	-
ENSMUSG00000057329.7	776,5388913	-1,559565938	0,383844552	1,79E-06	0,000990746	DOWN	Bcl2	1:106538178-106714274	protein_coding	-
ENSMUSG00000098021.1	24,34802028	-3,55E-06	0,001442697	2,03E-06	0,001050771	DOWN	Gm9522	15:39180511-39181441	processed_pseudogen	+
ENSMUSG00000032549.7	23,21842099	-4,04E-06	0,001442698	3,11E-06	0,001486325	DOWN	Rab6b	9:103111787-103185276	protein_coding	+
ENSMUSG00000058297.16	140,2811761	-1,902697029	0,493815159	4,22E-06	0,001949156	DOWN	Spock2	10:60106219-60135198	protein_coding	+
ENSMUSG00000033066.15	487,7036949	-1,54782489	0,409231815	5,55E-06	0,002308077	DOWN	Gas7	11:67455437-67688990	protein_coding	+
ENSMUSG00000046841.4	239,9292099	-2,342197877	0,622634944	5,77E-06	0,002308077	DOWN	Ckap4	10:84526305-84534036	protein_coding	-
ENSMUSG00000030653.16	393,7111331	-1,02E-05	0,001442707	6,42E-06	0,002471514	DOWN	Pde2a	7:101421691-101512829	protein_coding	+
ENSMUSG00000022901.13	84,04250319	-2,276014061	0,612935477	7,45E-06	0,002790788	DOWN	Cd86	16:36603869-36666081	protein_coding	-
ENSMUSG00000042724.7	130,9320555	-2,31644103	0,638672508	9,86E-06	0,003597384	DOWN	Map3k9	12:81714950-81781170	protein_coding	-
ENSMUSG00000054672.12	60,20426029	-2,336917716	0,648199577	1,13E-05	0,004005211	DOWN	5830411N06Rik	7:140247301-140299791	protein_coding	+
ENSMUSG00000038357.10	1030,48061	-6,07E-06	0,001442699	1,17E-05	0,00404916	DOWN	Camp	9:109847379-109849617	protein_coding	-
ENSMUSG00000076490.6	1293,449488	-1,680537278	0,471551765	1,24E-05	0,004092469	DOWN	Trbc1	6:41538218-41539881	TR_C_gene	+
ENSMUSG00000000157.15	229,6230735	-2,177311134	0,610882338	1,23E-05	0,004092469	DOWN	Itgb2l	16:96422288-96443619	protein_coding	-
ENSMUSG00000024681.11	67,01878341	-3,507315787	0,988822776	1,33E-05	0,004193041	DOWN	Ms4a3	19:11629496-11640851	protein_coding	-
ENSMUSG00000052212.6	742,4761857	-5,79E-06	0,001442699	2,36E-05	0,006545036	DOWN	Cd177	7:24743983-24760311	protein_coding	-
ENSMUSG00000078122.4	130,0913087	-2,181350274	0,652473643	2,72E-05	0,007395875	DOWN	F630028O10Rik	X:96239926-96243636	antisense	+
ENSMUSG00000086513.3	98,82965145	-2,261096403	0,683569881	3,04E-05	0,008105663	DOWN	9130208D14Rik	7:105824223-105831515	unprocessed_pseudog	-
ENSMUSG00000064147.6	98,23242181	-1,912546666	0,587731721	3,74E-05	0,009776557	DOWN	Rab44	17:29135056-29148980	protein_coding	+
ENSMUSG00000025997.13	857,3444899	-1,550178183	0,485396131	4,49E-05	0,011044499	DOWN	Ikzf2	1:69531214-69687245	protein_coding	-
ENSMUSG00000032496.7	3147,475347	-1,909185874	0,605339653	4,54E-05	0,011044499	DOWN	Ltf	9:111019271-111042767	protein_coding	+
ENSMUSG00000031824.14	187,2132496	-2,045325426	0,640049034	4,41E-05	0,011044499	DOWN	6430548M08Rik	8:120114152-120165306	protein_coding	+
ENSMUSG00000003134.10	158,812535	-2,022107427	0,640666467	4,99E-05	0,011920023	DOWN	Tbc1d8	1:39371492-39478755	protein_coding	-
ENSMUSG00000026835.15	67,62312031	-2,669672829	0,85348409	5,54E-05	0,012795012	DOWN	Fcnb	2:28076378-28084885	protein_coding	-
ENSMUSG00000024164.15	698,45881	-1,780597045	0,580114816	6,56E-05	0,014413415	DOWN	C3	17:57203970-57228136	protein_coding	-
ENSMUSG00000000204.15	624,7306886	-1,354070801	0,441744631	6,87E-05	0,014640946	DOWN	Slfn4	11:83175186-83190221	protein_coding	+
ENSMUSG00000092517.1	61,48808005	-2,499893909	0,819301191	7,23E-05	0,015180346	DOWN	Art2a-ps	7:101552453-101560865	polymorphic_pseudoge	-
ENSMUSG00000047798.15	158,3491866	-1,943541186	0,654694122	9,00E-05	0,018333712	DOWN	Cd300lf	11:115116214-115133992	protein_coding	-
ENSMUSG00000091679.8	94,5582597	-1,804134373	0,612087637	9,80E-05	0,019639139	DOWN	Vmn2r96	17:18581727-18598157	protein_coding	+
ENSMUSG00000029086.15	49,39459651	-2,791170198	0,94838017	9,92E-05	0,019639139	DOWN	Prom1	5:43993620-44102032	protein_coding	-
ENSMUSG00000021457.14	276,6540265	-7,21E-06	0,0014427	0,000138815	0,025997825	DOWN	Syk	13:52583173-52648792	protein_coding	+
ENSMUSG00000051506.16	242,1840557	-6,47E-06	0,001442699	0,00014574	0,026710429	DOWN	Wdfy4	14:32959547-33185508	protein_coding	-
ENSMUSG00000052435.6	44,56117005	-2,683611167	0,955411304	0,000146475	0,026710429	DOWN	Cebpe	14:54710363-54712174	protein_coding	-
ENSMUSG00000100815.1	73,37313612	-1,97154278	0,710964763	0,000159861	0,028772971	DOWN	Gm29112	1:69595036-69598522	sense_intronic	-
ENSMUSG00000084350.1	19,35362759	-3,39E-06	0,001442696	0,000166409	0,029193156	DOWN	Znf41-ps	4:145825237-145829942	unprocessed_pseudog	+
ENSMUSG00000035004.3	141,3963614	-1,892741778	0,691706835	0,000175449	0,029652956	DOWN	Igsf6	7:121064067-121074572	protein_coding	-
ENSMUSG00000076846.3	17,72215085	-3,04E-06	0,001442696	0,000178901	0,029872235	DOWN	Trav13-2	14:53634829-53635399	TR_V_gene	+
ENSMUSG00000071552.4	612,6877405	-1,238977378	0,454662226	0,000187423	0,030922535	DOWN	Tigit	16:43648861-43664184	protein_coding	-
ENSMUSG00000015970.18	23,23296523	-3,05E-06	0,001442696	0,000191371	0,031202546	DOWN	Chdh	14:30009023-30040466	protein_coding	+

ENSMUSG00000038463.8	75,64616203	-2,462236514	0,915236383	0,000194731	0,031381076	DOWN	Olfml2b	1:170644532-170682789	protein_coding	+
ENSMUSG00000031722.9	493,6853723	-5,28E-06	0,001442698	0,000209012	0,033295386	DOWN	Hp	8:109575130-109579172	protein_coding	-
ENSMUSG00000025701.12	91,52994157	-1,970345411	0,736684612	0,000211558	0,033318053	DOWN	Alox5	6:116410077-116461178	protein_coding	-
ENSMUSG00000016200.13	22,00900994	-3,36E-06	0,001442696	0,000237802	0,036618814	DOWN	Syt14	1:192891233-193035775	protein_coding	-
ENSMUSG00000025348.8	36,87885886	-4,70E-06	0,001442697	0,000245551	0,037345494	DOWN	Itga7	10:128933813-128958277	protein_coding	+
ENSMUSG00000059901.12	41,93680324	-2,338275796	0,89163908	0,00024791	0,037345494	DOWN	Adamts14	10:61197112-61273438	protein_coding	-
ENSMUSG00000068747.14	165,8175497	-4,99E-06	0,001442697	0,000251397	0,037463566	DOWN	Sort1	3:108284082-108361511	protein_coding	+
ENSMUSG00000002111.8	142,6062964	-5,37E-06	0,001442697	0,00025892	0,038174131	DOWN	Spi1	2:91082390-91115756	protein_coding	+
ENSMUSG00000043740.14	97,71062258	-1,847899767	0,722149591	0,000286308	0,040906659	DOWN	B430306N03Rik	17:48316141-48327024	protein_coding	+
ENSMUSG00000031825.16	73,84259987	-1,857666577	0,729542162	0,000299416	0,042342888	DOWN	Crispld2	8:119992438-120052793	protein_coding	+
ENSMUSG00000037095.7	179,351581	-5,04E-06	0,001442697	0,00030846	0,042822064	DOWN	Lrg1	17:56119680-56121946	protein_coding	-
ENSMUSG00000029455.14	199,9647438	-5,88E-06	0,001442698	0,000308984	0,042822064	DOWN	Aldh2	5:121566027-121593824	protein_coding	-
ENSMUSG00000049608.8	30,29663432	-3,50E-06	0,001442696	0,00031934	0,042968217	DOWN	Gpr55	1:85938318-85961007	protein_coding	-
ENSMUSG00000026579.8	82,62196919	-2,06491667	0,822417701	0,0003173	0,042968217	DOWN	F5	1:164151838-164220277	protein_coding	+
ENSMUSG00000026011.13	753,4348695	-7,74E-06	0,0014427	0,000344451	0,045464285	DOWN	Ctla4	1:60887000-60915832	protein_coding	+
ENSMUSG00000041268.16	159,3018795	-4,67E-06	0,001442695	0,00035427	0,046303905	DOWN	Dmxl2	9:54365158-54501760	protein_coding	-
ENSMUSG00000023903.7	90,37320192	-1,799974356	0,728369175	0,000357495	0,046303905	DOWN	Mmp25	17:23629458-23645269	protein_coding	-
ENSMUSG00000046805.9	571,8105854	-1,717072919	0,703905424	0,000371387	0,047657892	DOWN	Mpeg1	19:12460779-12465284	protein_coding	+
