# Supplementary material for: EOMES and IL-10 regulate antitumor activity of T regulatory type 1 CD4+ T cells in chronic lymphocytic leukemia
Source: Leukemia. 2021 Feb 1;35(8):2311–24. doi: 10.1038/s41375-021-01136-1 (PMC8324479; doi:10.1038/s41375-021-01136-1)
Supplement: Supplementary file 11 — Suppl. Table 6_sheet 3_intersection [file 41375_2021_1136_MOESM11_ESM.pdf]

Intersection comparison 1 and 2
external_gene_name
6430548M08Rik
Abca13
Adamts14
Aim1l
Aldh2
Alox5
Amica1
Arnt2
B430306N03Rik
C3
Camp
Cass4
Cd177
Cd300lf
Cebpe
Ckap4
Clgn
Cpm
Dmxl2
F5
F630028O10Rik
Fam101b
Fcnb
Foxp3
Gas7
Gm29112
Gpr55
Hp
Igsf6
Ikzf2
Il10
Ildr1
Itgax
Itgb1
Itgb2l
Lrg1
Ltf
Map3k9
Mpeg1
Ms4a3
Nedd4
Olfml2b
Pdcd1
Pde2a

Prg2
Prom1
Rab44
Raver2
S1pr5
Slfn4
Socs3
Sort1
Spi1
Spock2
Syk
Tbc1d8
Tigit
Tox
Trav7-5
Wdfy4
Zfp683
