# Supplementary material for: EOMES and IL-10 regulate antitumor activity of T regulatory type 1 CD4+ T cells in chronic lymphocytic leukemia
Source: Leukemia. 2021 Feb 1;35(8):2311–24. doi: 10.1038/s41375-021-01136-1 (PMC8324479; doi:10.1038/s41375-021-01136-1)
Supplement: Supplementary file 12 — Suppl. Table 7 [file 41375_2021_1136_MOESM12_ESM.pdf]

¬© 2000-2020 QIAGEN. All rights reserved.

| Upstream Regulator | Molecule Type | Predicted Activation State | Activation z-score | p-value of overlap | Target Molecules in Dataset |
|---|---|---|---|---|---|
| STAT3 | transcription regulator | | -1,335 | 1,41E-10 | BCL2,CD86,CTLA4,FOXP3,GBP3,HP,IFI16,IKZF2,IL10,ITGB1,LTF,NOTCH3,PDCD1,PROM1,SLFN12L,SOCS3,SPI1 |
| IL10RA | transmembrane receptor | | 0,632 | 1,73E-10 | ALDH2,ALOX5,BCL2,C3,CD300LF,CTLA4,FOXP3,IFI16,LRG1,MMP25,PDCD1,PDE2A,SOCS3 |
| IL2 | cytokine | | -1,341 | 2,09E-10 | ARNT2,BCL2,C3,CD86,CHN2,CTLA4,CX3CR1,FOXP3,IKZF2,IL10,IL12RB2,ITGB1,JAML,MMP25,PDCD1,SOCS3,TIGIT |
| tretinoin | chemical - endogenous mammalian | | 0,515 | 7,41E-10 | ALOX5,BCL2,C3,CD86,CEBPE,CHN2,CTLA4,CX3CR1,FOXP3,GAS7,GJB4,IFI16,IL10,IL12RB2,ITGAX,ITGB1,JAML,KIAA0513,LTF,MS4A3,PRG2,PROM1,RFLNB,SPI1,ZEB2 |
| IL4 | cytokine | | -1,143 | 7,53E-10 | ALDH2,ALOX5,BCL2,C3,CD300LF,CD86,CKAP4,FOSB,FOXP3,GBP3,IFI16,IL10,IL12RB2,ITGAX,ITGB1,MPEG1,Nedd4,PDCD1,SOCS3,SYK |
| STAT1 | transcription regulator | | -0,743 | 1,25E-09 | C3,CD86,CX3CR1,FOXP3,GBP3,IFI16,IL10,IL12RB2,ITGAX,MS4A3,SLFN12L,SOCS3,SORT1 |
| CSF3 | cytokine | Inhibited | -2,601 | 1,30E-09 | BCL2,CD177,CD300LF,CEBPE,CTLA4,CX3CR1,IL10,LTF,SOCS3,SPI1 |
| lipopolysaccharide | chemical drug | | -1,511 | 2,11E-09 | ALDH2,ALOX5,Art2a,BCL2,C3,CAMP,CD300LF,CD86,CKAP4,CX3CR1,FOSB,GBP3,H2-T10,HP,IFI16,IGSF6,IL10,IL12RB2,ITGAX,ITGB1,LTF,NOTCH3,OLFML2B,PDCD1,SLFN12L,SOCS3,SPI1 |
| IFNG | cytokine | | -0,424 | 2,70E-09 | ARNT2,Art2a,BCL2,C3,CD86,CX3CR1,FCN1,FOSB,FOXP3,GBP3,IFI16,IL10,IL12RB2,ITGAX,ITGB1,NOTCH3,PDCD1,PROM1,SLFN12L,SOCS3,SORT1,SPI1,ZEB2 |
| SRF | transcription regulator | Activated | 2,176 | 7,74E-09 | BCL2,CAMP,CD177,CEBPE,CPM,FCN1,FOSB,ITGB1,LTF,MS4A3,PDE2A,PRG2 |
| TGFB1 | growth factor | | -0,99 | 1,19E-08 | ALDH2,ALOX5,ARNT2,BCL2,C3,CAMP,CD86,CTLA4,CX3CR1,F5,FOSB,FOXP3,GAS7,IFI16,IL10,ITGA7,ITGAX,ITGB1,NOTCH3,PDCD1,PROM1,RFLNB,SOCS3,SPI1,ZEB2 |
| cholecalciferol | chemical - endogenous mammalian | | -1,065 | 1,60E-08 | ALOX5,BCL2,CAMP,CD86,FOXP3,GAS7,IL10,ITGAX |
| poly rI:rC-RNA | biologic drug | Inhibited | -2,064 | 2,01E-08 | BCL2,C3,CAMP,CD86,FCN1,IFI16,IL10,ITGAX,LTF,MS4A3,NOTCH3,SLFN12L,SOCS3,SYK,WDFY4 |
| tetrachlorodibenzodioxin | chemical toxicant | | -1,117 | 2,17E-08 | ALOX5,ARNT2,BCL2,CD86,CTLA4,DMXL2,H2-T10,IL10,ITGA7,ITGAX,Nedd4,SPOCK2 |
| FOXP3 | transcription regulator | | 1,987 | 3,05E-08 | BCL2,CTLA4,FOXP3,IKZF2,IL10,IL12RB2,PDCD1,TIGIT,ZEB2 |
| Immunoglobulin | complex | | 0,732 | 3,79E-08 | BCL2,CD86,CHN2,CRISPLD2,FOSB,FOXP3,GAS7,IKZF2,IL10,ITGAX,PDCD1,SOCS3,SPI1,TIGIT,ZEB2 |
| IL6 | cytokine | Inhibited | -2,735 | 3,81E-08 | BCL2,C3,CD86,CX3CR1,FOXP3,GBP3,HP,IFI16,IL10,ITGB1,LRG1,LTF,PDCD1,PROM1,SOCS3,SPI1 |
| AGT | growth factor | | -1,962 | 4,31E-08 | BCL2,C3,CD86,CLGN,HP,IFI16,IL10,ITGA7,ITGB1,NOTCH3,SOCS3,SRGAP3,ZEB2 |
| GATA2 | transcription regulator | | 0 | 5,39E-08 | ABCA13,ALOX5,ARNT2,CAMP,CD177,CEBPE,FCN1,LTF,MS4A3,PRG2,RAB44,SPI1 |
| TNF | cytokine | Inhibited | -2,67 | 7,27E-08 | ALDH2,ALOX5,BCL2,C3,CAMP,CD86,CRISPLD2,CTLA4,CX3CR1,FOSB,FOXP3,GBP3,HP,IFI16,IL10,ITGAX,ITGB1,LRG1,OLFML2B,PDCD1,PDE2A,SOCS3,TBC1D8,TOX |
| NFATC2 | transcription regulator | | -0,16 | 9,62E-08 | CD86,CTLA4,CX3CR1,FOXP3,IL10,IL12RB2,PDCD1,SLFN12L,SOCS3 |
| dexamethasone | chemical drug | | -1,578 | 9,89E-08 | ALDH2,ALOX5,BCL2,C3,CAMP,CD177,CD86,CRISPLD2,CX3CR1,F5,FOSB,FOXP3,GBP3,HP,IL10,ITGB1,MPEG1,MS4A3,NOTCH3,OLFML2B,PDCD1,SOCS3,SORT1,SPI1,SPOCK2,SYK |
| Tgf beta | group | | 1,011 | 1,70E-07 | BCL2,CTLA4,CX3CR1,FOXP3,IL10,ITGB1,PDCD1,PROM1,SLFN12L,SOCS3 |
| SP600125 | chemical - kinase inhibitor | | 0,601 | 2,05E-07 | ALOX5,BCL2,CD86,CKAP4,CPM,CTLA4,FOSB,FOXP3,IL10,SOCS3 |
| CTLA4 | transmembrane receptor | | 1,195 | 2,47E-07 | CD86,FOXP3,IL10,IL12RB2,ITGAX,PDCD1 |
| MRTFB | transcription regulator | Activated | 2,646 | 2,60E-07 | CAMP,CD177,CEBPE,FCN1,ITGB1,LTF,MS4A3,PRG2 |
| IL21 | cytokine | | -1,666 | 3,71E-07 | BCL2,CD86,FOXP3,IFI16,IL10,IL12RB2,PDCD1,SOCS3 |
| minocycline | chemical drug | | 0 | 4,41E-07 | ALOX5,BCL2,CD86,IL10,ITGAX,ITGB1 |
| tacrolimus | chemical drug | | 0,557 | 4,56E-07 | BCL2,CHN2,F5,FOSB,FOXP3,IKZF2,IL10,PROM1,TIGIT |
| MRTFA | transcription regulator | Activated | 2,813 | 4,58E-07 | CAMP,CD177,CEBPE,FCN1,ITGB1,LTF,MS4A3,PRG2 |
| E. coli B5 lipopolysaccharide | chemical - endogenous non-mammalian | | -0,619 | 6,54E-07 | ALDH2,CD86,CTLA4,CX3CR1,FOXP3,IL10,IL12RB2,PRDM16,SOCS3 |
| IL12A | cytokine | | -0,179 | 8,46E-07 | BCL2,CD86,IL10,IL12RB2,SOCS3 |
| JAK3 | kinase | | -0,395 | 1,05E-06 | BCL2,FOXP3,IL10,PDCD1,SOCS3 |
| CEBPA | transcription regulator | Inhibited | -2,216 | 1,11E-06 | BCL2,C3,CAMP,CEBPE,CKAP4,HP,IL10,ITGAX,LTF,SOCS3,SPI1 |
| IL10 | cytokine | | 0,54 | 1,21E-06 | ARNT2,BCL2,CAMP,CD86,CTLA4,CX3CR1,FOXP3,IL10,IL12RB2,PDCD1,SOCS3 |
| E. coli B4 lipopolysaccharide | chemical toxicant | | -1,187 | 1,36E-06 | BCL2,CD86,F5,FOSB,IFI16,IL10,Klrb1f,PDCD1,SOCS3 |
| SOCS3 | phosphatase | | -1,143 | 1,46E-06 | BCL2,CD86,CTLA4,FOXP3,IL10,SOCS3 |
| CEBPE | transcription regulator | | -1,317 | 1,91E-06 | BCL2,CAMP,IL10,LTF,PRG2 |
| AKT1 | kinase | | -1,701 | 1,96E-06 | BCL2,CD86,FOSB,FOXP3,IL10,ITGB1,PDCD1,PROM1,SORT1 |
| MOG | other | | -1,109 | 2,09E-06 | CD86,CTLA4,FOXP3,IL10,PDCD1 |
| VEGFA | growth factor | | -0,835 | 2,25E-06 | ALDH2,BCL2,CD86,CTLA4,FOSB,IL10,ITGB1,PDCD1,TBC1D8 |
| STAT4 | transcription regulator | | -1,231 | 2,26E-06 | BCL2,C3,IL10,IL12RB2,ITGA7,PDCD1,PRDM16,SOCS3 |
| TNFRSF4 | transmembrane receptor | | -0,339 | 2,30E-06 | BCL2,CTLA4,FOXP3,IL10 |
| TGM2 | enzyme | | 0,164 | 2,58E-06 | C3,CD86,CX3CR1,ITGAX,JAML,KIAA0513,MS4A3,RFLNB |
| EZH2 | transcription regulator | | -0,2 | 2,67E-06 | BCL2,C3,CRISPLD2,FOXP3,IL10,IL12RB2,PRG2,PROM1,SOCS3,SPOCK2 |
| immethridine | chemical reagent | Activated | 2 | 2,71E-06 | C3,HP,IFI16,SOCS3 |
| CpG oligonucleotide | chemical drug | | -0,795 | 2,79E-06 | BCL2,CD86,GBP3,IL10,ITGAX,PDCD1,SYK |
| VCAN | other | | -0,114 | 2,91E-06 | C3,CRISPLD2,IL10,ITGB1,NOTCH3,OLFML2B,SORT1 |
| prostaglandin E2 | chemical - endogenous mammalian | | -0,207 | 3,09E-06 | BCL2,CAMP,CD86,CTLA4,FOSB,FOXP3,IL10,ITGB1,SOCS3 |
| genistein | chemical drug | | -1,51 | 3,11E-06 | ALOX5,BCL2,C3,CD86,CX3CR1,FOXP3,IL10,LTF,SORT1,ZEB2 |
| SATB1 | transcription regulator | | -0,055 | 3,41E-06 | BCL2,CD86,F5,FOSB,ITGB1,PDCD1,SPI1 |
| VIP | other | | -0,744 | 3,50E-06 | CD86,CTLA4,FOXP3,IL10,PDCD1,SORT1 |
| fluticasone propionate | chemical drug | | -1,147 | 4,31E-06 | ALOX5,C3,CAMP,CD86,FCN1,IL10,OLFML2B |
| GATA3 | transcription regulator | | -0,636 | 4,37E-06 | BCL2,CTLA4,FOXP3,IL10,IL12RB2,PDCD1,SPI1,ZEB2 |
| DUSP1 | phosphatase | | 1,709 | 5,30E-06 | BCL2,CAMP,CD86,IL10,IL12RB2,LTF |
| CSF1 | cytokine | | -0,168 | 5,70E-06 | BCL2,CPM,GAS7,IL10,ITGAX,ITGB1,SLFN12L,SPI1 |
| FTH1 | enzyme | | | 6,21E-06 | CD86,IL10,PDCD1 |
| lipoteichoic acid | chemical - endogenous non-mammalian | | -1,96 | 7,46E-06 | BCL2,CAMP,CD86,IL10,PDCD1 |
| cyclosporin A | biologic drug | | 1,609 | 7,87E-06 | BCL2,CAMP,CD86,CTLA4,CX3CR1,F5,FOSB,FOXP3,IL10,IL12RB2 |
| ZFPM1 | transcription regulator | | | 8,00E-06 | ALOX5,IKZF2,IL12RB2,PRG2,SPI1 |
| Interferon alpha | group | | -1,271 | 8,83E-06 | BCL2,C3,CD86,GBP3,IFI16,IL10,IL12RB2,ITGB1,SLFN12L,SOCS3 |
| E. coli lipopolysaccharide | chemical - endogenous non-mammalian | | -0,93 | 9,15E-06 | BCL2,CD86,FOXP3,IL10,SOCS3 |
| SMAD3 | transcription regulator | | 0,304 | 9,63E-06 | BCL2,C3,CRISPLD2,FOXP3,HP,ITGB1,PDCD1,ZEB2 |
| PD98059 | chemical - kinase inhibitor | | 0,769 | 1,02E-05 | BCL2,C3,CAMP,CD86,FOSB,FOXP3,GAS7,IL10,ITGAX,ITGB1,SOCS3 |

| | | | | | |
|---|---|---|---|---|---|
| AHR | ligand-dependent nuclear receptor | | 0,152 | 1,04E-05 | ALDH2,ALOX5,DMXL2,FOXP3,HP,IL10,ITGA7,NOTCH3,SOCS3 |
| ID3 | transcription regulator | | | 1,08E-05 | BCL2,FOXP3,IFI16,IKZF2,IL10,PDCD1,SOCS3 |
| SB203580 | chemical - kinase inhibitor | | 1,925 | 1,11E-05 | BCL2,C3,CAMP,CD86,CX3CR1,FOSB,IL10,S1PR5,SOCS3,TOX |
| IL12 (family) | group | | -1,413 | 1,11E-05 | BCL2,CD86,FOXP3,IL10,IL12RB2,PDCD1 |
| Sb202190 | chemical - kinase inhibitor | | 0,114 | 1,11E-05 | ALOX5,BCL2,CAMP,CD86,IL10,SPI1 |
| BCR (complex) | complex | | -0,786 | 1,27E-05 | BCL2,CD86,FOSB,IL10,ITGB1,PDCD1 |
| resiquimod | chemical drug | | | 1,35E-05 | BCL2,C3,CD86,IFI16,IL10,ITGAX,MMP25,SPI1 |
| IL22 | cytokine | Inhibited | -2,4 | 1,38E-05 | BCL2,C3,CAMP,HP,IL10,SOCS3 |
| TET2 | enzyme | | 0,447 | 1,50E-05 | FCN1,FOXP3,IFI16,ITGAX,JAML,RAB44 |
| IFNB1 | cytokine | | -1,104 | 1,60E-05 | Art2a,BCL2,CD86,GBP3,IFI16,IL10,ITGAX,PDCD1 |
| ID2 | transcription regulator | | | 1,73E-05 | BCL2,IFI16,IKZF2,IL10,PDCD1,PROM1,SOCS3 |
| ITK | kinase | | -0,714 | 1,80E-05 | CX3CR1,FOXP3,IL10,Klrb1f,SRGAP3 |
| DICER1 | enzyme | | 0 | 1,81E-05 | CD86,FOXP3,GBP3,IL10,ITGAX,ITGB1,SOCS3,SPI1 |
| IL27 | cytokine | | 0,264 | 1,98E-05 | CD86,CTLA4,FOXP3,IL10,IL12RB2,SOCS3 |
| MAPK3 | kinase | | 1,387 | 2,02E-05 | CD86,FOSB,FOXP3,IL10,ITGB1 |
| Tcf7 | transcription regulator | | 0,378 | 2,12E-05 | BCL2,CHDH,MMP25,PDCD1,SOCS3,TIGIT,TOX |
| CHRNA1 | transmembrane receptor | | | 2,32E-05 | CD86,CTLA4,IL10 |
| SIRT1 | transcription regulator | | 0,186 | 2,44E-05 | ARNT2,BCL2,CRYBG2,FOXP3,GBP3,IL10,PRDM16,SOCS3,TBC1D8 |
| 6-hydroxydopamine | chemical toxicant | | -0,902 | 2,51E-05 | BCL2,C3,CEBPE,FOSB,IL10 |
| lipid A | chemical toxicant | | -1,967 | 2,55E-05 | C3,CD86,IL10,SYK |
| alitretinoin | chemical drug | | -0,297 | 2,98E-05 | BCL2,CD86,CEBPE,CPM,FOXP3,LTF |
| CSF2 | cytokine | Inhibited | -2,091 | 3,16E-05 | ALOX5,BCL2,C3,CD86,CTLA4,FOXP3,IL10,ITGAX,SOCS3,SPI1 |
| STAT6 | transcription regulator | | 0,77 | 3,43E-05 | ALDH2,BCL2,CKAP4,FOXP3,GBP3,IFI16,IL10,MPEG1,SYK |
| Cyp2c23 | enzyme | | | 3,45E-05 | CD86,CX3CR1,F5 |
| LAT | other | | | 3,45E-05 | CTLA4,FOXP3,IL10 |
| C5 | other | | | 3,45E-05 | IL10,ITGB1,SOCS3 |
| eicosapentenoic acid | chemical drug | | -0,739 | 3,59E-05 | CD86,IL10,ITGAX,PROM1,SPI1 |
| Ige | complex | | -1,551 | 3,81E-05 | ALOX5,BCL2,C3,CD86,IL10,ITGAX,SOCS3,SRGAP3 |
| CD274 | enzyme | | 0,323 | 3,89E-05 | CD86,FOXP3,IL10,PDCD1 |
| IFNA2 | cytokine | | -1,593 | 3,89E-05 | ALOX5,BCL2,CD86,IFI16,IL10,IL12RB2,SOCS3 |
| CD46 | transmembrane receptor | | | 4,13E-05 | C3,FOXP3,IL10 |
| ITGAL | transmembrane receptor | | | 4,13E-05 | CD86,IL10,IL12RB2 |
| CAMP | other | | -0,303 | 4,15E-05 | BCL2,CAMP,CD86,IL10,ITGAX |
| RPTOR | other | | -1,643 | 4,15E-05 | CD86,CTLA4,IL10,ITGAX,SPI1 |
| TSC22D3 | transcription regulator | | -0,373 | 4,21E-05 | BCL2,CD86,IL10,PDCD1 |
| TWIST1 | transcription regulator | | -0,798 | 4,21E-05 | BCL2,C3,LRG1,PROM1,SPOCK2,ZEB2 |
| galactosylceramide-alpha | chemical reagent | | -0,428 | 4,55E-05 | FOXP3,IL10,IL12RB2,PDCD1 |
| NFKBIA | transcription regulator | | -0,744 | 4,56E-05 | BCL2,C3,CAMP,CD86,GBP3,IFI16,IL10,ITGB1,SOCS3 |
| filgrastim | biologic drug | | -1,628 | 4,56E-05 | ALOX5,CD177,F5,GBP3,HP,IL10,MPEG1,MS4A3,SORT1 |
| Nfat (family) | group | | -1,432 | 4,56E-05 | BCL2,CTLA4,FOXP3,IL10,PDCD1 |
| IL15 | cytokine | | -1,213 | 4,64E-05 | BCL2,CD86,CX3CR1,FOXP3,IL10,IL12RB2,ITGAX,ITGB1,PDCD1 |
| IL1A | cytokine | | -0,692 | 4,66E-05 | BCL2,C3,CAMP,CD86,FOSB,IL10,ITGB1 |
| IL3 | cytokine | | -1,692 | 4,70E-05 | ALDH2,BCL2,CD86,CTLA4,ITGAX,PRG2,SOCS3,SPI1 |
| mir-130 | microRNA | | | 4,89E-05 | CAMP,CEBPE,LTF |
| TREM2 | transmembrane receptor | | | 4,89E-05 | CD86,CX3CR1,IL10 |
| docosahexaenoic acid | chemical drug | | -0,795 | 5,30E-05 | BCL2,CD86,CTLA4,FOXP3,IL10,SOCS3 |
| OSM | cytokine | | -1,493 | 5,42E-05 | BCL2,CAMP,CPM,FOSB,GAS7,HP,IL10,LRG1,NOTCH3,SOCS3 |
| ICAM1 | transmembrane receptor | | 0 | 5,68E-05 | CD86,IL12RB2,PROM1,ZEB2 |
| PIM1 | kinase | | 0,689 | 5,68E-05 | BCL2,CTLA4,IL10,IL12RB2 |
| BACH2 | transcription regulator | | 0,247 | 5,68E-05 | BCL2,FOXP3,IL10,IL12RB2 |
| PRDM1 | transcription regulator | Inhibited | -2,381 | 5,69E-05 | CD86,CRISPLD2,F5,IL10,MMP25,PDCD1,TIGIT |
| ssRNA40 | chemical reagent | | | 5,74E-05 | CD86,IL10,SOCS3 |
| EBI3 | cytokine | | -0,751 | 6,10E-05 | CD86,IL10,IL12RB2,PDCD1 |
| calcitriol | chemical drug | | -0,491 | 6,18E-05 | ALOX5,BCL2,C3,CAMP,CTLA4,FOXP3,IL10,ITGAX,LTF,SOCS3 |
| CD28 | transmembrane receptor | | -0,281 | 6,23E-05 | BCL2,CD86,CTLA4,FOXP3,IL10,IL12RB2,PDCD1,SOCS3 |
| TLR3 | transmembrane receptor | Inhibited | -2,019 | 6,41E-05 | C3,CD86,CPM,GBP3,IFI16,IL10,SOCS3 |
| STING1 | other | | -1,941 | 6,54E-05 | CD86,GAS7,IFI16,IL10 |
| S100A8 | other | | -0,625 | 6,60E-05 | BCL2,CHDH,CPM,CTLA4,IL10,SOCS3 |
| IDO1 | enzyme | | | 6,67E-05 | CD86,IL10,ITGAX |
| KLF2 | transcription regulator | | 1,109 | 6,80E-05 | CD86,CX3CR1,FOXP3,ITGB1,NOTCH3,S1PR5 |
| IL18 | cytokine | | -0,791 | 6,80E-05 | BCL2,CAMP,CD86,IL10,IL12RB2,PDCD1 |
| NOTCH1 | transcription regulator | | -1,348 | 7,05E-05 | BCL2,FOSB,IFI16,IL10,ITGB1,NOTCH3,SPI1 |
| hyaluronic acid | chemical - endogenous mammalian | | -0,524 | 7,41E-05 | BCL2,CD86,FOXP3,IL10,SOCS3 |
| IL15RA | transmembrane receptor | | | 7,71E-05 | BCL2,IL10,ITGAX |
| IRF4 | transcription regulator | Inhibited | -2,173 | 7,90E-05 | CTLA4,CX3CR1,FOXP3,IKZF2,IL10,ITGB1 |
| ionomycin | chemical reagent | | -1,021 | 7,90E-05 | BCL2,CTLA4,FOSB,FOXP3,IL10,PDCD1 |
| adenosine | chemical - endogenous mammalian | | | 8,00E-05 | CD86,CX3CR1,IL10,SOCS3 |
| STAT2 | transcription regulator | | | 8,00E-05 | BCL2,CD86,IL10,SOCS3 |
| NOTCH3 | transcription regulator | | | 8,00E-05 | BCL2,FOXP3,IL10,PROM1 |
| C1QTNF6 | other | | | 8,50E-05 | IL10,ITGAX |
| abrocitinib | chemical drug | | | 8,50E-05 | BCL2,SOCS3 |
| phenanthridine | chemical toxicant | | | 8,50E-05 | CD86,IL10 |
| NOS2 | enzyme | | -1,485 | 8,62E-05 | BCL2,CD86,FOXP3,H2-T10,IL10,SLFN12L |
| TCR | complex | | -0,422 | 8,79E-05 | BCL2,CTLA4,F5,FOXP3,IL10,IL12RB2,PDCD1,SOCS3 |
| MBP | other | | | 8,84E-05 | FOXP3,IL10,IL12RB2 |
| TYROBP | transmembrane receptor | | 0,849 | 9,10E-05 | CD86,IL10,ITGAX,ITGB1 |
| OSMR | transmembrane receptor | | | 9,69E-05 | BCL2,ITGAX,LRG1,SOCS3 |
| BCL3 | transcription regulator | | 1,062 | 9,69E-05 | BCL2,CAMP,CD86,IL10 |
| salmonella minnesota R595 lipopolysaccharides | chemical - endogenous non-mammalian | | -1,446 | 9,83E-05 | CD86,FOSB,IL10,SLFN12L,SOCS3 |
| PDCD1 | phosphatase | | -0,686 | 1,10E-04 | BCL2,CD86,IL10,PDCD1 |
| TRA | transmembrane receptor | | | 1,14E-04 | BCL2,IL10,SOCS3 |
| NODAL | growth factor | | | 1,14E-04 | CD86,IL10,ITGAX |
| ibrutinib | chemical drug | | | 1,14E-04 | BCL2,CD86,ITGB1 |
| hemocyanin | biologic drug | | | 1,14E-04 | CD86,IL10,ITGAX |
| CRH | cytokine | | 0,228 | 1,16E-04 | BCL2,CAMP,IL10,SOCS3 |
| NFKB1 | transcription regulator | Inhibited | -2,187 | 1,23E-04 | BCL2,CD86,FOSB,FOXP3,IFI16,IL10,SPI1 |
| ATP-gamma-S | chemical reagent | | | 1,23E-04 | C3,CD86,IL10,MPEG1 |

| | | | | | |
|---|---|---|---|---|---|
| zymosan | chemical - endogenous non-mammalian | | -1,929 | 1,31E-04 | ARNT2,CD86,IL10,SOCS3 |
| TBX21 | transcription regulator | | 1,95 | 1,38E-04 | CX3CR1,IL12RB2,PDCD1,ZEB2 |
| carvacrol | chemical - endogenous non-mammalian | | | 1,41E-04 | BCL2,IL10 |
| TLN1 | other | | | 1,41E-04 | BCL2,ITGB1 |
| ACAN | other | | | 1,41E-04 | FOXP3,IL10 |
| scoparone | chemical - endogenous non-mammalian | | | 1,41E-04 | BCL2,SOCS3 |
| 3-nitrotyrosine | chemical reagent | | | 1,41E-04 | ITGA7,ITGB1 |
| IL12 (complex) | complex | | -0,602 | 1,42E-04 | CD86,FOXP3,IL10,IL12RB2,PDCD1,SOCS3 |
| FLT3LG | cytokine | | -0,747 | 1,46E-04 | CD86,FOXP3,IL10,SPI1 |
| BATF | transcription regulator | | | 1,61E-04 | CTLA4,IKZF2,IL10 |
| MALP-2s | chemical reagent | | | 1,61E-04 | CAMP,CD86,IL10 |
| LGALS1 | other | | -1,914 | 1,63E-04 | CTLA4,IL10,PDCD1,TOX |
| ERK1/2 | group | | -1,501 | 1,68E-04 | ALOX5,BCL2,C3,CD86,FOSB,IL10,SOCS3 |
| IgG | complex | | -0,446 | 1,97E-04 | C3,CD86,FOSB,FOXP3,IL10,IL12RB2 |
| FCGR2B | transmembrane receptor | | | 1,99E-04 | CD86,IL10,SOCS3 |
| U0126 | chemical - kinase inhibitor | | 1,181 | 1,99E-04 | Art2a,BCL2,C3,CAMP,FOSB,IL10,ITGB1,SOCS3,SYK |
| CD40 | transmembrane receptor | | -1,198 | 2,02E-04 | BCL2,CD86,CTLA4,IL10,ITGB1,PDCD1 |
| EGF | growth factor | | -0,91 | 2,08E-04 | BCL2,CEBPE,FOSB,IL10,ITGB1,LTF,PROM1,SOCS3,SPI1 |
| obatoclax | chemical drug | | | 2,11E-04 | CTLA4,FOXP3 |
| W-54011 | chemical reagent | | | 2,11E-04 | CD86,IL10 |
| Trbv13-2 | other | | | 2,11E-04 | FOXP3,IL10 |
| fenebrutinib | chemical drug | | | 2,11E-04 | BCL2,CD86 |
| [Ac-His1,D-Phe2,Lys15,Arg16,Leu27]VIP-(3-7)-GRF-(8-27) | chemical reagent | | | 2,11E-04 | CD86,IL10 |
| APEX1 | enzyme | | | 2,19E-04 | ALOX5,FOSB,IL10 |
| BCL6 | transcription regulator | | 0,391 | 2,22E-04 | BCL2,CTLA4,FOXP3,IL10,SOCS3,SYK |
| delta-9-tetrahydrocannabinol | chemical drug | | 0,068 | 2,34E-04 | FOSB,IL12RB2,LTF,SOCS3 |
| Fc gamma receptor | group | | | 2,42E-04 | CD86,IL10,SOCS3 |
| epigallocatechin-gallate | chemical drug | | -0,739 | 2,44E-04 | BCL2,FOSB,FOXP3,GBP3,IL10,PRG2 |
| IL7 | cytokine | | -0,625 | 2,44E-04 | BCL2,CD86,FOXP3,PDCD1,SOCS3 |
| Gsk3 | group | | | 2,45E-04 | BCL2,FOSB,IL10,PDCD1 |
| fingolimod | chemical drug | | 0 | 2,45E-04 | BCL2,CD86,FOSB,IL10 |
| IL13 | cytokine | | -1,009 | 2,51E-04 | C3,CD86,CHN2,DMXL2,GAS7,IL10,SOCS3,SORT1 |
| IFN alpha/beta | group | | -1,953 | 2,57E-04 | CD86,GAS7,IFI16,IL10 |
| JUND | transcription regulator | | | 2,57E-04 | C3,CTLA4,IKZF2,IL10 |
| butyric acid | chemical - endogenous mammalian | | -0,782 | 2,88E-04 | ALOX5,BCL2,CAMP,CD86,IFI16,IL10,SOCS3,SYK |
| OSCAR | other | | | 2,90E-04 | BCL2,CD86,CX3CR1 |
| JUNB | transcription regulator | | 0 | 2,94E-04 | C3,FOXP3,IFI16,IKZF2,IL10 |
| IL1B | cytokine | | -1,949 | 2,94E-04 | BCL2,C3,CD86,CX3CR1,FOSB,GBP3,HP,IFI16,IL10,ITGB1,PDCD1,SOCS3 |
| PIMREG | other | | | 2,95E-04 | IL10,SOCS3 |
| DDT | chemical toxicant | | | 2,95E-04 | IL10,LTF |
| Nr1h | group | | 0,447 | 3,03E-04 | ARNT2,BCL2,C3,IL10,SLFN12L |
| cytokine | group | | -1,185 | 3,03E-04 | ALOX5,BCL2,CD86,FOXP3,SOCS3 |
| PRKCQ | kinase | | | 3,16E-04 | FOXP3,IL10,ITGB1 |
| indole-3-carbinol | chemical drug | | | 3,16E-04 | BCL2,FOXP3,HP |
| SP1 | transcription regulator | | -1,154 | 3,18E-04 | ALOX5,BCL2,CAMP,FOXP3,IL10,IL12RB2,ITGAX,PROM1,ZEB2 |
| PTEN | phosphatase | | 0,546 | 3,29E-04 | BCL2,C3,CD86,CX3CR1,IL10,ITGB1,Nedd4,SOCS3,SYK,ZEB2 |
| IFNAR1 | transmembrane receptor | | -1,803 | 3,31E-04 | CD86,IFI16,IL10,SLFN12L,SOCS3 |
| SMAD4 | transcription regulator | | | 3,35E-04 | FOXP3,HP,IL10,ITGB1,NOTCH3,SPI1 |
| TLR9 | transmembrane receptor | Inhibited | -2,096 | 3,35E-04 | BCL2,CD86,CPM,IFI16,IL10,SOCS3 |
| PLP1 | other | | | 3,44E-04 | FOXP3,IL10,NOTCH3 |
| CBFB | transcription regulator | | -1 | 3,53E-04 | CTLA4,FOXP3,IKZF2,Nedd4 |
| TCL1A | transcription regulator | | | 3,69E-04 | CAMP,IGSF6,LTF,MPEG1 |
| niacinamide | chemical - endogenous mammalian | | 0 | 3,69E-04 | BCL2,CAMP,FOXP3,IL10 |
| 4-phenylbutyric acid | chemical - endogenous mammalian | | | 3,69E-04 | BCL2,CAMP,FOXP3,IL10 |
| CAMK4 | kinase | | | 3,73E-04 | BCL2,FOSB,FOXP3 |
| CD4 | transmembrane receptor | | | 3,73E-04 | BCL2,IL10,PDCD1 |
| IL27RA | transmembrane receptor | | | 3,73E-04 | CD86,IL10,SOCS3 |
| Proinsulin | group | | | 3,93E-04 | CTLA4,IL10 |
| CBX1 | transcription regulator | | | 3,93E-04 | FOXP3,ITGA7 |
| PIGR | transporter | | | 3,93E-04 | CD86,IL10 |
| benzoic acid | chemical - endogenous mammalian | | | 3,93E-04 | FOXP3,ITGAX |
| pranlukast | chemical drug | | | 3,93E-04 | BCL2,IL10 |
| lacto-N-fucopentaose III | chemical - endogenous mammalian | | | 3,93E-04 | CD86,IL10 |
| CYLD | transcription regulator | | | 4,04E-04 | CD86,CTLA4,IL10 |
| ADORA2B | G-protein coupled receptor | | | 4,04E-04 | BCL2,CD86,IL10 |
| ethylene glycol tetraacetic acid | chemical reagent | | | 4,04E-04 | BCL2,CD86,IL10 |
| bucladesine | chemical toxicant | Inhibited | -2,037 | 4,04E-04 | BCL2,CAMP,CD86,CTLA4,IL10,SOCS3,ZEB2 |
| MAPK9 | kinase | | 1,342 | 4,05E-04 | BCL2,FOXP3,IFI16,IL12RB2,SLFN12L |
| RELA | transcription regulator | | -0,816 | 4,08E-04 | BCL2,C3,CAMP,FOSB,FOXP3,IKZF2,IL10,SPI1 |
| beta-estradiol | chemical - endogenous mammalian | Inhibited | -2,737 | 4,19E-04 | ARNT2,BCL2,C3,CD86,CPM,CTLA4,FOSB,FOXP3,IGSF6,IL10,ITGB1,LTF,MPEG1,NOTCH3,PROM1,SOCS3,SPOCK2,SYK,ZEB2 |
| AG490 | chemical - kinase inhibitor | | 0,471 | 4,36E-04 | BCL2,FOXP3,PDCD1,SOCS3 |
| INPP5D | phosphatase | | | 4,37E-04 | CD86,FOXP3,IL10 |
| rosiglitazone | chemical drug | Inhibited | -2,393 | 4,38E-04 | ALOX5,BCL2,C3,CD86,HP,IL10,LRG1,SOCS3 |
| curcumin | chemical drug | | 0,782 | 4,47E-04 | ALOX5,BCL2,CAMP,CD86,FOSB,IL10,SOCS3 |
| RARA | ligand-dependent nuclear receptor | | 0,6 | 4,70E-04 | ALOX5,ARNT2,CEBPE,FOXP3,GJB4,IL12RB2,LTF |

| | | | | | |
|---|---|---|---|---|---|
| 4-methylnitrosoamino-1-(3-pyridinyl)-1-butanone | chemical toxicant | | | 4,71E-04 | ALOX5,BCL2,HP |
| Ifnar | group | | -1,944 | 4,73E-04 | C3,CD86,IFI16,IL10 |
| S100A9 | other | | -0,6 | 4,77E-04 | C3,CHDH,CPM,CTLA4,SOCS3 |
| IRF7 | transcription regulator | | 0,277 | 4,90E-04 | CTLA4,FOXP3,GBP3,IFI16,ITGAX |
| daidzein | chemical drug | | | 4,92E-04 | BCL2,CD86,FOSB,ZEB2 |
| 6-amino-4-(4-phenoxyphenylethylamino)quinazoline | chemical reagent | | | 5,04E-04 | C3,ITGB1 |
| cytidylyl-3'-5'-guanosine | chemical reagent | | | 5,04E-04 | BCL2,CD86 |
| CTSZ | peptidase | | | 5,04E-04 | CD86,IL10 |
| CDKN2B | transcription regulator | | | 5,04E-04 | BCL2,CD86 |
| HA900 | chemical reagent | | | 5,04E-04 | IL10,SOCS3 |
| ammonium trichloro(dioxoethylene-O,O'-)tellurate | chemical drug | | | 5,04E-04 | BCL2,IL10 |
| PGF | growth factor | | | 5,06E-04 | ALOX5,BCL2,FOSB |
| miR-146a-5p (and other miRNAs w/seed GAGAACU) | mature microRNA | | 1 | 5,32E-04 | CAMP,IL10,IL12RB2,LTF |
| LGALS3 | other | | 1,053 | 5,32E-04 | BCL2,IL10,ITGB1,SOCS3 |
| CD2 | transmembrane receptor | | | 5,44E-04 | BCL2,CD86,IL10 |
| ADIPOQ | other | | -0,241 | 5,44E-04 | BCL2,FOXP3,IL10,SOCS3,SPI1 |
| tetradecanoylphorbol acetate | chemical drug | | -0,76 | 5,53E-04 | BCL2,C3,CTLA4,FOSB,FOXP3,GPR55,IFI16,IL10,IL12RB2,ITGAX,ITGB1,PDCD1,SOCS3 |
| JUN | transcription regulator | | -1,936 | 5,67E-04 | C3,CAMP,IFI16,IL10,ITGB1,SOCS3,SYK,ZEB2 |
| P38 MAPK | group | | -1,173 | 5,69E-04 | BCL2,CD86,HP,IL10,Nedd4,NOTCH3,SOCS3 |
| FAS | transmembrane receptor | | | 5,69E-04 | BCL2,CD86,F5,FOSB,IL10,ITGA7,SYK |
| CEBPD | transcription regulator | | | 5,96E-04 | C3,HP,IL10,ITGAX |
| MYD88 | other | | -1,738 | 6,06E-04 | C3,CD86,HP,IL10,ITGAX,SOCS3 |
| IL10RB | transmembrane receptor | | | 6,28E-04 | IL10,SOCS3 |
| TNFRSF17 | transmembrane receptor | | | 6,28E-04 | BCL2,CD86 |
| KCNA3 | ion channel | | | 6,28E-04 | CTLA4,IL10 |
| progesterone | chemical - endogenous mammalian | | 0,729 | 6,52E-04 | BCL2,C3,CD86,FOXP3,IL10,ITGB1,LTF,NOTCH3,TBC1D8 |
| 4-hydroxytamoxifen | chemical drug | | -0,393 | 6,66E-04 | BCL2,C3,FOSB,IL10,NOTCH3 |
| FLT3 | kinase | | | 6,67E-04 | BCL2,CEBPE,IL10 |
| NR4A1 | ligand-dependent nuclear receptor | | 0,059 | 6,79E-04 | BCL2,FOXP3,IKZF2,IL10,ITGB1,PDCD1 |
| TLR4 | transmembrane receptor | Inhibited | -2,191 | 6,96E-04 | BCL2,C3,CD86,IFI16,IL10,SLFN12L,SOCS3 |
| LTA | cytokine | | | 7,11E-04 | FOXP3,IL10,ITGB1 |
| hexamethylene bisacetamide | chemical reagent | | | 7,11E-04 | BCL2,CEBPE,SPI1 |
| NFATC1 | transcription regulator | | | 7,14E-04 | BCL2,CX3CR1,FOXP3,PDCD1 |
| IRF1 | transcription regulator | | | 7,34E-04 | BCL2,FOXP3,IL10,IL12RB2,SPI1 |
| Hsp27 | group | | | 7,58E-04 | BCL2,CD86,IL10 |
| C3AR1 | G-protein coupled receptor | | | 7,58E-04 | C3,CD86,IL10 |
| quinolinic acid | chemical - endogenous mammalian | | | 7,58E-04 | BCL2,FOSB,FOXP3 |
| TNFSF18 | cytokine | | | 7,66E-04 | CTLA4,FOXP3 |
| acriflavine | chemical toxicant | | | 7,66E-04 | IL10,SOCS3 |
| kukoamine A | chemical - endogenous non-mammalian | | | 7,66E-04 | ALOX5,BCL2 |
| NUP98-DDX10 | fusion gene/product | | | 8,06E-04 | ALOX5,BCL2,MS4A3 |
| MAPK14 | kinase | | -1,274 | 8,08E-04 | BCL2,CD86,IL10,SLFN12L,SOCS3 |
| IKBKB | kinase | | -0,663 | 8,15E-04 | BCL2,C3,GBP3,IFI16,IL10,SOCS3 |
| TLR2 | transmembrane receptor | | -1,963 | 8,27E-04 | CD86,FOXP3,GBP3,IL10,SOCS3 |
| Akt | group | | -0,148 | 8,45E-04 | BCL2,FOXP3,IL10,Nedd4,PRDM16,SOCS3 |
| IL24 | cytokine | | | 8,56E-04 | BCL2,FOXP3,IL10 |
| CD86 | transmembrane receptor | | | 8,56E-04 | CD86,CTLA4,IL10 |
| HOXA3 | transcription regulator | | | 8,56E-04 | ALOX5,ITGB1,SPI1 |
| GATA1 | transcription regulator | | -1,117 | 8,91E-04 | ALOX5,BCL2,FOSB,ITGAX,PRG2,SPOCK2 |
| EPO | cytokine | | -0,125 | 9,07E-04 | ABCA13,BCL2,CLGN,FOSB,ITGB1,SOCS3 |
| KIT | transmembrane receptor | | | 9,08E-04 | BCL2,IL10,ITGAX,SOCS3 |
| highly active antiretroviral therapy | chemical drug | | | 9,16E-04 | BCL2,CTLA4 |
| Il8r | group | | | 9,16E-04 | BCL2,ITGAX |
| TCIRG1 | enzyme | | | 9,16E-04 | CD86,CTLA4 |
| CLEC1B | transmembrane receptor | | | 9,16E-04 | IL10,SYK |
| LECT2 | other | | | 9,16E-04 | C3,IL10 |
| LY-2510924 | biologic drug | | | 9,16E-04 | BCL2,MS4A3 |
| TNFAIP3 | enzyme | | | 9,63E-04 | BCL2,IL10,SOCS3 |
| PI3K (complex) | complex | | -1,377 | 9,72E-04 | BCL2,CD86,IL10,ITGB1,SOCS3,SPI1 |
| BTK | kinase | | -0,943 | 1,00E-03 | CD86,IL10,ITGAX,SPI1 |
| EGFR | kinase | | 0,64 | 1,04E-03 | BCL2,FOXP3,HP,IL10,PROM1,SOCS3,ZEB2 |
| phytohemagglutinin | chemical drug | | -1,953 | 1,06E-03 | ALDH2,CPM,CTLA4,GPR55,IL10,IL12RB2 |
| CD3 group | group | | | 1,08E-03 | FOXP3,IL10,PDCD1 |
| CD5 | transmembrane receptor | | | 1,08E-03 | BCL2,CD86,IL10 |
| WP1066 | chemical drug | | | 1,08E-03 | BCL2,CD86 |
| BPIFA1 | other | | | 1,08E-03 | CAMP,LTF |
| IgG2a | complex | | | 1,08E-03 | BCL2,IL10 |
| CD19 | transmembrane receptor | | | 1,08E-03 | IL10,PDCD1 |
| DOCK2 | other | | | 1,08E-03 | IL10,PRDM16 |
| CD180 | other | | | 1,08E-03 | CD86,IL10 |
| 1-chloro-2-(2,2,2-trichloro-1-(4-chlorophenyl)ethyl)benzene | chemical toxicant | | | 1,08E-03 | ARNT2,LTF |
| auranofin | chemical drug | | | 1,08E-03 | C3,HP |
| deferoxamine | chemical drug | | -0,277 | 1,08E-03 | BCL2,CAMP,GAS7,IFI16,PROM1 |
| etoposide | chemical drug | | 0,776 | 1,11E-03 | BCL2,CD86,FOXP3,IL10,NOTCH3 |
| mir-155 | microRNA | | 1,961 | 1,13E-03 | BCL2,IL10,SOCS3,SPI1 |

| | | | | |
|---|---|---|---|---|
| AKT2 | kinase | | 1,14E-03 | BCL2,FOXP3,ITGB1 |
| HDL | complex | | 1,20E-03 | CD86,CX3CR1,ITGB1 |
| CLEC11A | growth factor | | 1,20E-03 | ALOX5,BCL2,SPI1 |
| PARP1 | enzyme | 1,091 | 1,24E-03 | BCL2,CTLA4,FOXP3,IL10 |
| NFKBID | transcription regulator | | 1,26E-03 | CD86,IL10 |
| SCAVENGER receptor CLASS A | group | | 1,26E-03 | CD86,IL10 |
| MGAT3 | enzyme | | 1,26E-03 | NOTCH3,PROM1 |
| CSF3R | transmembrane receptor | | 1,26E-03 | CEBPE,LTF |
| salmeterol | chemical drug | | 1,26E-03 | IL10,SOCS3 |
| E64d | chemical - protease inhibitor | | 1,26E-03 | C3,ITGB1 |
| CALC | group | | 1,26E-03 | CD86,SORT1 |
| PTPN6 | phosphatase | | 1,26E-03 | BCL2,CD86,IL10 |
| CHUK | kinase | -1,133 | 1,31E-03 | C3,GBP3,IFI16,IL10,SOCS3 |
| NCOR2 | transcription regulator | | 1,33E-03 | BCL2,C3,CEBPE |
| ZEB2 | transcription regulator | | 1,33E-03 | CX3CR1,FCN1,MS4A3 |
| IL32 | cytokine | | 1,33E-03 | BCL2,IL10,SPI1 |
| FGF1 | growth factor | 0,762 | 1,36E-03 | IKZF2,ITGAX,NOTCH3,RFLNB |
| CD14 | transmembrane receptor | | 1,40E-03 | IL10,ITGB1,SOCS3 |
| CpG ODN 1668 | chemical reagent | | 1,40E-03 | CD86,IL10,SOCS3 |
| TLR7 | transmembrane receptor | -1,933 | 1,42E-03 | BCL2,CD86,DMXL2,IL10,ITGAX |
| monophosphoryl lipid A | chemical drug | | 1,45E-03 | CD86,IL10 |
| cyclic GMP | chemical - endogenous mammalian | | 1,45E-03 | BCL2,IL12RB2 |
| TRAF5 | transporter | | 1,45E-03 | CD86,IL10 |
| CD83 | transmembrane receptor | | 1,45E-03 | CD86,IL10 |
| CD209 | other | | 1,45E-03 | CD86,IL10 |
| ALK | kinase | | 1,45E-03 | FOXP3,IL10 |
| VEGFC | growth factor | | 1,45E-03 | BCL2,ITGB1 |
| NRP1 | transmembrane receptor | | 1,45E-03 | BCL2,CD86 |
| IL18R1 | transmembrane receptor | | 1,45E-03 | IL10,SOCS3 |
| PDE5A | enzyme | | 1,45E-03 | FOXP3,PDE2A |
| FITC | chemical reagent | | 1,45E-03 | CD86,IL10 |
| IL11 | cytokine | | 1,47E-03 | BCL2,IL10,SOCS3 |
| IL12B | cytokine | | 1,47E-03 | CD86,IL10,SOCS3 |
| dimethyl sulfoxide | chemical drug | | 1,52E-03 | ALOX5,CEBPE,IFI16,SPI1 |
| chloroquine | chemical drug | | 1,54E-03 | BCL2,CD86,SOCS3 |
| Creb | group | -1,546 | 1,56E-03 | ALOX5,BCL2,CAMP,FOSB,IFI16,IL10 |
| MET | kinase | 1,119 | 1,57E-03 | BCL2,ITGB1,SOCS3,ZEB2 |
| ETS1 | transcription regulator | 1,968 | 1,61E-03 | FOXP3,IKZF2,IL10,SPI1,ZEB2 |
| PDGF BB | complex | -0,851 | 1,61E-03 | C3,FOSB,ITGB1,SOCS3,SPI1,SYK |
| vitamin D | chemical drug | | 1,62E-03 | BCL2,CAMP,IL10 |
| TAL1 | transcription regulator | -1 | 1,64E-03 | BCL2,C3,SPI1,SYK,TOX |
| misoprostol | chemical drug | | 1,65E-03 | IL10,SOCS3 |
| GPNMB | enzyme | | 1,65E-03 | ITGB1,SPI1 |
| IGFBP1 | other | | 1,65E-03 | BCL2,ITGB1 |
| MAPKAP1 | other | | 1,65E-03 | FOXP3,IFI16 |
| CR2 | transmembrane receptor | | 1,65E-03 | BCL2,PDCD1 |
| VIPR1 | G-protein coupled receptor | | 1,65E-03 | CD86,IL10 |
| GFAP | other | | 1,65E-03 | ITGA7,ITGB1 |
| 2-acetylaminofluorene | chemical toxicant | | 1,65E-03 | BCL2,NOTCH3 |
| erythromycin | chemical drug | | 1,65E-03 | CD86,IL10 |
| myricetin | chemical - endogenous non-mammalian | | 1,65E-03 | BCL2,IL10 |
| C5AR1 | G-protein coupled receptor | | 1,70E-03 | C3,CD86,IL10 |
| carbon monoxide | chemical - endogenous mammalian | | 1,70E-03 | BCL2,IL10,ITGB1 |
| forskolin | chemical toxicant | -0,951 | 1,70E-03 | BCL2,CAMP,CD86,CEBPE,CRISPLD2,FOSB,HP,IL10,LTF |
| IL6ST | transmembrane receptor | | 1,78E-03 | HP,LTF,SOCS3 |
| BCL11B | transcription regulator | | 1,86E-03 | BCL2,FOXP3,IL10 |
| TNFRSF21 | transmembrane receptor | | 1,87E-03 | CTLA4,IL10 |
| GAL | other | | 1,87E-03 | BCL2,SORT1 |
| HHEX | transcription regulator | | 1,87E-03 | CTLA4,FOXP3 |
| TNK1 | kinase | | 1,87E-03 | IFI16,IL10 |
| PIM2 | kinase | | 1,87E-03 | FOXP3,IL12RB2 |
| FABP5 | transporter | | 1,87E-03 | FOXP3,SOCS3 |
| CCAT2 | other | | 1,87E-03 | BCL2,ZEB2 |
| ethyl pyruvate | chemical drug | | 1,87E-03 | BCL2,IL10 |
| clenbuterol | chemical drug | | 1,87E-03 | IL10,SOCS3 |
| palmitic acid | chemical - endogenous mammalian | 0,651 | 1,87E-03 | BCL2,CD86,IL10,SOCS3,SORT1 |
| IKBKG | kinase | -0,927 | 1,90E-03 | C3,GBP3,IFI16,SOCS3 |
| LEP | growth factor | -1,289 | 2,02E-03 | BCL2,FOXP3,IL10,ITGAX,PRDM16,SOCS3,SPI1 |
| SOCS1 | other | 1,091 | 2,05E-03 | BCL2,CD86,IFI16,SOCS3 |
| magnolol | chemical - endogenous non-mammalian | | 2,09E-03 | BCL2,IL10 |
| TGAL copolymer | biologic drug | | 2,09E-03 | FOXP3,IL10 |
| neuroprotectin D1 | chemical - endogenous mammalian | | 2,09E-03 | BCL2,IL10 |
| FST | other | | 2,12E-03 | HP,LTF,PRDM16 |
| RUNX1 | transcription regulator | -0,147 | 2,13E-03 | CTLA4,FOXP3,IL10,ITGB1,SPI1 |
| TNFSF13B | cytokine | | 2,21E-03 | BCL2,CD86,IL10 |
| TSC1 | other | | 2,21E-03 | BCL2,FOXP3,PDCD1 |
| TICAM1 | other | -1,989 | 2,22E-03 | CD86,IFI16,IL10,SOCS3 |
| estrogen receptor | group | -1,187 | 2,25E-03 | BCL2,C3,ITGB1,LTF,NOTCH3 |
| IFN Beta | group | -1,042 | 2,27E-03 | BCL2,CD86,IFI16,IL10 |
| prostaglandin E1 | chemical - endogenous mammalian | | 2,33E-03 | CD86,IL10 |
| Rp-cAMPS | chemical - kinase inhibitor | | 2,33E-03 | ALDH2,BCL2 |

| | | | | |
|---|---|---|---|---|
| rituximab | biologic drug | | 2,33E-03 | BCL2,IL10 |
| IL31 | other | | 2,33E-03 | CD86,SOCS3 |
| NUMB | other | | 2,33E-03 | IFI16,PROM1 |
| PTPN2 | phosphatase | | 2,33E-03 | IL10,PRDM16 |
| ITGA6 | transmembrane receptor | | 2,33E-03 | BCL2,ITGA7 |
| morphine | chemical drug | 0,728 | 2,39E-03 | BCL2,FOSB,IL10,Nedd4 |
| JAK1 | kinase | | 2,40E-03 | IL10,LRG1,SOCS3 |
| MTOR | kinase | -1,458 | 2,42E-03 | BCL2,CD86,FOXP3,IFI16,IL10,SOCS3 |
| NFATC3 | transcription regulator | | 2,50E-03 | BCL2,FOSB,IL10 |
| ERK | group | -1,053 | 2,51E-03 | BCL2,FOXP3,IL10,NOTCH3,SORT1 |
| Tnf (family) | group | -1 | 2,51E-03 | BCL2,CD86,IL12RB2,SOCS3 |
| dacinostat | chemical drug | | 2,59E-03 | BCL2,IL10 |
| KLF10 | transcription regulator | | 2,59E-03 | BCL2,FOXP3 |
| HULC | other | | 2,59E-03 | BCL2,ITGB1 |
| sulfasalazine | chemical drug | | 2,59E-03 | FOXP3,HP |
| zinc sulfate | chemical drug | | 2,59E-03 | C3,IL10 |
| zymosan A | chemical - endogenous non-mammalian | | 2,59E-03 | CD86,IL10 |
| S-(2,3-bispalmitoyloxypropyl)-cysteine-GDPKHPKSF | chemical reagent | | 2,59E-03 | CAMP,CD86 |
| naloxone | chemical drug | | 2,59E-03 | BCL2,IL10 |
| MEF2D | transcription regulator | | 2,60E-03 | FOSB,IL10,SYK |
| ADAM17 | peptidase | | 2,71E-03 | ITGB1,LRG1,SOCS3 |
| IKZF1 | transcription regulator | -0,596 | 2,73E-03 | FUT10,IFI16,IL10,NOTCH3,SPI1 |
| JAK2 | kinase | | 2,76E-03 | ALOX5,F5,PDE2A,SOCS3 |
| NOD2 | other | | 2,81E-03 | CAMP,IL10,SOCS3 |
| MAP3K8 | kinase | | 2,82E-03 | HP,IL10,SLFN12L,SOCS3 |
| Pkg | group | | 2,85E-03 | BCL2,FOSB |
| Hmgb1 | transcription regulator | | 2,85E-03 | BCL2,IL10 |
| FUT8 | enzyme | | 2,85E-03 | ALDH2,PROM1 |
| Iga | complex | | 2,85E-03 | CD86,IL10 |
| ACKR1 | G-protein coupled receptor | | 2,85E-03 | IFI16,TBC1D8 |
| MGAT5 | enzyme | | 2,85E-03 | ITGB1,PROM1 |
| TREX1 | enzyme | | 2,85E-03 | CD86,IFI16 |
| LIPG | enzyme | | 2,85E-03 | BCL2,IL10 |
| EPOR | transmembrane receptor | | 2,85E-03 | SOCS3,SPI1 |
| CBX3 | transcription regulator | | 2,85E-03 | FOXP3,ITGA7 |
| tranilast | chemical drug | | 2,85E-03 | CD86,IL10 |
| W7 | chemical reagent | | 2,85E-03 | CD86,IL10 |
| dichloroacetic acid | chemical drug | | 2,85E-03 | FOXP3,IL10 |
| butaprost | chemical drug | | 2,85E-03 | IL10,SOCS3 |
| KITLG | growth factor | -1,964 | 2,92E-03 | ALOX5,BCL2,IL10,LTF,SYK |
| BTNL2 | transmembrane receptor | | 2,92E-03 | FOXP3,GBP3,IKZF2 |
| cyclophosphamide | chemical drug | | 2,92E-03 | BCL2,FOXP3,IL10 |
| CD3 | complex | -0,458 | 2,93E-03 | BCL2,CD86,CTLA4,FOXP3,IL10,IL12RB2,PDCD1,SOCS3 |
| CTNNB1 | transcription regulator | -1,011 | 3,01E-03 | BCL2,IL10,ITGB1,MPEG1,NOTCH3,PDCD1,PROM1,SPI1,SYK,ZEB2 |
| NFkB (complex) | complex | -1,967 | 3,10E-03 | BCL2,C3,CAMP,CD86,FOXP3,IL10,ITGB1,SOCS3 |
| Tnf receptor | group | | 3,13E-03 | C3,IL10 |
| IgG1 | complex | | 3,13E-03 | BCL2,CD86 |
| CD200 | other | | 3,13E-03 | FOXP3,IL10 |
| IL2RA | transmembrane receptor | | 3,13E-03 | CD86,IL10 |
| SFTPD | other | | 3,13E-03 | CTLA4,IL10 |
| CXCR3 | G-protein coupled receptor | | 3,13E-03 | BCL2,IL10 |
| GADD45A | other | | 3,13E-03 | BCL2,FOSB |
| ADIPOR1 | transmembrane receptor | | 3,13E-03 | BCL2,IL10 |
| IL21R | transmembrane receptor | | 3,13E-03 | IL10,SOCS3 |
| GRN | growth factor | | 3,13E-03 | BCL2,IL10 |
| LILRB1 | transmembrane receptor | | 3,13E-03 | BCL2,ITGB1 |
| coenzyme Q10 | chemical drug | | 3,13E-03 | BCL2,IL10 |
| Notch | group | | 3,15E-03 | IL10,NOTCH3,ZEB2 |
| nitric oxide | chemical - endogenous mammalian | | 3,24E-03 | BCL2,FOXP3,IL10,IL12RB2 |
| IRAK4 | kinase | | 3,27E-03 | CD86,FOSB,IL10 |
| IL17A | cytokine | -0,756 | 3,28E-03 | BCL2,C3,CAMP,IL10,SOCS3 |
| EGR2 | transcription regulator | -1,99 | 3,39E-03 | BCL2,IL10,SOCS3,ZEB2 |
| MAP2K1/2 | group | | 3,39E-03 | C3,FOXP3,IL10 |
| SFTPA1 | transporter | | 3,39E-03 | FOSB,FOXP3,IL10 |
| DYSF | other | | 3,39E-03 | IFI16,ITGB1,MPEG1 |
| NfkB-RelA | complex | | 3,42E-03 | BCL2,IL10 |
| desipramine | chemical drug | | 3,42E-03 | BCL2,IL10 |
| lipoarabinomannan | chemical - endogenous non-mammalian | | 3,42E-03 | CAMP,ITGA7 |
| cannabidiol | chemical drug | | 3,42E-03 | GPR55,SOCS3 |
| cyclic AMP | chemical - endogenous mammalian | -1,91 | 3,44E-03 | BCL2,CD86,CTLA4,IL10,SOCS3 |
| troglitazone | chemical drug | 1,478 | 3,55E-03 | BCL2,C3,CD86,HP,IFI16,NOTCH3 |
| LDL | complex | | 3,56E-03 | BCL2,C3,CX3CR1,FOSB,SOCS3 |
| NR4A2 | ligand-dependent nuclear receptor | | 3,64E-03 | CD86,FOXP3,IL10 |
| risperidone | chemical drug | | 3,72E-03 | BCL2,IL10 |
| Ciap | group | | 3,72E-03 | BCL2,IL10 |
| IPMK | kinase | | 3,72E-03 | FOSB,IL10 |
| GRHL2 | transcription regulator | | 3,72E-03 | BCL2,NOTCH3 |
| MAP3K3 | kinase | | 3,72E-03 | BCL2,IL10 |
| PLD1 | enzyme | | 3,72E-03 | BCL2,PROM1 |
| diosgenin | chemical - endogenous non-mammalian | | 3,72E-03 | ALOX5,BCL2 |
| Cd64 | group | | 3,80E-03 | IL10 |
| N(6)-methyl-2'-deoxyadenosine | chemical reagent | | 3,80E-03 | SOCS3 |
| ALG10 | enzyme | | 3,80E-03 | PROM1 |

| | | | | |
|---|---|---|---|---|
| RNY4 | other | | 3,80E-03 | IL10 |
| hexacosanoyl-coenzyme A | chemical - endogenous non-mammalian | | 3,80E-03 | ALOX5 |
| sodium thiosulfate | chemical drug | | 3,80E-03 | BCL2 |
| MOGS | enzyme | | 3,80E-03 | PROM1 |
| RGN | enzyme | | 3,80E-03 | BCL2 |
| ALG8 | enzyme | | 3,80E-03 | PROM1 |
| TSSK3 | kinase | | 3,80E-03 | IL10 |
| LMAN1 | other | | 3,80E-03 | F5 |
| Ctf2 | cytokine | | 3,80E-03 | SOCS3 |
| ALG6 | enzyme | | 3,80E-03 | PROM1 |
| CSGALNACT1 | enzyme | | 3,80E-03 | IL10 |
| Flicr | other | | 3,80E-03 | FOXP3 |
| ALG5 | enzyme | | 3,80E-03 | PROM1 |
| GPR87 | G-protein coupled receptor | | 3,80E-03 | PROM1 |
| PX-866 | chemical drug | | 3,80E-03 | SORT1 |
| Psg18 (includes others) | other | | 3,80E-03 | IL10 |
| METAP2 | peptidase | | 3,80E-03 | BCL2 |
| MGAT4C | enzyme | | 3,80E-03 | PROM1 |
| SYT11 | transporter | | 3,80E-03 | IL10 |
| Mannosidase2 | group | | 3,80E-03 | PROM1 |
| voxtalisib | chemical drug | | 3,80E-03 | BCL2 |
| NTHL1 | enzyme | | 3,80E-03 | IL10 |
| EHD1 | other | | 3,80E-03 | ITGB1 |
| NAPA | transporter | | 3,80E-03 | BCL2 |
| AFAP1 | other | | 3,80E-03 | ITGB1 |
| TNF-kinoid | biologic drug | | 3,80E-03 | CTLA4 |
| CFI | peptidase | | 3,80E-03 | C3 |
| miR-466l-3p (miRNAs w/seed AUAAAUA) | mature microRNA | | 3,80E-03 | IL10 |
| mir-490 | microRNA | | 3,80E-03 | FOXP3 |
| MIR4281 | microRNA | | 3,80E-03 | FOXP3 |
| DYNLT3 | other | | 3,80E-03 | BCL2 |
| PROTOR | group | | 3,80E-03 | FOXP3 |
| adavosertib | chemical drug | | 3,80E-03 | IL10 |
| Trib2 | other | | 3,80E-03 | IL10 |
| Clec4b2 | other | | 3,80E-03 | IL10 |
| TM7SF2 | enzyme | | 3,80E-03 | NOTCH3 |
| PTCRA | other | | 3,80E-03 | NOTCH3 |
| RIT1 | enzyme | | 3,80E-03 | BCL2 |
| VPS18 | transporter | | 3,80E-03 | ITGB1 |
| TPO | enzyme | | 3,80E-03 | FOXP3 |
| CFD | peptidase | | 3,80E-03 | C3 |
| ShK-186 peptide | biologic drug | | 3,80E-03 | IL10 |
| TPD52 | other | | 3,80E-03 | BCL2 |
| ZNF433 | transcription regulator | | 3,80E-03 | ALOX5 |
| HG-9-88-01 | chemical reagent | | 3,80E-03 | IL10 |
| HG-10-7-01 | chemical reagent | | 3,80E-03 | IL10 |
| HG-10-11-01 | chemical reagent | | 3,80E-03 | IL10 |
| HG-9-148-01 | chemical reagent | | 3,80E-03 | IL10 |
| HG-9-150-01 | chemical reagent | | 3,80E-03 | IL10 |
| CGP 52608 | chemical reagent | | 3,80E-03 | ALOX5 |
| pubchem compound 1117216 | chemical reagent | | 3,80E-03 | IL10 |
| nafoxidine | chemical drug | | 3,80E-03 | C3 |
| melarsoprol | chemical drug | | 3,80E-03 | BCL2 |
| PF 956980 | chemical reagent | | 3,80E-03 | SORT1 |
| AZ10606120 | chemical reagent | | 3,80E-03 | CD86 |
| SH-4-54 | chemical reagent | | 3,80E-03 | FOXP3 |
| SDM25N | chemical reagent | | 3,80E-03 | CD86 |
| AuNP@PEG@e14a2 | chemical reagent | | 3,80E-03 | BCL2 |
| (S)-ethyl 6-chloro-3-(3,7-dimethyloctanoyl)-1H-indole-2-carboxylate | chemical reagent | | 3,80E-03 | IL10 |
| 18-hydroxy-retinoic acid | chemical - endogenous mammalian | | 3,80E-03 | ITGAX |
| cytisine | chemical drug | | 3,80E-03 | FOSB |
| 6alpha-fluorotestosterone | chemical toxicant | | 3,80E-03 | BCL2 |
| ESR2 | ligand-dependent nuclear receptor | 0,7 | 3,81E-03 | ARNT2,BCL2,C3,CPM,ITGB1,PROM1,SOCS3 |
| vorinostat | chemical drug | 0,651 | 3,85E-03 | BCL2,CAMP,IL10,SOCS3,SPI1 |
| ethanol | chemical - endogenous mammalian | -0,06 | 4,02E-03 | ALOX5,BCL2,FOSB,IL10,ITGAX,PDCD1 |
| CFTR | ion channel | | 4,03E-03 | LRG1,SLFN12L,SOCS3 |
| BCL2L11 | other | | 4,03E-03 | BCL2,IL10 |
| NMU | other | | 4,03E-03 | FOSB,IL10 |
| Gm21596/Hmgb1 | transcription regulator | | 4,03E-03 | IL10,PROM1 |
| PTH | other | | 4,11E-03 | BCL2,FOSB,SOCS3,SORT1 |
| 3,4,5,3',4'-pentachlorobiphenyl | chemical toxicant | | 4,16E-03 | CTLA4,H2-T10,ITGA7 |
| SPI1 | transcription regulator | -1,34 | 4,28E-03 | CEBPE,IL10,LTF,PRG2,SPI1 |
| IL6R | transmembrane receptor | | 4,30E-03 | HP,IL10,SOCS3 |
| LYN | kinase | | 4,30E-03 | BCL2,CD86,SOCS3 |
| amphetamine | chemical drug | | 4,30E-03 | BCL2,FOSB,IL10 |
| resolvin E1 | chemical - endogenous mammalian | | 4,36E-03 | CAMP,IL10 |
| vemurafenib | chemical drug | | 4,36E-03 | IL10,PDCD1 |
| TNFSF4 | cytokine | | 4,36E-03 | IL10,PDCD1 |
| MTA3 | transcription regulator | | 4,36E-03 | SYK,ZEB2 |
| CARD11 | kinase | | 4,36E-03 | IL10,SPI1 |
| HSPD1 | enzyme | | 4,36E-03 | IL10,SOCS3 |

| | | | | | |
|---|---|---|---|---|---|
| diazoxide | chemical drug | | | 4,36E-03 | BCL2,KCNJ8 |
| curdlan | chemical - endogenous non-mammalian | | | 4,36E-03 | FOXP3,IL10 |
| BMP7 | growth factor | | | 4,46E-03 | LTF,PRDM16,SOCS3,ZEB2 |
| dextran sulfate | chemical drug | | -0,772 | 4,54E-03 | C3,CD177,CD86,IL10,SOCS3 |
| AREG | growth factor | | | 4,59E-03 | BCL2,C3,FOXP3 |
| mir-21 | microRNA | | 0,6 | 4,68E-03 | BCL2,FOXP3,GBP3,IFI16,IL12RB2 |
| STAT5B | transcription regulator | | -0,068 | 4,68E-03 | BCL2,FOXP3,PROM1,SOCS3,TOX |
| lithocholic acid | chemical - endogenous mammalian | | | 4,70E-03 | BCL2,CAMP |
| CTSS | peptidase | | | 4,70E-03 | ITGA7,ITGB1 |
| ICOSLG/LOC102723996 | other | | | 4,70E-03 | FOXP3,IL10 |
| LTF | peptidase | | | 4,70E-03 | CD86,IL10 |
| DPP4 | peptidase | | | 4,70E-03 | CD86,IL10 |
| PTPN22 | phosphatase | | | 4,70E-03 | IL10,PDCD1 |
| NLRP3 | other | | | 4,73E-03 | BCL2,FOXP3,IL10 |
| RHOA | enzyme | | | 4,88E-03 | BCL2,ITGB1,Nedd4 |
| STAT5A | transcription regulator | | | 4,96E-03 | BCL2,FOSB,IL12RB2,S1PR5,SOCS3 |
| TRAP1 | enzyme | | | 5,04E-03 | BCL2,ITGB1,SYK |
| ZFP36 | transcription regulator | | | 5,04E-03 | CAMP,IL10,LTF |
| CCL2 | cytokine | | | 5,04E-03 | BCL2,CD86,IL10 |
| POU2F2 | transcription regulator | | | 5,04E-03 | ALOX5,IL10,SPI1 |
| 2-(4-acetoxyphenyl)-2-chloro-N-methylethylamine | chemical reagent | | | 5,05E-03 | FOXP3,IL10 |
| CD47 | transmembrane receptor | | | 5,05E-03 | IL10,IL12RB2 |
| JAK inhibitor I | chemical - kinase inhibitor | | | 5,05E-03 | C3,SOCS3 |
| WNT3A | cytokine | Inhibited | -2,236 | 5,10E-03 | ADAMTS14,BCL2,IKZF2,IL10,RFLNB |
| DNMT1 | enzyme | | | 5,19E-03 | FOXP3,IL10,PRDM16 |
| OGA | enzyme | | -0,816 | 5,34E-03 | BCL2,CD86,CX3CR1,PROM1,SLFN12L,SOCS3 |
| sirolimus | chemical drug | | 0,739 | 5,35E-03 | BCL2,CD86,CTLA4,FOXP3,IL10,PROM1,SOCS3 |
| DLL1 | enzyme | | | 5,41E-03 | CD86,IL10 |
| ELF1 | transcription regulator | | | 5,41E-03 | IL10,SPI1 |
| stearic acid | chemical - endogenous mammalian | | | 5,41E-03 | BCL2,IL10 |
| CALCA | other | | | 5,51E-03 | CD86,SORT1,SPI1 |
| KMT2A | transcription regulator | | | 5,51E-03 | BCL2,PRDM16,PROM1 |
| SOX4 | transcription regulator | | 0,218 | 5,63E-03 | BCL2,SORT1,SPI1,STAG3 |
| glucocorticoid | chemical drug | | 0,97 | 5,63E-03 | ALOX5,BCL2,FOXP3,IL10,SOCS3 |
| HTT | transcription regulator | | | 5,75E-03 | BCL2,GAS7,IFI16,ITGB1,LTF,MPEG1,PROM1,SORT1 |
| ARRB1 | other | | | 5,78E-03 | BCL2,CAMP |
| LTB4R | G-protein coupled receptor | | | 5,78E-03 | ALOX5,IL10 |
| bardoxolone methyl | chemical drug | | | 5,78E-03 | BCL2,IL10 |
| CD36 | transmembrane receptor | | | 5,84E-03 | CD86,IL10,SYK |
| SMAD2 | transcription regulator | | | 6,02E-03 | CRISPLD2,FOXP3,ITGB1 |
| CARD9 | other | | | 6,16E-03 | C3,IL10 |
| TIRAP | other | | | 6,16E-03 | CD86,IL10 |
| MAPK11 | kinase | | | 6,16E-03 | BCL2,IL10 |
| ZBTB48 | transcription regulator | | | 6,16E-03 | CPM,FOSB |
| clodronic acid | chemical drug | | | 6,16E-03 | CD86,IL10 |
| CEBPB | transcription regulator | Inhibited | -2,391 | 6,17E-03 | BCL2,C3,HP,IL10,PRG2,SOCS3 |
| mir-8 | microRNA | | 0,561 | 6,17E-03 | BCL2,ITGB1,PROM1,ZEB2 |
| bee venom | chemical - endogenous non-mammalian | | | 6,19E-03 | FOSB,IL10,SOCS3 |
| FOS | transcription regulator | | -1,953 | 6,34E-03 | C3,CAMP,FOSB,IFI16,IL10,ITGB1,SOCS3 |
| Ifn gamma | complex | | | 6,36E-03 | CD86,IL10,SOCS3 |
| Pdgf (complex) | complex | | | 6,54E-03 | BCL2,FOSB,NOTCH3 |
| TRB | transmembrane receptor | | | 6,56E-03 | BCL2,IL10 |
| UXT | transcription regulator | | | 6,56E-03 | CTLA4,F5 |
| hymecromone | chemical drug | | | 6,56E-03 | BCL2,FOXP3 |
| bleomycin | chemical drug | | -0,469 | 6,63E-03 | BCL2,C3,CD86,ITGB1 |
| uric acid | chemical - endogenous mammalian | | | 6,73E-03 | BCL2,CD86,IL10 |
| TREM1 | transmembrane receptor | | -0,976 | 6,87E-03 | BCL2,CD86,IL10,ITGAX |
| 3M-011 | chemical reagent | | | 6,96E-03 | BCL2,CD86 |
| IL23A | cytokine | | | 6,96E-03 | HP,IL10 |
| ALOX15 | enzyme | | | 6,96E-03 | BCL2,NOTCH3 |
| CR1L | other | | | 6,96E-03 | C3,IL10 |
| VTN | other | | | 6,96E-03 | BCL2,ITGB1 |
| NR1H2 | ligand-dependent nuclear receptor | | | 7,10E-03 | C3,ITGAX,PRDM16 |
| CXCL12 | cytokine | | | 7,11E-03 | BCL2,ITGAX,ITGB1,SOCS3 |
| NRAS | enzyme | | | 7,11E-03 | BCL2,CD86,CX3CR1,IFI16 |
| EDN1 | cytokine | | 0,061 | 7,11E-03 | BCL2,FOSB,ITGB1,SOCS3 |
| TGFBR2 | kinase | | 0,655 | 7,11E-03 | ALOX5,IL10,KCNJ8,SOCS3 |
| GW501516 | chemical drug | | | 7,29E-03 | BCL2,CAMP,LTF |
| Rar | group | | | 7,38E-03 | BCL2,CEBPE |
| oleanolic acid | chemical - endogenous non-mammalian | | | 7,38E-03 | ITGB1,SOCS3 |
| Secretase gamma | complex | | | 7,38E-03 | CD86,IL10 |
| SKP2 | other | | | 7,38E-03 | FOXP3,ZEB2 |
| EZR | other | | | 7,38E-03 | BCL2,IL10 |
| mir-126 | microRNA | | | 7,38E-03 | FOXP3,SPI1 |
| CDC42 | enzyme | | | 7,38E-03 | CD86,ITGB1 |
| bryostatin 1 | chemical drug | | | 7,38E-03 | CD86,FOXP3 |
| mir-10 | microRNA | | | 7,48E-03 | ALOX5,CD86,PROM1 |
| amikacin | chemical drug | | | 7,58E-03 | BCL2 |
| MICU1 | other | | | 7,58E-03 | BCL2 |
| interferon gamma-1b | biologic drug | | | 7,58E-03 | IL10 |
| epratuzumab | biologic drug | | | 7,58E-03 | IL10 |
| Cofilin | group | | | 7,58E-03 | IL10 |
| AEOL-10150 | chemical drug | | | 7,58E-03 | BCL2 |

| | | | | |
|---|---|---|---|---|
| Tgf beta receptor | complex | | 7,58E-03 | FOXP3 |
| Rbp | group | | 7,58E-03 | SOCS3 |
| thymol | chemical reagent | | 7,58E-03 | BCL2 |
| Calcb | other | | 7,58E-03 | SPI1 |
| GSDMB | other | | 7,58E-03 | ALOX5 |
| PCDH9 | other | | 7,58E-03 | BCL2 |
| TRIB2 | kinase | | 7,58E-03 | IL10 |
| PIBF1 | other | | 7,58E-03 | IL10 |
| TLN2 | other | | 7,58E-03 | ITGB1 |
| ZNF366 | transcription regulator | | 7,58E-03 | IL10 |
| BCL6 peptide inhibitor | chemical drug | | 7,58E-03 | BCL2 |
| alvelestat | chemical drug | | 7,58E-03 | IL10 |
| bucillamine | chemical drug | | 7,58E-03 | BCL2 |
| Fcer | group | | 7,58E-03 | IL10 |
| CACYBP | other | | 7,58E-03 | BCL2 |
| safflor yellow B | chemical - endogenous non-mammalian | | 7,58E-03 | BCL2 |
| NOSIP | other | | 7,58E-03 | BCL2 |
| CAMKK1 | kinase | | 7,58E-03 | IL10 |
| STXBP4 | other | | 7,58E-03 | ITGB1 |
| SYCP3 | other | | 7,58E-03 | BCL2 |
| huo-luo-xiao-ling dan | chemical drug | | 7,58E-03 | IL10 |
| AKAP10 | other | | 7,58E-03 | IL10 |
| mir-153 | microRNA | | 7,58E-03 | BCL2 |
| mir-3473 | microRNA | | 7,58E-03 | IL10 |
| PRG2 | other | | 7,58E-03 | IL10 |
| HIF1A-AS1 | other | | 7,58E-03 | BCL2 |
| PIM | group | | 7,58E-03 | BCL2 |
| STK38L | kinase | | 7,58E-03 | ITGB1 |
| potassium nitrate | chemical drug | | 7,58E-03 | IL10 |
| REG3A | enzyme | | 7,58E-03 | SOCS3 |
| TUSC2 | other | | 7,58E-03 | IL10 |
| PLIN3 | other | | 7,58E-03 | BCL2 |
| AKT inhibitor VII | chemical reagent | | 7,58E-03 | IL10 |
| cyclo(iso-Asp-GR)-LLIIKLAKLAKKLAKLAK | chemical reagent | | 7,58E-03 | BCL2 |
| PLEKHA1 | other | | 7,58E-03 | CD86 |
| PTPRT | phosphatase | | 7,58E-03 | SOCS3 |
| NTSR2 | G-protein coupled receptor | | 7,58E-03 | BCL2 |
| PLEKHA2 | other | | 7,58E-03 | CD86 |
| ITGA7 | other | | 7,58E-03 | ITGB1 |
| NEU2 | enzyme | | 7,58E-03 | BCL2 |
| UBE2V1 | transcription regulator | | 7,58E-03 | BCL2 |
| Gm4836 (includes others) | other | | 7,58E-03 | BCL2 |
| VPS26B | transporter | | 7,58E-03 | SORT1 |
| VNN1 | enzyme | | 7,58E-03 | BCL2 |
| mycobacterium smegmatis lipoarabinomannan | chemical - endogenous non-mammalian | | 7,58E-03 | IL10 |
| 3830403N18Rik/Xlr | other | | 7,58E-03 | BCL2 |
| ERH | other | | 7,58E-03 | SOCS3 |
| NIBR-14 | chemical reagent | | 7,58E-03 | IL10 |
| LY3039478 | chemical drug | | 7,58E-03 | PROM1 |
| P144 | biologic drug | | 7,58E-03 | IL10 |
| S7 | chemical - kinase inhibitor | | 7,58E-03 | BCL2 |
| SI163 | chemical - kinase inhibitor | | 7,58E-03 | BCL2 |
| S29 | chemical - kinase inhibitor | | 7,58E-03 | BCL2 |
| kanamycin A | chemical drug | | 7,58E-03 | SORT1 |
| trimellitic anhydride | chemical toxicant | | 7,58E-03 | IL10 |
| daphnoretin | chemical - endogenous non-mammalian | | 7,58E-03 | BCL2 |
| ephedrine | chemical drug | | 7,58E-03 | IL10 |
| XK469 | chemical drug | | 7,58E-03 | BCL2 |
| talipexole | chemical drug | | 7,58E-03 | BCL2 |
| AN-207 | chemical toxicant | | 7,58E-03 | BCL2 |
| potassium superoxide | chemical toxicant | | 7,58E-03 | IL12RB2 |
| phenyl butyrate | chemical reagent | | 7,58E-03 | CAMP |
| SSR180575 | chemical drug | | 7,58E-03 | BCL2 |
| trichosanthin | chemical drug | | 7,58E-03 | IL10 |
| pSTAT3 (Tyr705) inhibitory peptide | chemical reagent | | 7,58E-03 | IL10 |
| all-trans-4-Oxo-retinoic acid | chemical - endogenous mammalian | | 7,58E-03 | ITGAX |
| isoalantolactone | chemical - endogenous non-mammalian | | 7,58E-03 | BCL2 |
| swainsonine | chemical - endogenous non-mammalian | | 7,58E-03 | BCL2 |
| epoxyeicosatrienoic acid | chemical - other | | 7,58E-03 | ALOX5 |
| 3-hydroxykynurenine | chemical - endogenous mammalian | | 7,58E-03 | FOXP3 |
| ELAVL1 | other | -0,762 | 7,74E-03 | CD86,IFI16,MPEG1,PROM1 |
| ITGAM | transmembrane receptor | | 7,81E-03 | CD86,IL10 |
| ABCG1 | transporter | | 7,81E-03 | IL10,IL12RB2 |
| CYP27B1 | enzyme | | 7,81E-03 | CTLA4,IL10 |
| GLI2 | transcription regulator | | 7,88E-03 | BCL2,IL10,ITGB1 |
| ARNT2 | transcription regulator | -1 | 8,01E-03 | ARNT2,CD86,IFI16,LTF |
| EIF4E | translation regulator | | 8,01E-03 | BCL2,CX3CR1,IFI16,PRG2 |
| BCL2 | transporter | | 8,08E-03 | BCL2,IL10,ITGB1 |
| Ap2 | group | | 8,25E-03 | BCL2,C3 |

| | | | | | |
|---|---|---|---|---|---|
| PF4 | cytokine | | | 8,25E-03 | CD86,CPM |
| rolipram | chemical drug | | | 8,25E-03 | CD86,IL10 |
| PPARA | ligand-dependent nuclear receptor | | 1,128 | 8,36E-03 | ALDH2,BCL2,C3,H2-T10,IL10,PRDM16 |
| CD44 | other | | 1,165 | 8,41E-03 | BCL2,CX3CR1,IL10,ITGAX |
| BMP2 | growth factor | | 1,067 | 8,41E-03 | BCL2,FOSB,FOXP3,IFI16 |
| camptothecin | chemical drug | | | 8,48E-03 | BCL2,CD86,F5,FOSB,IFI16,ITGA7,SYK |
| NR5A2 | ligand-dependent nuclear receptor | | | 8,50E-03 | BCL2,C3,HP |
| berberine | chemical drug | | | 8,50E-03 | BCL2,CD86,IL10 |
| APOE | transporter | | | 8,66E-03 | CD86,GAS7,IL10,ITGAX,SOCS3 |
| 3M-001 | chemical drug | | | 8,70E-03 | BCL2,CD86 |
| RNASE2 | enzyme | | | 8,70E-03 | CD86,IL10 |
| IKZF2 | transcription regulator | | | 8,70E-03 | FOXP3,IL10 |
| CD80 | transmembrane receptor | | | 8,70E-03 | IL10,PDCD1 |
| KEAP1 | transcription regulator | | | 8,70E-03 | BCL2,IL10 |
| peptidoglycan | chemical - endogenous non-mammalian | | | 8,71E-03 | CD86,FOXP3,IL10 |
| ciprofloxacin | chemical drug | | | 8,71E-03 | ALDH2,CD86,MPEG1 |
| SIM1 | transcription regulator | | -1 | 8,83E-03 | ARNT2,CD86,IFI16,LTF |
| SYK | kinase | | | 8,93E-03 | BCL2,IL10,PDCD1 |
| simvastatin | chemical drug | | -0,218 | 8,98E-03 | BCL2,FOXP3,IL10,SOCS3 |
| ANGPT2 | growth factor | | -0,52 | 9,12E-03 | BCL2,CD86,IL10,ITGAX |
| PI3K (family) | group | | | 9,15E-03 | BCL2,IL10,ITGB1 |
| 3-methyladenine | chemical toxicant | | | 9,16E-03 | BCL2,CD86 |
| NSD2 | enzyme | | | 9,16E-03 | BCL2,ITGB1 |
| TSLP | cytokine | | | 9,16E-03 | CD86,IL10 |
| NQO1 | enzyme | | | 9,16E-03 | BCL2,SPI1 |
| SAA1 | transporter | | | 9,16E-03 | BCL2,IL10 |
| TLR5 | transmembrane receptor | | | 9,16E-03 | CD86,IL10 |
| NR2F2 | ligand-dependent nuclear receptor | | | 9,16E-03 | ALDH2,LTF |
| PAK2 | kinase | | | 9,16E-03 | IFI16,ZEB2 |
| cilostazol | chemical drug | | | 9,16E-03 | BCL2,IL10 |
| Jnk | group | | | 9,27E-03 | CD86,FOSB,IL10,SOCS3 |
| Rxr | group | | | 9,37E-03 | BCL2,CEBPE,LTF |
| TGFB3 | growth factor | | | 9,59E-03 | BCL2,FOSB,ZEB2 |
| calcimycin | chemical reagent | | | 9,59E-03 | CD86,CTLA4,FOSB |
| JAG2 | growth factor | | | 9,63E-03 | C3,NOTCH3 |
| NR2F1 | ligand-dependent nuclear receptor | | | 9,63E-03 | ALDH2,LTF |
| TNFRSF9 | transmembrane receptor | | | 9,63E-03 | BCL2,CD86 |
| pristane | chemical toxicant | | | 9,63E-03 | CD86,IFI16 |
| FOXO3 | transcription regulator | | 0,9 | 9,65E-03 | BCL2,FOSB,FOXP3,IKZF2,IL10 |
| Tlr | group | | | 1,01E-02 | CD86,IL10,SOCS3 |
| RAC1 | enzyme | | | 1,01E-02 | CD86,IL10,ITGB1 |
| CEACAM1 | transporter | | | 1,01E-02 | CD86,NOTCH3 |
| Cdc42 | enzyme | | | 1,01E-02 | CD86,SPI1 |
| SMARCA4 | transcription regulator | | -1,673 | 1,03E-02 | ALDH2,BCL2,CPM,IFI16,ITGA7,ITGB1,SYK |
| JQ1 | chemical reagent | | | 1,05E-02 | BCL2,FOXP3,IL10 |
| 1,4-bis[2-(3,5-dichloropyridyloxy)]benzene | chemical toxicant | | | 1,05E-02 | CKAP4,LRG1,Nedd4 |
| cisplatin | chemical drug | | | 1,06E-02 | BCL2,DMXL2,ITGA7,ITGB1,NOTCH3,PRG2,PROM1,SOCS3 |
| conjugated linoleic acid | chemical drug | | | 1,06E-02 | IL10,SOCS3 |
| TLR7/8 | group | | | 1,06E-02 | BCL2,IL10 |
| eflornithine | chemical drug | | | 1,06E-02 | C3,ITGAX |
| IL1 | group | Inhibited | -2,195 | 1,08E-02 | BCL2,C3,HP,IL10,SOCS3 |
| Ifn | group | | | 1,10E-02 | CD86,IFI16,IL10 |
| GW3965 | chemical reagent | | | 1,10E-02 | CD86,CX3CR1,SOCS3 |
| NKX2-5 | transcription regulator | | | 1,11E-02 | BCL2,IFI16 |
| FASLG | cytokine | | | 1,11E-02 | BCL2,IL10 |
| fasudil | chemical drug | | | 1,11E-02 | BCL2,IL10 |
| S-nitrosoglutathione | chemical toxicant | | | 1,11E-02 | CD86,FOXP3 |
| ribavirin | chemical drug | | | 1,11E-02 | CD86,IL10 |
| ARNT | transcription regulator | | | 1,13E-02 | BCL2,IL10,SOCS3 |
| GSK3B | kinase | | | 1,13E-02 | BCL2,IL10,PDCD1 |
| sulfatides | chemical - endogenous mammalian | | | 1,13E-02 | IL10 |
| L-tyrosine | chemical - endogenous mammalian | | | 1,13E-02 | BCL2 |
| adalimumab | biologic drug | | | 1,13E-02 | CD86 |
| galactosylceramide | chemical - endogenous mammalian | | | 1,13E-02 | CD86 |
| atypical protein kinase C | group | | | 1,13E-02 | IL10 |
| lipoxygenase | group | | | 1,13E-02 | IL10 |
| sethoxydim | chemical toxicant | | | 1,13E-02 | BCL2 |
| clobetasol | chemical drug | | | 1,13E-02 | CAMP |
| lipopeptide | chemical - endogenous non-mammalian | | | 1,13E-02 | IL10 |
| INTS11 | other | | | 1,13E-02 | FOSB |
| SLC48A1 | transporter | | | 1,13E-02 | ITGB1 |
| NOP58 | enzyme | | | 1,13E-02 | LTF |
| NIPBL | transcription regulator | | | 1,13E-02 | FOXP3 |
| IL10R | group | | | 1,13E-02 | SOCS3 |
| NLRP10 | other | | | 1,13E-02 | ALOX5 |
| hyperoside | chemical - endogenous non-mammalian | | | 1,13E-02 | BCL2 |
| KLF14 | transcription regulator | | | 1,13E-02 | FOXP3 |
| TMEM106A | other | | | 1,13E-02 | CD86 |
| STAB1 | transporter | | | 1,13E-02 | IL10 |
| LAPTM4B | other | | | 1,13E-02 | BCL2 |

| | | | | |
|---|---|---|---|---|
| Integrin alpha 4 beta 1 | complex | | 1,13E-02 | BCL2 |
| ESRP2 | other | | 1,13E-02 | ZEB2 |
| CKLF | cytokine | | 1,13E-02 | BCL2 |
| INAVA | other | | 1,13E-02 | IL10 |
| EPX | enzyme | | 1,13E-02 | IL10 |
| xenon | chemical drug | | 1,13E-02 | BCL2 |
| iron sucrose | chemical drug | | 1,13E-02 | IL10 |
| ACAT1 | enzyme | | 1,13E-02 | ITGB1 |
| GEM231 | chemical drug | | 1,13E-02 | BCL2 |
| cerebrolysin | chemical drug | | 1,13E-02 | BCL2 |
| CCR3 | G-protein coupled receptor | | 1,13E-02 | PRG2 |
| miR-136-5p (miRNAs w/seed CUCCAUU) | mature microRNA | | 1,13E-02 | BCL2 |
| miR-27a-5p (miRNAs w/seed GGGCUUA) | mature microRNA | | 1,13E-02 | CX3CR1 |
| mir-483 | microRNA | | 1,13E-02 | SOCS3 |
| miR-153-3p (miRNAs w/seed UGCAUAG) | mature microRNA | | 1,13E-02 | BCL2 |
| CD34 | other | | 1,13E-02 | IL10 |
| SIPA1 | other | | 1,13E-02 | ITGB1 |
| DDOST | enzyme | | 1,13E-02 | PROM1 |
| MLST8 | other | | 1,13E-02 | FOXP3 |
| CCL13 | cytokine | | 1,13E-02 | IL10 |
| IFNGR2 | transmembrane receptor | | 1,13E-02 | GBP3 |
| GFRA2 | transmembrane receptor | | 1,13E-02 | FOSB |
| DISC1 | other | | 1,13E-02 | ITGB1 |
| ANKRD1 | transcription regulator | | 1,13E-02 | BCL2 |
| CTAG1A/CTAG1B | other | | 1,13E-02 | PDCD1 |
| CCL8 | cytokine | | 1,13E-02 | IL10 |
| CCNL2 | other | | 1,13E-02 | BCL2 |
| MAP1S | enzyme | | 1,13E-02 | BCL2 |
| AP2A2 | transporter | | 1,13E-02 | BCL2 |
| IL20RA | transmembrane receptor | | 1,13E-02 | IL10 |
| SJN 2511 | chemical reagent | | 1,13E-02 | FOXP3 |
| PDCD5 | other | | 1,13E-02 | FOXP3 |
| MT3 | other | | 1,13E-02 | BCL2 |
| EXOC7 | transporter | | 1,13E-02 | ZEB2 |
| CHTOP | other | | 1,13E-02 | LTF |
| capivasertib | chemical drug | | 1,13E-02 | PROM1 |
| 4-ethylsulfanylphenylmethylene hydantoin | chemical reagent | | 1,13E-02 | ITGB1 |
| 4-hydroxyphenylmethylene hydantoin | chemical reagent | | 1,13E-02 | ITGB1 |
| C20-D3-vitamin A | chemical drug | | 1,13E-02 | C3 |
| IPI-549 | chemical drug | | 1,13E-02 | IL10 |
| URB597 | chemical drug | | 1,13E-02 | IL10 |
| chlordecone | chemical toxicant | | 1,13E-02 | LTF |
| pramipexole | chemical drug | | 1,13E-02 | BCL2 |
| ketotifen | chemical drug | | 1,13E-02 | IL10 |
| 1-heptanol | chemical reagent | | 1,13E-02 | CD86 |
| adapalene | chemical drug | | 1,13E-02 | IL10 |
| lead nitrate | chemical toxicant | | 1,13E-02 | BCL2 |
| imisopasem manganese | chemical drug | | 1,13E-02 | BCL2 |
| 18-alpha-glycyrrhetinic acid | chemical - endogenous non-mammalian | | 1,13E-02 | IL10 |
| 10-hydroxycamptothecin | chemical - endogenous mammalian | | 1,13E-02 | BCL2 |
| FOXA2 | transcription regulator | | 1,15E-02 | ALOX5,C3,CPM,IL10 |
| CLDN7 | other | | 1,15E-02 | C3,CKAP4,F5 |
| lithium chloride | chemical drug | | 1,15E-02 | BCL2,IL10,NOTCH3 |
| JAK | group | | 1,16E-02 | IL10,SOCS3 |
| RPS6KB1 | kinase | | 1,16E-02 | BCL2,IL10 |
| SELPLG | other | | 1,16E-02 | IL10,PDCD1 |
| IFNE | cytokine | | 1,16E-02 | CD86,IL10 |
| IRAK1 | kinase | | 1,16E-02 | FOXP3,IL10 |
| cinnamaldehyde | chemical toxicant | | 1,16E-02 | BCL2,PRDM16 |
| D-galactosamine | chemical - endogenous mammalian | | 1,16E-02 | BCL2,IL10 |
| 3M-002 | chemical reagent | | 1,18E-02 | CD86,ITGAX,SLFN12L |
| C5 | cytokine | | 1,18E-02 | BCL2,CD86,IL10 |
| HGF | growth factor | -0,34 | 1,19E-02 | BCL2,FOSB,IL10,ITGB1,SOCS3,TBC1D8 |
| Growth hormone | group | | 1,20E-02 | BCL2,C3,SOCS3,ZEB2 |
| ANGPT1 | growth factor | | 1,21E-02 | BCL2,ITGB1 |
| HBEGF | growth factor | | 1,21E-02 | BCL2,ITGB1 |
| ILK | kinase | | 1,21E-02 | BCL2,ITGB1 |
| TXN | enzyme | | 1,21E-02 | BCL2,CD86 |
| SST | other | | 1,21E-02 | IL10,TOX |
| STAT5a/b | group | | 1,23E-02 | BCL2,FOXP3,SOCS3 |
| WT1 | transcription regulator | | 1,23E-02 | BCL2,FOSB,IL10,NOTCH3 |
| ADCYAP1 | other | -1,634 | 1,25E-02 | BCL2,CD86,IL10,SOCS3 |
| cholesterol | chemical - endogenous mammalian | | 1,25E-02 | IL10,ITGAX,MPEG1,SOCS3 |
| HMOX1 | enzyme | | 1,25E-02 | BCL2,FOXP3,IL10 |
| 2-methoxyestradiol | chemical - endogenous mammalian | | 1,27E-02 | IL10,LTF |
| sodium bisulfide | chemical reagent | | 1,27E-02 | BCL2,IL10 |
| thymoquinone | chemical drug | | 1,27E-02 | BCL2,IL10 |
| HDAC6 | transcription regulator | | 1,27E-02 | BCL2,IL10 |
| PCGF2 | transcription regulator | | 1,27E-02 | NOTCH3,ZEB2 |

| | | | | |
|---|---|---|---|---|
| Ro31-8220 | chemical - kinase inhibitor | | 1,27E-02 | BCL2,ITGB1 |
| REL | transcription regulator | | 1,29E-02 | BCL2,CD86,FOXP3,PDCD1 |
| TO-901317 | chemical reagent | | 1,29E-02 | ALDH2,C3,HP,MPEG1 |
| IL23 | complex | | 1,32E-02 | BCL2,IL10 |
| EGR3 | transcription regulator | | 1,32E-02 | SOCS3,ZEB2 |
| mir-146 | microRNA | | 1,32E-02 | FOXP3,IL10 |
| IFNA4 | cytokine | | 1,32E-02 | CD86,JAML |
| ICOS | transmembrane receptor | | 1,32E-02 | CTLA4,IL10 |
| Pam3-Cys | chemical toxicant | | 1,32E-02 | CD86,IL10 |
| N-acetyl-L-cysteine | chemical drug | 0,686 | 1,32E-02 | BCL2,C3,CD86,IL10 |
| hydrogen peroxide | chemical - endogenous mammalian | 0,141 | 1,33E-02 | ALOX5,BCL2,CD86,IL10,Nedd4,NOTCH3 |
| WNT5A | cytokine | | 1,34E-02 | BCL2,IL10,SOCS3 |
| SPHK1 | kinase | | 1,38E-02 | IL10,SOCS3 |
| NOTCH2 | transcription regulator | | 1,38E-02 | BCL2,IL10 |
| KDR | kinase | | 1,38E-02 | CTLA4,PDCD1 |
| TCF3 | transcription regulator | | 1,38E-02 | CKAP4,FOXP3,IFI16,SOCS3,TOX |
| prednisolone | chemical drug | 1,732 | 1,42E-02 | BCL2,CD86,IL10,NOTCH3 |
| mir-223 | microRNA | | 1,42E-02 | ABCA13,IFI16,MMP25 |
| MEF2C | transcription regulator | | 1,42E-02 | FOSB,MMP25,SYK |
| FYN | kinase | | 1,43E-02 | FOXP3,SOCS3 |
| MYBL2 | transcription regulator | | 1,43E-02 | BCL2,ITGB1 |
| PRKD1 | kinase | | 1,43E-02 | CD86,IL10 |
| aldesleukin | biologic drug | | 1,43E-02 | BCL2,CD86 |
| IL33 | cytokine | -1,934 | 1,44E-02 | CD86,IL10,PRG2,SOCS3 |
| Hsp70 | group | | 1,49E-02 | BCL2,CD86 |
| baicalin | chemical - endogenous non-mammalian | | 1,49E-02 | BCL2,FOXP3 |
| IL11RA | transmembrane receptor | | 1,49E-02 | FOXP3,SOCS3 |
| IRF9 | transcription regulator | | 1,49E-02 | PDCD1,SOCS3 |
| MYOD1 | transcription regulator | -1,154 | 1,50E-02 | BCL2,IFI16,ITGA7,PRDM16 |
| behenic acid | chemical - endogenous mammalian | | 1,51E-02 | IL10 |
| cloprostenol | chemical drug | | 1,51E-02 | SOCS3 |
| OSGIN1 | growth factor | | 1,51E-02 | BCL2 |
| ENPP7 | enzyme | | 1,51E-02 | IL10 |
| LY75 | transmembrane receptor | | 1,51E-02 | CD86 |
| SOX12 | transcription regulator | | 1,51E-02 | FOXP3 |
| enecadin | chemical drug | | 1,51E-02 | BCL2 |
| cathepsin L inhibitor | chemical drug | | 1,51E-02 | C3 |
| BTN2A2 | other | | 1,51E-02 | FOXP3 |
| MITF-p300/CBP | complex | | 1,51E-02 | BCL2 |
| Fc receptor | group | | 1,51E-02 | IL10 |
| SERPING1 | other | | 1,51E-02 | IL10 |
| PF-4691502 | chemical drug | | 1,51E-02 | PROM1 |
| dabrafenib | chemical drug | | 1,51E-02 | IL10 |
| IL20RB | other | | 1,51E-02 | IL10 |
| MZB1 | other | | 1,51E-02 | CD86 |
| ARTN | growth factor | | 1,51E-02 | BCL2 |
| CHST4 | enzyme | | 1,51E-02 | IL10 |
| MS4A2 | transmembrane receptor | | 1,51E-02 | BCL2 |
| IFNGR | complex | | 1,51E-02 | SOCS3 |
| CPB2 | peptidase | | 1,51E-02 | IL10 |
| CDH17 | transporter | | 1,51E-02 | ITGB1 |
| LAMB1 | other | | 1,51E-02 | ITGB1 |
| HSPB6 | other | | 1,51E-02 | BCL2 |
| TRHR | G-protein coupled receptor | | 1,51E-02 | SOCS3 |
| RAPSN | other | | 1,51E-02 | SOCS3 |
| CANX | other | | 1,51E-02 | BCL2 |
| SIGMAR1 | transmembrane receptor | | 1,51E-02 | BCL2 |
| CELF2 | other | | 1,51E-02 | BCL2 |
| CACNA1S | ion channel | | 1,51E-02 | IL10 |
| IRAK1BP1 | other | | 1,51E-02 | IL10 |
| graptopetalum paraguayense methanol extract | chemical - endogenous non-mammalian | | 1,51E-02 | IL10 |
| CHST2 | enzyme | | 1,51E-02 | IL10 |
| LGALS2 | other | | 1,51E-02 | BCL2 |
| TSN | other | | 1,51E-02 | BCL2 |
| RPS6KA1 | kinase | | 1,51E-02 | IL10 |
| CD300LD | other | | 1,51E-02 | CD300LF |
| CXCL9 | cytokine | | 1,51E-02 | IL10 |
| PALLD | other | | 1,51E-02 | ITGB1 |
| Trem2 | other | | 1,51E-02 | IL10 |
| WTAP | other | | 1,51E-02 | BCL2 |
| NSC719239 | chemical drug | | 1,51E-02 | ITGB1 |
| TAS-103 | chemical drug | | 1,51E-02 | BCL2 |
| ascomycin | chemical reagent | | 1,51E-02 | CD86 |
| pyridoxal phosphate | chemical - endogenous mammalian | | 1,51E-02 | BCL2 |
| hemicholinium 3 | chemical reagent | | 1,51E-02 | IL10 |
| cromolyn | chemical drug | | 1,51E-02 | IL10 |
| chlorophyll a | chemical - endogenous non-mammalian | | 1,51E-02 | BCL2 |
| CCI-007 | chemical reagent | | 1,51E-02 | BCL2 |
| BMS336 | chemical reagent | | 1,51E-02 | IL10 |
| palmatine | chemical toxicant | | 1,51E-02 | IL10 |
| galanthamine | chemical drug | | 1,51E-02 | BCL2 |
| erucic acid | chemical - endogenous non-mammalian | | 1,51E-02 | ALOX5 |
| 1-methyl-DL-tryptophan | chemical drug | | 1,51E-02 | IL10 |

| | | | | |
|---|---|---|---|---|
| LY294002 | chemical - kinase inhibitor | 1,525 | 1,52E-02 | BCL2,CD86,FOSB,IL10,SOCS3,SYK |
| MAPT | other | | 1,53E-02 | C3,DMXL2,GAS7,PDE2A,SRGAP3 |
| AURK | group | | 1,55E-02 | PDE2A,SRGAP3 |
| RETN | other | | 1,55E-02 | CX3CR1,SOCS3 |
| KAT2B | transcription regulator | | 1,55E-02 | FOSB,FOXP3 |
| ANLN | other | | 1,55E-02 | PDE2A,SRGAP3 |
| IFN type 1 | group | | 1,61E-02 | BCL2,IFI16 |
| PPARD | ligand-dependent nuclear receptor | 1,131 | 1,62E-02 | ALDH2,BCL2,IL10,MPEG1 |
| IRF8 | transcription regulator | | 1,66E-02 | CD86,CEBPE,ZEB2 |
| triptolide | chemical drug | | 1,66E-02 | BCL2,IL10,SOCS3 |
| SRC (family) | group | | 1,67E-02 | IL10,PROM1 |
| PRMT1 | enzyme | | 1,67E-02 | BCL2,ZEB2 |
| ADRB2 | G-protein coupled receptor | | 1,67E-02 | CD86,IL10 |
| IRF6 | transcription regulator | | 1,67E-02 | CD86,IL10 |
| 2,3,4,7,8-pentachlorodibenzofuran | chemical toxicant | | 1,67E-02 | H2-T10,ITGA7 |
| MYC | transcription regulator | -0,971 | 1,69E-02 | ALOX5,BCL2,CD86,CEBPE,GBP3,IFI16,IL10,ITGAX,ITGB1,PROM1 |
| ESR1 | ligand-dependent nuclear receptor | -0,7 | 1,70E-02 | ALOX5,BCL2,C3,CPM,FOSB,FOXP3,GAS7,LTF,MAP3K9,NOTCH3,PROM1 |
| CREBBP | transcription regulator | | 1,71E-02 | BCL2,CRISPLD2,FOSB,IL10,SOCS3 |
| MTORC1 | complex | | 1,73E-02 | FOXP3,Nedd4 |
| BAX | transporter | | 1,73E-02 | BCL2,H2-T10 |
| sulforafan | chemical drug | | 1,75E-02 | BCL2,HP,SOCS3 |
| platelet activating factor | chemical - endogenous mammalian | | 1,79E-02 | IL10,SOCS3 |
| IL4R | transmembrane receptor | | 1,79E-02 | IL12RB2,SOCS3 |
| hydrocortisone | chemical - endogenous mammalian | | 1,82E-02 | CAMP,CD86,IL10 |
| lovastatin | chemical drug | | 1,82E-02 | BCL2,CD86,IL10 |
| TCF4 | transcription regulator | -1,342 | 1,85E-02 | CKAP4,GBP3,IFI16,SOCS3,ZEB2 |
| oblimersen | biologic drug | | 1,86E-02 | BCL2,IFI16 |
| TSHZ3 | transcription regulator | | 1,86E-02 | FOSB,PDE2A |
| IL2RG | transmembrane receptor | | 1,86E-02 | BCL2,FOXP3 |
| anisomycin | chemical - endogenous non-mammalian | | 1,86E-02 | BCL2,IL10 |
| 3-methylcholanthrene | chemical toxicant | | 1,86E-02 | HP,IL10 |
| bicuculline | chemical - endogenous non-mammalian | | 1,86E-02 | FOSB,Nedd4 |
| eritoran | chemical drug | | 1,88E-02 | IL10 |
| p38 Sapk | group | | 1,88E-02 | CD86 |
| ILX-23-7553 | chemical drug | | 1,88E-02 | BCL2 |
| [Lys15,Arg16,Leu27]VIP(1-7)GRF(8-27) | chemical reagent | | 1,88E-02 | CD86 |
| KLK8 | peptidase | | 1,88E-02 | IL10 |
| saxagliptin | chemical drug | | 1,88E-02 | ITGAX |
| Alp | group | | 1,88E-02 | BCL2 |
| (-)-gossypol | chemical drug | | 1,88E-02 | BCL2 |
| linseed oil | chemical reagent | | 1,88E-02 | SYK |
| W146 | chemical reagent | | 1,88E-02 | IL10 |
| RAR-RXR | complex | | 1,88E-02 | PRDM16 |
| CPT1 | group | | 1,88E-02 | PROM1 |
| CYP | group | | 1,88E-02 | CD86 |
| lupeol | chemical drug | | 1,88E-02 | PROM1 |
| acyline | biologic drug | | 1,88E-02 | BCL2 |
| Trbv13-1 | other | | 1,88E-02 | IL10 |
| propolis | biologic drug | | 1,88E-02 | IL10 |
| RUBCN | other | | 1,88E-02 | IL10 |
| sGC | complex | | 1,88E-02 | FOXP3 |
| UTP18 | other | | 1,88E-02 | BCL2 |
| C1GALT1 | enzyme | | 1,88E-02 | BCL2 |
| RSF1 | transcription regulator | | 1,88E-02 | BCL2 |
| PU-H71 | chemical drug | | 1,88E-02 | SYK |
| Vla-4 | complex | | 1,88E-02 | BCL2 |
| benzenesulfonic acid | chemical reagent | | 1,88E-02 | CD86 |
| CSF | group | | 1,88E-02 | C3 |
| Mucin | group | | 1,88E-02 | CD86 |
| MAP4K1 | kinase | | 1,88E-02 | BCL2 |
| CD6 | transmembrane receptor | | 1,88E-02 | FOXP3 |
| RIDA | enzyme | | 1,88E-02 | IL10 |
| IL31RA | transmembrane receptor | | 1,88E-02 | SOCS3 |
| LUCAT1 | other | | 1,88E-02 | BCL2 |
| MCF2 | other | | 1,88E-02 | ITGB1 |
| RNF34 | enzyme | | 1,88E-02 | BCL2 |
| GAS7 | transcription regulator | | 1,88E-02 | ITGB1 |
| ADAR | enzyme | | 1,88E-02 | SPI1 |
| APAF1 | other | | 1,88E-02 | BCL2 |
| LSP1 | other | | 1,88E-02 | ITGB1 |
| TIMP4 | other | | 1,88E-02 | BCL2 |
| ELMO1 | other | | 1,88E-02 | IL10 |
| ARG2 | enzyme | | 1,88E-02 | BCL2 |
| NRP2 | kinase | | 1,88E-02 | BCL2 |
| CD59 | other | | 1,88E-02 | BCL2 |
| NME3 | kinase | | 1,88E-02 | ITGB1 |
| TFDP2 | transcription regulator | | 1,88E-02 | STAG3 |
| beta-elemene | chemical - endogenous non-mammalian | | 1,88E-02 | ZEB2 |
| ADAM9 | peptidase | | 1,88E-02 | ITGB1 |
| Mcpt1 | peptidase | | 1,88E-02 | IL10 |
| STK17B | kinase | | 1,88E-02 | BCL2 |
| CLDN2 | other | | 1,88E-02 | ITGB1 |
| HG-9-91-01 | chemical reagent | | 1,88E-02 | IL10 |

| | | | | |
|---|---|---|---|---|
| ampicillin | chemical drug | | 1,88E-02 | IL10 |
| dextroamphetamine | chemical drug | | 1,88E-02 | FOSB |
| lindane | chemical drug | | 1,88E-02 | BCL2 |
| atrasentan | chemical drug | | 1,88E-02 | BCL2 |
| Collagen type V | complex | | 1,88E-02 | ITGB1 |
| Z-VRPR-FMK | chemical - protease inhibitor | | 1,88E-02 | IL10 |
| 2-butenal | chemical toxicant | | 1,88E-02 | IL10 |
| 3-hydroxymorphinan | chemical - endogenous mammalian | | 1,88E-02 | SORT1 |
| CB-PIC | chemical reagent | | 1,88E-02 | BCL2 |
| ampelopsin | chemical drug | | 1,88E-02 | BCL2 |
| azelaic acid | chemical - endogenous mammalian | | 1,88E-02 | BCL2 |
| phloroglucinol | chemical drug | | 1,88E-02 | BCL2 |
| chlorine | chemical toxicant | | 1,88E-02 | ALOX5 |
| dideoxyadenosine | chemical toxicant | | 1,88E-02 | IL10 |
| 5-hydroxytryptophan | chemical - endogenous mammalian | | 1,88E-02 | BCL2 |
| lactose | chemical - endogenous mammalian | | 1,88E-02 | CAMP |
| heme | chemical - endogenous mammalian | | 1,92E-02 | CD86,IL10 |
| TNFSF11 | cytokine | 0,747 | 1,95E-02 | BCL2,CD300LF,IL10,SOCS3 |
| SERPINF1 | other | | 1,98E-02 | BCL2,IL10 |
| PTGER4 | G-protein coupled receptor | | 2,02E-02 | FOXP3,IFI16,IL10 |
| MAP2K4 | kinase | | 2,05E-02 | BCL2,CD86 |
| GPER1 | G-protein coupled receptor | | 2,05E-02 | FOSB,LTF |
| CCL11 | cytokine | | 2,05E-02 | BCL2,ITGB1 |
| fulvestrant | chemical drug | 1,868 | 2,10E-02 | BCL2,C3,ITGB1,LTF |
| PRKCB | kinase | | 2,12E-02 | CD86,IL10 |
| telmisartan | chemical drug | | 2,12E-02 | BCL2,ITGAX |
| pravastatin | chemical drug | | 2,12E-02 | FOXP3,IL10 |
| emodin | chemical drug | | 2,12E-02 | BCL2,CD86 |
| 17-alpha-ethinylestradiol | chemical drug | | 2,12E-02 | C3,IFI16,SPOCK2 |
| CREM | transcription regulator | | 2,12E-02 | BCL2,CAMP,CD86 |
| Pam3-Cys-Ser-Lys4 | chemical reagent | | 2,16E-02 | CD86,IL10,SPI1 |
| mir-181 | microRNA | | 2,18E-02 | BCL2,ZEB2 |
| thioctic acid | chemical drug | | 2,18E-02 | BCL2,IL10 |
| medroxyprogesterone acetate | chemical drug | | 2,23E-02 | BCL2,IL10,ITGB1 |
| TNFRSF8 | transmembrane receptor | | 2,25E-02 | IFI16,IL10 |
| SCD | enzyme | | 2,25E-02 | BCL2,PRDM16 |
| ELK1 | transcription regulator | | 2,25E-02 | FOSB,ITGB1 |
| CUX1 | transcription regulator | | 2,25E-02 | CEBPE,LTF |
| clozapine | chemical drug | | 2,25E-02 | IL10,PRDM16 |
| N-acetylmuramyl-L-alanyl-D-isoglutamine | chemical - endogenous non-mammalian | | 2,25E-02 | CD86,IL10 |
| Iberiotoxin | chemical reagent | | 2,26E-02 | BCL2 |
| VAV | group | | 2,26E-02 | IL10 |
| F Actin | complex | | 2,26E-02 | CD86 |
| P110 | group | | 2,26E-02 | BCL2 |
| ficusin | chemical drug | | 2,26E-02 | ITGB1 |
| blinatumomab | biologic drug | | 2,26E-02 | IL10 |
| givinostat | chemical drug | | 2,26E-02 | BCL2 |
| benzyladenine | chemical - endogenous non-mammalian | | 2,26E-02 | IL10 |
| CLEC6A | transmembrane receptor | | 2,26E-02 | IL10 |
| RASSF3 | other | | 2,26E-02 | BCL2 |
| BPIFB1 | other | | 2,26E-02 | BCL2 |
| ZNF395 | other | | 2,26E-02 | IFI16 |
| Ap2 alpha | group | | 2,26E-02 | BCL2 |
| lumefantrine | chemical drug | | 2,26E-02 | BCL2 |
| LOC100288798 | other | | 2,26E-02 | MS4A3 |
| peptide T | biologic drug | | 2,26E-02 | IL10 |
| Cr3 | complex | | 2,26E-02 | CD86 |
| CASP6 | peptidase | | 2,26E-02 | IL10 |
| SAMD4A | translation regulator | | 2,26E-02 | PRDM16 |
| BAD | other | | 2,26E-02 | BCL2 |
| BCL11A | transcription regulator | | 2,26E-02 | BCL2 |
| ABCD2 | transporter | | 2,26E-02 | ALOX5 |
| PROK2 | other | | 2,26E-02 | BCL2 |
| entolimod | biologic drug | | 2,26E-02 | IL10 |
| (-)-arctigenin | chemical reagent | | 2,26E-02 | BCL2 |
| KCNIP2 | other | | 2,26E-02 | FOSB |
| CR1 | transmembrane receptor | | 2,26E-02 | C3 |
| FES | kinase | | 2,26E-02 | ITGAX |
| miR-219a-5p (and other miRNAs w/seed GAUUGUC) | mature microRNA | | 2,26E-02 | ALOX5 |
| mir-28 | microRNA | | 2,26E-02 | PDCD1 |
| miR-193a-5p (miRNAs w/seed GGGUCUU) | mature microRNA | | 2,26E-02 | IL10 |
| SOX18 | transcription regulator | | 2,26E-02 | BCL2 |
| ATP2B2 | transporter | | 2,26E-02 | BCL2 |
| LIMS2 | other | | 2,26E-02 | ITGB1 |
| GMFG | growth factor | | 2,26E-02 | ITGB1 |
| F13A1 | enzyme | | 2,26E-02 | IL10 |
| LRIG1 | other | | 2,26E-02 | BCL2 |
| DNAJA3 | other | | 2,26E-02 | BCL2 |
| BGLAP | other | | 2,26E-02 | PRDM16 |
| ATP6V0A2 | transporter | | 2,26E-02 | IL10 |

| | | | | | |
|---|---|---|---|---|---|
| ADCY5 | enzyme | | | 2,26E-02 | BCL2 |
| COL4A1 | other | | | 2,26E-02 | BCL2 |
| 19,20-epoxydocosapentaenoic acid | chemical - endogenous mammalian | | | 2,26E-02 | ZEB2 |
| CLEC4M | other | | | 2,26E-02 | C3 |
| Hrg | other | | | 2,26E-02 | IL10 |
| GNRH2 | other | | | 2,26E-02 | FOSB |
| NET1 | other | | | 2,26E-02 | ITGB1 |
| BCL2A1 | other | | | 2,26E-02 | BCL2 |
| ZYX | other | | | 2,26E-02 | ITGB1 |
| MIA-459 | chemical reagent | | | 2,26E-02 | BCL2 |
| MIA-313 | chemical reagent | | | 2,26E-02 | BCL2 |
| CIMO | chemical reagent | | | 2,26E-02 | BCL2 |
| Congo Red | chemical toxicant | | | 2,26E-02 | C3 |
| 6,7-dinitroquinoxaline-2,3-dione | chemical reagent | | | 2,26E-02 | FOSB |
| lestaurtinib | chemical drug | | | 2,26E-02 | CEBPE |
| nigericin | chemical drug | | | 2,26E-02 | PROM1 |
| etomidate | chemical drug | | | 2,26E-02 | IL10 |
| metronidazole | chemical drug | | | 2,26E-02 | IL10 |
| 3-hydroxyanthranilic acid | chemical - endogenous mammalian | | | 2,26E-02 | FOXP3 |
| FC-99 | chemical reagent | | | 2,26E-02 | CD86 |
| beta-aminoarteether | chemical drug | | | 2,26E-02 | IL10 |
| ZFA-fmk | chemical reagent | | | 2,26E-02 | C3 |
| propyl-2-(8-(3,4-difluorobenzyl)-2',5'-dioxo-8-azaspiro[bicyclo[3.2.1]octane-3,4'-imidazolidine]-1'-yl)acetate | chemical reagent | | | 2,26E-02 | BCL2 |
| scopoletin | chemical - endogenous non-mammalian | | | 2,26E-02 | IL10 |
| heroin | chemical drug | | | 2,26E-02 | FOSB |
| alpha-hydroxyglutarate | chemical - endogenous mammalian | | | 2,26E-02 | ZEB2 |
| CV 3988 | chemical reagent | | | 2,26E-02 | ITGB1 |
| sparfosic acid | chemical drug | | | 2,26E-02 | BCL2 |
| drospirenone | chemical drug | | | 2,26E-02 | PRDM16 |
| bisphenol A | chemical - endogenous mammalian | | | 2,27E-02 | ARNT2,BCL2,C3 |
| SIN3A | transcription regulator | | | 2,32E-02 | BCL2,FOXP3 |
| CSHL1 | growth factor | | | 2,32E-02 | SOCS3,TOX |
| seocalcitol | chemical drug | | | 2,32E-02 | BCL2,ITGA7 |
| sodium arsenite | chemical drug | | | 2,32E-02 | BCL2,SOCS3 |
| CD38 | enzyme | | | 2,34E-02 | CD86,CKAP4,SOCS3 |
| geldanamycin | chemical - endogenous non-mammalian | Activated | 2 | 2,38E-02 | BCL2,IFI16,ILDR1,ITGB1 |
| TNFRSF1A | transmembrane receptor | | | 2,38E-02 | CD86,IL10,SOCS3 |
| sunitinib | chemical drug | | | 2,39E-02 | ITGB1,PDCD1 |
| IL25 | cytokine | | | 2,39E-02 | FOSB,IL10 |
| PPP3R1 | phosphatase | | | 2,39E-02 | ABCA13,GBP3 |
| HIF1A | transcription regulator | | -0,873 | 2,43E-02 | BCL2,HP,IL10,PROM1,SOCS3 |
| estrogen | chemical drug | | -1,279 | 2,43E-02 | BCL2,IL10,ITGB1,LTF |
| IL5 | cytokine | | -1 | 2,46E-02 | BCL2,CKAP4,CX3CR1,IL10 |
| NR4A3 | ligand-dependent nuclear receptor | | | 2,46E-02 | FOXP3,IL10 |
| dihydrotestosterone | chemical - endogenous mammalian | | 0,139 | 2,47E-02 | BCL2,C1orf21,FOXP3,IL10,ITGB1,SORT1 |
| nitrofurantoin | chemical drug | | | 2,54E-02 | C3,IL10,ITGB1 |
| 12-(3-adamantan-1-yl-ureido) dodecanoic acid | chemical reagent | | | 2,54E-02 | BCL2,IL10 |
| NRG2 | growth factor | | | 2,54E-02 | BCL2,FOSB |
| MUC1 | other | | | 2,54E-02 | CD86,IL10 |
| azoxymethane | chemical toxicant | | | 2,54E-02 | BCL2,IL10 |
| Go 6976 | chemical - kinase inhibitor | | | 2,54E-02 | IL10,ITGB1 |
| infliximab | biologic drug | | | 2,61E-02 | CTLA4,IL12RB2 |
| AIRE | transcription regulator | | | 2,61E-02 | FOXP3,LTF |
| MBD2 | transcription regulator | | | 2,61E-02 | FOXP3,PROM1 |
| CD3E | transmembrane receptor | | | 2,61E-02 | CTLA4,IL10 |
| 15-epi-lipoxin A4 | chemical - endogenous mammalian | | | 2,63E-02 | CAMP |
| glucosylceramide | chemical - endogenous mammalian | | | 2,63E-02 | IL10 |
| coconut oil | chemical drug | | | 2,63E-02 | HP |
| bis(3',5')-cyclic diguanylic acid | chemical - endogenous non-mammalian | | | 2,63E-02 | CD86 |
| GPRC6A | G-protein coupled receptor | | | 2,63E-02 | PRDM16 |
| SLC5A8 | transporter | | | 2,63E-02 | BCL2 |
| saracatinib | chemical drug | | | 2,63E-02 | IL10 |
| TH2 Cytokine | group | | | 2,63E-02 | SOCS3 |
| ganetespib | chemical drug | | | 2,63E-02 | BCL2 |
| catumaxomab | biologic drug | | | 2,63E-02 | PDCD1 |
| urotensin II | biologic drug | | | 2,63E-02 | ALOX5 |
| Collagen type III | complex | | | 2,63E-02 | ITGB1 |
| HLA-DQ | complex | | | 2,63E-02 | IL10 |
| IER3 | other | | | 2,63E-02 | BCL2 |
| PRKG2 | kinase | | | 2,63E-02 | PROM1 |
| STIM2 | transporter | | | 2,63E-02 | IL10 |
| STRA6 | transporter | | | 2,63E-02 | SOCS3 |

| | | | |
|---|---|---|---|
| SWAP70 | other | 2,63E-02 | CD86 |
| RBX1 | enzyme | 2,63E-02 | BCL2 |
| MACC1-AS1 | other | 2,63E-02 | PROM1 |
| PTBP3 | other | 2,63E-02 | ZEB2 |
| CTSE | peptidase | 2,63E-02 | CD86 |
| TPSD1 | peptidase | 2,63E-02 | IL10 |
| SH3RF1 | enzyme | 2,63E-02 | MAP3K9 |
| PPM1A | phosphatase | 2,63E-02 | ITGB1 |
| RAD9A | enzyme | 2,63E-02 | ITGB1 |
| SHCBP1 | other | 2,63E-02 | PROM1 |
| TNKS | enzyme | 2,63E-02 | SORT1 |
| UBASH3A | enzyme | 2,63E-02 | IL10 |
| casticin | chemical - endogenous non-mammalian | 2,63E-02 | BCL2 |
| MS4A1 | other | 2,63E-02 | CD86 |
| GLP1R | G-protein coupled receptor | 2,63E-02 | CAMP |
| TSPO | transmembrane receptor | 2,63E-02 | BCL2 |
| LTC4S | enzyme | 2,63E-02 | IL10 |
| Klrk1 | transmembrane receptor | 2,63E-02 | IL10 |
| H4C3 | other | 2,63E-02 | FOSB |
| triacetyl-3-hydroxyphenyladenosine | chemical reagent | 2,63E-02 | IL10 |
| nicorandil | chemical drug | 2,63E-02 | BCL2 |
| selegiline | chemical drug | 2,63E-02 | BCL2 |
| phytoestrogen | chemical drug | 2,63E-02 | LTF |
| BW 245C | chemical reagent | 2,63E-02 | IFI16 |
| CHS-828 | chemical drug | 2,63E-02 | PROM1 |
| gliotoxin | chemical toxicant | 2,63E-02 | BCL2 |
| diacerein | chemical drug | 2,63E-02 | BCL2 |
| eugenol | chemical - endogenous non-mammalian | 2,63E-02 | IL10 |
| ferric chloride | chemical toxicant | 2,63E-02 | IL10 |
| BAR501 | chemical reagent | 2,63E-02 | IL10 |
| miR-124 mimic | chemical reagent | 2,63E-02 | BCL2 |
| Boc-D-FMK | chemical reagent | 2,63E-02 | SYK |
| murabutide | chemical reagent | 2,63E-02 | CD86 |
| rutin | chemical toxicant | 2,63E-02 | BCL2 |
| citric acid | chemical - endogenous mammalian | 2,63E-02 | BCL2 |
| non-esterified fatty acid | chemical - endogenous mammalian | 2,63E-02 | IL10 |
| sivelestat | chemical drug | 2,63E-02 | IL10 |
| Salmonella enterica serotype abortus equi lipopolysaccharide | chemical toxicant | 2,66E-02 | GBP3,IL10,SOCS3 |
| CIP2A | other | 2,68E-02 | BCL2,PDE2A |
| TBP | transcription regulator | 2,68E-02 | BCL2,FOXP3 |
| cytarabine | chemical drug | 2,68E-02 | BCL2,CD86 |
| IGF2 | growth factor | 2,70E-02 | IL10,PRDM16,SOCS3 |
| DNMT3A | enzyme | 2,70E-02 | FOXP3,PDE2A,SOCS3 |
| pioglitazone | chemical drug | 2,74E-02 | BCL2,CD86,FOXP3 |
| Igm | complex | 2,76E-02 | BCL2,IL10 |
| Pro-inflammatory Cytokine | group | 2,76E-02 | HP,SOCS3 |
| Map3k7 | kinase | 2,76E-02 | IL10,PROM1 |
| caffeine | chemical drug | 2,76E-02 | BCL2,FOSB |
| RXRA | ligand-dependent nuclear receptor | 2,77E-02 | C3,CEBPE,IL10,LTF |
| MAPK8 | kinase | 2,78E-02 | BCL2,IL10,MAP3K9 |
| RARB | ligand-dependent nuclear receptor | 2,82E-02 | GJB4,HP,LTF |
| FASN | enzyme | 2,83E-02 | ALOX5,IL10 |
| 2-deoxyglucose | chemical drug | 2,91E-02 | BCL2,SPI1 |
| DDX58 | enzyme | 2,99E-02 | GBP3,SOCS3 |
| GLI3 | transcription regulator | 2,99E-02 | BCL2,FOSB |
| ID1 | transcription regulator | 2,99E-02 | IFI16,PROM1 |
| ITGB3 | transmembrane receptor | 2,99E-02 | BCL2,IL10 |
| IKZF3 | transcription regulator | 2,99E-02 | ALOX5,BCL2 |
| entinostat | chemical drug | 2,99E-02 | BCL2,ITGAX |
| gemfibrozil | chemical drug | 2,99E-02 | FOXP3,SOCS3 |
| ganglioside GD1a | chemical - endogenous mammalian | 3,00E-02 | IL10 |
| rhodamine 6G | chemical toxicant | 3,00E-02 | BCL2 |
| tocilizumab | biologic drug | 3,00E-02 | IL10 |
| ibudilast | chemical drug | 3,00E-02 | IL10 |
| VTCN1 | other | 3,00E-02 | IL10 |
| CLCF1 | cytokine | 3,00E-02 | SOCS3 |
| ARID5A | transcription regulator | 3,00E-02 | IL10 |
| HDAC11 | transcription regulator | 3,00E-02 | IL10 |
| SULT2B1 | enzyme | 3,00E-02 | BCL2 |
| IL26 | cytokine | 3,00E-02 | IL10 |
| borage oil | chemical drug | 3,00E-02 | IL10 |
| collagen | group | 3,00E-02 | IL10 |
| ABCC1 | transporter | 3,00E-02 | BCL2 |
| LITAF | transcription regulator | 3,00E-02 | IL10 |
| immune complex | complex | 3,00E-02 | SOCS3 |
| HINT1 | enzyme | 3,00E-02 | BCL2 |
| GRK5 | kinase | 3,00E-02 | BCL2 |
| VCL | enzyme | 3,00E-02 | ITGB1 |
| IL23R | transmembrane receptor | 3,00E-02 | IL10 |
| CD82 | other | 3,00E-02 | IL10 |
| IL22RA2 | transmembrane receptor | 3,00E-02 | SOCS3 |

| | | | | |
|---|---|---|---|---|
| CXCR1 | G-protein coupled receptor | | 3,00E-02 | BCL2 |
| miR-192-5p (and other miRNAs w/seed UGACCUA) | mature microRNA | | 3,00E-02 | ZEB2 |
| DKK2 | other | | 3,00E-02 | BCL2 |
| GAL3ST1 | enzyme | | 3,00E-02 | ITGB1 |
| CYSLTR1 | G-protein coupled receptor | | 3,00E-02 | BCL2 |
| GSN | other | | 3,00E-02 | BCL2 |
| ponesimod | chemical drug | | 3,00E-02 | IL10 |
| GSC | transcription regulator | | 3,00E-02 | ZEB2 |
| MAOA | enzyme | | 3,00E-02 | BCL2 |
| APCS | other | | 3,00E-02 | IL10 |
| LAMA4 | enzyme | | 3,00E-02 | ITGB1 |
| CALCB | other | | 3,00E-02 | SPI1 |
| MAD2L1 | other | | 3,00E-02 | BCL2 |
| MAP3K10 | kinase | | 3,00E-02 | MAP3K9 |
| LY86 | other | | 3,00E-02 | CD86 |
| PPP1CA | phosphatase | | 3,00E-02 | IL10 |
| NEU3 | enzyme | | 3,00E-02 | BCL2 |
| CCX771 | chemical reagent | | 3,00E-02 | ITGB1 |
| teasaponin | chemical - endogenous non-mammalian | | 3,00E-02 | SOCS3 |
| DIABLO | other | | 3,00E-02 | PDCD1 |
| Havcr1 | other | | 3,00E-02 | IL10 |
| CCL7 | cytokine | | 3,00E-02 | IL10 |
| TBL1XR1 | transcription regulator | | 3,00E-02 | HP |
| CCL-34 | chemical reagent | | 3,00E-02 | ITGAX |
| MM-401 | chemical reagent | | 3,00E-02 | BCL2 |
| itraconazole | chemical drug | | 3,00E-02 | CAMP |
| methoxychlor | chemical toxicant | | 3,00E-02 | LTF |
| bisperoxovanadium 1,10-phenanthroline | chemical reagent | | 3,00E-02 | IL10 |
| PCM1-JAK2 | fusion gene/product | | 3,00E-02 | SOCS3 |
| GSKJ4 | chemical reagent | | 3,00E-02 | CD86 |
| astragaloside IV | chemical - endogenous non-mammalian | | 3,00E-02 | FOXP3 |
| aplidine | biologic drug | | 3,00E-02 | FOSB |
| alpha-tocopherol succinate | chemical drug | | 3,00E-02 | BCL2 |
| cordycepin | chemical drug | | 3,00E-02 | IL10 |
| N,N-dimethylarginine | chemical - endogenous mammalian | | 3,00E-02 | BCL2 |
| gefitinib | chemical drug | | 3,03E-02 | BCL2,C3,LTF |
| silibinin | chemical drug | | 3,06E-02 | PROM1,ZEB2 |
| diethylstilbestrol | chemical drug | -1,235 | 3,07E-02 | C3,IGSF6,LTF,PROM1 |
| MYB | transcription regulator | | 3,08E-02 | BCL2,C3,PDE2A |
| FOXO1 | transcription regulator | 0,882 | 3,10E-02 | BCL2,FOSB,FOXP3,IKZF2,PDCD1 |
| AGER | transmembrane receptor | | 3,14E-02 | BCL2,IL10 |
| methamphetamine | chemical drug | | 3,22E-02 | BCL2,FOSB |
| acetaminophen | chemical drug | | 3,25E-02 | BCL2,ITGB1,SOCS3 |
| metformin | chemical drug | | 3,25E-02 | BCL2,IL10,SORT1 |
| ETV6-RUNX1 | fusion gene/product | 0 | 3,26E-02 | ALOX5,IFI16,ITGB1,PROM1 |
| E. coli serotype 0127B8 lipopolysaccharide | chemical - endogenous non-mammalian | | 3,30E-02 | IL10,NOTCH3,SOCS3 |
| corticosteroid | chemical drug | | 3,30E-02 | IL10,MS4A3 |
| miR-141-3p (and other miRNAs w/seed AACACUG) | mature microRNA | | 3,30E-02 | ITGB1,ZEB2 |
| RELB | transcription regulator | | 3,30E-02 | BCL2,IL10 |
| decitabine | chemical drug | -1,812 | 3,34E-02 | ALOX5,CX3CR1,IFI16,SOCS3,SPI1,STAG3,SYK |
| 15-deoxy-delta-12,14 -PGJ 2 | chemical - endogenous mammalian | | 3,34E-02 | BCL2,IL10,SOCS3 |
| tannic acid | chemical toxicant | | 3,36E-02 | BCL2 |
| phosphatidylcholine | chemical - endogenous mammalian | | 3,36E-02 | BCL2 |
| ganglioside GD3 | chemical - endogenous mammalian | | 3,36E-02 | IL10 |
| RUNX1T1 | transcription regulator | | 3,36E-02 | SPI1 |
| SLC11A1 | transporter | | 3,36E-02 | IL10 |
| abatacept | biologic drug | | 3,36E-02 | PDCD1 |
| bazedoxifene | chemical drug | | 3,36E-02 | BCL2 |
| 4-nonylphenol | chemical toxicant | | 3,36E-02 | IL10 |
| TGFBR | group | | 3,36E-02 | SOCS3 |
| ZFAS1 | other | | 3,36E-02 | ZEB2 |
| S6K1 | group | | 3,36E-02 | IL10 |
| IRF3 dimer | complex | | 3,36E-02 | IL10 |
| BCG vaccine | biologic drug | | 3,36E-02 | IL10 |
| propargylamine | chemical reagent | | 3,36E-02 | BCL2 |
| SASH3 | other | | 3,36E-02 | IL10 |
| salmonella typhimurium lipopolysaccharide | chemical - endogenous non-mammalian | | 3,36E-02 | CD86 |
| (¬±)-2-hydroxyoleic acid | chemical drug | | 3,36E-02 | CAMP |
| LRRC32 | other | | 3,36E-02 | FOXP3 |
| CD80/CD86 | group | | 3,36E-02 | CTLA4 |
| ABCD1 | transporter | | 3,36E-02 | ALOX5 |
| UNC5B | transmembrane receptor | | 3,36E-02 | IL10 |
| SERPINB2 | other | | 3,36E-02 | BCL2 |
| LIMS1 | other | | 3,36E-02 | ITGB1 |
| MYDGF | cytokine | | 3,36E-02 | IL10 |
| RPS6KB2 | kinase | | 3,36E-02 | BCL2 |

| | | | | |
|---|---|---|---|---|
| Ighg2b | other | | 3,36E-02 | IL10 |
| ERP29 | transporter | | 3,36E-02 | ZEB2 |
| NACC1 | transcription regulator | | 3,36E-02 | BCL2 |
| PANDAR | other | | 3,36E-02 | BCL2 |
| UBA7 | enzyme | | 3,36E-02 | IL10 |
| USP37 | peptidase | | 3,36E-02 | CEBPE |
| GPR183 | G-protein coupled receptor | | 3,36E-02 | BCL2 |
| di-spiropyrrolizidino oxindole andrographolide derivative CY2 | chemical reagent | | 3,36E-02 | BCL2 |
| CD99 | other | | 3,36E-02 | FOSB |
| XCL1 | cytokine | | 3,36E-02 | IL10 |
| LGALS9B | other | | 3,36E-02 | IL10 |
| USP17L2 (includes others) | peptidase | | 3,36E-02 | PROM1 |
| DEK | transcription regulator | | 3,36E-02 | CEBPE |
| 1,1-bis(3'-indolyl)-1-(4-hydroxyphenyl)methane | chemical reagent | | 3,36E-02 | BCL2 |
| cyclic guanosine monophosphate-adenosine monophosphate | chemical - endogenous non-mammalian | | 3,36E-02 | CD86 |
| UCA1 | other | | 3,36E-02 | BCL2 |
| niflumic acid | chemical drug | | 3,36E-02 | CD86 |
| leflunomide | chemical drug | | 3,36E-02 | FOXP3 |
| dantrolene | chemical drug | | 3,36E-02 | BCL2 |
| benazepril | chemical drug | | 3,36E-02 | BCL2 |
| brimonidine | chemical drug | | 3,36E-02 | BCL2 |
| PD 168393 | chemical - kinase inhibitor | | 3,36E-02 | BCL2 |
| RP 73401 | chemical toxicant | | 3,36E-02 | CEBPE |
| prodigiosin | chemical toxicant | | 3,36E-02 | BCL2 |
| 8-hydroxy-2-(di-n-propylamino)tetralin | chemical reagent | | 3,36E-02 | FOSB |
| rhodioloside | chemical - endogenous non-mammalian | | 3,36E-02 | BCL2 |
| miR-182 inhibitor | chemical reagent | | 3,36E-02 | BCL2 |
| 2,4-dinitrobenzenesulfonic acid | chemical reagent | | 3,36E-02 | CD86 |
| iodine | chemical - endogenous mammalian | | 3,36E-02 | BCL2 |
| tyrphostin AG 127 | chemical - kinase inhibitor | | 3,36E-02 | BCL2 |
| oleoylethanolamide | chemical - endogenous mammalian | | 3,36E-02 | FOSB |
| arachidic acid | chemical - endogenous mammalian | | 3,36E-02 | IL10 |
| saturated fatty acid | chemical - other | | 3,36E-02 | IL10 |
| Il3 | cytokine | | 3,38E-02 | BCL2,LTF |
| ATG7 | enzyme | | 3,38E-02 | IL10,SOCS3 |
| SOX2 | transcription regulator | -0,625 | 3,46E-02 | ALOX5,GBP3,NOTCH3,PROM1,S1PR5 |
| MAP2K6 | kinase | | 3,47E-02 | BCL2,CD86 |
| FOSL1 | transcription regulator | | 3,47E-02 | FOSB,IL10 |
| 1-methyl-4-phenyl-1,2,3,6-tetrahydropyridine | chemical toxicant | | 3,55E-02 | BCL2,FOSB |
| rottlerin | chemical toxicant | | 3,55E-02 | IL10,ITGB1 |
| mifepristone | chemical drug | 0 | 3,56E-02 | BCL2,C3,IL10,LTF |
| PRKCD | kinase | | 3,58E-02 | BCL2,IL10,PROM1 |
| enterotoxin B | biologic drug | | 3,63E-02 | CTLA4,IL10 |
| MMP9 | peptidase | | 3,63E-02 | IL10,ITGB1 |
| PTPN11 | phosphatase | | 3,63E-02 | LTF,SOCS3 |
| NFKB2 | transcription regulator | | 3,63E-02 | BCL2,CD86 |
| ciglitazone | chemical drug | | 3,63E-02 | BCL2,IL10 |
| (+)-MK-801 | chemical drug | | 3,63E-02 | FOSB,IL10 |
| PIN1 | enzyme | | 3,72E-02 | NOTCH3,PDCD1 |
| C3 | peptidase | | 3,72E-02 | C3,IL10 |
| doxycycline | chemical drug | | 3,72E-02 | CD86,ITGAX |
| IGF1 | growth factor | -1,498 | 3,73E-02 | BCL2,CAMP,FOSB,IL10,SOCS3 |
| beraprost | chemical drug | | 3,73E-02 | CD86 |
| EPHB2 | kinase | | 3,73E-02 | BCL2 |
| PI3KŒ≥ | group | | 3,73E-02 | IL10 |
| niclosamide | chemical drug | | 3,73E-02 | BCL2 |
| HOXA11-AS | other | | 3,73E-02 | ZEB2 |
| HULC | other | | 3,73E-02 | ITGB1 |
| CAMK2N2 | other | | 3,73E-02 | BCL2 |
| ANKRD17 | other | | 3,73E-02 | SOCS3 |
| NQO2 | enzyme | | 3,73E-02 | BCL2 |
| Shc | group | | 3,73E-02 | BCL2 |
| TRAF3IP3 | other | | 3,73E-02 | FOXP3 |
| L2HGDH | enzyme | | 3,73E-02 | ITGAX |
| Sox2ot | other | | 3,73E-02 | PROM1 |
| necrostatin-1 | chemical reagent | | 3,73E-02 | BCL2 |
| BMS-754807 | chemical drug | | 3,73E-02 | BCL2 |
| ITGB5 | other | | 3,73E-02 | IL10 |
| PF-3084014 | chemical drug | | 3,73E-02 | BCL2 |
| EEF1A2 | translation regulator | | 3,73E-02 | IL10 |
| ANG | enzyme | | 3,73E-02 | BCL2 |
| DCLK1 | kinase | | 3,73E-02 | ZEB2 |
| PDGFA | growth factor | | 3,73E-02 | PROM1 |

| | | | | |
|---|---|---|---|---|
| PDK2 | kinase | | 3,73E-02 | IL10 |
| mir-503 | microRNA | | 3,73E-02 | BCL2 |
| CDK6 | kinase | | 3,73E-02 | IL10 |
| PIM3 | kinase | | 3,73E-02 | IL12RB2 |
| SLC6A1 | transporter | | 3,73E-02 | BCL2 |
| CXCL13 | cytokine | | 3,73E-02 | IL10 |
| LAT2 | other | | 3,73E-02 | IL10 |
| PTGER3 | G-protein coupled receptor | | 3,73E-02 | IL10 |
| ARID4B | transcription regulator | | 3,73E-02 | FOXP3 |
| gamma-secretase inhibitor XX | chemical reagent | | 3,73E-02 | NOTCH3 |
| premarin | chemical drug | | 3,73E-02 | BCL2 |
| nedocromil sodium | chemical drug | | 3,73E-02 | IL10 |
| trifluoperazine | chemical drug | | 3,73E-02 | PROM1 |
| piroxicam | chemical drug | | 3,73E-02 | IL10 |
| levamisole | chemical drug | | 3,73E-02 | BCL2 |
| midazolam | chemical drug | | 3,73E-02 | CD86 |
| polymyxin B | biologic drug | | 3,73E-02 | IL10 |
| formononetin | chemical - endogenous non-mammalian | | 3,73E-02 | CD86 |
| nicotine | chemical drug | | 3,77E-02 | BCL2,CD86,IL10 |
| CXCL8 | cytokine | | 3,80E-02 | BCL2,ITGAX |
| MXD1 | transcription regulator | | 3,80E-02 | ITGAX,WDFY4 |
| PPP3CA | phosphatase | | 3,80E-02 | BCL2,C3 |
| apigenin | chemical - endogenous non-mammalian | | 3,80E-02 | BCL2,IL10 |
| CpG ODN 1826 | chemical reagent | | 3,80E-02 | CD86,IL10 |
| dehydroisoandrosterone | chemical - endogenous mammalian | | 3,80E-02 | BCL2,IL10 |
| fluoxetine | chemical drug | | 3,89E-02 | FOSB,IL10 |
| PP2/AG1879 tyrosine kinase inhibitor | chemical - kinase inhibitor | | 3,97E-02 | BCL2,IL10 |
| TRAF2 | enzyme | | 3,97E-02 | ITGB1,MPEG1 |
| cobalt chloride | chemical reagent | | 3,97E-02 | BCL2,PROM1 |
| AICAR | chemical - endogenous mammalian | | 3,97E-02 | IL10,SOCS3 |
| spironolactone | chemical drug | | 3,97E-02 | BCL2,PRDM16 |
| zinc | chemical drug | | 3,97E-02 | CD86,IL10 |
| POU5F1 | transcription regulator | | 3,98E-02 | BCL2,GBP3,PROM1,ZEB2 |
| thapsigargin | chemical toxicant | | 4,01E-02 | CAMP,CD86,PDCD1 |
| PPARG | ligand-dependent nuclear receptor | 0,016 | 4,01E-02 | BCL2,C3,HP,IL10,SOCS3 |
| Z-LLL-CHO | chemical - protease inhibitor | 0 | 4,05E-02 | BCL2,ITGB1,NOTCH3,SORT1 |
| 4-hydroxyestradiol-17beta | chemical - endogenous mammalian | | 4,10E-02 | LTF |
| naringin | chemical - endogenous non-mammalian | | 4,10E-02 | BCL2 |
| Adaptor protein 1 | complex | | 4,10E-02 | IL10 |
| TLR2/3/4/9 | group | | 4,10E-02 | CD86 |
| ABCA4 | transporter | | 4,10E-02 | C3 |
| SNHG20 | other | | 4,10E-02 | ZEB2 |
| Traj18 | other | | 4,10E-02 | CD86 |
| Cebp | complex | | 4,10E-02 | IL10 |
| Mcpt4 | peptidase | | 4,10E-02 | BCL2 |
| MCL1 | transporter | | 4,10E-02 | BCL2 |
| NRBP2 | kinase | | 4,10E-02 | PROM1 |
| PDE3B | enzyme | | 4,10E-02 | SOCS3 |
| DRD1 | G-protein coupled receptor | | 4,10E-02 | FOSB |
| APOH | transporter | | 4,10E-02 | CD86 |
| LINC-ROR | other | | 4,10E-02 | BCL2 |
| IL17RB | transmembrane receptor | | 4,10E-02 | BCL2 |
| LNX2 | other | | 4,10E-02 | BCL2 |
| SART1 | other | | 4,10E-02 | PROM1 |
| PIKFYVE | kinase | | 4,10E-02 | CD86 |
| LB-205 | chemical reagent | | 4,10E-02 | BCL2 |
| ARID4A | transcription regulator | | 4,10E-02 | FOXP3 |
| CAMK2G | kinase | | 4,10E-02 | CEBPE |
| ENTPD1 | enzyme | | 4,10E-02 | CD86 |
| CHRNA3 | transmembrane receptor | | 4,10E-02 | BCL2 |
| CNB-001 | chemical reagent | | 4,10E-02 | ALOX5 |
| 2-[[9-isopropyl-6-[[4-(2-pyridyl)phenyl]methylamino]purin-2-yl]amino]butan-1-ol | chemical - kinase inhibitor | | 4,10E-02 | CD86 |
| CLEC4A | transmembrane receptor | | 4,10E-02 | IL10 |
| MAP3K11 | kinase | | 4,10E-02 | MAP3K9 |
| I-BET-151 | chemical reagent | | 4,10E-02 | FOXP3 |
| Ifnz (includes others) | cytokine | | 4,10E-02 | CD86 |
| oligomycin | chemical - endogenous non-mammalian | | 4,10E-02 | BCL2 |
| montelukast | chemical drug | | 4,10E-02 | IL10 |
| sevoflurane | chemical drug | | 4,10E-02 | BCL2 |
| bosutinib | chemical drug | | 4,10E-02 | IL10 |
| ferric ammonium citrate | chemical drug | | 4,10E-02 | C3 |
| carbamazepine | chemical drug | | 4,10E-02 | BCL2 |
| lidocaine | chemical drug | | 4,10E-02 | BCL2 |
| myelin oligodendrocyte glycoprotein (35-55) | chemical reagent | | 4,10E-02 | IL10 |

| | | | | |
|---|---|---|---|---|
| 7-(4-fluorobenzylamino)-1,3,4,8-tetrahydropyrrolo[4,3,2-de]quinolin-8(1H)-one | chemical reagent | | 4,10E-02 | BCL2 |
| pterostilbene | chemical drug | | 4,10E-02 | BCL2 |
| tin protoporphyrin IX | chemical reagent | | 4,10E-02 | CD86 |
| SS18-SSX2 | fusion gene/product | | 4,10E-02 | BCL2 |
| phospholipid | chemical - endogenous mammalian | | 4,10E-02 | ALOX5 |
| CA074-methyl ester | chemical reagent | | 4,10E-02 | BCL2 |
| delphinidin | chemical - endogenous non-mammalian | | 4,10E-02 | BCL2 |
| hesperetin | chemical drug | | 4,10E-02 | BCL2 |
| puerarin | chemical drug | | 4,10E-02 | BCL2 |
| desmosterol | chemical - endogenous mammalian | | 4,10E-02 | IL10 |
| trestolone | chemical drug | | 4,10E-02 | BCL2 |
| 5-O-mycolyl-beta-araf-(1->2)-5-O-mycolyl-alpha-araf-(1->1')-glycerol | chemical - endogenous non-mammalian | | 4,15E-02 | CX3CR1,SOCS3 |
| HMGB1 | transcription regulator | | 4,15E-02 | CD86,IL10 |
| histamine | chemical - endogenous mammalian | | 4,15E-02 | CD86,IL10 |
| IRF3 | transcription regulator | | 4,16E-02 | CD86,IFI16,IL10 |
| NPC1 | transporter | | 4,24E-02 | CAMP,ITGAX |
| MAFB | transcription regulator | | 4,24E-02 | IL10,OLFML2B |
| CCN1 | other | | 4,24E-02 | C3,ITGB1 |
| homocysteine | chemical - endogenous mammalian | | 4,24E-02 | BCL2,SOCS3 |
| wortmannin | chemical - kinase inhibitor | | 4,27E-02 | BCL2,IL10,SOCS3 |
| IL7R | transmembrane receptor | | 4,33E-02 | BCL2,FOXP3 |
| haloperidol | chemical drug | | 4,33E-02 | BCL2,FOSB |
| Vegf | group | -0,714 | 4,40E-02 | BCL2,FOSB,ITGB1,SOCS3,TBC1D8 |
| IKBKE | kinase | | 4,42E-02 | BCL2,IFI16 |
| miR-29b-3p (and other miRNAs w/seed AGCACCA) | mature microRNA | | 4,42E-02 | ADAMTS14,GAS7 |
| mir-29 | microRNA | | 4,42E-02 | ADAMTS14,BCL2 |
| TGFBR1 | kinase | | 4,42E-02 | CD86,FOXP3 |
| vancomycin | biologic drug | | 4,42E-02 | C3,IL10 |
| clobetasol propionate | chemical drug | | 4,46E-02 | CD86 |
| embelin | chemical - endogenous non-mammalian | | 4,46E-02 | BCL2 |
| ulipristal acetate | chemical drug | | 4,46E-02 | BCL2 |
| SOX2-OT | other | | 4,46E-02 | PROM1 |
| HRH1 | G-protein coupled receptor | | 4,46E-02 | IL10 |
| METTL3 | enzyme | | 4,46E-02 | SOCS3 |
| UCN2 | other | | 4,46E-02 | BCL2 |
| STAT1/3/5 dimer | complex | | 4,46E-02 | SOCS3 |
| HCAR1 | G-protein coupled receptor | | 4,46E-02 | IL10 |
| MFN2 | enzyme | | 4,46E-02 | BCL2 |
| AFAP1-AS1 | other | | 4,46E-02 | BCL2 |
| MTORC2 | complex | | 4,46E-02 | FOXP3 |
| STAR | transporter | | 4,46E-02 | CD86 |
| PAK4 | kinase | | 4,46E-02 | BCL2 |
| MALT1 | peptidase | | 4,46E-02 | IL10 |
| JUP | other | | 4,46E-02 | BCL2 |
| CCR7 | G-protein coupled receptor | | 4,46E-02 | CD86 |
| SH3KBP1 | other | | 4,46E-02 | CD86 |
| miR-451a (and other miRNAs w/seed AACCGUU) | mature microRNA | | 4,46E-02 | BCL2 |
| PLTP | enzyme | | 4,46E-02 | IL10 |
| PRAME | other | | 4,46E-02 | BCL2 |
| PDCD1LG2 | enzyme | | 4,46E-02 | IL10 |
| LMO4 | transcription regulator | | 4,46E-02 | HP |
| EFNB1 | other | | 4,46E-02 | BCL2 |
| ZBTB32 | transcription regulator | | 4,46E-02 | IL10 |
| PAWR | transcription regulator | | 4,46E-02 | BCL2 |
| TIGIT | other | | 4,46E-02 | IL10 |
| ACHE | enzyme | | 4,46E-02 | SPI1 |
| DOK1 | kinase | | 4,46E-02 | FOXP3 |
| CDH5 | other | | 4,46E-02 | BCL2 |
| AIM2 | other | | 4,46E-02 | IL10 |
| BAG3 | other | | 4,46E-02 | BCL2 |
| MAPKAPK3 | kinase | | 4,46E-02 | BCL2 |
| ELN | other | | 4,46E-02 | CD86 |
| CACNA1A | ion channel | | 4,46E-02 | IL10 |
| IL16 | cytokine | | 4,46E-02 | FOXP3 |
| BEX2 | other | | 4,46E-02 | BCL2 |
| CXCL1 | cytokine | | 4,46E-02 | SOCS3 |
| cantharidin | chemical drug | | 4,46E-02 | BCL2 |
| toremifene | chemical drug | | 4,46E-02 | CD86 |
| abscisic acid | chemical - endogenous non-mammalian | | 4,46E-02 | IL10 |
| quinacrine | chemical drug | | 4,46E-02 | BCL2 |
| indinavir | chemical drug | | 4,46E-02 | CEBPE |

| | | | | |
|---|---|---|---|---|
| D-sphingosine | chemical - endogenous mammalian | | 4,46E-02 | BCL2 |
| catecholamine | chemical - other | | 4,46E-02 | IL10 |
| nordihydroguaiaretic acid | chemical drug | | 4,46E-02 | BCL2 |
| zinostatin | biologic drug | | 4,46E-02 | BCL2 |
| cucurbitacin B | chemical - endogenous non-mammalian | | 4,46E-02 | BCL2 |
| diaminopimelic acid | chemical - endogenous non-mammalian | | 4,46E-02 | IL10 |
| Mt | group | | 4,46E-02 | BCL2 |
| androstenediol | chemical - endogenous mammalian | | 4,46E-02 | BCL2 |
| tert-butyl-hydroquinone | chemical reagent | | 4,51E-02 | BCL2,IL10 |
| ERBB2 | kinase | 0,776 | 4,53E-02 | BCL2,CHDH,HP,ITGB1,NOTCH3,PROM1,ZEB2 |
| atorvastatin | chemical drug | | 4,53E-02 | BCL2,FOSB,IL10 |
| DSCAML1 | other | | 4,60E-02 | NOTCH3,SRGAP3 |
| hemin | chemical - endogenous mammalian | | 4,60E-02 | FOXP3,IL10 |
| TRAF3 | enzyme | | 4,69E-02 | IL10,MPEG1 |
| SAMSN1 | other | | 4,69E-02 | SLFN12L,SOCS3 |
| BCR-ABL1 | fusion gene/product | | 4,69E-02 | BCL2,CEBPE |
| BMP4 | growth factor | | 4,69E-02 | BCL2,FOXP3,SPI1 |
| FN1 | enzyme | | 4,75E-02 | BCL2,ITGB1,SOCS3 |
| GW9662 | chemical reagent | | 4,78E-02 | BCL2,CD86 |
| D-alpha-tocopheryl succinate | chemical reagent | | 4,82E-02 | BCL2 |
| neomycin | chemical drug | | 4,82E-02 | IL10 |
| D-galactose | chemical - endogenous mammalian | | 4,82E-02 | BCL2 |
| ladostigil | chemical drug | | 4,82E-02 | BCL2 |
| SYK/ZAP | group | | 4,82E-02 | IL10 |
| mannosylated lipoarabinomannan | chemical - endogenous non-mammalian | | 4,82E-02 | IL10 |
| CLEC10A | other | | 4,82E-02 | IL10 |
| LILRA2 | other | | 4,82E-02 | IL10 |
| PLA2G2D | enzyme | | 4,82E-02 | ITGAX |
| GPBAR1 | G-protein coupled receptor | | 4,82E-02 | CAMP |
| harmine | chemical - endogenous non-mammalian | | 4,82E-02 | BCL2 |
| Ppp2c | group | | 4,82E-02 | BCL2 |
| SOX5 | transcription regulator | | 4,82E-02 | IL10 |
| mir-127 | microRNA | | 4,82E-02 | IL10 |
| PTGDR | G-protein coupled receptor | | 4,82E-02 | IFI16 |
| JARID2 | transcription regulator | | 4,82E-02 | ZEB2 |
| RPS6KA4 | kinase | | 4,82E-02 | IL10 |
| NLRC4 | other | | 4,82E-02 | CD86 |
| B4GALNT1 | enzyme | | 4,82E-02 | C3 |
| MMP8 | peptidase | | 4,82E-02 | IL10 |
| FRS2 | other | | 4,82E-02 | PROM1 |
| ALCAM | other | | 4,82E-02 | BCL2 |
| CRLF2 | transmembrane receptor | | 4,82E-02 | IL10 |
| PLA2G2E | enzyme | | 4,82E-02 | CAMP |
| VEGFD | growth factor | | 4,82E-02 | BCL2 |
| PTGER1 | G-protein coupled receptor | | 4,82E-02 | IL10 |
| 6-aminopyrazolopyrimidine derivative compound II | chemical - kinase inhibitor | | 4,82E-02 | IL10 |
| vatalanib | chemical drug | | 4,82E-02 | BCL2 |
| phencyclidine | chemical drug | | 4,82E-02 | BCL2 |
| aristolochic acid I | chemical toxicant | | 4,82E-02 | BCL2 |
| poly-L-lysine | chemical reagent | | 4,82E-02 | IL10 |
| thermozymocidin | chemical reagent | | 4,82E-02 | CD86 |
| palmitoleic acid | chemical - endogenous mammalian | | 4,82E-02 | IL10 |
| isatoribine | chemical reagent | | 4,82E-02 | CD86 |
| gambogic acid | chemical - endogenous non-mammalian | | 4,82E-02 | BCL2 |
| S-equol | chemical drug | | 4,82E-02 | CD86 |
| LDLR | transporter | | 4,91E-02 | CX3CR1,FOXP3,IL10 |
| SP3 | transcription regulator | | 4,91E-02 | FOXP3,IL10,IL12RB2 |
| isobutylmethylxanthine | chemical toxicant | | 4,97E-02 | IL10,NOTCH3,SYK |
| DMD | other | | 4,97E-02 | MAP3K9,MPEG1,SORT1 |
| RAF1 | kinase | | 4,97E-02 | BCL2,IL10,ITGB1 |
| PDLIM2 | other | | 4,97E-02 | BCL2,CRISPLD2 |
| miR-291a-3p (and other miRNAs w/seed AAGUGCU) | mature microRNA | | 4,97E-02 | C3,GBP3 |
| miR-125b-5p (and other miRNAs w/seed CCCUGAG) | mature microRNA | | 4,97E-02 | ALOX5,PROM1 |
| ATF2 | transcription regulator | | 4,97E-02 | BCL2,IL10 |
| trinitrobenzenesulfonic acid | chemical reagent | | 4,97E-02 | IL10,SOCS3 |
| testosterone | chemical - endogenous mammalian | -1,945 | 5,02E-02 | BCL2,FOSB,ITGB1,SYK |
| doxorubicin | chemical drug | 1,956 | 8,75E-02 | BCL2,CKAP4,FOXP3,NOTCH3 |
